# Metabolome of canine and human saliva: a non-targeted metabolomics study

**DOI:** 10.1007/s11306-020-01711-0

**Published:** 2020-08-25

**Authors:** Soile Turunen, Jenni Puurunen, Seppo Auriola, Arja M. Kullaa, Olli Kärkkäinen, Hannes Lohi, Kati Hanhineva

**Affiliations:** 1 School of Pharmacy, Faculty of Health Sciences, University of Eastern Finland, Kuopio, Finland; 2 Department of Veterinary Biosciences, and Department of Medical and Clinical Genetics, University of Helsinki, Helsinki, Finland; 3 Folkhälsan Research Center, Helsinki, Finland; 4 Institute of Dentistry, School of Medicine, Faculty of Health Sciences, University of Eastern Finland, Kuopio, Finland; 5 Institute of Public Health and Clinical Nutrition, Faculty of Health Sciences, University of Eastern Finland, Kuopio, Finland

**Keywords:** Saliva, Human, Dog, Lipid, Metabolomics, Liquid chromatography, Mass spectrometry

## Abstract

**Introduction:**

Saliva metabolites are suggested to reflect the health status of an individual in humans. The same could be true with the dog (*Canis lupus familiaris*), an important animal model of human disease, but its saliva metabolome is unknown. As a non-invasive sample, canine saliva could offer a new alternative material for research to reveal molecular mechanisms of different (patho)physiological stages, and for veterinary medicine to monitor dogs’ health trajectories.

**Objectives:**

To investigate and characterize the metabolite composition of dog and human saliva in a non-targeted manner.

**Methods:**

Stimulated saliva was collected from 13 privately-owned dogs and from 14 human individuals. We used a non-targeted ultra-high-performance liquid chromatography-quadrupole time-of-flight mass spectrometry (UHPLC-qTOF-MS) method to measure metabolite profiles from saliva samples.

**Results:**

We identified and classified a total of 211 endogenous and exogenous salivary metabolites. The compounds included amino acids, amino acid derivatives, biogenic amines, nucleic acid subunits, lipids, organic acids, small peptides as well as other metabolites, like metabolic waste molecules and other chemicals. Our results reveal a distinct metabolite profile of dog and human saliva as 25 lipid compounds were identified only in canine saliva and eight dipeptides only in human saliva. In addition, we observed large variation in ion abundance within and between the identified saliva metabolites in dog and human.

**Conclusion:**

The results suggest that non-targeted metabolomics approach utilizing UHPLC-qTOF-MS can detect a wide range of small compounds in dog and human saliva with partially overlapping metabolite composition. The identified metabolites indicate that canine saliva is potentially a versatile material for the discovery of biomarkers for dog welfare. However, this profile is not complete, and dog saliva needs to be investigated in the future with other analytical platforms to characterize the whole canine saliva metabolome. Furthermore, the detailed comparison of human and dog saliva composition needs to be conducted with harmonized study design.

**Electronic supplementary material:**

The online version of this article (10.1007/s11306-020-01711-0) contains supplementary material, which is available to authorized users.

## Introduction

Human saliva has been studied and characterized extensively in recent years. Most of the saliva is water (over 99%) containing a variety of electrolytes, different kinds of proteins as well as low molecular weight (< 1500 Da of mass) metabolites (Dame et al. 2015; Humphrey and Williamson 2001; Gardner et al. 2020). Mucus, epithelial and blood cells, food remainders and traces of medications or chemical products are also found in saliva (Aps and Martens 2005; Elmongy and Abdel-Rehim 2016). Moreover, biological material such as DNA and bacteria with their metabolites exist in saliva (Cuevas-Cordoba and Santiago-Garcia 2014).

Since saliva is rich in small molecules and given its role as “a mirror of the body”, there is a growing interest towards saliva usage as a non-invasive sample material for monitoring health trajectories to aid diagnosis or reveal the molecular mechanisms of disease pathologies. The same applies to domestic dogs which suffer from similar diseases to humans such as metabolic diseases, chronic inflammation, and cancers, manifested as diabetes (O'Kell et al. 2017), inflammatory bowel disease (Minamoto et al. 2015) and leukemia (Breen and Modiano 2008), respectively. Physiological similarity with humans and the large size of the canine have been reasons for the rise of these animals to one of the biomedical models alongside the rodents, for example in the study of genomics (Hytonen and Lohi 2016; van Steenbeek et al. 2016) and behavior (Puurunen et al. 2018). Despite the rising interest in dogs and saliva metabolomics, there is no data available for the canine saliva metabolome.

Humans share the same anatomy and salivary gland structure with dogs, except for dogs' zygomatic glands. The basic functions of saliva, such as lubrication, maintenance of oral homeostasis and dental welfare as well as bactericidal effects against pathogens, resemble each other (Dame et al. 2015; de Sousa-Pereira et al. 2015; Humphrey and Williamson 2001). Moreover, dogs use panting and evaporative cooling as the major function when exposed to heat and/or exercise (Goldberg et al. 1981). Differences between human and canine saliva have been revealed in the comparison of the proteome signature where, for example, cystatins with antimicrobial properties have been recognized in lower levels in the saliva of canines compared to saliva of humans (Sanguansermsri et al. 2018). In addition, different antimicrobial protein family members are identified in human and dog saliva, such as cathelicidin 1, cathelicidin antimicrobial peptide and CRISP1 in dog saliva, whereas cathelicidins were not detected in healthy humans but CRISP3 was (de Sousa-Pereira et al. 2015).

Several studies of the human salivary metabolome link it to various conditions, including oral and breast cancers (Sugimoto et al. 2010), type 2 diabetes (Barnes et al. 2014) and Sjögren’s syndrome (Mikkonen et al. 2013). Therefore, also the salivary metabolome of the dog could reflect the metabolic activity of canines’ oral cavity and total body. In this study, we compared the metabolome of dog and human saliva utilizing UHPLC-qTOF-MS -based non-targeted metabolomics approach. We aimed to identify a wide range of saliva metabolites to explore the metabolic profiles of both species and their overlap.

## Materials and methods

### Animals and human participants

Voluntary Finnish dog owners were recruited for the canine saliva donation. The saliva collection was conducted from 13 privately-owned dogs with the owners’ written consent and presence. The dogs were healthy referring no disease with one exception (cataract) and were not subjected to any drug treatment according to their owners. The breeds were Belgian Sheepdog (n = 2), Belgian Tervueren (n = 2), Weimaraner (n = 2), Rottweiler (n = 3), Golden Retriever (n = 2) and Flat-Coated Retriever (n = 2). The age of the dogs varied from 1.2 years to 9.3 years. The mean age was 5.5 years and SD 2.5 years. The number of males were 5 and females 8. Two of the female dogs were neutered.

Human saliva samples were collected from 14 healthy, non-smoking females, aged between 30 and 70 years (mean age 53 years, SD 11) who were recruited from the dental education clinic of Kuopio University Hospital. The volunteers had no recent history of systemic diseases or were not taking any medication. Inclusion criteria were healthy subjects, with normal excretion of saliva and no medications. Exclusion criteria were smokers, wearing removable dentures, having systemic diseases or medication, having a treatment history for cancer, or being incapable of communication. Out of all the patients examined, no males met these criteria. At the time of the study, every subject underwent an oral and dental examination performed by a dentist, and their oral health were good, no gingivitis, missing/broken teeth or caries.

### Collection of saliva samples

Canine saliva samples were collected between 9 to 11 a.m. by the same person at the dog’s home. The dogs were fasted and rested 12 h before sampling. Saliva was collected without causing any stress or harm to dogs as follows. Salivation was stimulated with prospect of food, i.e. the dog could see or sniff the treat but was not allowed to eat it. Saliva was collected under the tongue and from the surface of the mucosal lining of lips and cheek straight to 1.5 ml Eppendorf tube. The maximum collection time was four minutes. No contaminations, e.g. hair and blood, were observed in visual inspection. Immediately after sampling, proteins were precipitated, and metabolites extracted with two volumes of acetonitrile mixed with 1 volume of saliva simultaneously mixing gently in vortex and finally at maximum speed 10 s. Samples were kept on ice during shipping and stored in − 20 °C 3–5 days prior to metabolomics analysis.

Human saliva samples were collected at least one hour after eating and drinking between 9 and 11 a.m. Stimulated saliva was collected using the standard technique according to Navazesh (1993) as follows. The saliva flow was stimulated by chewing a paraffin wax (1 g; Orion Diagnostica, Espoo, Finland) for 30 s, followed by the collection of the produced saliva into a glass cup for five minutes. Saliva samples were transported to the laboratory on ice, and then clarified by centrifugation (3000×*g*, 20 min, + 4 °C). The supernatants were stored at − 20 °C for later use.

### Sample preparation

Dog and human saliva samples were thawed on ice. Human saliva samples were precipitated and extracted similarly as dog saliva (200 µL of saliva and 400 µL of acetonitrile). All samples were centrifuged (10,600×*g*, 5 min, + 4 °C), and the supernatants were filtrated through 0.2 µm Acrodisc® Syringe Filters with a PTFE membrane (PALL Corporation, Ann Arpor, MI) prior subjecting to the LC–MS analyses. Quality control (QC) samples were made separately from dog and human samples by mixing aliquots of 30 µl from every dog or human supernatant to one tube. QC mixed sample contained aliquots from every dog and human sample mixed into one tube.

HPLC-grade acetonitrile (VWR Chemicals, Fontenay-sous-Bois, France) was used for sample preparation. LC–MS grade methanol (Riedel-de Haën™, Honeywell, Seelze, Germany), HPLC-grade acetonitrile (VWR Chemicals, Fontenay-sous-Bois, France), LC–MS grade formic acid (Fluka™, Honeywell, Seelze, Germany), ammonium formate (Fluka™, Honeywell, Seelze, Germany) and class 1 ultra-pure water (ELGA Purelab ultra Analytical, UK) were used for mobile phase eluents in RP and HILIC chromatographic separation.

### UHPLC-qTOF-MS analysis

The samples were analyzed by a 1290 LC system coupled to a 6540 UHD accurate-mass qTOF spectrometer (Agilent Technologies, Waldbronn, Karlsruhe, Germany) using electrospray ionization (ESI, Jet Stream) in positive (+) and negative (−) polarity. Separation was performed using reversed phase (RP) chromatography with a Zorbax Eclipse XDB-C18 column (2.1 × 100 mm, 1.8 µm, Agilent Technologies, Palo Alto, CA, USA). The column temperature was 50 °C and flow rate 0.4 ml/min. Mobile phase consisted either water (A) or methanol (B) both with 0.1% (v/v) formic acid. The gradient was as follows: 2% B followed by a gradient to 100% B in 10 min, an isocratic step at 100% B for 4.5 min and 2% B for 2 min. Hydrophilic interaction (HILIC) chromatographic separation was performed on Acquity UPLC® BEH Amide column (2.1 × 100 mm, 1.7 µm, Waters Corporation, Milford, MA). The column temperature was 45 °C and flow rate 0.6 ml/min. Mobile phase consisted of 50% v/v acetonitrile in water (A) or 90% v/v acetonitrile in water (B) both with 20 mM ammonium formate buffer. The gradient was as follows: 100% B for 2.5 min followed by a gradient to 0% B in 10 min and 100% B for 2.5 min. The sample volume of 2.0 µl was injected for each chromatographic run.

The ESI source operated using the following conditions: capillary voltage 3500 V, nozzle voltage 1000 V, fragmentor voltage 100 V, skimmer 45 V, nebulizer pressure 45 psi, drying gas temperature 325 °C and flow 10 l/min and sheath gas temperature 350 °C and flow 11 l/min. Mass data were acquired with scan time of 600 ms over a 50–1600 m/z range. For automatic MS/MS analyses, four ions with the highest intensities were selected for fragmentation from every precursor scan cycle where precursor isolation width was set to 1.3 Da. Selected precursor ions were excluded after two product ion spectra and released after a 0.25-min hold. Precursor scan time either ended at 20,000 counts or after 500 ms, depending on the ion intensity. Product ion scan time was 500 ms. Collision energies were 10 V, 20 V and 40 V. Continuous internal calibration was performed during analyses to assure the desired mass accuracy. The reference ions from infusion solution were m/z 121.05087300 and 922.00979800 for positive mode and m/z 112.985587 and 966.000725 for negative mode. For the quality assurance of the chromatographic and mass spectrometry runs, QC mixed sample were injected at the beginning of the analysis and after every 9 samples. Separate dog QC and human QC samples were analyzed in the beginning of the corresponding analysis to provide the MS data, and used for the automatic data-dependent MS/MS analyses. The data acquisition was accomplished with MassHunter Acquisition B.05.01 software (Agilent Technologies).

### Non-targeted metabolomics data preprocessing

The LC–MS raw data from four different analytical modes (RP+, RP−, HILIC+, HILIC−) was exported to MassHunter Qualitative Analysis B.07.00 (Agilent Technologies, USA) for feature extraction and peak picking combined with chromatographic alignment across all data files per mode. To remove the redundant and non-specific information considered as background noise, peaks with ion abundance less than 10,000 were excluded from further analysis. The feature files were imported as compound exchange format (.cef-files) into Agilent Mass Profiler Professional software (MPP version 13.1.1, Agilent Technologies) for compound alignment yielding a peak list which was exported to Microsoft Excel 2016. Altogether, 8375 molecular features were collected in the four analytical modes. Out of those, molecular features that were present in at least 50% of the samples in either of the sample groups (5468 features) were considered for metabolite identification. Principal component analysis was performed using SIMCA (version 15, Umetrics).

### Metabolite identification

The putative metabolite identification was performed using an open-source software, MS-DIAL (RIKEN PRIMe). Collected MS/MS data was converted as.abf-files using Analysis Base File Converter program (Reifycs Inc.) and converted files were imported to MS-DIAL (versions 2.66 to 3.12). Public databases, Metlin and MassBank of North America (MoNA), and in-house LC–MS/MSMS standard library were downloaded to MS-DIAL for utilization of retention time, accurate mass, isotope ratio and MS/MS spectrum information for peak and metabolite identification. The built-in MS-DIAL library was utilized for lipid identification. Each matched spectrum was manually inspected. The guidelines from Sumner et al. (2007) were used for ranking metabolite identifications as follows: Compounds in identification level 1 were verified by comparing exact mass, retention time and MS/MS fragmentation spectra with in-house LC–MS/MSMS standard library. Compounds in level 2 were matched with exact mass and MSMS spectra from public databases mentioned above. MassHunter Profinder B.08.00 software (Agilent Technologies) was applied for targeted feature extraction to minimize the appearance of false negative features implemented with the manual inspection and integration of the targeted feature.

## Results

**Fig. 1:**
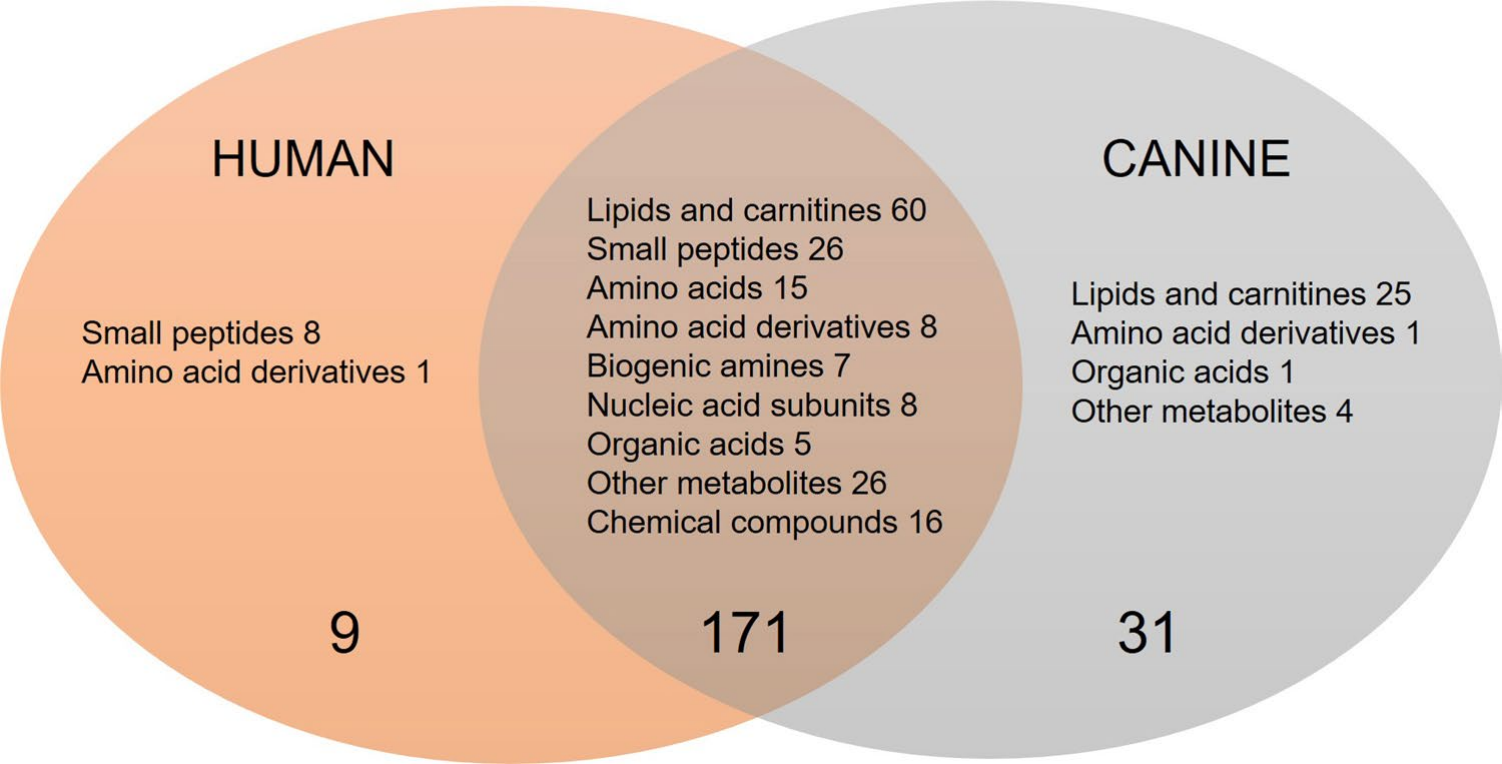
Venn diagram displaying the shared and unique salivary metabolites by classes among the two species. Metabolite was annotated as unique when the ion abundance was confirmed as zero in all samples per study group. Unique metabolites with zero ion abundance were confirmed with the manual inspection and integration of the targeted feature using MassHunter Profinder B.08.00 software (Agilent Technologies)

With the aim to explore salivary metabolite composition in dog and human, we focused on 5468 metabolic features collected with four analytical modes using a non-targeted metabolomics approach. A total of 211 metabolites were identified (Table 1) including both endogenous and exogenous compounds. Among those, 31 metabolites (14.6%) were found only in dog saliva, and 9 metabolites (4.2%) only in human saliva (Fig. 1). The identities of 69 metabolites were verified as level 1 identification (Sumner et al. 2007) whereas 142 metabolites were in identification level 2. Characteristics and reference spectra for all identified metabolites in human and dog saliva are given as supplementary material (S1). The identified metabolites were classified as amino acids, amino acids derivatives, biogenic amines, lipids and carnitines, nucleic acid subunits, organic acids, small peptides, chemicals, and other metabolites.

**Table 1:**
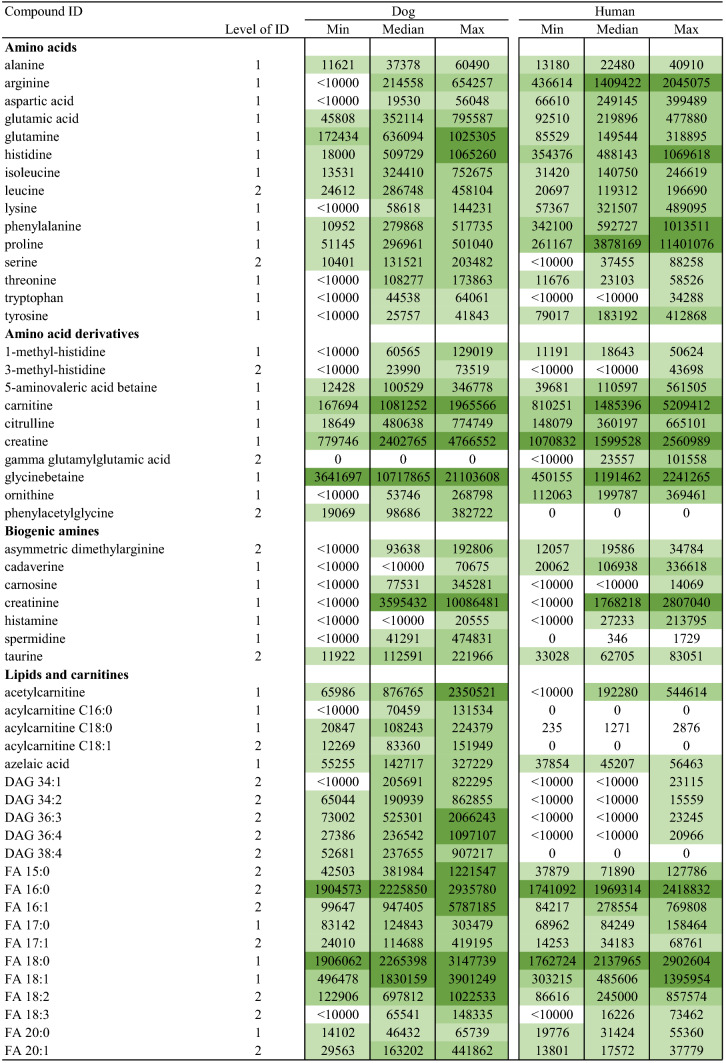

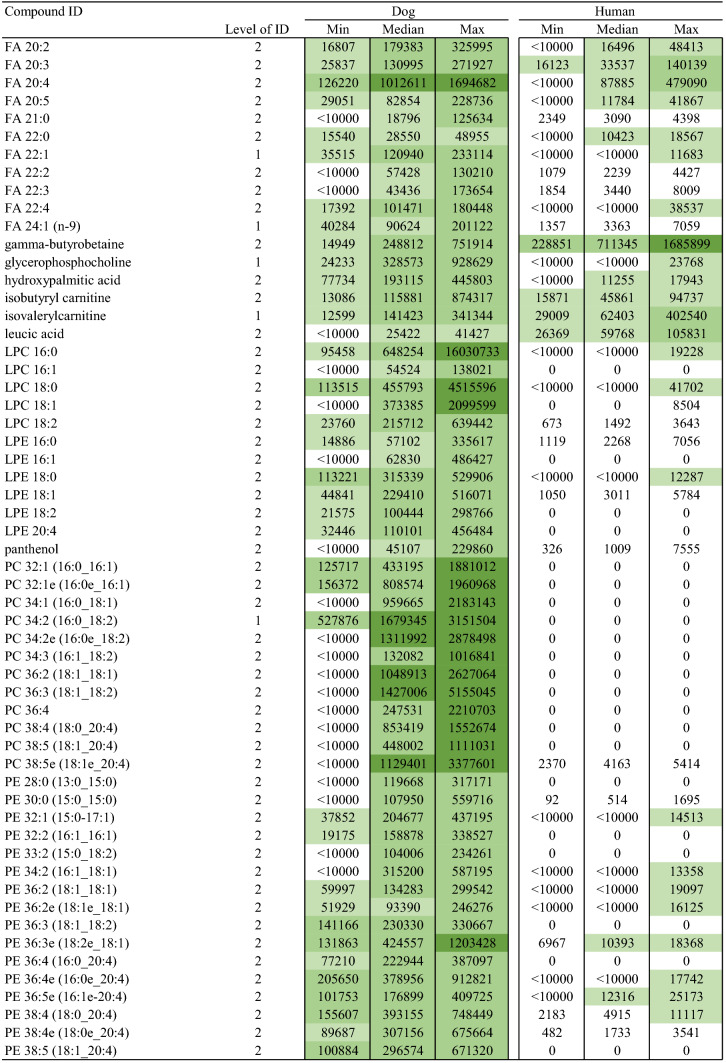

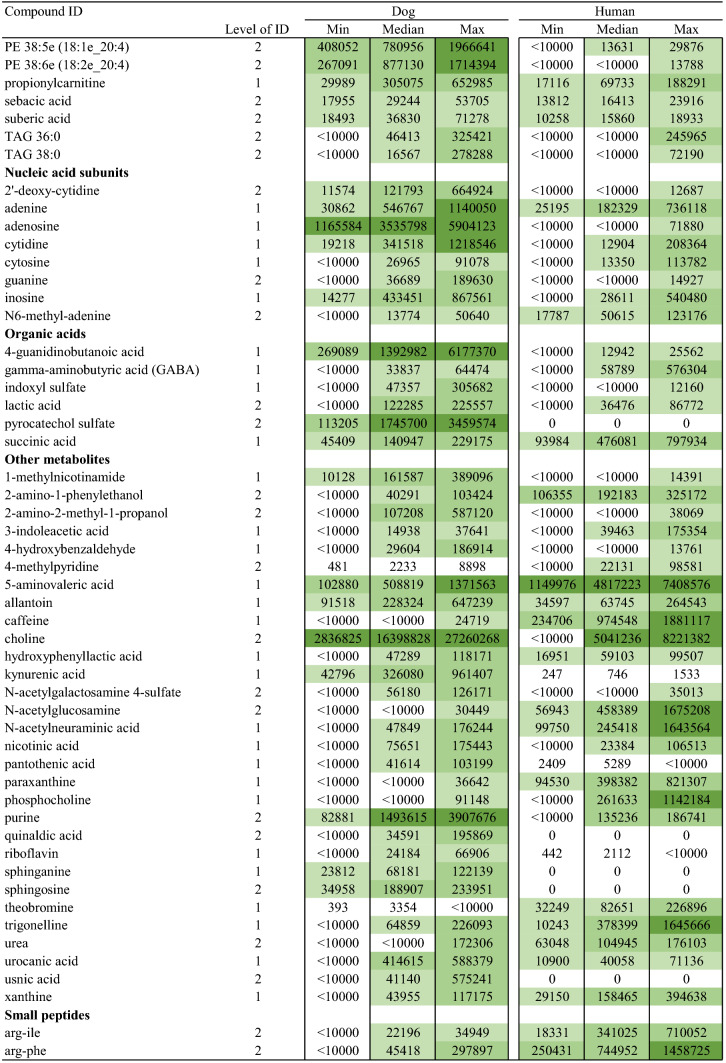

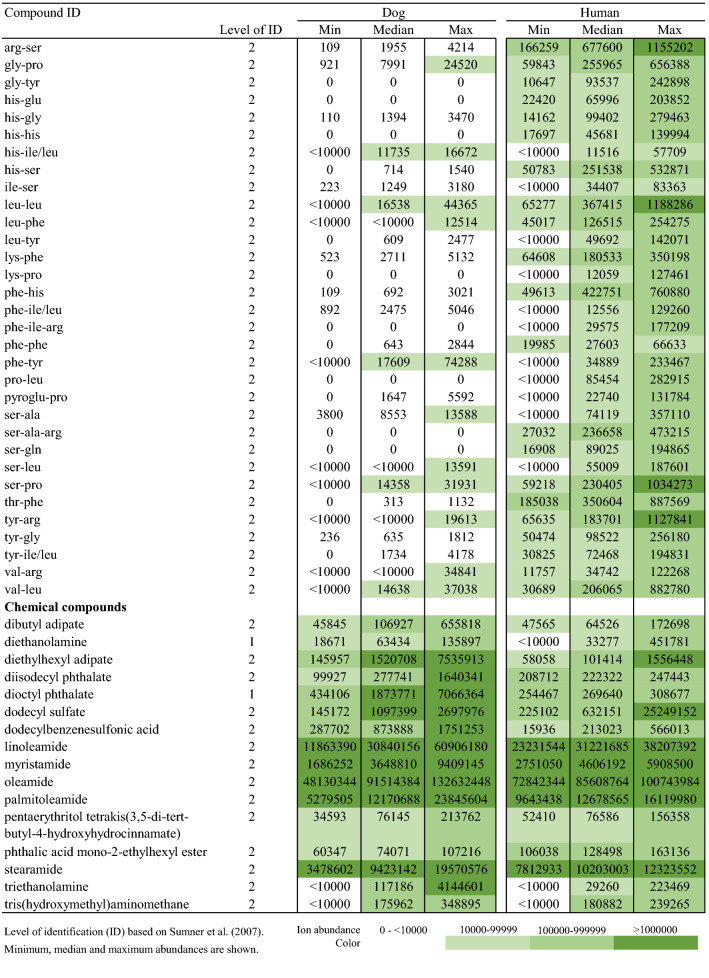
Identified metabolites in dog and human saliva in the non-targeted LC–MS analysis

The major difference between the human and dog saliva metabolites was observed in the lipid group. Dog saliva contained 25 lipids or lipid-like molecules (i.e. carnitines), which were absent in the human saliva, including 11 phosphatidylcholines (PC), 6 phosphatidylethanolamines (PE), 3 lysophosphatidylethanolamines (LPE), 2 lysophosphatidylcholines (LPC), 1 diacylglycerol (DAG) and 2 acylcarnitines. In contrast, small peptides, including mostly dipeptides, were more prevalent in human when compared to canine saliva. Dogs were completely lacking eight of the 34 identified small peptides, and in total, 13 dipeptides had minor ion abundance in the dog saliva.

Both the dog and human saliva contained 15 of the 20 generic amino acids. However, asparagine, cysteine, glycine, methionine and valine were not detected from the saliva of both species. The group of amino acid derivatives included ten metabolites. Among those, gamma glutamylglutamic acid was detected only in humans, and phenylacetylglycine only in canines. Besides amino acids and their derivatives, asymmetric dimethylarginine (ADMA), cadaverine, carnosine, creatinine, histamine, spermidine and taurine were identified as biogenic amines. Those seven metabolites and eight different nucleic acid subunits were detected in both species. Furthermore, canine saliva contained also one unique organic acid which was identified as pyrocatechol sulfate, and four other compounds named quinaldic acid, sphinganine, sphingosine and usnic acid.

The entity of identified metabolites in canine and human saliva indicate partially comprised species-specific metabolic profiles (Table 1). In addition, a large variation in ion abundance within and between the identified saliva metabolites were observed in both species. Inter-individual variation and sample variation is shown with descriptive statistics in the supplementary material (S2). Furthermore, the principal component analysis clearly separated the different species, however large variation was also observed within species (supplementary material S3). Example total ion chromatograms of salivary samples of dog and humans from all four analytical modes are shown in the supplementary material (S4).

## Discussion

This study provides the first characterization of canine saliva metabolome and compares its content to the human saliva. We identified altogether 211 metabolites in 13 dog and 14 human saliva samples. Dog saliva contained 31 unique metabolites that were mostly lipids and lipid-like molecules. This study demonstrates the feasibility of UHPLC-qTOF-MS in canine and human salivary metabolomics. Exploitation of both, high-resolution precursor and fragmentation data in MS/MS, enables the identification of metabolites that typically exist in lower amounts in saliva than in serum.

Comparison of the content of canine and human saliva revealed differences in the salivary lipids and lipid-like molecules. PCs were not detected in our current analysis in the human saliva, and in addition, eight out of 18 PEs were absent or found with low ion abundance in the human saliva. However, previous studies have reported on PCs in human saliva (Dame et al. 2015). Likewise, our analytical method is capable of detecting PCs, as we have reported them earlier from e.g. human plasma, and therefore most likely other than methodological issues are the reason why they were not detected in the current analysis from human saliva. PCs and PEs are major phospholipids of the plasma membranes in animal cells. This result may indicate the presence of epithelial cell membranes from the oral cavity in the dog saliva samples. Other identified lipids also had lower ion abundance in the human saliva samples when compared with the canine samples. An exception was observed with the most abundant fatty acids in tissues, FA 16:0, FA 18:0 and FA 18:1, which were present with high ion signals also in the human samples. These findings agree with a quantitative study conducted by Larsson et al. (1996) where lipids were detected only in low concentrations in human saliva. Instead, the whole saliva was found to contain more free fatty acids and neutral lipids like di- and triglycerides than polar lipids, such as PCs or PEs (Larsson et al. 1996).

In contrast to lipids, small peptides were found predominantly in human saliva. Canine saliva included only 13 clear signals from di- and tripeptides, whereas 34 were found from human samples. Small peptides in saliva originate from protein degradation induced by host and bacterial proteases (Liebsch et al. 2019). Thus, oral health status, and especially periodontitis, can affect the salivary dipeptides. In the present study, human participants were healthy, and according to dog owners’, the dogs were not reported to have any diseases with one exception (cataract). It is unclear if the different fasting time (12 h for dogs versus minimum of one hour for human participants) and/or oral health, combined with the differences in antimicrobial and homeostatic protein compositions between species, affect to peptide levels observed in this study. In addition, the differences in sample collection (e.g. using paraffin wax) and handling may have had an effect.

We identified six additional metabolites, which were present only in the canine saliva. Those included sphingosine and its derivative sphinganine, which are the major bases of the sphingolipids in mammals. Metabolites included also phenylacetylglycine (amino acid derivative) and pyrocatechol sulfate (organic acid) which are reported to be normal human metabolites. Previously, pyrocatechol sulfate was detected in our platform not only in human plasma but also in dog plasma (Hanhineva et al. 2015; Puurunen et al. 2016). Moreover, we putatively identified usnic acid in dog saliva. Usnic acid originates from lichens and might be a trace from a dog food. On the contrary to endogenous metabolites, exogenous compounds in saliva are traces from, for example, food, cosmetics, drug intake and environment (Dame et al. 2015). We identified 16 chemical compounds in both species, including phthalates, which are used as plasticizers. Furthermore, we identified two surfactants, dodecyl sulfate and dodecylbenzenesulfonic acid, which are used in cosmetics and foods. In addition, fatty acid amides myristamide, palmitoleamide, stearamide, oleamide and linoleamide, were found with high ion abundance in both species. Although these compounds are recognized as endogenous plasma metabolites (Kim et al. 2019), the identified fatty acid amides are also known as lubricants, detergents and softeners which we have found to be derived from the filters used in sample preparation (data not shown). Thus, they are considered as contaminants in this study.

The identified metabolites in dog and human saliva were characterized by inter-individual variation. Several factors, such as diurnal variation, oral health status, physiological condition, gender, age and nutrition are known to have an influence on the metabolite composition of human saliva (Kawanishi et al. 2019; Liebsch et al. 2019; Mikkonen et al. 2013). These factors should be investigated in dogs when the potential of saliva as a sample material for research and diagnostics is discussed as the differences in saliva metabolites between dog breeds, age and sex remain unsolved in this study. Those above-mentioned factors have been identified as affecting the dog plasma metabolome (Lloyd et al. 2016, 2017) and saliva proteome (Pasha et al. 2018). Nevertheless, saliva provides information from several organs and its utility as “the new blood” for the diagnosis and monitoring of human systemic diseases has been studied through omics (Cuevas-Cordoba and Santiago-Garcia 2014). This could also be a case for dogs and veterinary medicine after overcoming the sampling problems and conducting metabolic profiling with larger sample size.

There are some limitations in our study. Firstly, there is no standard operation procedure for collecting dog saliva. In this study, we designed the canine saliva sampling protocol according to the reviewed literature (Elmongy and Abdel-Rehim 2016; Lensen et al. 2015) which aimed to be the most appropriate for the LC–MS analysis. We observed that, even though the dogs were trained for showing their teeth and to cooperate with their owners, the collection of saliva was still tricky to execute due to the characteristic features of saliva being elastic and mucous. Therefore, alternative sampling techniques that are comfortable for the dogs and easy to perform, and which provide enough sample material, need to be developed. Secondly, the non-targeted LC–MS method yields semi-quantitative data suitable for identification and sample-wise comparison but does not provide exact quantities for the detected compounds in saliva. Therefore, when exact quantities are required, other analytical approaches, such as nuclear magnetic resonance spectroscopy (NMR) or targeted LC–MS methods should be applied. However, the low sensitivity of NMR compared to MS limits the detection of the salivary metabolites identified in this study with NMR technique. Furthermore, metabolite identification was focused to the compounds that were classified into identification level 1 and level 2 according Sumner et al. (2007). A wide range of unidentified metabolites still exist in the canine saliva. This is evident according to our data, where in total over 8000 molecular features were detected providing a couple of hundred identifications. The identification of the metabolites behind these molecular features remains a challenge, and only small fraction of measured molecular features can usually be identified in a non-targeted metabolomics study. In addition, the applied LC–MS method does not capture the whole canine saliva metabolome. Characterization of the whole canine saliva metabolome would require use of more diverse set of methods (e.g. NMR and GC–MS) (Dame et al. 2015). Thirdly, our human subjects were all women. For comparing the metabolomes between species, both genders should be included to the study populations despite studies reporting only quantitative differences in salivary metabolites between men and women (Okuma et al. 2017; Takeda et al. 2009). Moreover, more restricted age range could have reduced variation seen in the canine samples. Finally, comparability between human and canine salivary metabolite profiles would improve if more aspects of the study design including sampling protocol could be harmonized between the species.

In conclusion, we were able to identify 211 metabolites in the dog and human saliva using non-targeted metabolite profiling. This study provides novel information that encourages the continuation of the studies with larger cohorts. The results demonstrate the potential of dog saliva metabolome to be used in understanding, for example, disease pathology or changes in metabolism due to xenobiotics or nutrition. Furthermore, saliva could be a source of specific biomarkers also for canines’ oral health problems as well as other diseases, but further research is needed to establish and validate the canine saliva biomarkers. Understanding the differences between dogs and humans will then allow the results to be extrapolated to human health.

## Electronic supplementary material

Below is the link to the electronic supplementary material.

Supplementary file1 (PDF 3325 kb)

Supplementary file2 (PDF 176 kb)

Supplementary file3 (PDF 152 kb)

Supplementary file4 (PDF 374 kb)

## Data Availability

The datasets generated during and/or analysed during the current study are available from the corresponding author on reasonable request.

## Abbreviations

ala: Alanine
arg: Arginine
DAG: Diacylglycerol
FA: Fatty acid
gln: Glutamine
glu: Glutamic acid
gly: Glycine
his: Histidine
ile: Isoleucine
leu: Leucine
LPC: Lysophosphatidylcholine
LPE: Lysophosphatidylethanolamine
PC: Phosphatidylcholine
PE: Phosphatidylethanolamine
phe: Phenylalanine
pro: Proline
pyroglu: Pyroglutamic acid
ser: Serine
TAG: Triacylglycerol
thr: Threonine
tyr: Tyrosine
val: Valine
