# Supplementary material for: Metabolome of canine and human saliva: a non-targeted metabolomics study
Source: Metabolomics. 2020 Aug 25;16(9):90. doi: 10.1007/s11306-020-01711-0 (PMC7447669; doi:10.1007/s11306-020-01711-0)

METABOLOME OF CANINE AND HUMAN SALIVA: A NON-TARGETED METABOLOMICS STUDY

Soile Turunen[1*], Jenni Puurunen[2,3], Seppo Auriola[1], Arja M Kullaa[4], Olli Kärkkäinen[1], Hannes Lohi[2,3], Kati Hanhineva[5]

[1]School of Pharmacy, Faculty of Health Sciences, University of Eastern Finland, Kuopio, Finland

[2]Department of Veterinary Biosciences, and Department of Medical and Clinical Genetics, University of Helsinki, Helsinki, Finland

[3]Folkhälsan Research Center, Helsinki, Finland

[4]Institute of Dentistry, School of Medicine, Faculty of Health Sciences, University of Eastern Finland, Kuopio, Finland

[5]Institute of Public Health and Clinical Nutrition, Faculty of Health Sciences, University of Eastern Finland, Kuopio, Finland

*corresponding author Soile Turunen, soiru@uef.fi, +358503455549

S1 Characteristics and reference spectra for the identified metabolites in canine and human saliva.

Compound ID (level of identification), column and ionization mode, mass per charge ratio (*m/z*) and adduct, molecular weight (MW), retention time (RT), MSMS energy (eV), database identification

Amino acids

alanine (1), hilic pos, m/z 90.055 (M+H)+, MW 89.0474, RT 5.54 min, 10V, HMDB00161

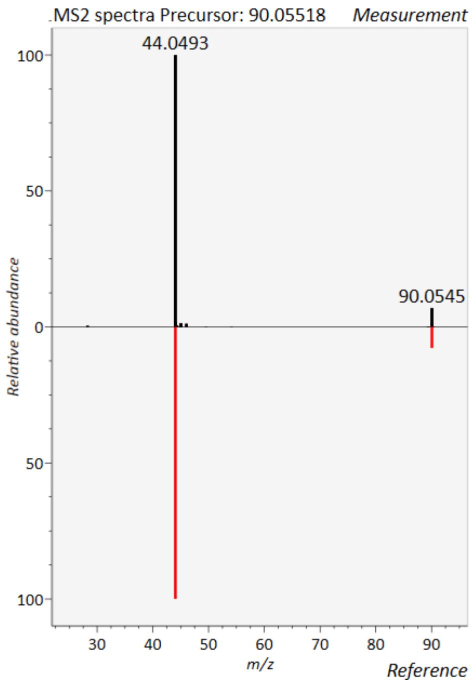

arginine (1), hilic pos, m/z 175.1191 (M+H)+, MW 174.1119, RT 6.84 min, 20V, HMDB00517

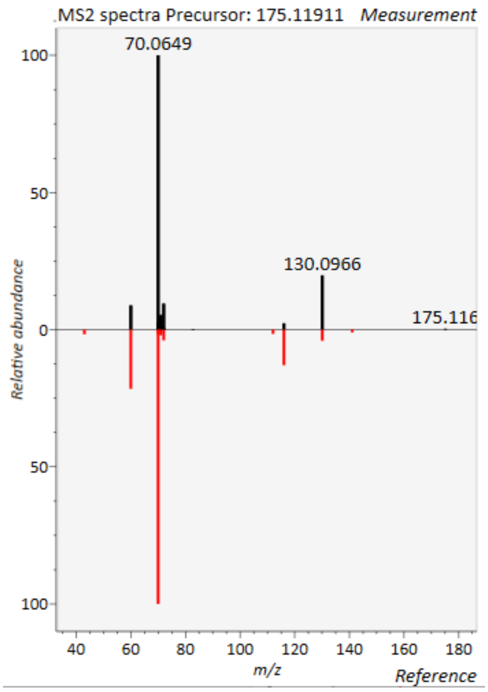

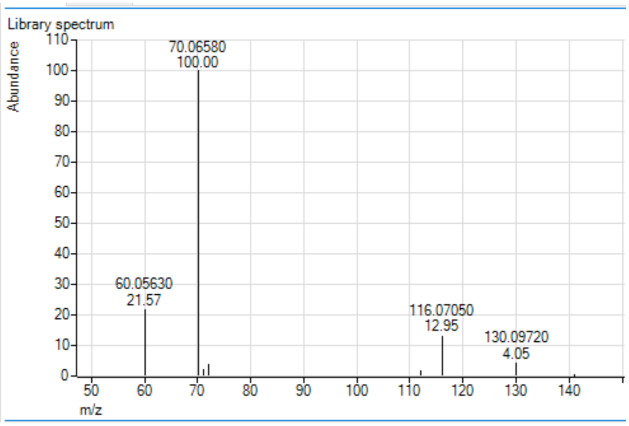

aspartic acid (1), hilic pos, m/z 134.0449 (M+H)+, MW 133.0372, RT 6.65 min, 10V, HMDB06483

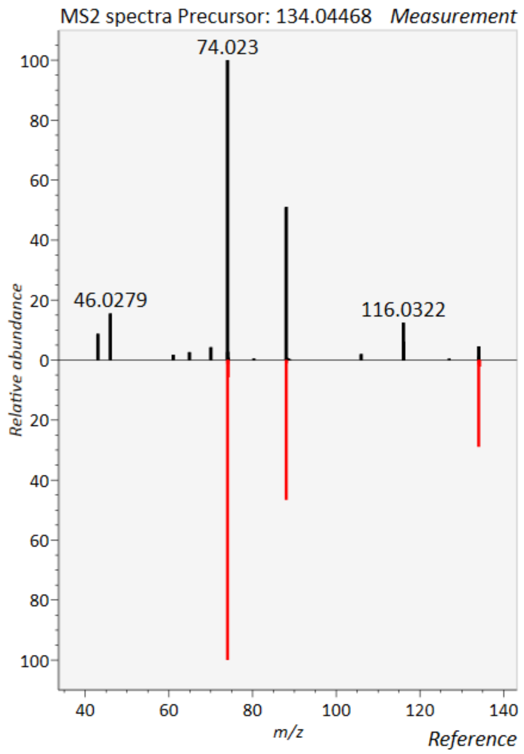

glutamic acid (1), hilic neg, m/z 146.0456 (M-H)-, MW 147.0529, RT 6.09 min, 10V, HMDB00148

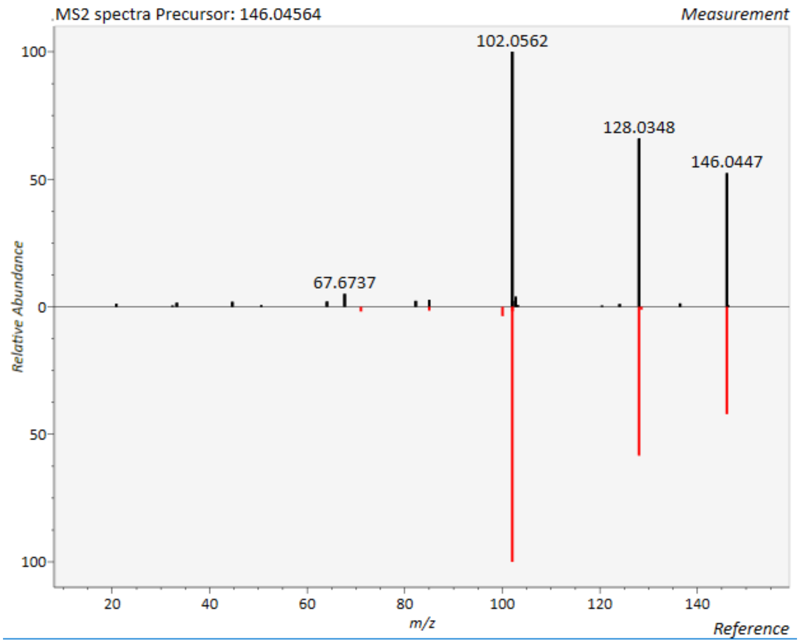

glutamine (1), hilic pos, m/z 147.0761 (M+H)+, MW 146.0689, RT 6.02 min, 10V, HMDB00641

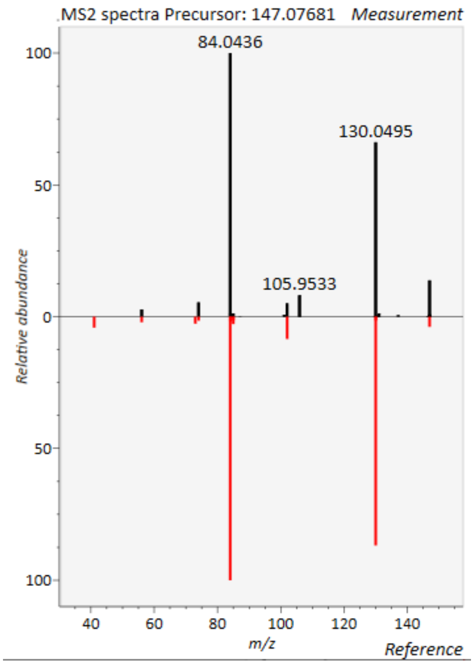

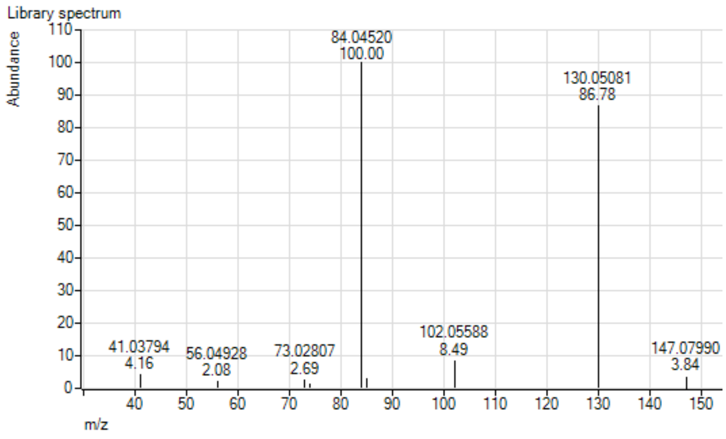

histidine (1), hilic pos, m/z 156.0769 (M+H)+, MW 155.069, RT 6.66 min, 20V, HMDB00177

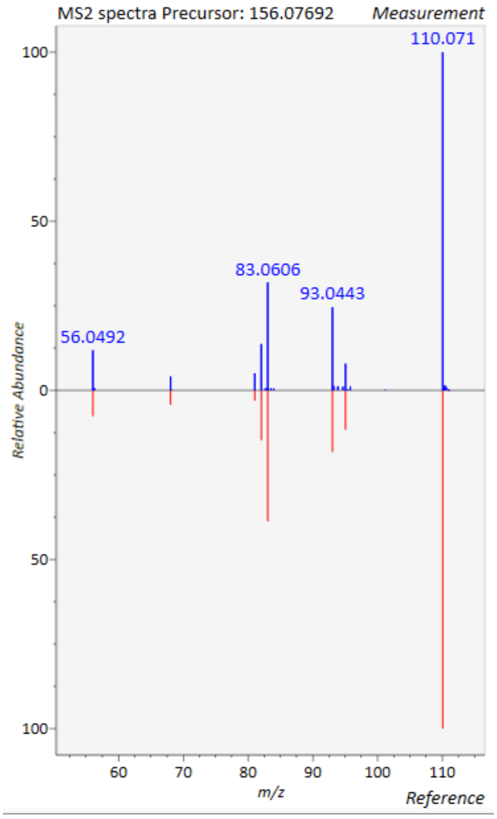

isoleucine (1), RP pos, m/z 132.1020 (M+H)+, MW 131.0947, RT 1.29 min, 20V, HMDB00172

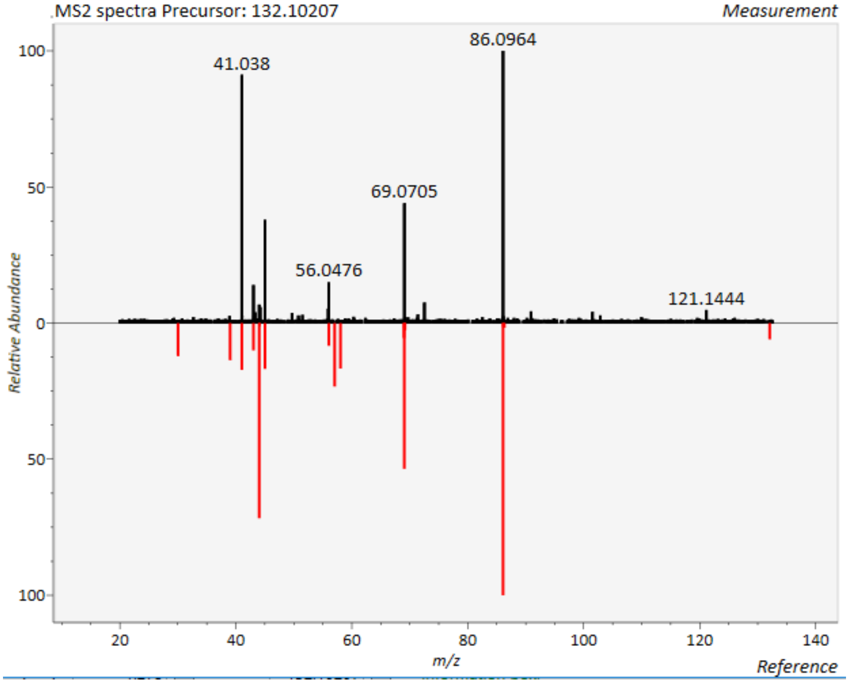

leucine (2), hilic pos, m/z 132.1019 (M+H)+, MW 131.0939, RT 3.94 min A: 20V, B: 40V, HMDB00687

A

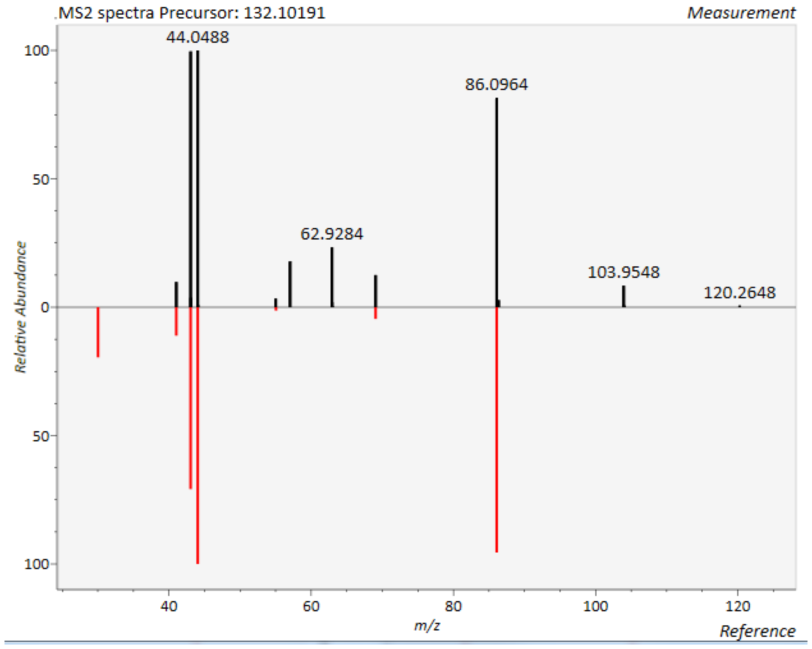

B

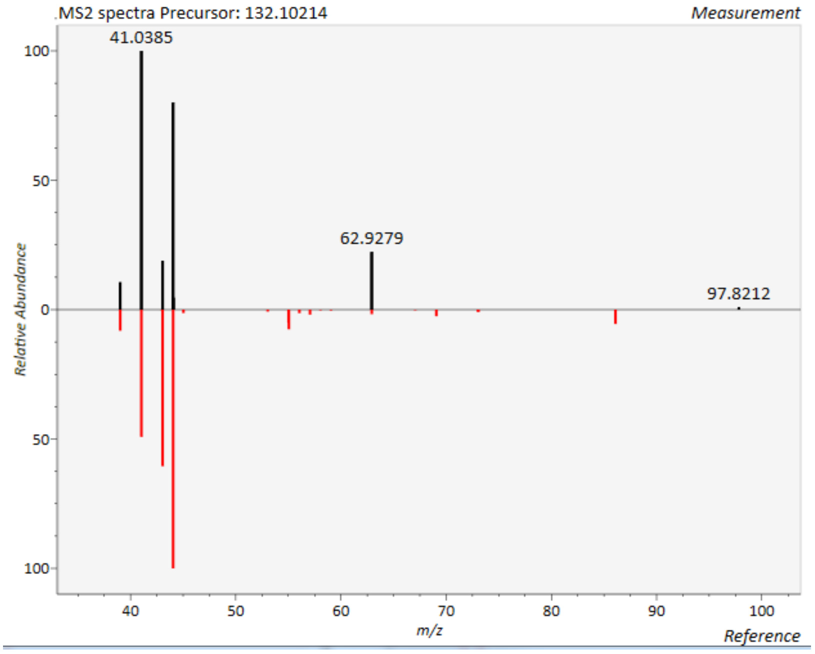

lysine (1), hilic pos, m/z 147.1122 (M+H)+, MW 146.1059, RT 6.99 min, 40V, HMDB00182

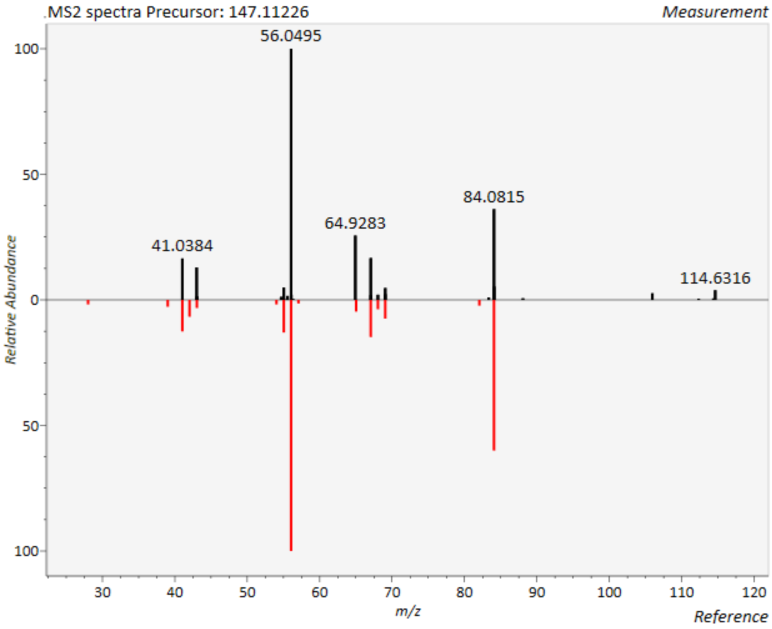

phenylalanine (1), RP pos, m/z 166.0867 (M+H)+, MW 165.0797, RT 1.87 min, 40V, HMDB00159

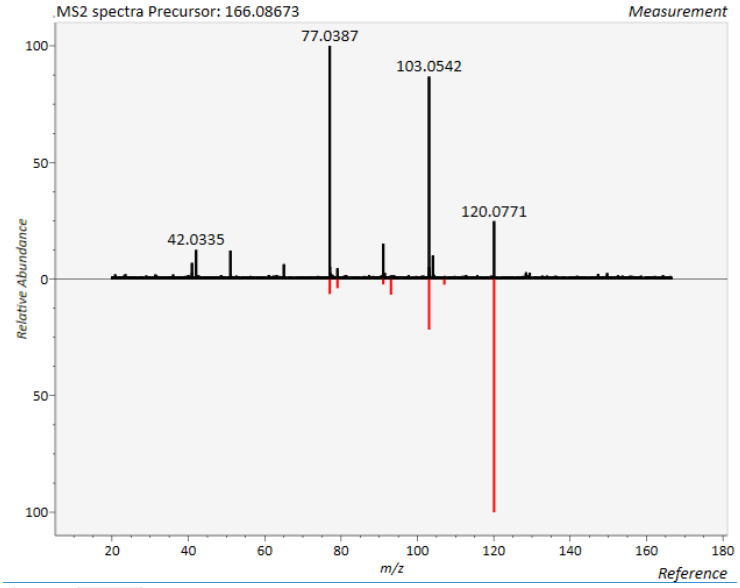

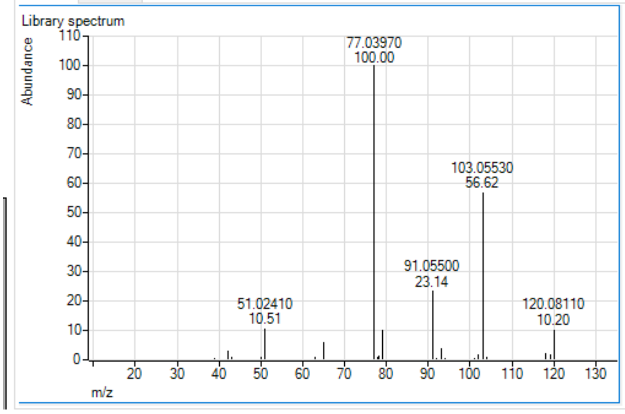

proline (1), hilic pos, m/z 116.0707 (M+H)+, MW 115.0637, RT 4.80 min, 20V, HMDB00162

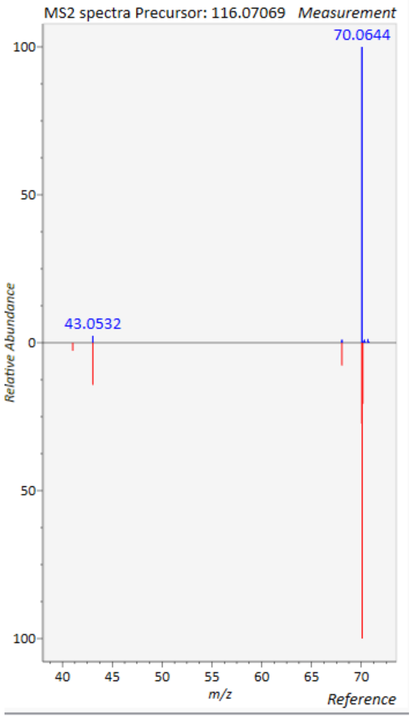

serine (2), hilic pos, m/z 106.0499 (M+H)+, MW 105.0425, RT 6.06 min, 20V, HMDB00187

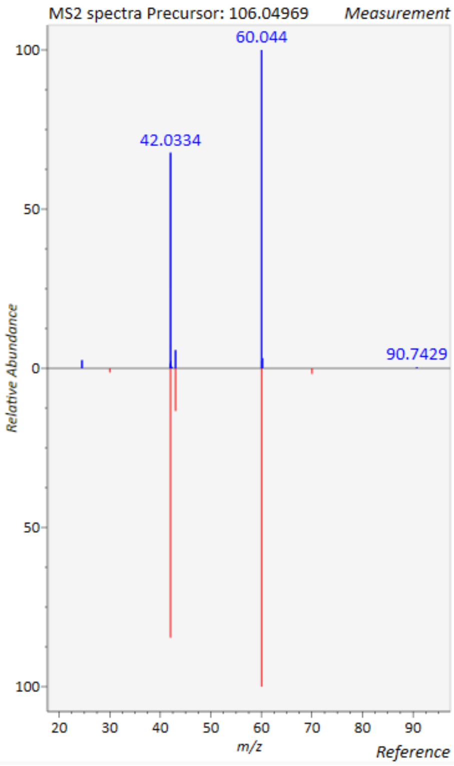

threonine (1), hilic pos, m/z 120.0629 (M+H)+, MW 119.0583, RT 5.69 min, 10V, HMDB00167

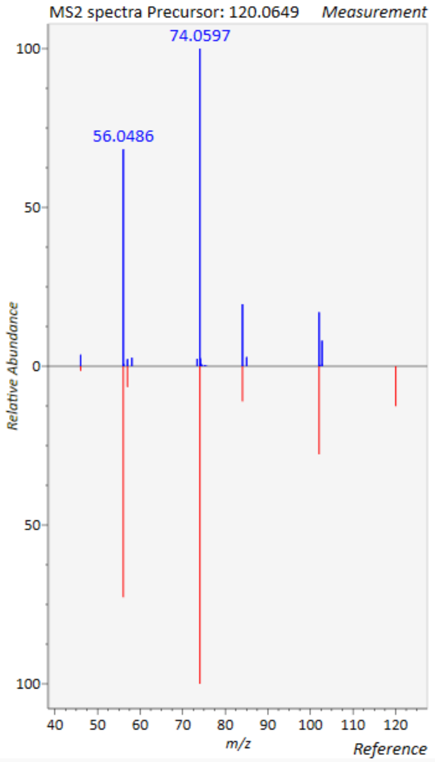

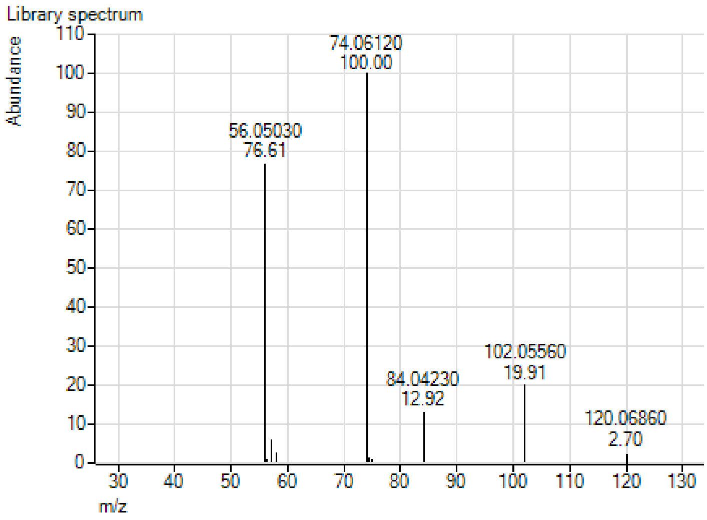

tryptophan (1), hilic pos, m/z 205.0977 (M+H)+, MW 204.09, RT 3.97 min, 20V, HMDB00929

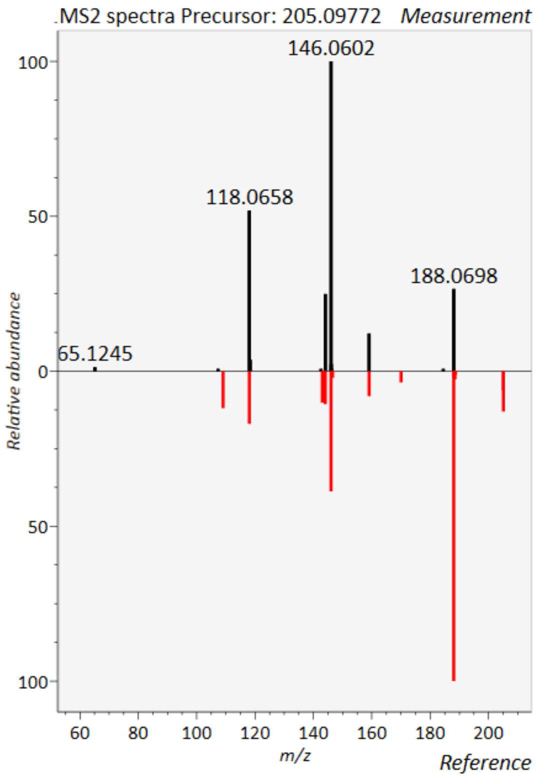

tyrosine (1), hilic pos, m/z 182.0811 (M+H)+, MW 181.0747, RT 5.02 min, 20V, HMDB00158

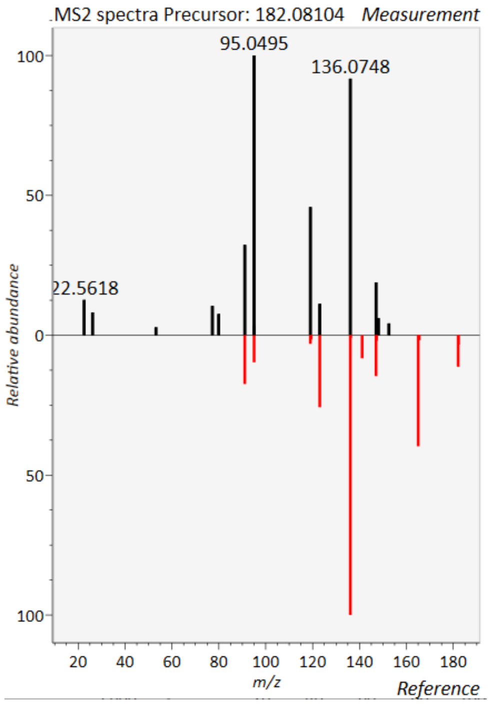

Amino acid derivatives

1-methyl-histidine (1), hilic pos, m/z 170.0928 (M+H)+, MW 169.0855, RT 6.18 min, 20V, HMDB00001

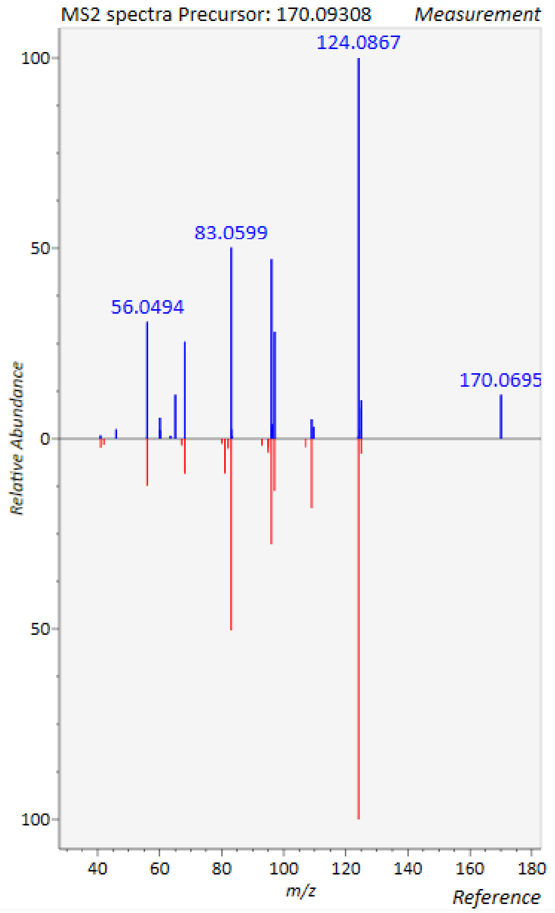

3-methyl-histidine (2), hilic pos, m/z 170.0928 (M+H)+, MW 169.0856, RT 6.75 min, 20V, HMDB00479

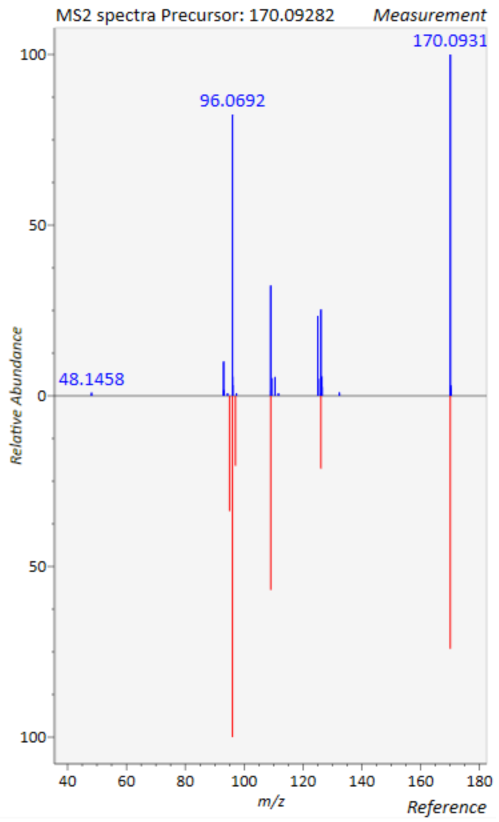

5-aminovaleric acid betaine (1), hilic pos, m/z 160.1333 (M+H)+, MW 159.1258, RT 2.11 min, 20V, PubChem CID 14274897

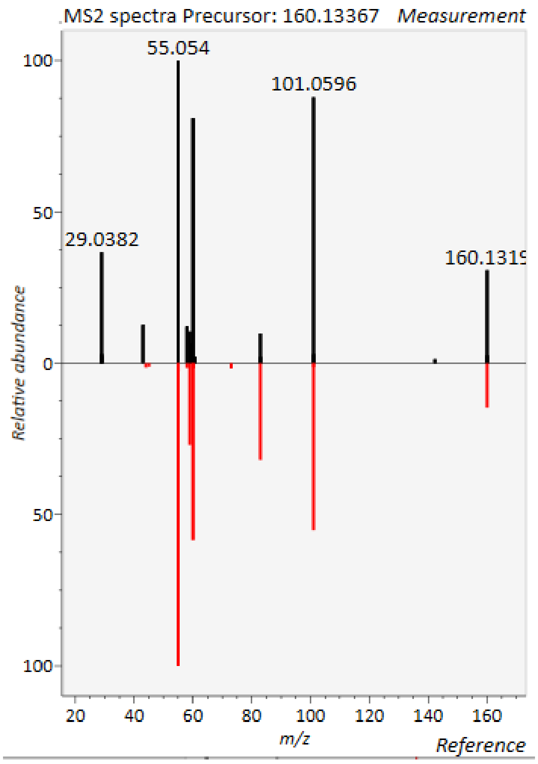

carnitine (1), hilic pos, m/z 162.1127 (M+H)+, MW 161.1057, RT 4.78 min, 20V, HMDB00062

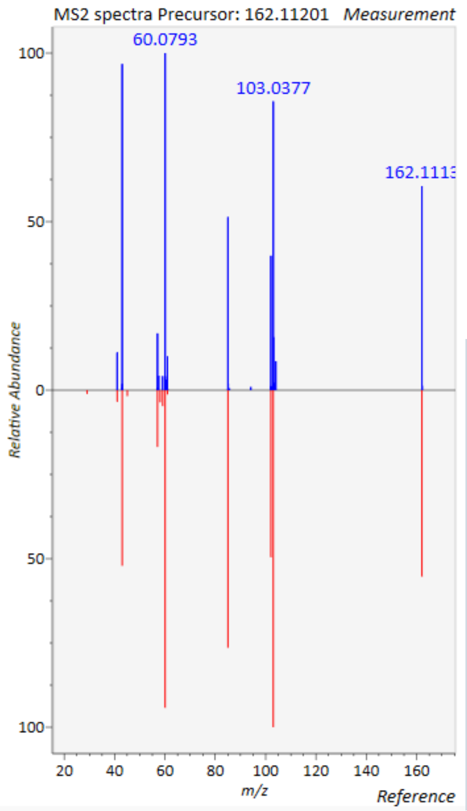

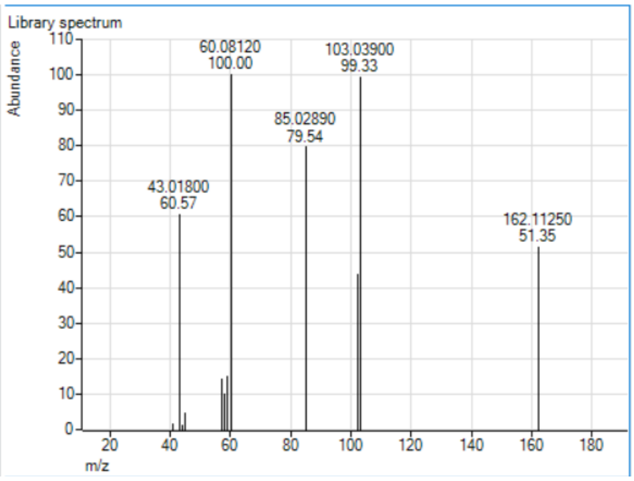

citrulline (1), hilic pos, m/z 176.1034 (M+H)+, MW 175.096, RT 6.25 min, 10V, HMDB00904

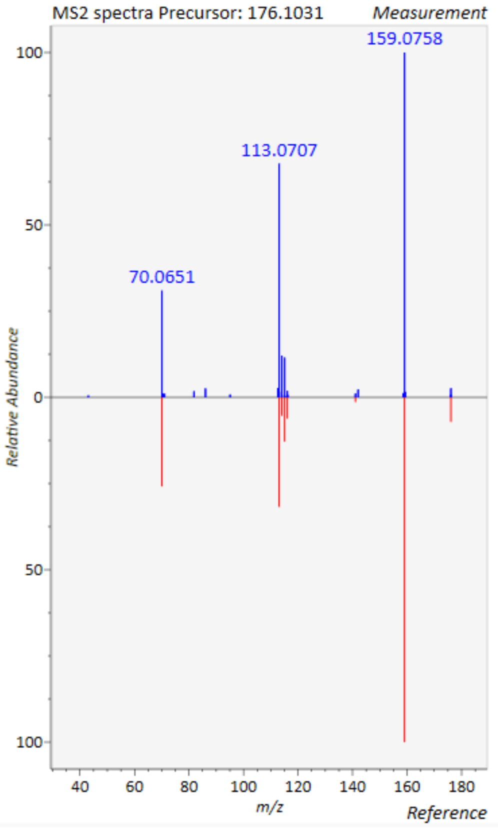

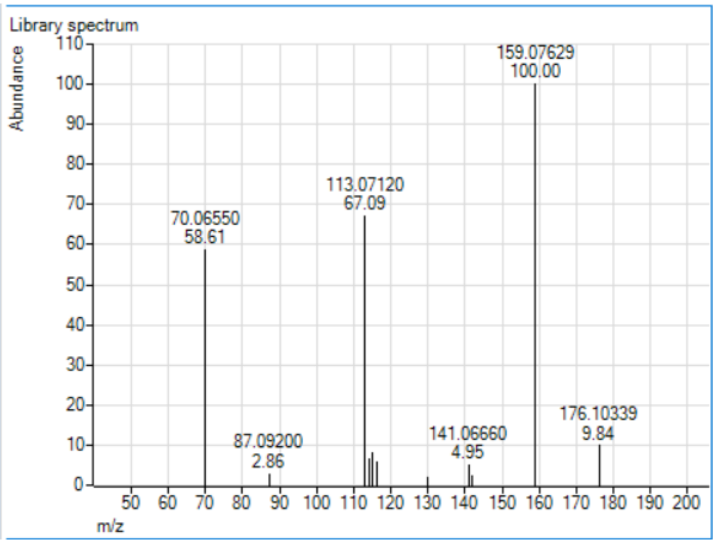

creatine (1), hilic pos, m/z 132.0768 (M+H)+, MW 131.07, RT 5.48 min, 20V, HMDB00064

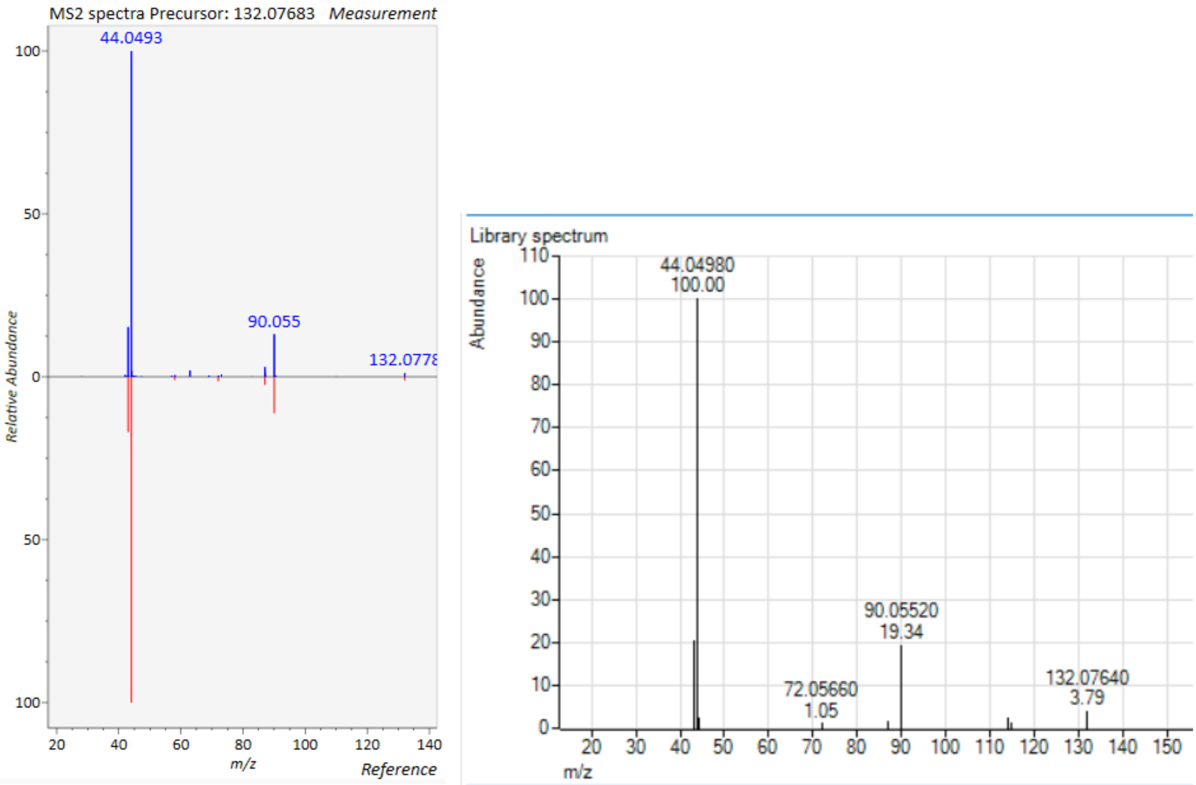

gamma glutamylglutamic acid (2), hilic pos, m/z 277.1035 (M+H)+, MW 276.0963, RT 6.76 min, 20V, HMDB11737

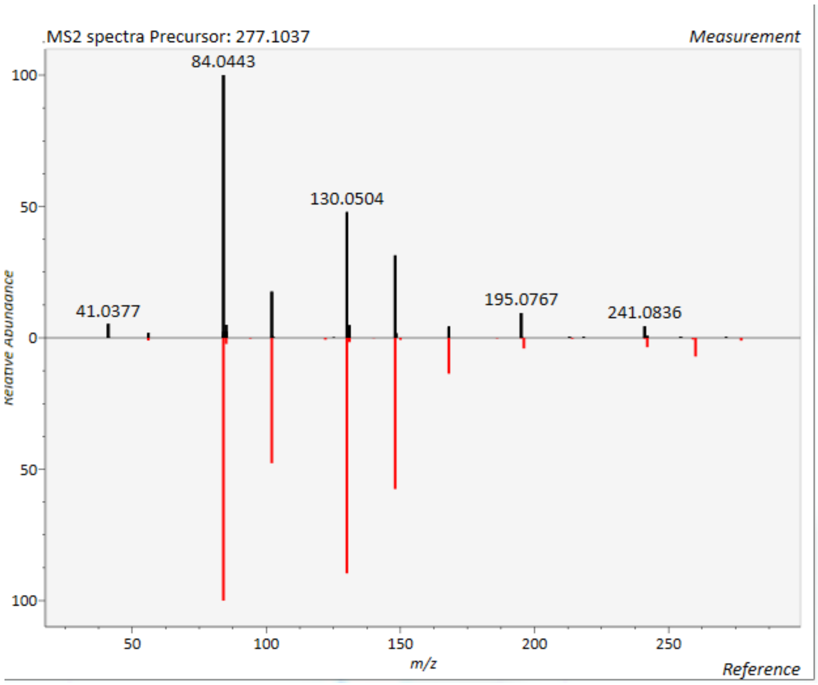

glycinebetaine (1), hilic pos, m/z 118.0862 (M+H)+, MW 117.079, RT 3.77 min, 20V, HMDB00043

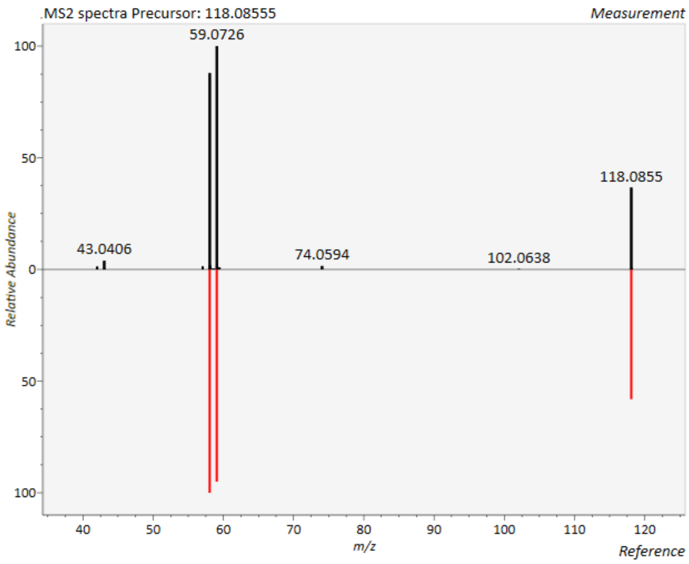

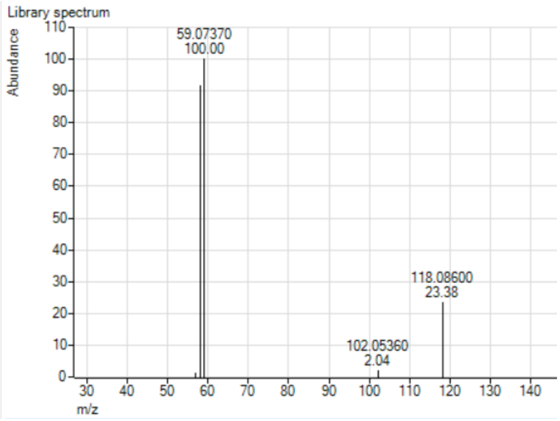

ornithine (1), hilic pos, m/z 133.0972 (M+H)+, MW 132.0894, RT 7.04 min, 10V, HMDB00214

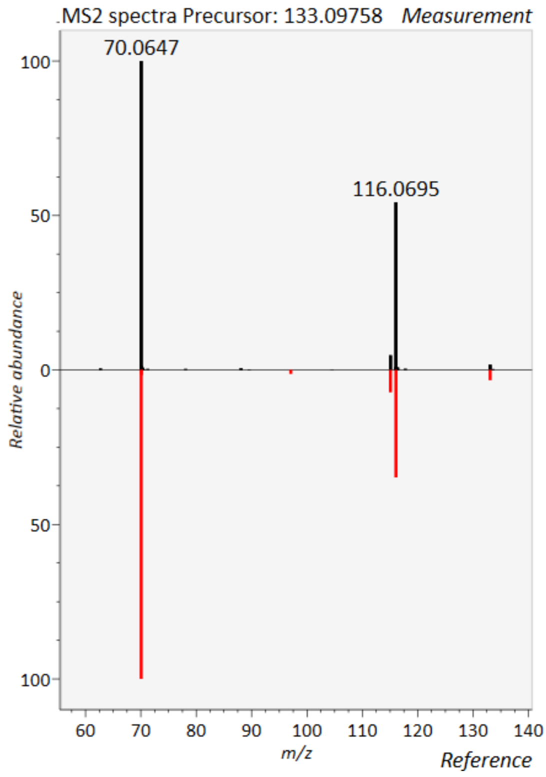

phenylacetylglycine (2), hilic neg, m/z 192.0666 (M-H)-, MW 193.0735, RT 0.82 min, 10V, HMDB00821

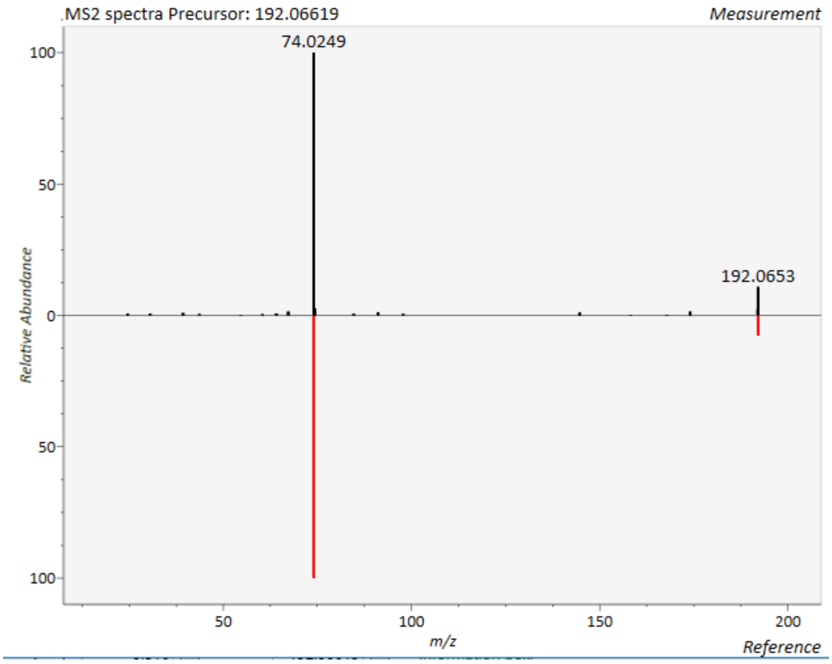

Biogenic amines

asymmetric dimethylarginine (ADMA) (2), hilic pos, m/z 203.1506 (M+H)+, MW 202.143, RT 6.34 min, 20V, HMDB01539

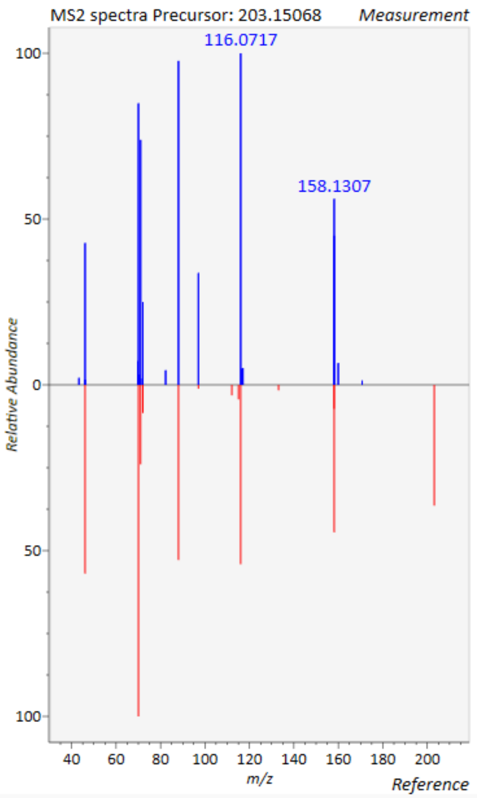

cadaverine (1), hilic pos, m/z 103.123 (M+H)+, MW 102.1154, RT 6.21 min, 20V, HMDB02322

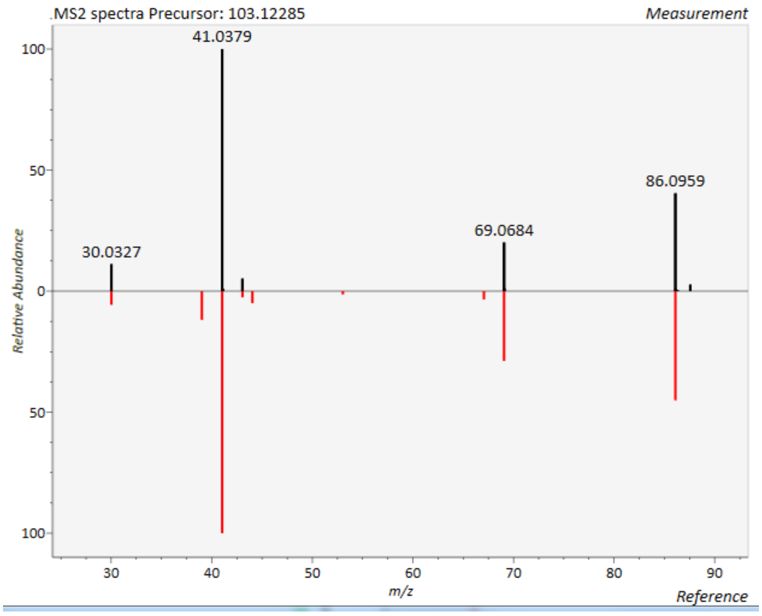

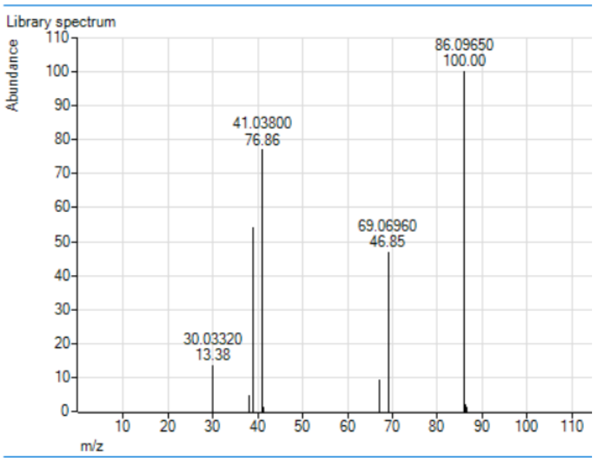

carnosine (1), hilic neg, m/z 225.0991 (M-H)-, MW 226.1062, RT 6.90 min, 20V, HMDB00033

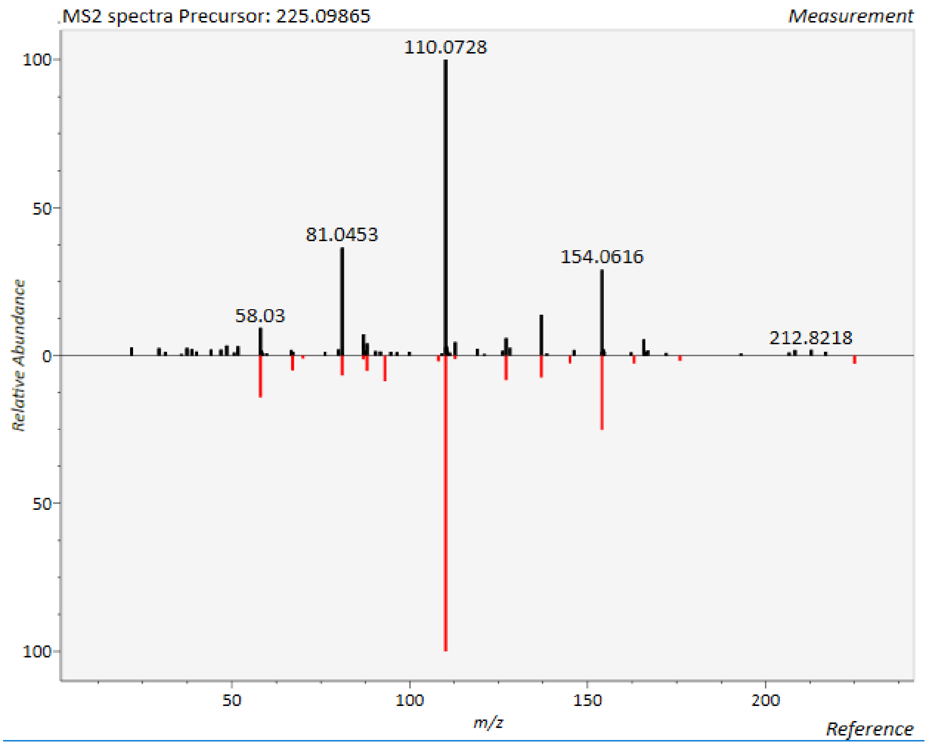

creatinine (1), hilic pos, m/z 114.0665 (M+H)+, MW 113.0593, RT 1.28 min, 20V, HMDB00562

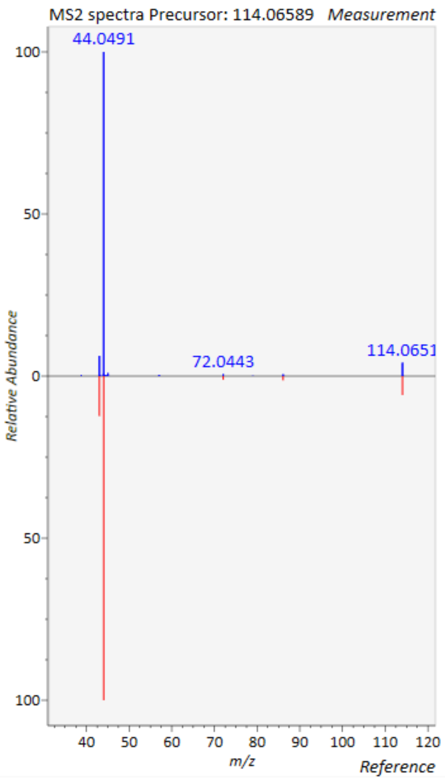

histamine (1), hilic pos, m/z 112.087 (M+H)+, MW 111.0793, RT 5.48 min, 10V, HMDB00870

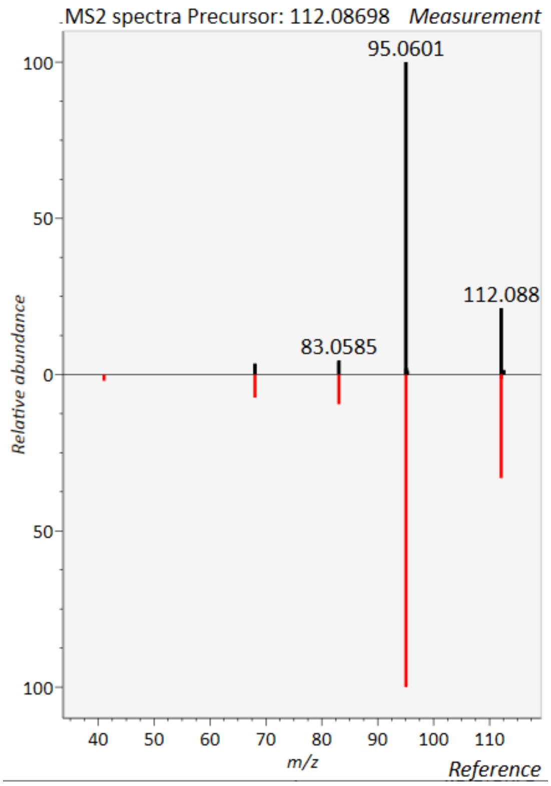

spermidine (1), hilic pos, m/z 146.1655 (M+H)+, MW 145.1576, RT 7.60 min, 20V, HMDB01257

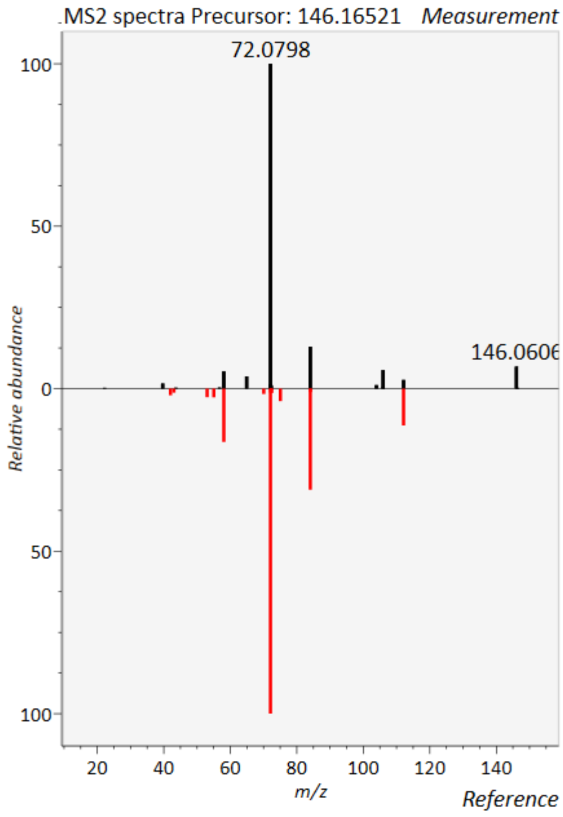

taurine (2), hilic pos, m/z 126.0217 (M+H)+, MW 125.0146, RT 5.28 min, 20V, HMDB00251

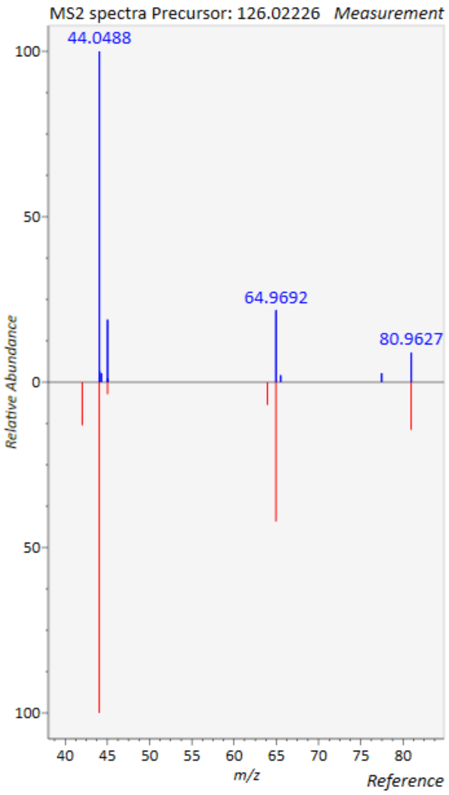

Chemical compounds

dibutyl adipate (2), RP pos, m/z 259.191 (M+H)+, MW 258.1833, RT 9.25 min, 20V, PubChem CID 7784

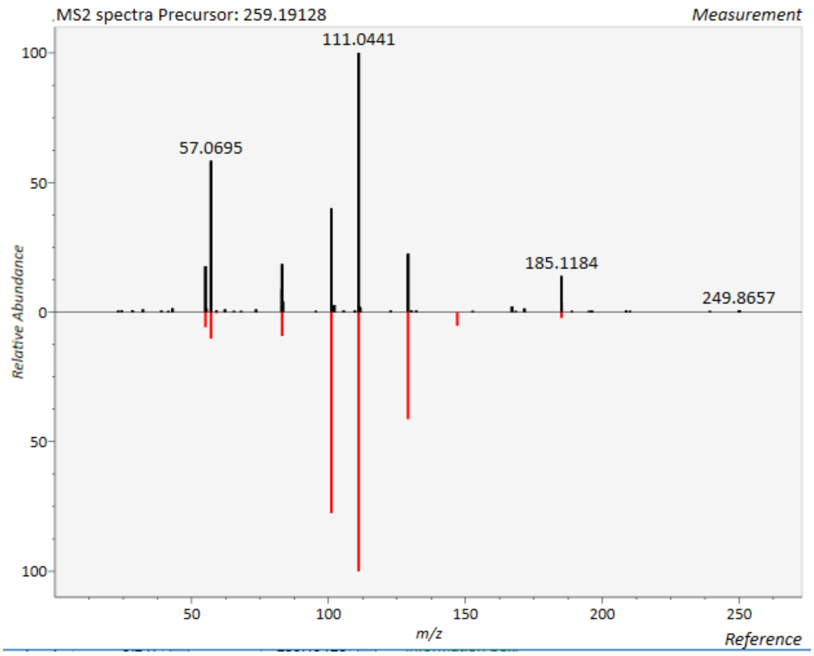

diethanolamine (1), hilic pos, m/z 106.0864 (M+H)+, MW 105.0787, RT 3.47 min, 20V, HMDB04437

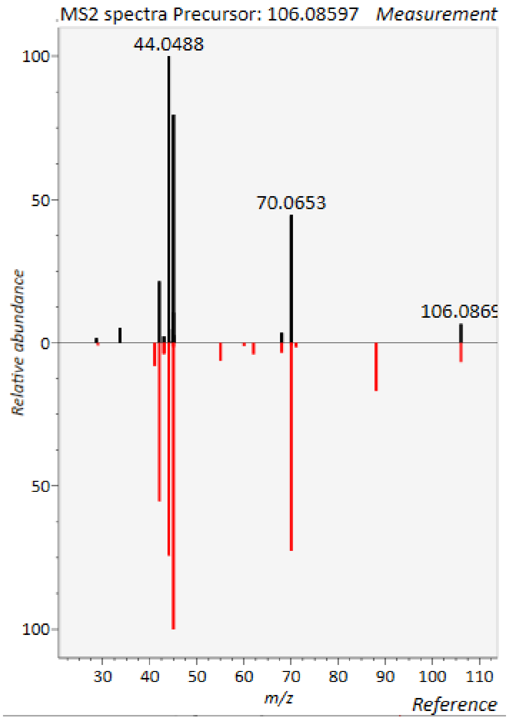

diethylhexyl adipate (DEHA) (2), RP pos, m/z 371.3165 (M+H)+, MW 370.3084, RT 10.93 min, 20V, HMDB40270

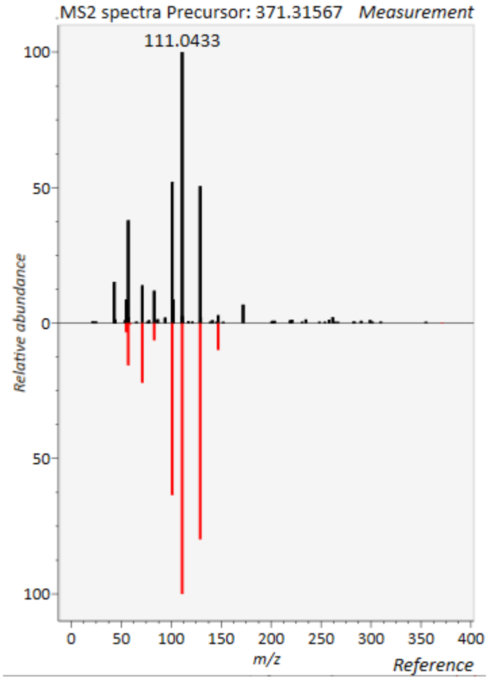

diisodecyl phthalate (2), RP pos, m/z 447.3482 (M+H)+, MW 446.3394, RT 11.51, 20V,

PubChem CID 33599

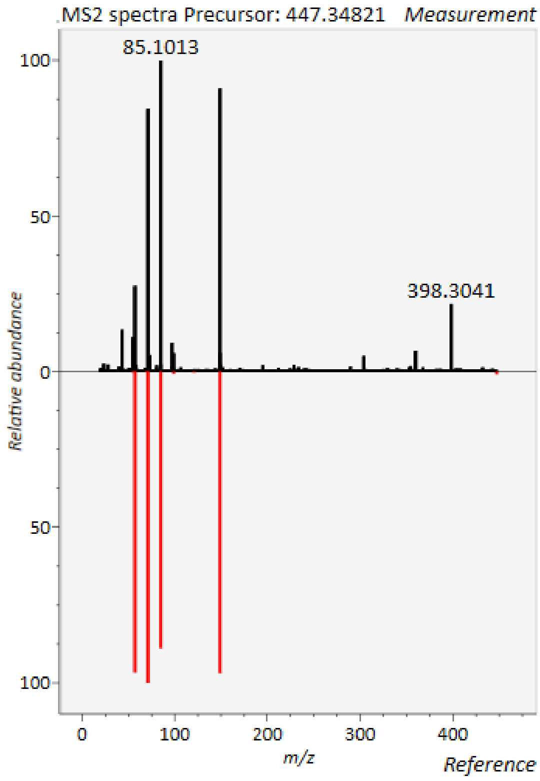

dioctyl phthalate (1), RP pos, m/z 391.285 (M+H)+, MW 390.2772, RT 10.89, 20V, PubChem CID 8346

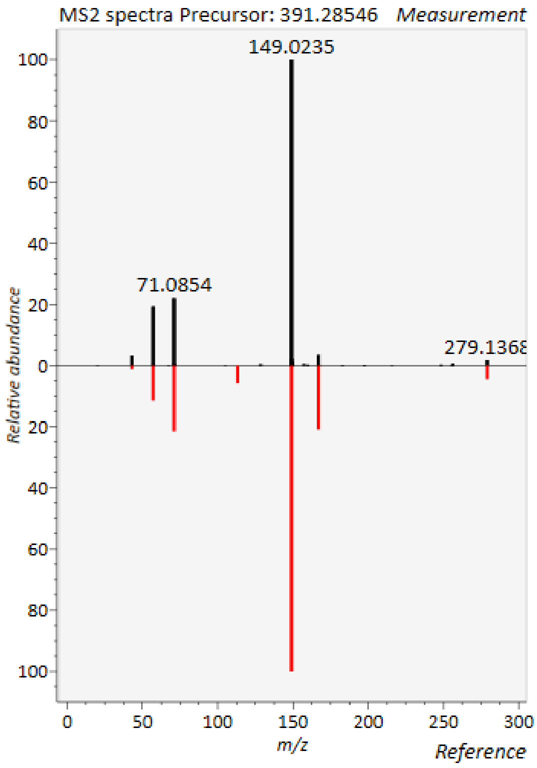

dodecyl sulfate (2), RP neg, m/z 265.1475 (M-H)-, MW 266.1547, RT 10.45, 40V, PubChem CID 4329331

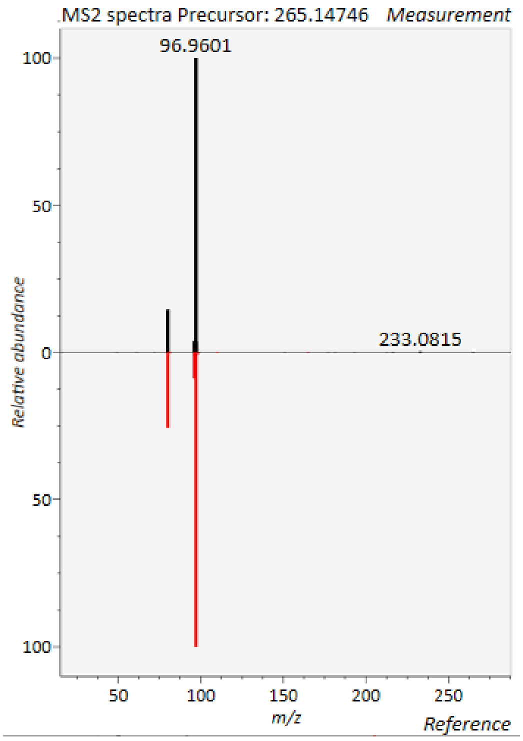

dodecylbenzenesulfonic acid (2), hilic neg, m/z 325.1835 (M-H)-, MW 326.1904, RT 0.47 min, 20V, isomers possible

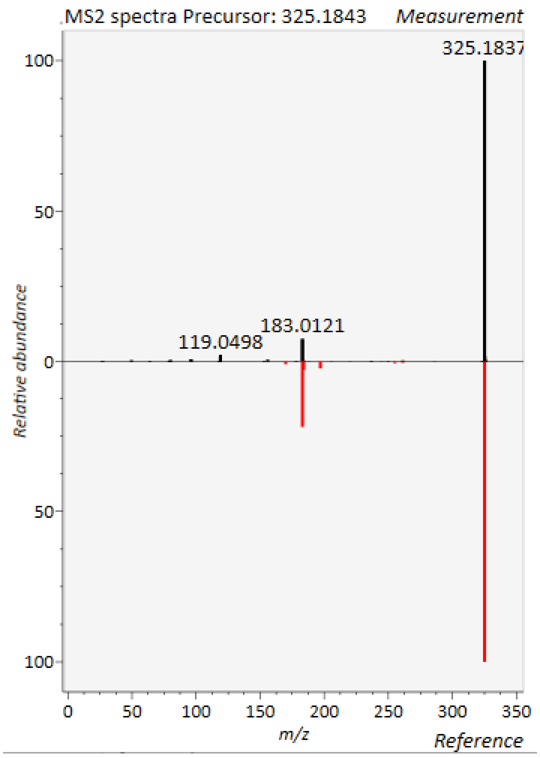

linoleamide (2), RP pos, m/z 280.2643 (M+H)+, MW 279.2567, RT 10.17 min, 20V, HMDB62656

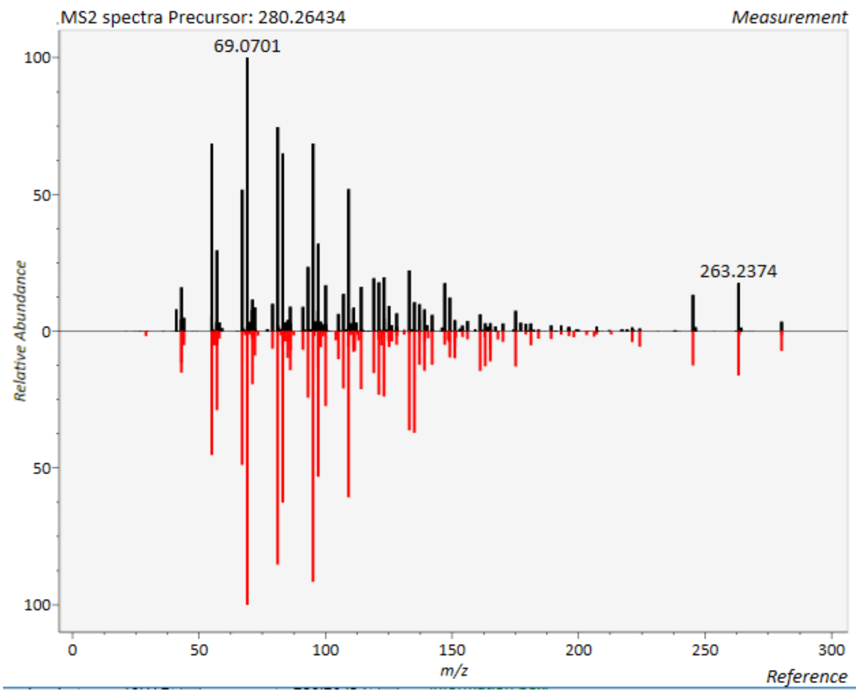

myristamide (2), RP pos, m/z 228.233 (M+H)+, MW 227.2254, RT 9.82 min, 20V, PubChem CID 69492

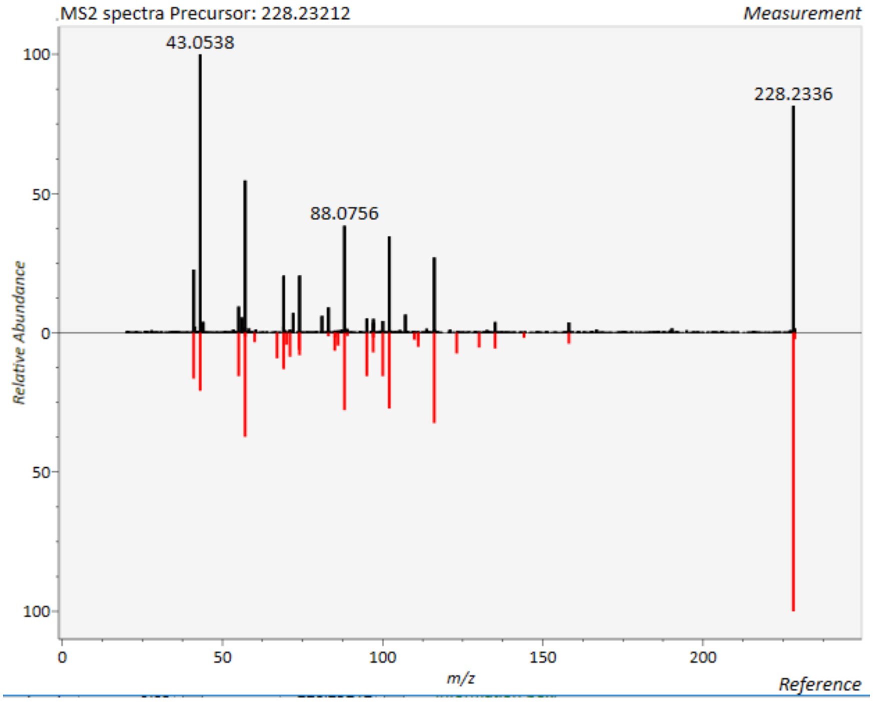

oleamide (2), RP pos, m/z 282.2799 (M+H)+, MW 281.2724, RT 10.42 min, 20V, HMDB02117

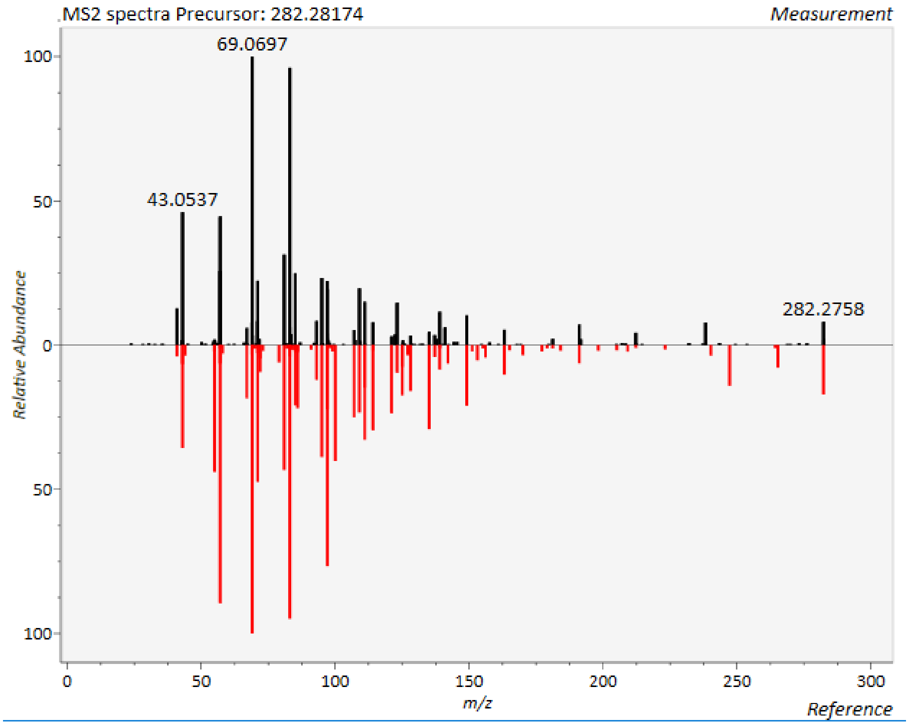

palmitoleamide (2), RP pos, m/z 254.2485 (M+H)+, MW 253.2413, RT 9.99 min, 20V,

PubChem CID 56936054

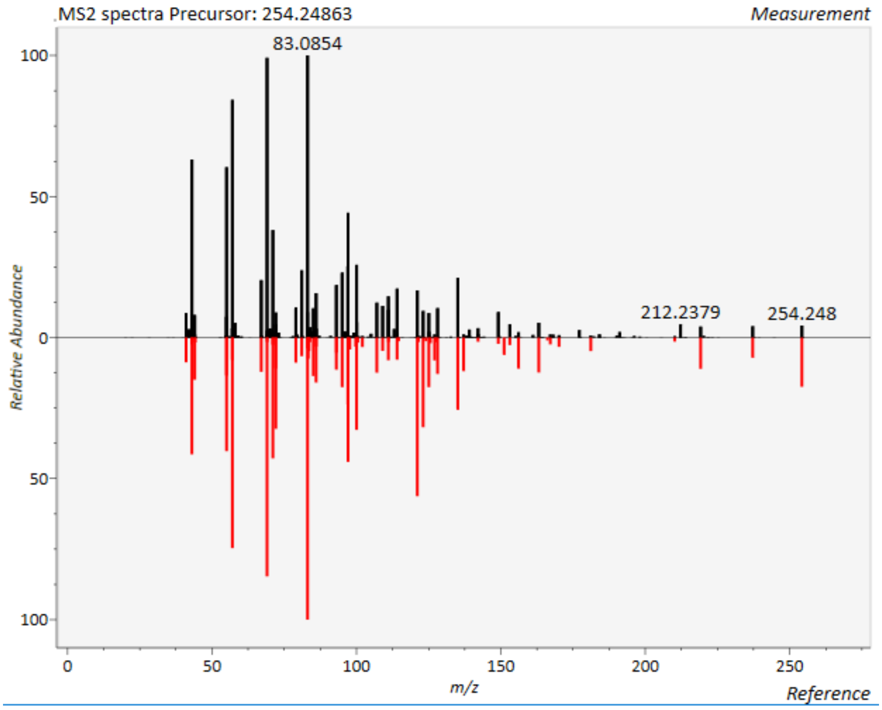

pentaerythritol tetrakis(3,5-di-tert-butyl-4-hydroxyhydrocinnamate) (2), RP neg, m/z 1175.774 (M-H)-,

MW 1176.78, RT 11.80 min, 40V, PubChem CID 64819

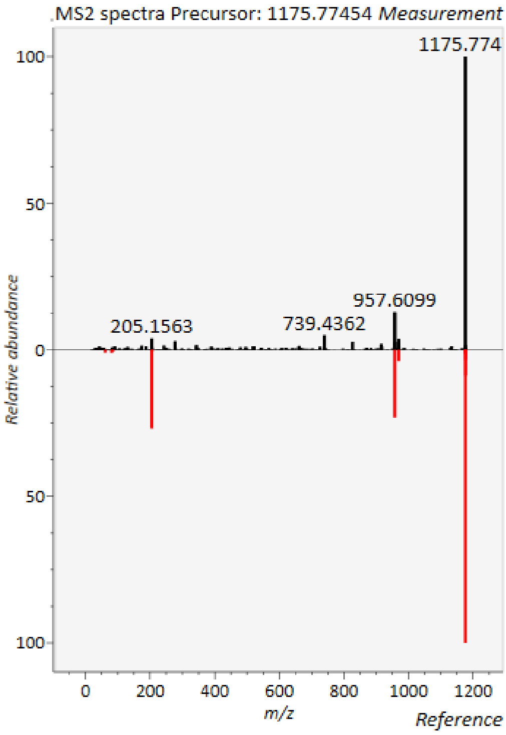

phthalic acid mono-2-ethylhexyl ester (2), RP pos, m/z 279.1598 (M+H)+, MW 278.1524, RT 9.17, 40V, HMDB13248

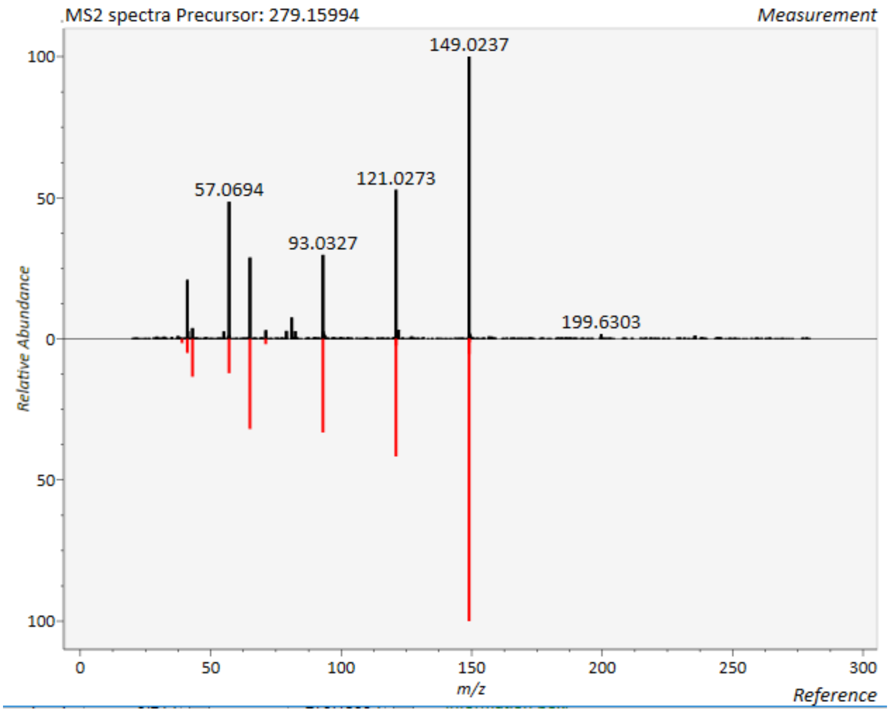

stearamide (2), RP pos, m/z 284.2953 (M+H)+, MW 283.2884, RT 10.69 min, 20V, HMDB34146

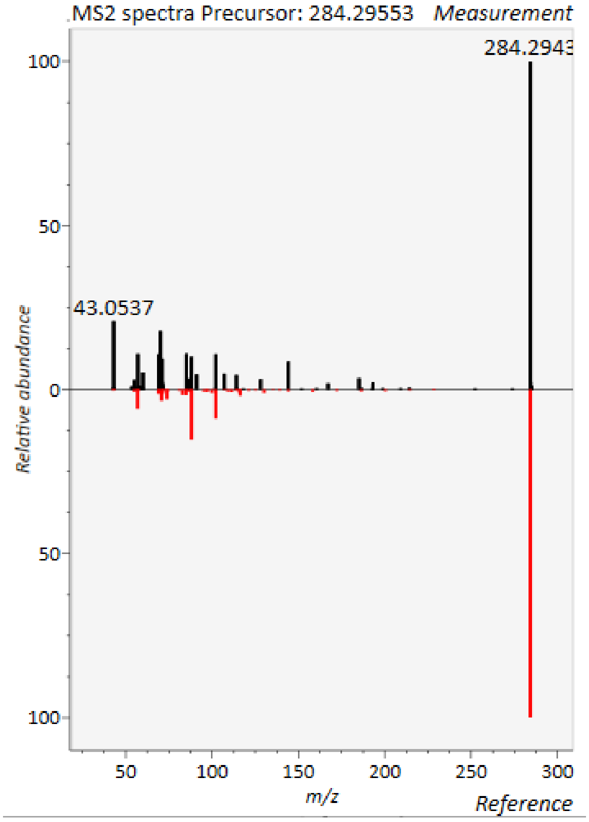

triethanolamine (2), hilic pos, m/z 150.1127 (M+H)+, MW 149.1053, RT 2.87 min, 20V, HMDB32538

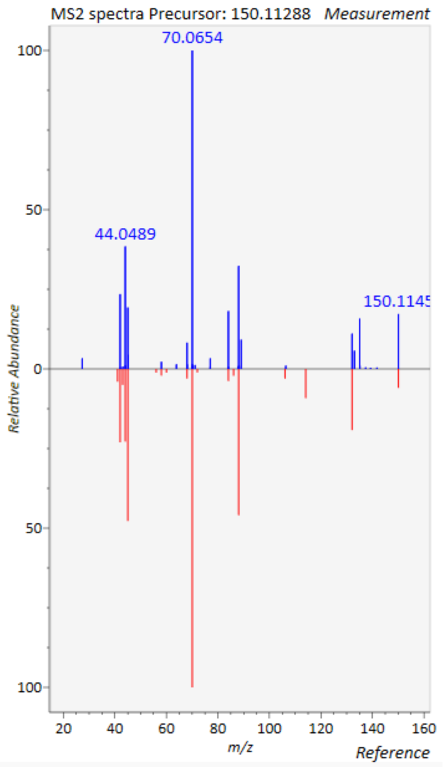

tris(hydroxymethyl)aminomethane (2), hilic pos, m/z 122.0814 (M+H)+, MW 121.0737, RT 5.17 min, 20V, HMDB0240288

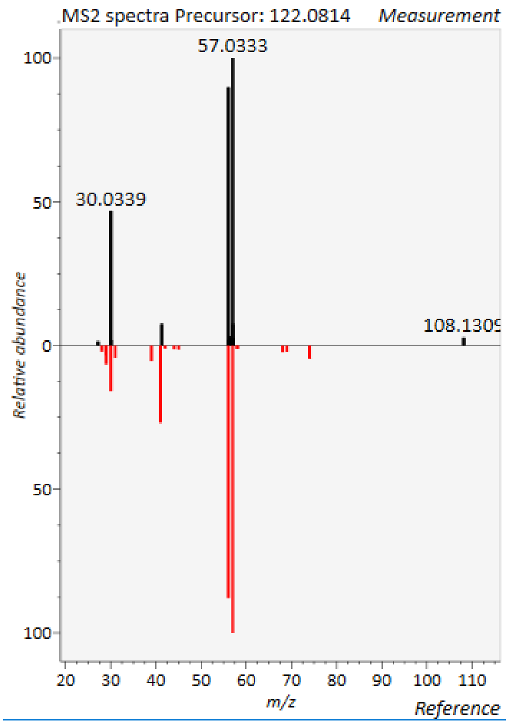

Lipids and carnitines

acetylcarnitine (1), hilic pos, m/z 204.1236 (M+H)+, MW 203.1163, RT 3.00, 20V, HMDB00201

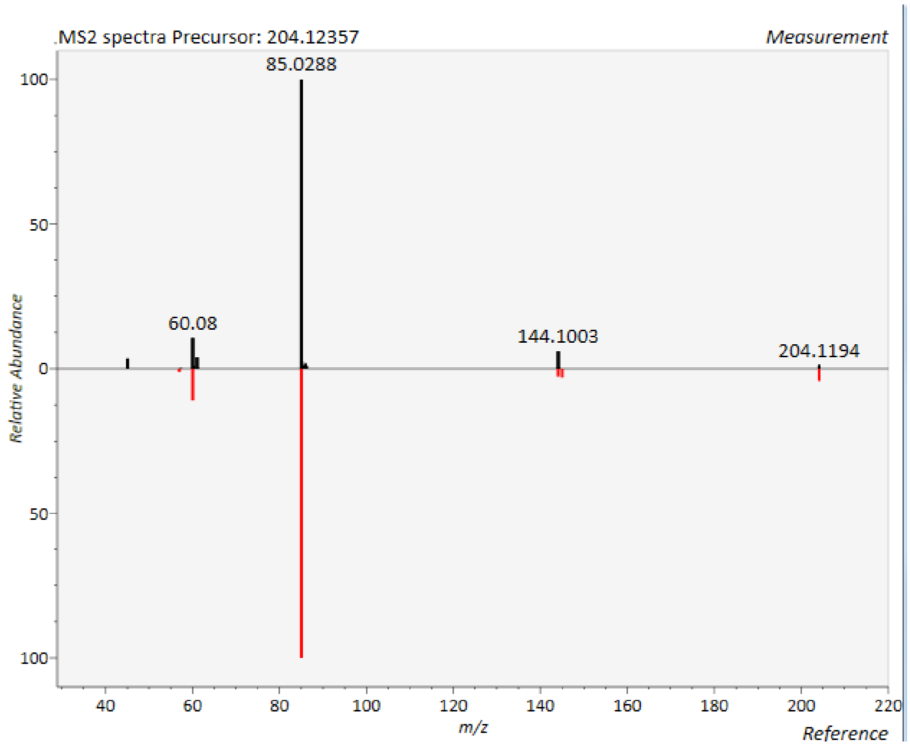

acylcarnitine C16:0 (1), RP pos, m/z 400.3429 (M+H)+, MW 399.3357, RT 9.04, 20V, HMDB00222

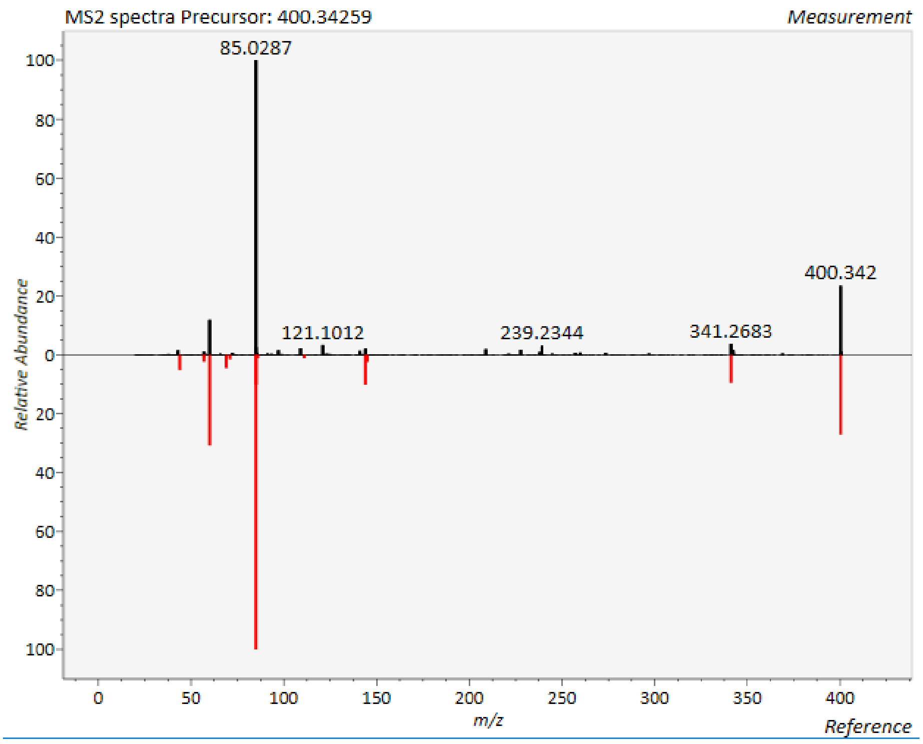

acylcarnitine C18:0 (1), RP pos, m/z 428.3744 (M+H)+, MW 427.3665, RT 9.44, 20V, HMDB00848

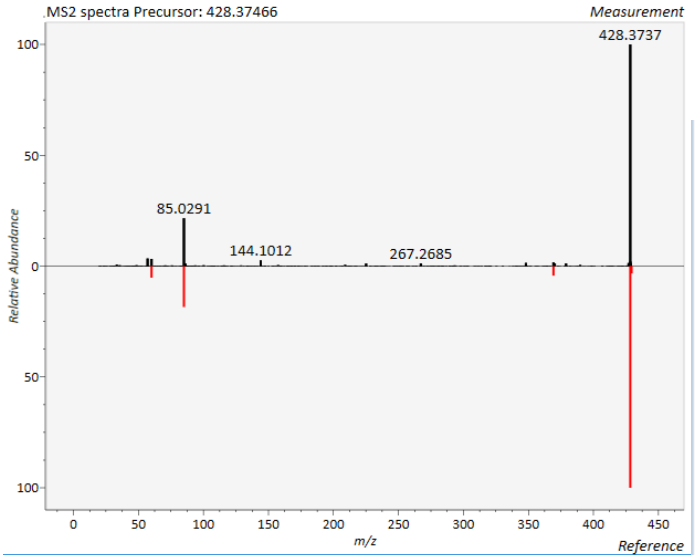

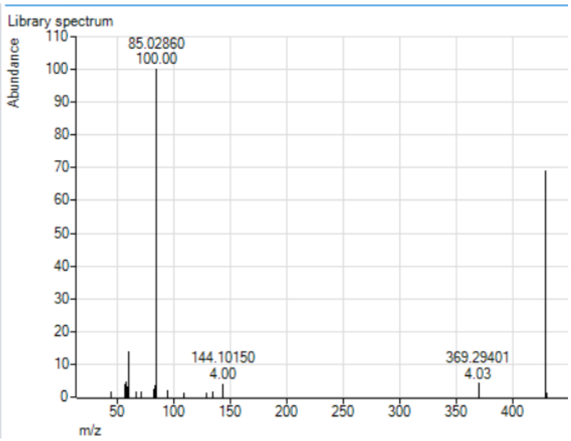

acylcarnitine C18:1 (2), RP pos, m/z 426.3589 (M+H)+, MW 425.3511, RT 9.16, 20V, HMDB06351

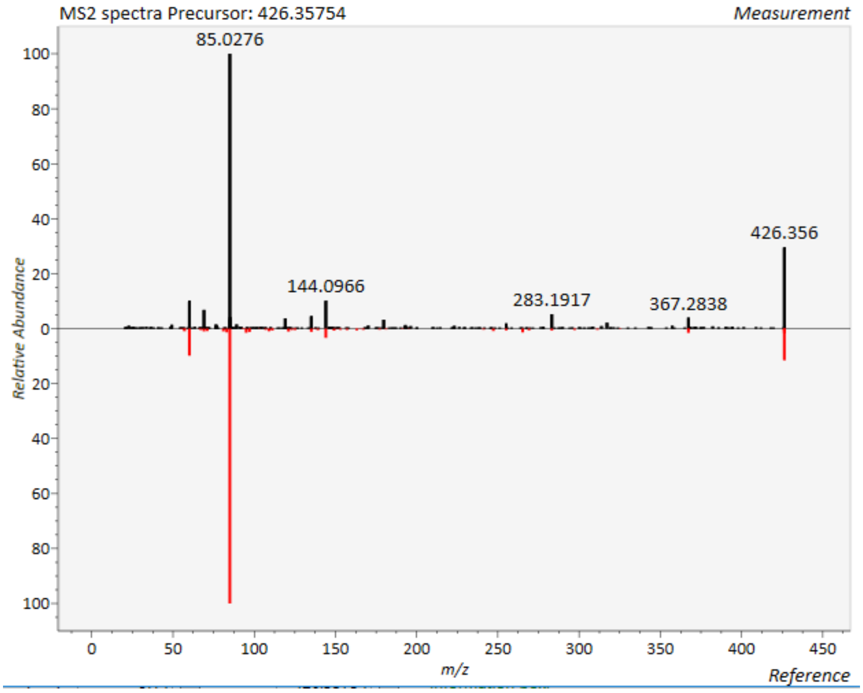

azelaic acid (1), RP neg, m/z 187.0975 (M-H)-, MW 188.1043, RT 5.59 min, 20V, HMDB00784

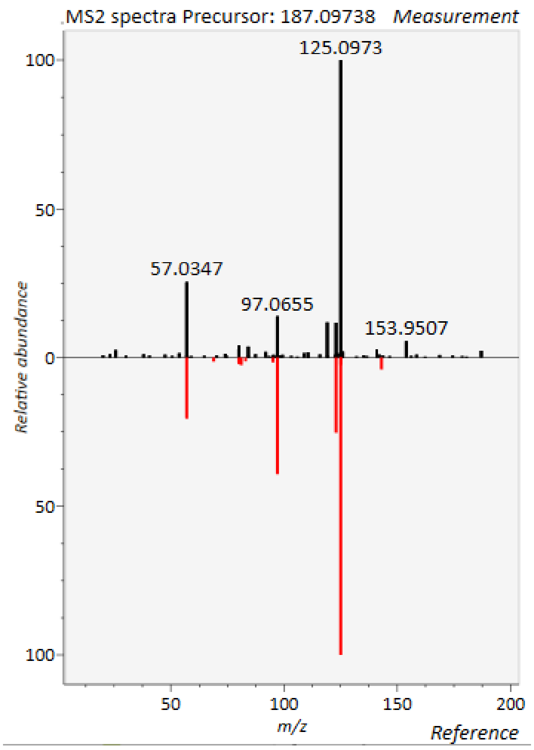

DAG 34:1 (2), RP pos, m/z 612.5573 (M+NH4)+, MW 594.5223, RT 14.24, 20V, isomers possible

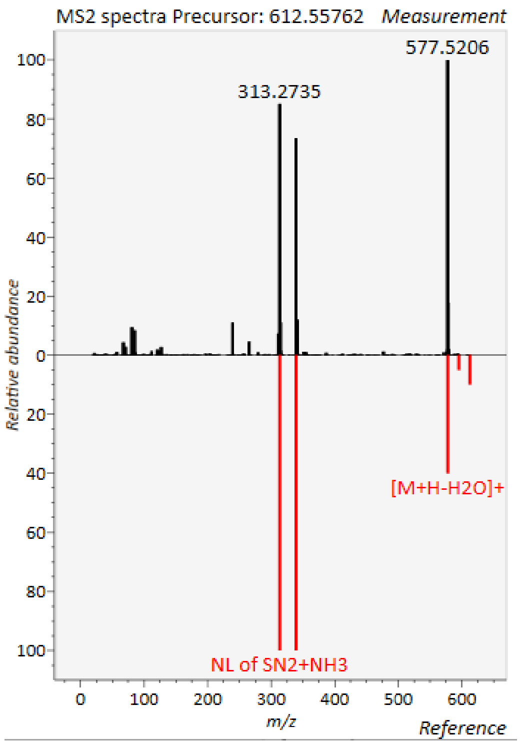

DAG 34:2 (2), RP pos, m/z 610.5417 (M+NH4)+, MW 592.5067, RT 13.46, 20V, isomers possible

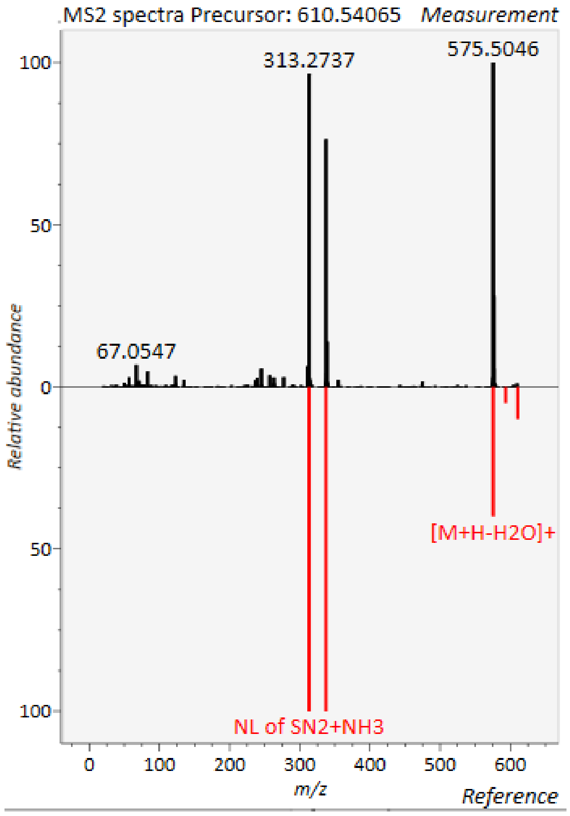

DAG 36:3 (2), RP pos, m/z 636.5575 (M+NH4)+, MW 618.5223, RT 13.67, 20V, isomers possible

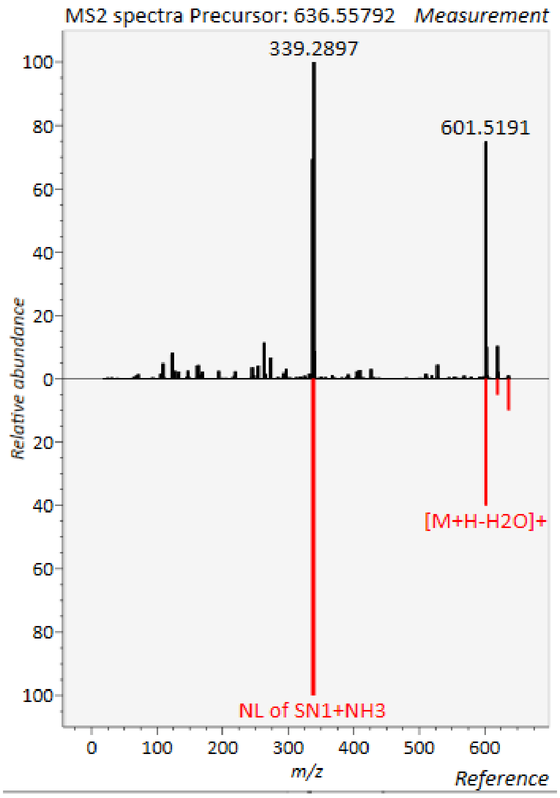

DAG 36:4 (2), RP pos, m/z 634.5416 (M+NH4)+, MW 616.5067, RT 13.03, 20V, isomers possible

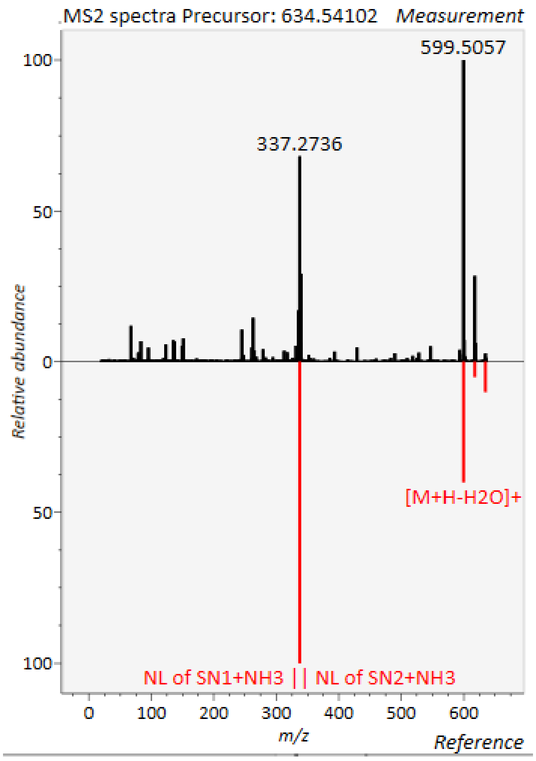

DAG 38:4 (2), RP pos, m/z 662.573 (M+NH4)+, MW 644.538, RT 14.43, 20V, isomers possible

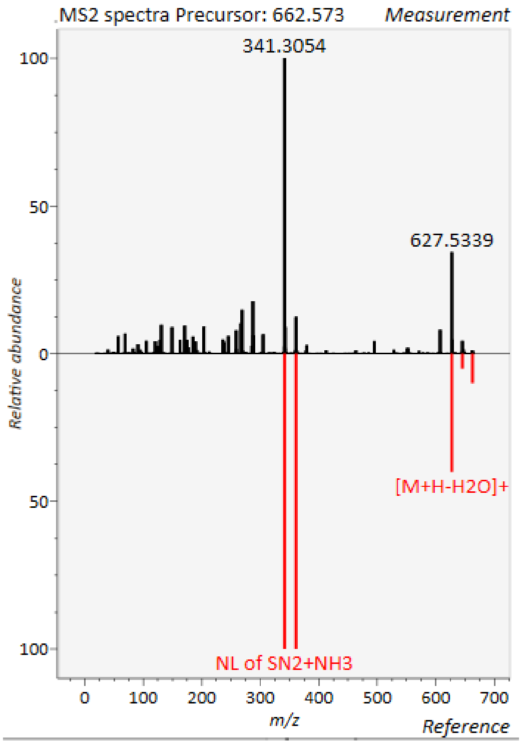

FA 15:0 (2), RP neg, m/z 241.2173 (M-H)-, MW 242.2238, RT 10.47 min, 10V, HMDB00826

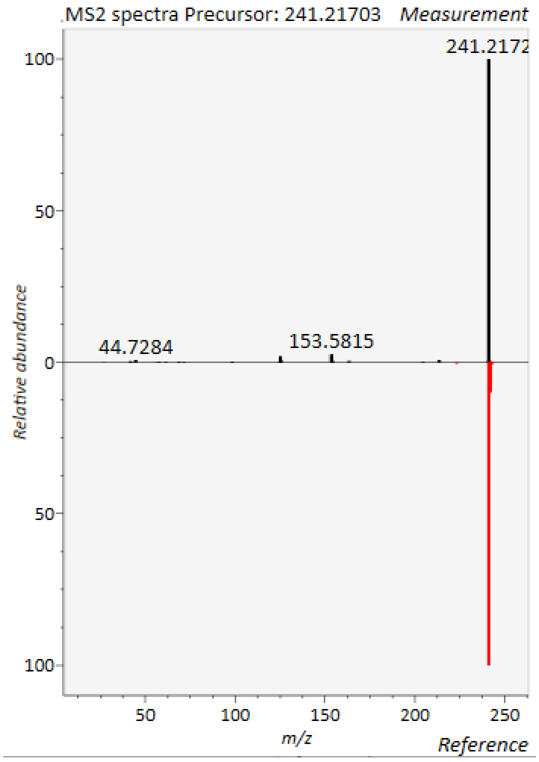

FA 16:0 (2), RP neg, m/z 255.2327 (M-H)-, MW 256.2401, RT 10.70 min, 10V, HMDB00220

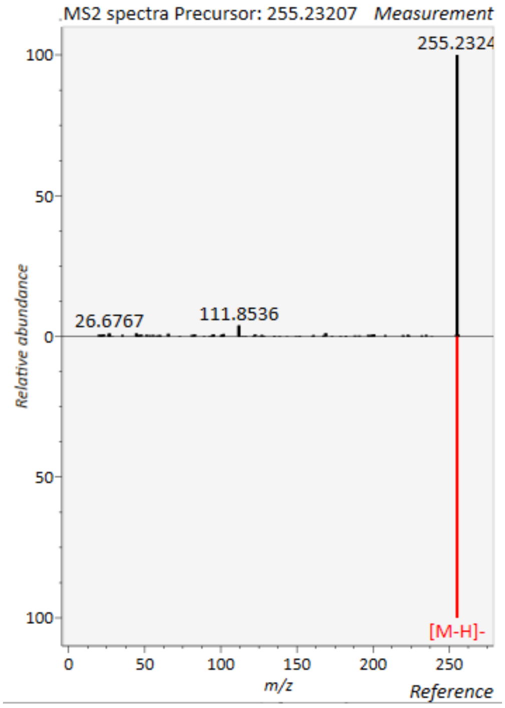

FA 16:1 (2), RP neg, m/z 253.2168 (M-H)-, MW 254.2239, RT 10.44 min, 10V, isomers possible

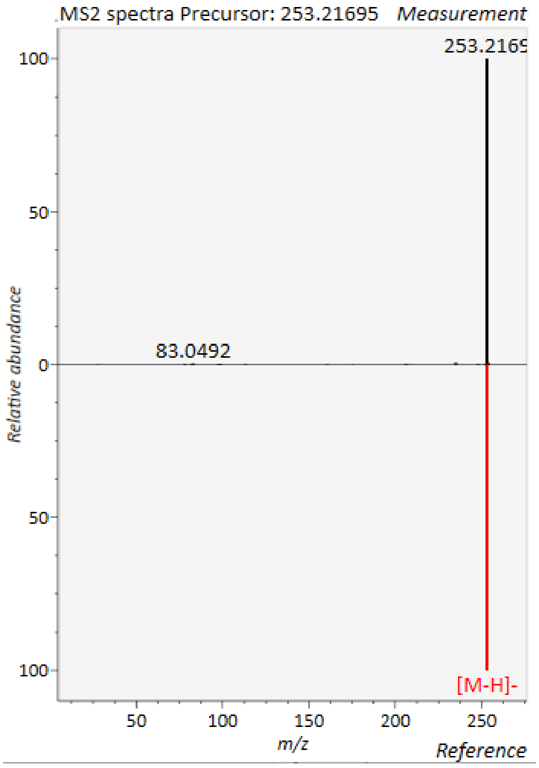

FA 17:0 (1), RP neg, m/z 269.2483 (M-H)-, MW 270.2554, RT 10.82 min, 20V, HMDB02259

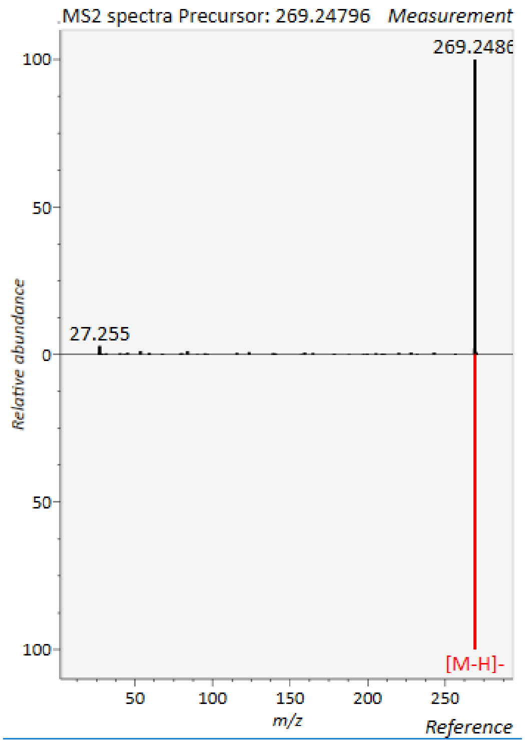

FA 17:1 (2), RP neg, m/z 267.2323 (M-H)-, MW 268.2398, RT 10.62 min, 10V, isomers possible

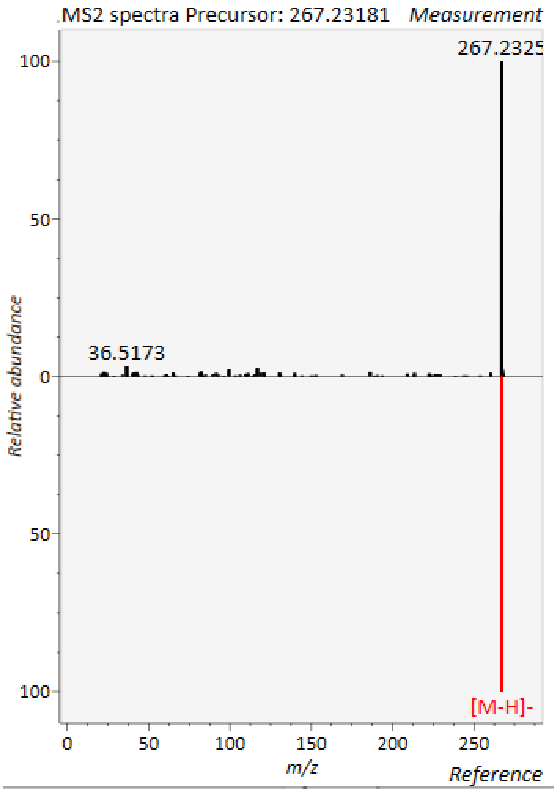

FA 18:0 (1), RP neg, m/z 283.2639 (M-H)-, MW 284.2713, RT 11.03 min, 20V, HMDB00827

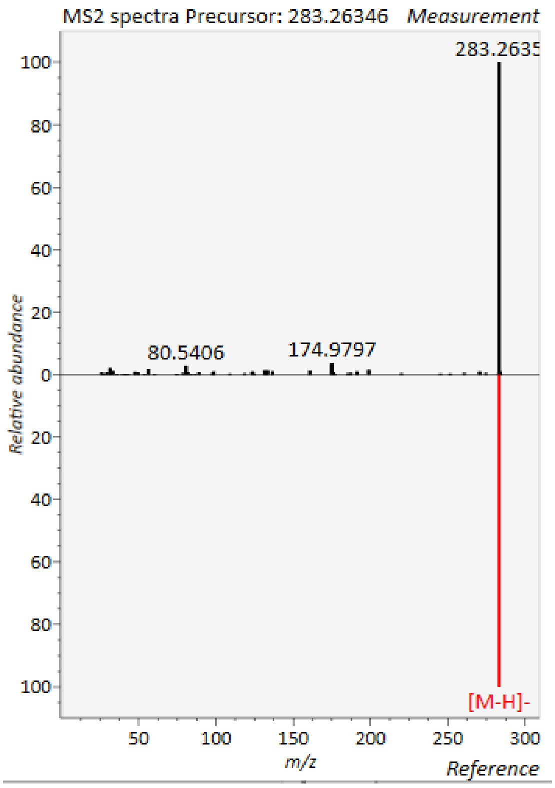

FA 18:1 (1), RP neg, m/z 281.2479 (M-H)-, MW 282.2556, RT 10.77 min, 20V, HMDB00207

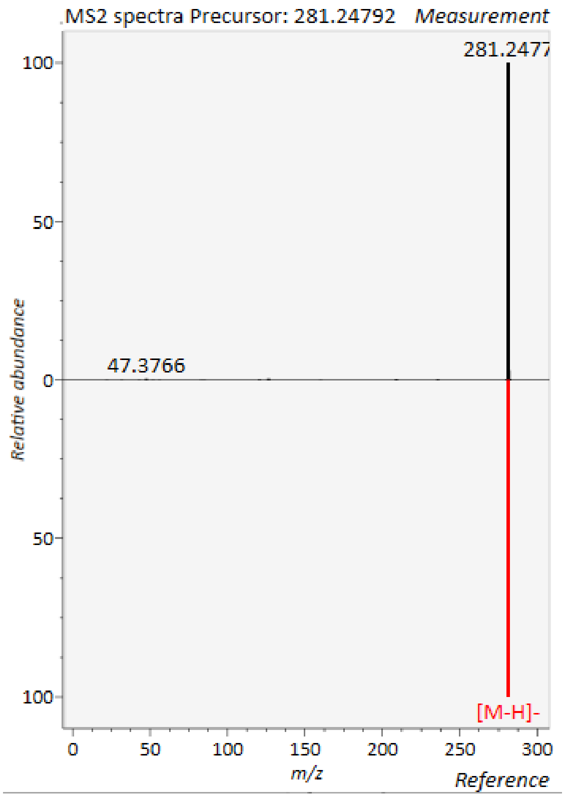

FA 18:2 (2), RP neg, m/z 279.2324 (M-H)-, MW 280.2401, RT 10.56 min, 10V, HMDB00673

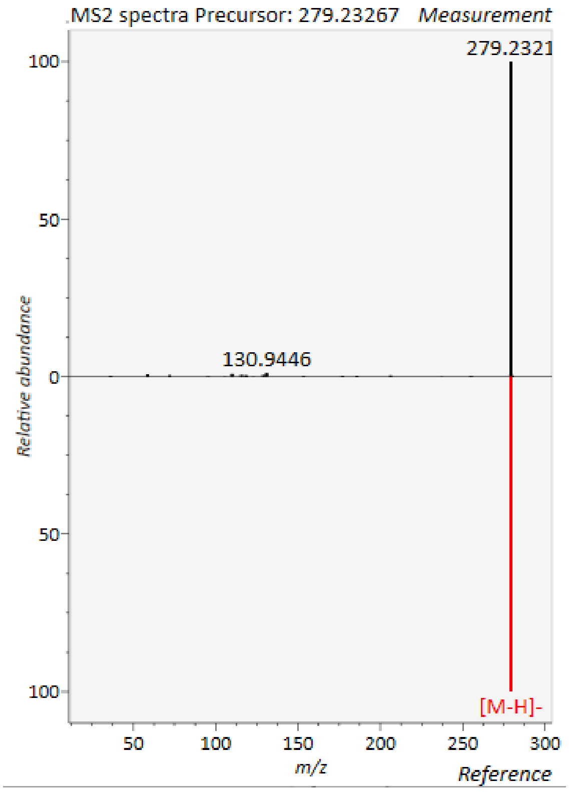

FA 18:3 (2), RP neg, m/z 277.217 (M-H)-, MW 278.2237, RT 10.34 min, 10V, isomers possible

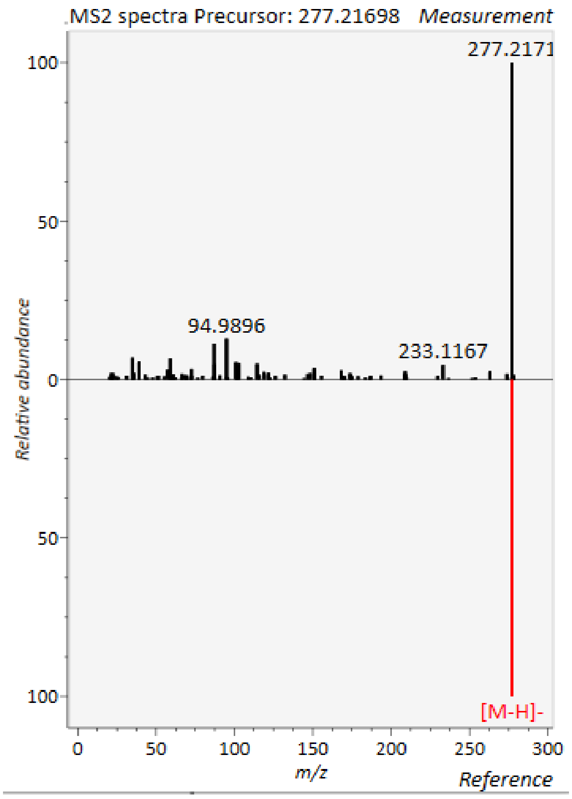

FA 20:0 (1), RP neg, m/z 311.2951 (M-H)-, MW 312.3017, RT 11.42 min, 20V, HMDB02212

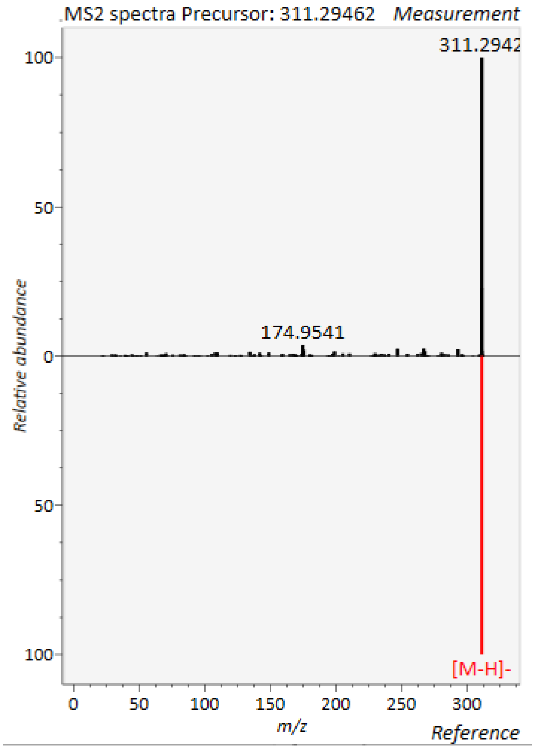

FA 20:1 (2), RP neg, m/z 309.2793 (M-H)-, MW 310.2863, RT 11.09 min, 10V, isomers possible

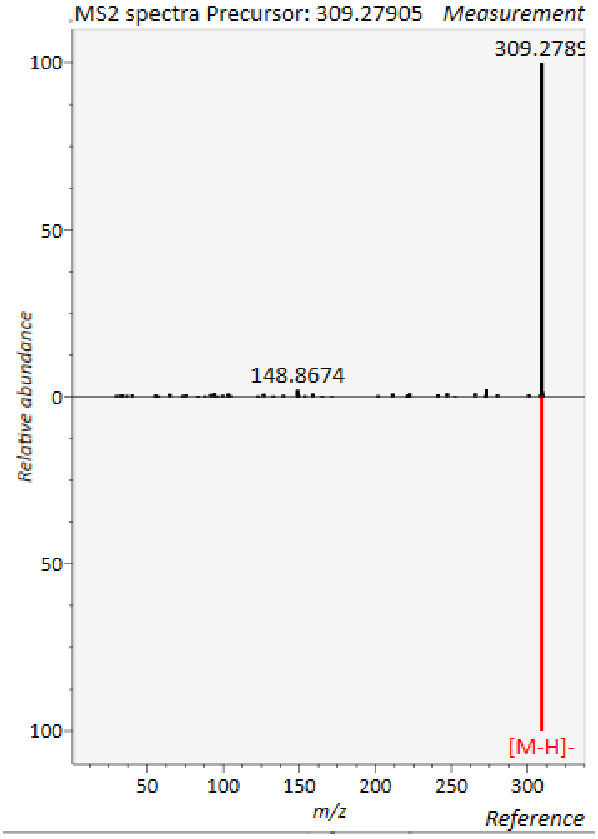

FA 20:2 (2), RP neg, m/z 307.2637 (M-H)-, MW 308.2705, RT 10.87 min, 10V, isomers possible

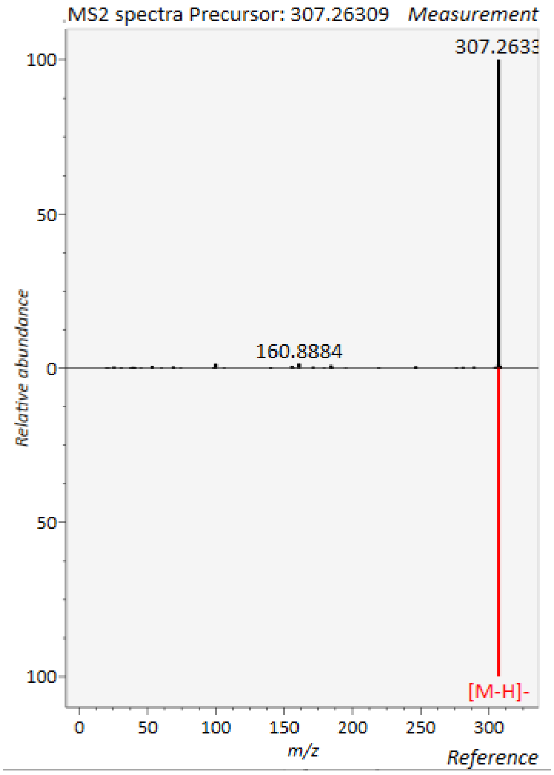

FA 20:3 (2), RP neg, m/z 305.2477 (M-H)-, MW 306.2552, RT 10.68 min, 10V, isomers possible

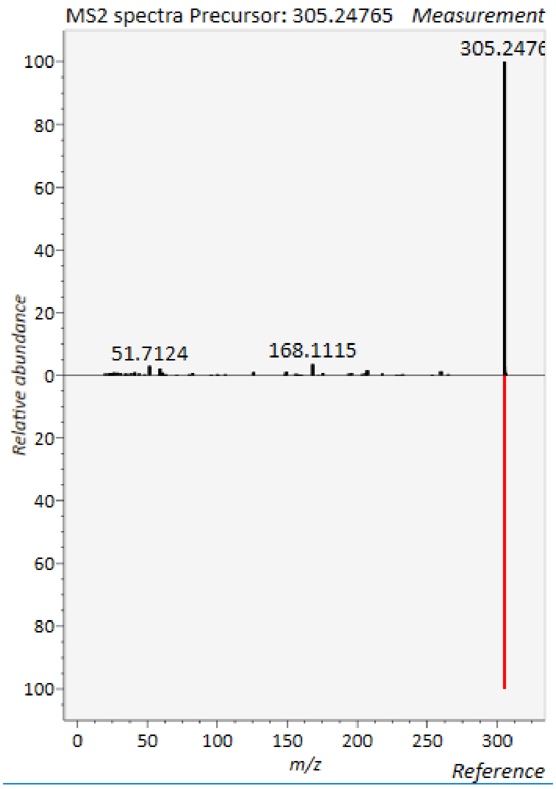

FA 20:4 (2), RP neg, m/z 303.2327 (M-H)-, MW 304.2394, RT 10.53 min, 10V, HMDB01043

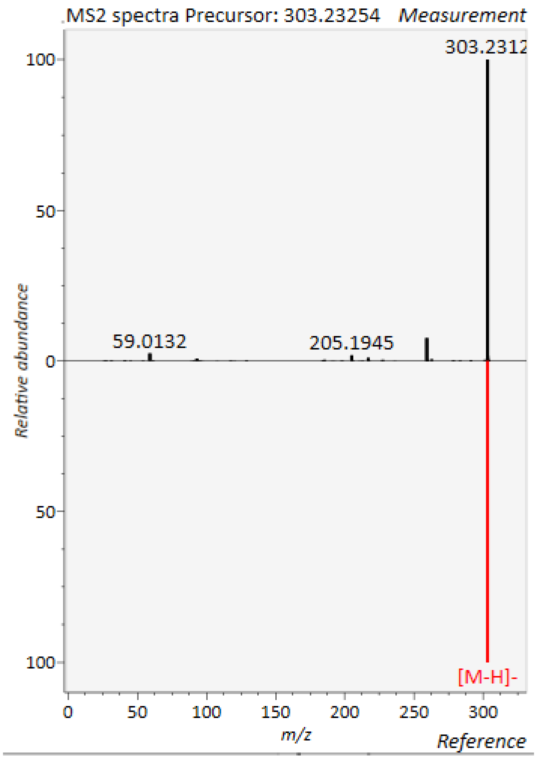

FA 20:5 (2), RP neg, m/z 301.2167 (M-H)-, MW 302.2238, RT 10.39 min, 20V, HMDB01999

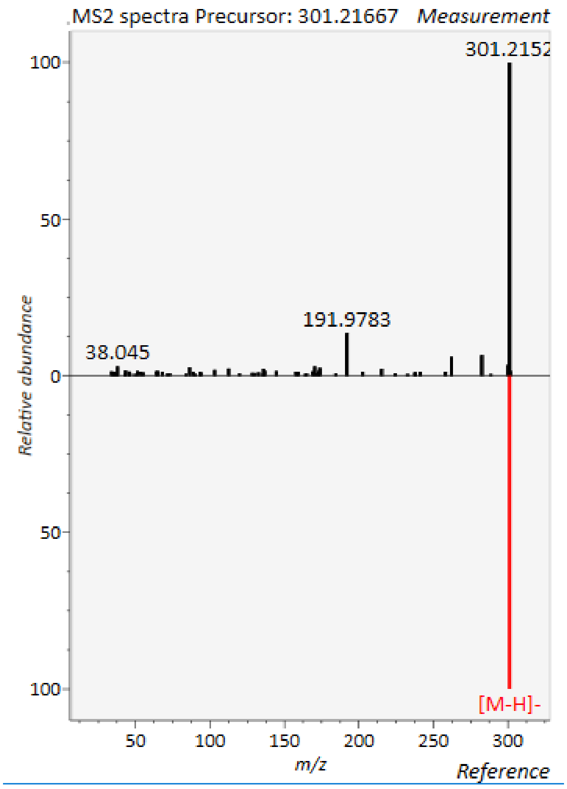

FA 21:0 (2), RP neg, m/z 325.3106 (M-H)-, MW 326.3178, RT 11.58 min, 10V, HMDB02345

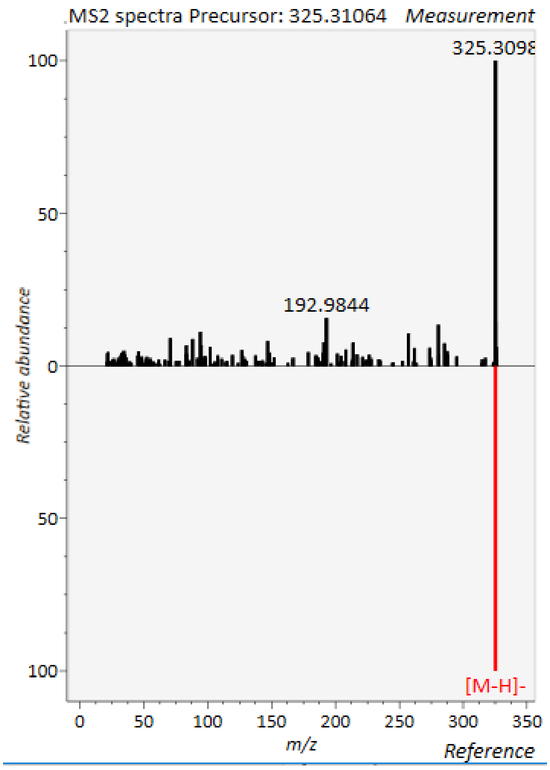

FA 22:0 (2), RP neg, m/z 339.3261 (M-H)-, MW 340.3333, RT 11.91 min, 20V, HMDB00944

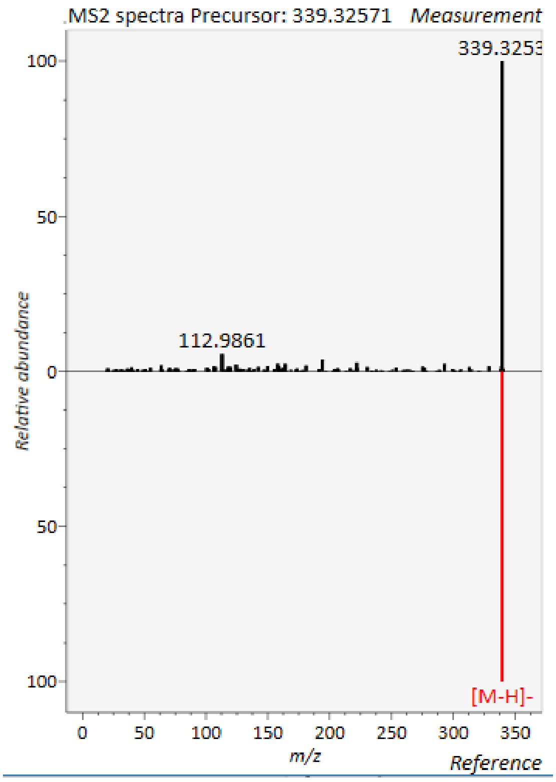

FA 22:1 (1), RP neg, m/z 337.3105 (M-H)-, MW 338.3177, RT 11.48 min, 20V, HMDB02068

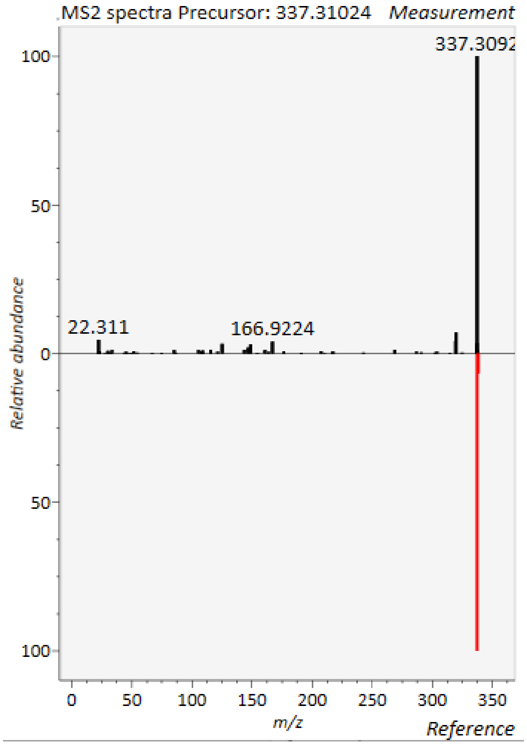

FA 22:2 (2), RP neg, m/z 335.2948 (M-H)-, MW 336.3019, RT 11.20 min, 10V, isomers possible

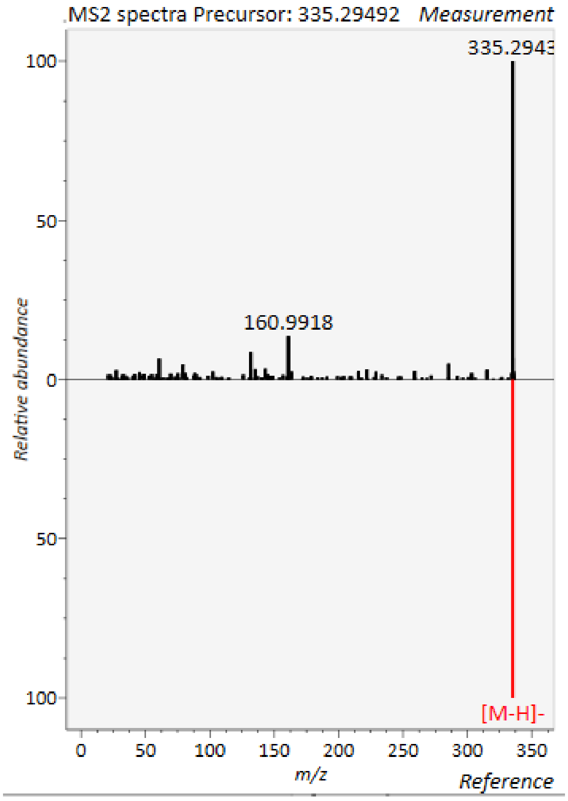

FA 22:3 (2), RP neg, m/z 333.2792 (M-H)-, MW 334.2855, RT 10.99 min, 10V, HMDB02823

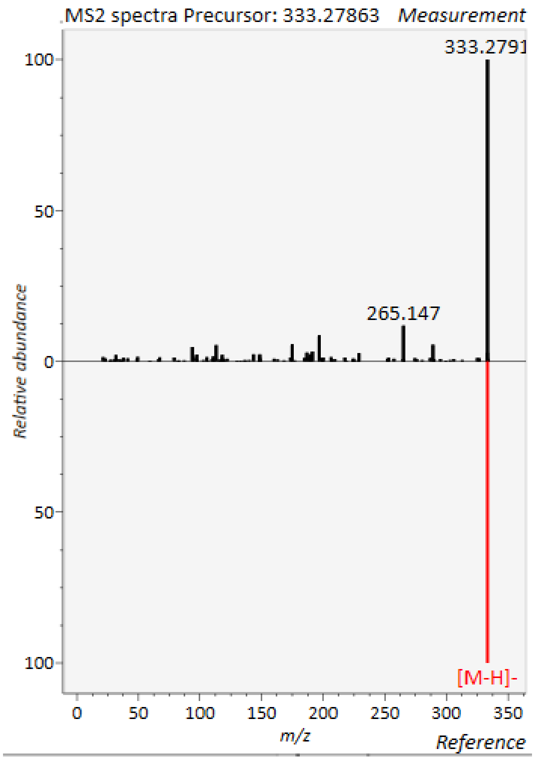

FA 22:4 (2), RP neg, m/z 331.2635 (M-H)-, MW 332.2706, RT 10.79 min, 10V, HMDB02226

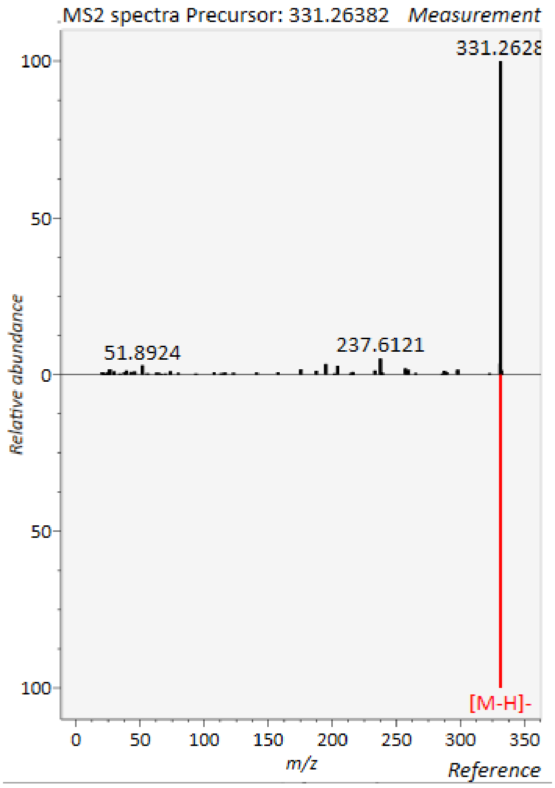

FA 24:1 (n-9) (nervonic acid) (1), RP neg, m/z 365.3417 (M-H)-, MW 366.3487, RT 11.98 min, 20V, HMDB02368

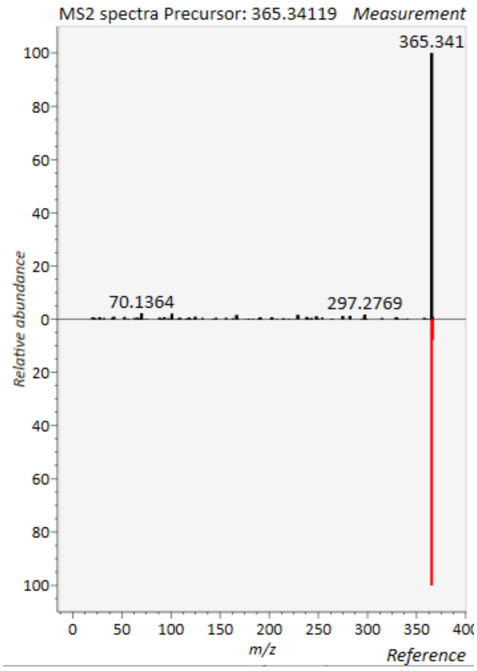

gamma-butyrobetaine (1), hilic pos, m/z 146.1177 (M+H)+, MW 145.1104, RT 3.38 min, 10V, HMDB01161

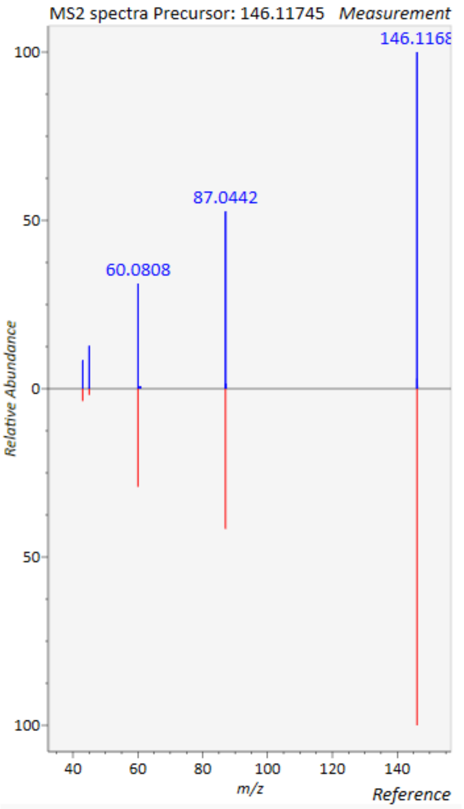

glycerophosphocholine (1), hilic pos, m/z 258.1107 (M+H)+, MW 257.1029, RT 6.12, 20V, HMDB00086

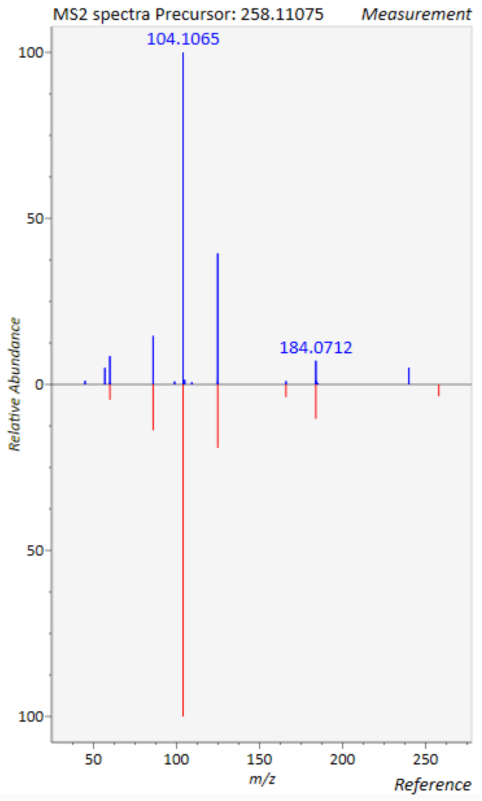

hydroxypalmitic acid (2), RP neg, m/z 271.2276 (M-H)-, MW 272.2344, RT 10.28 min, 20V, isomers possible

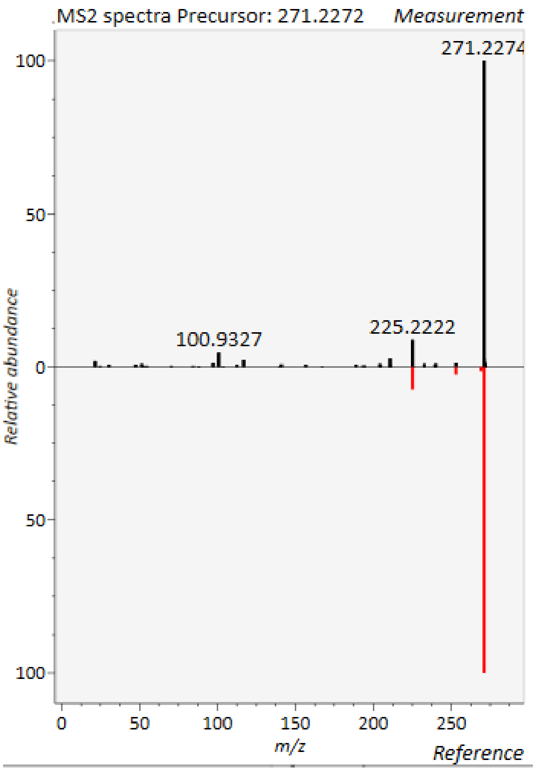

isobutyryl carnitine (2), hilic pos, m/z 232.155 (M+H)+, MW 231.1473, RT 1.59, 20V, HMDB00736

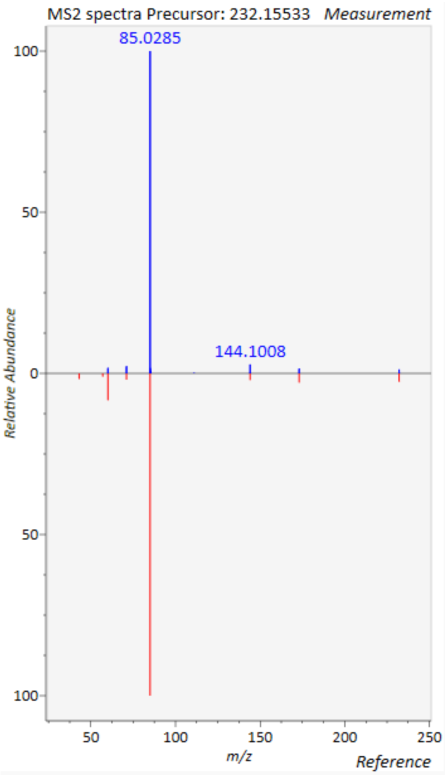

isovalerylcarnitine (1), hilic pos, m/z 246.1705 (M+H)+, MW 245.1634, RT 1.29, 20V, HMDB00688

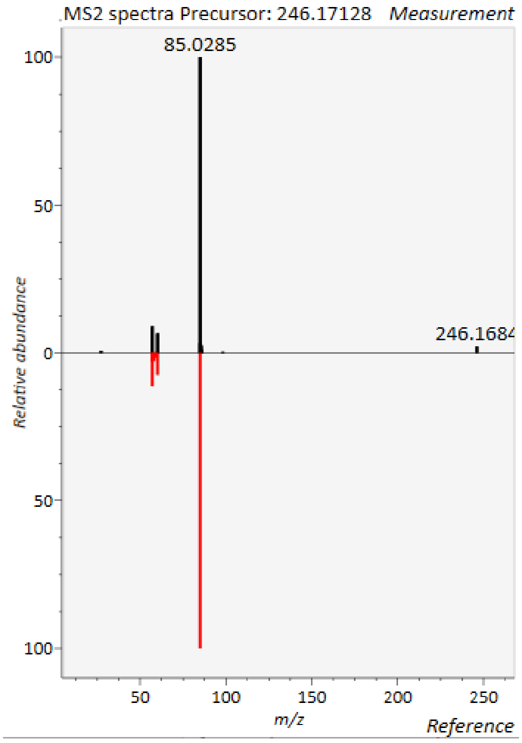

leucic acid (2), RP neg, m/z 131.0713 (M-H)-, MW 132.0787, RT 4.11 min, 10V, HMDB00665

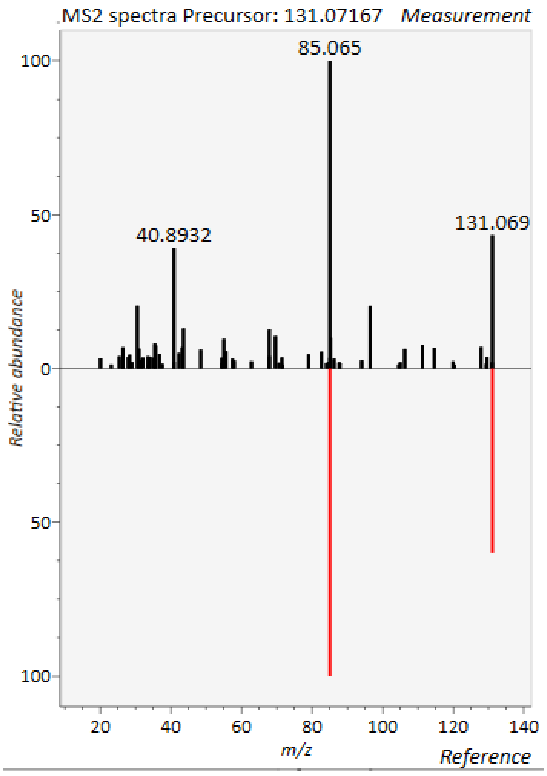

LPC 16:0 (2), left: RP pos, m/z 496.341 (M+H)+, MW 495.3333, RT 10.13 min, 10V

right: RP neg, m/z 540.3295 (M+FA-H)-, RT 10.14 min, 20V, isomers possible

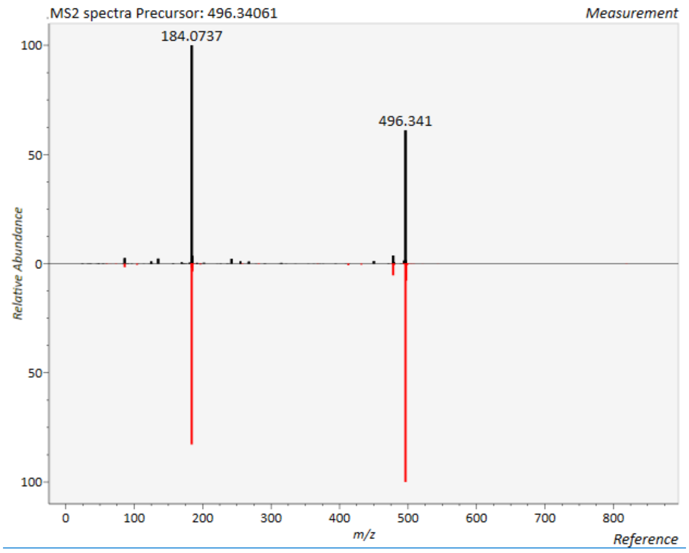

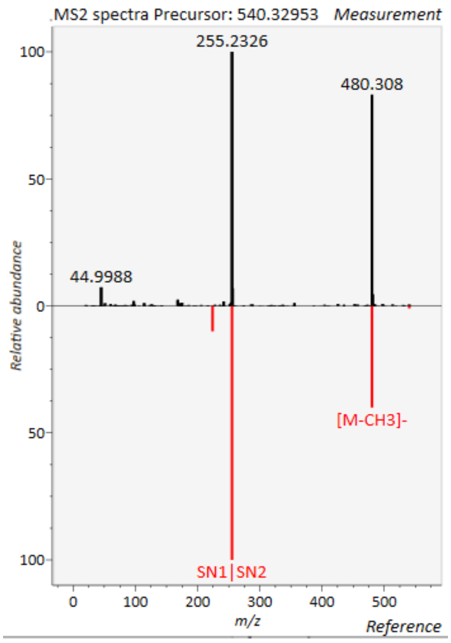

LPC 16:1 (2), left: RP pos, m/z 494.3255 (M+H)+, MW 493.3179, RT 9.77 min, 20V

right: RP neg, m/z 538.3130 (M+FA-H)-, RT 9.81 min, 20V, isomers possible

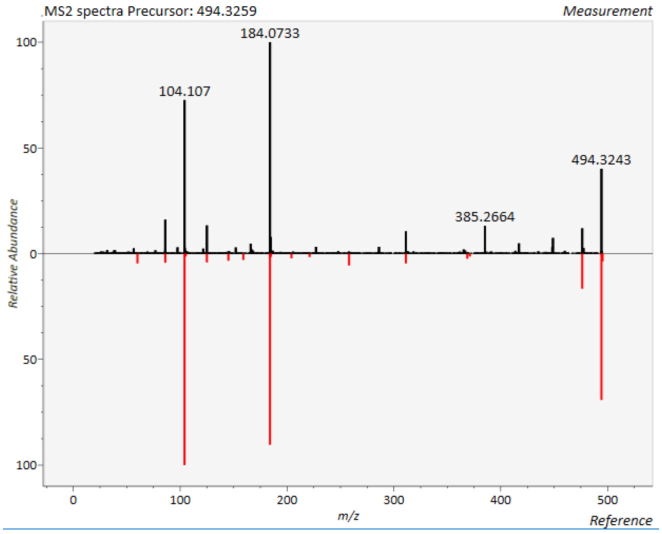

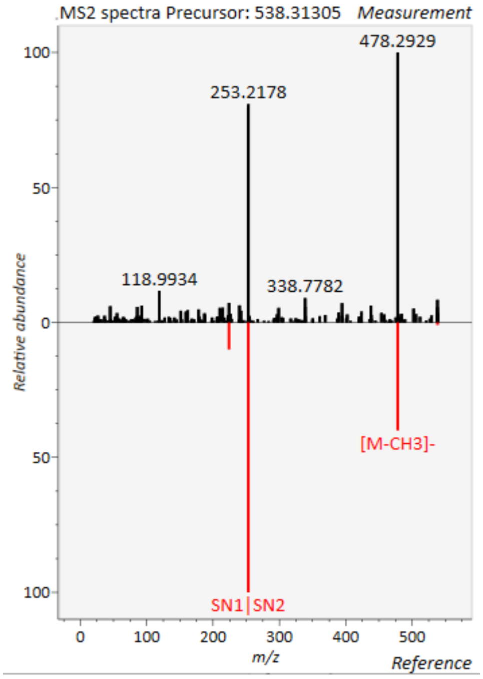

LPC 18:0 (2), left: RP pos, m/z 524.3725 (M+H)+, MW 523.3645, RT 10.53 min, 20V

right: RP neg, m/z 568.3602 (M+FA-H)-, RT 10.54 min, 20V, isomers possible

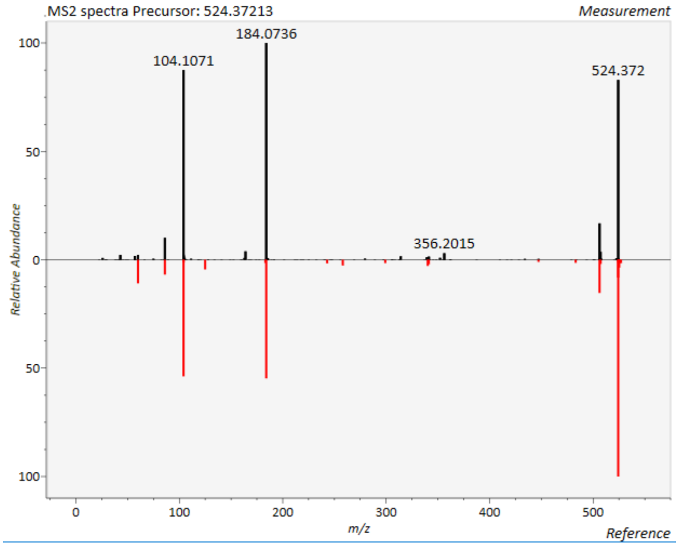

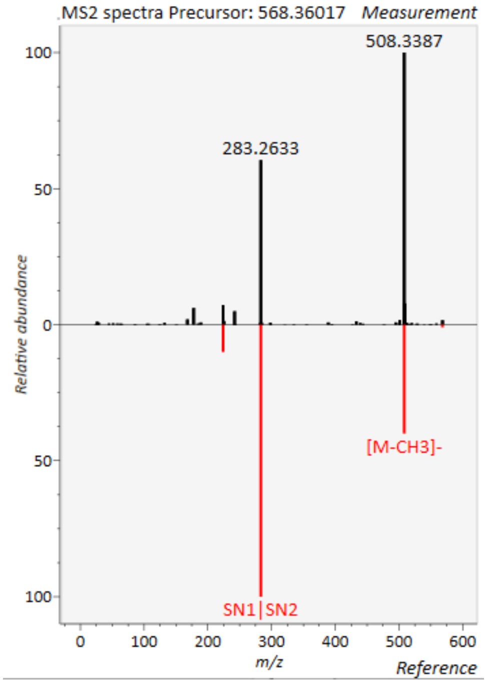

LPC 18:1 (2), left: RP neg, m/z 566.3453 (M-H)-, MW 567.3523, RT 10.26 min, 20V

right: RP pos, m/z 522.3559 (M+H)+, RT 10.24 min, 20V, isomers possible

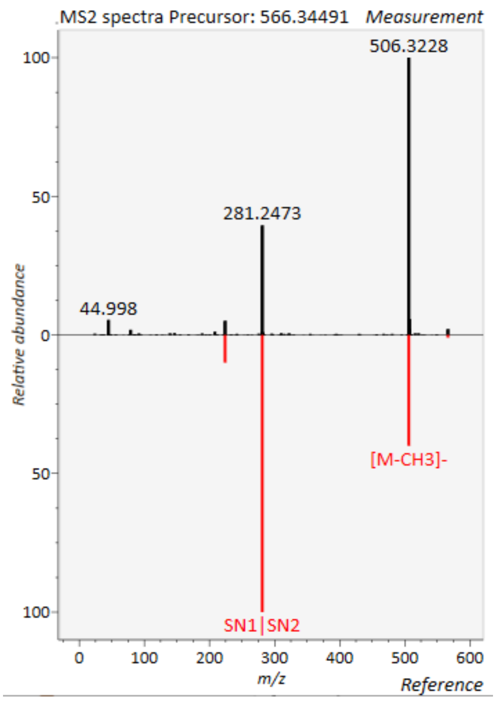

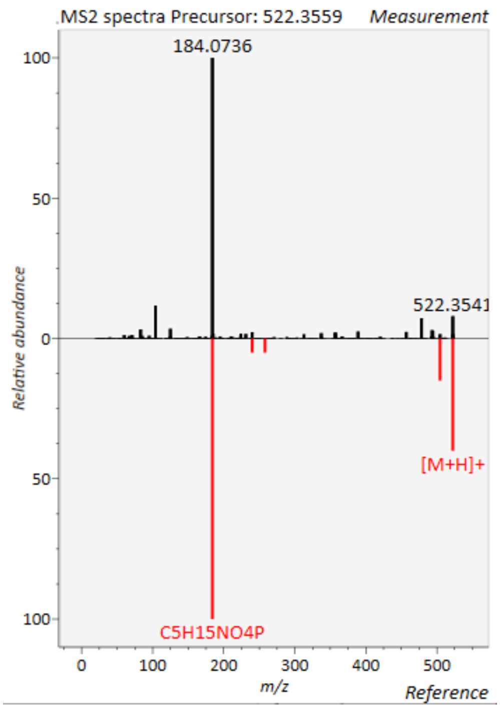

LPC 18:2 (2), left: RP neg, m/z 564.3296 (M-H)-, MW 565.3369, RT 9.99 min, 20V

right: RP pos, m/z 520.3404 (M+H)+, RT 9.95 min, 20V, isomers possible

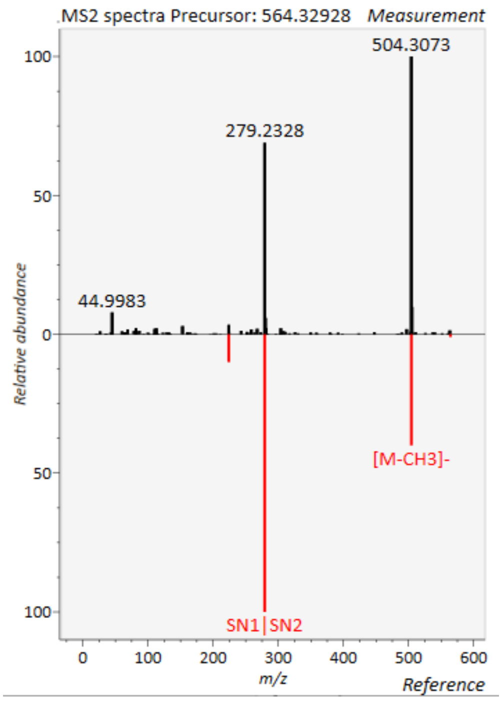

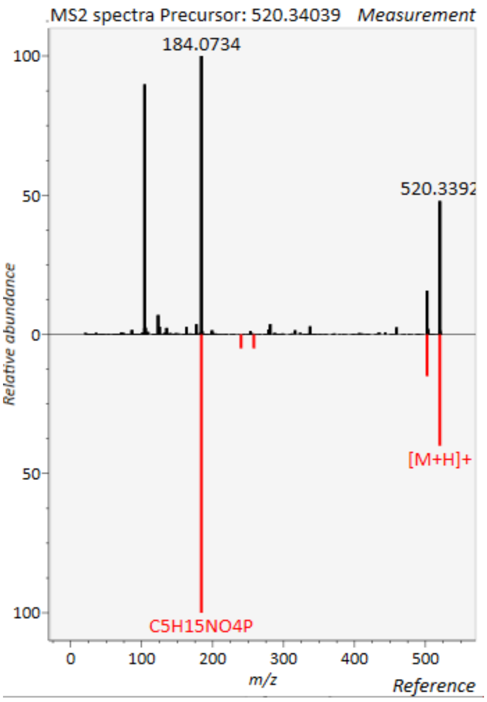

LPE 16:0 (2), left: RP neg, m/z 452.2773 (M-H)-, MW 453.2849, RT 10.10 min, 20V

right: RP pos, m/z 454.2934 (M+H)+, RT 10.09 min, 20 V, isomers possible

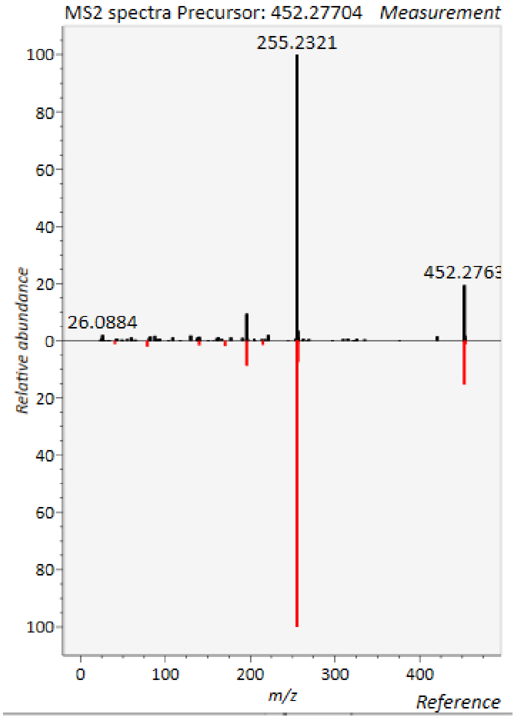

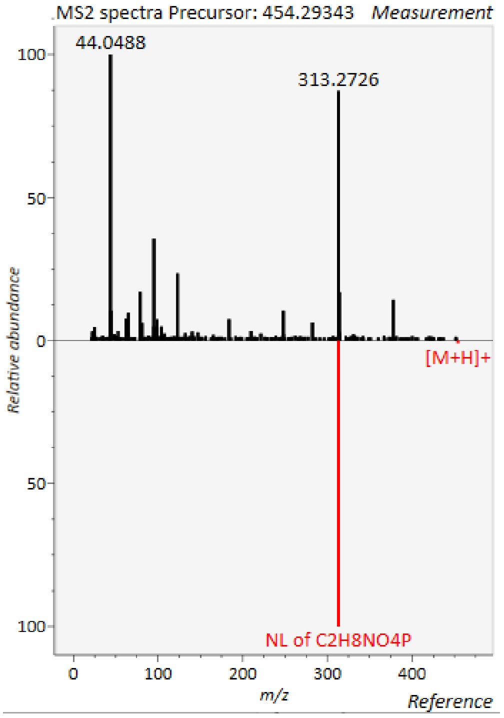

LPE 16:1 (2), left: RP neg, m/z 450.2618 (M-H)-, MW 451.2687, RT 9.78 min, 20V

right: RP pos, m/z 452.2773 (M+H)+, RT 9.76 min, 20V, isomers possible

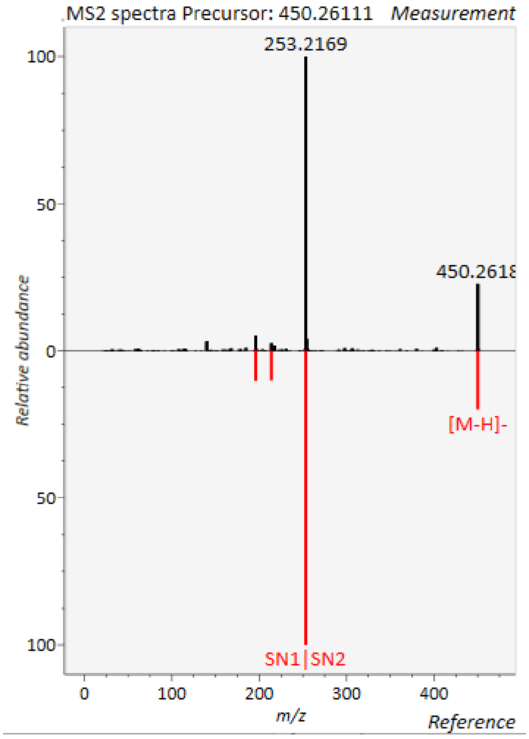

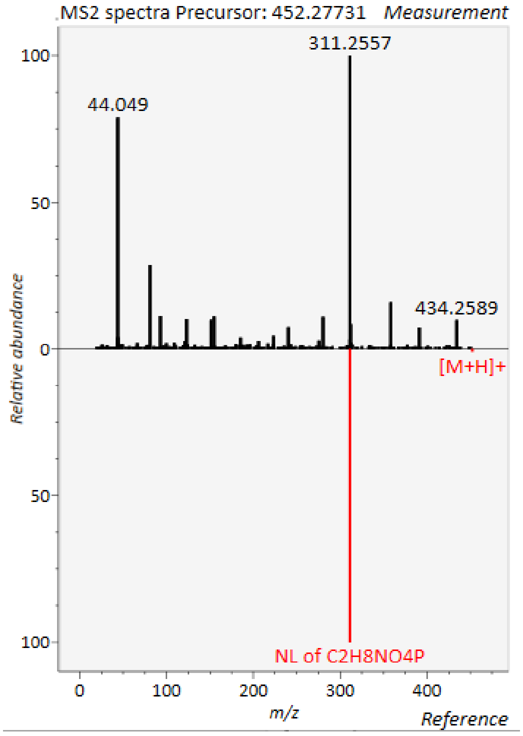

LPE 18:0 (2), left: RP neg, m/z 480.3087 (M-H)-, MW 481.3152, RT 10.50 min, 20V

right: RP pos, m/z 482.3263 (M+H)+, RT 10.49 min, 20V, isomers possible

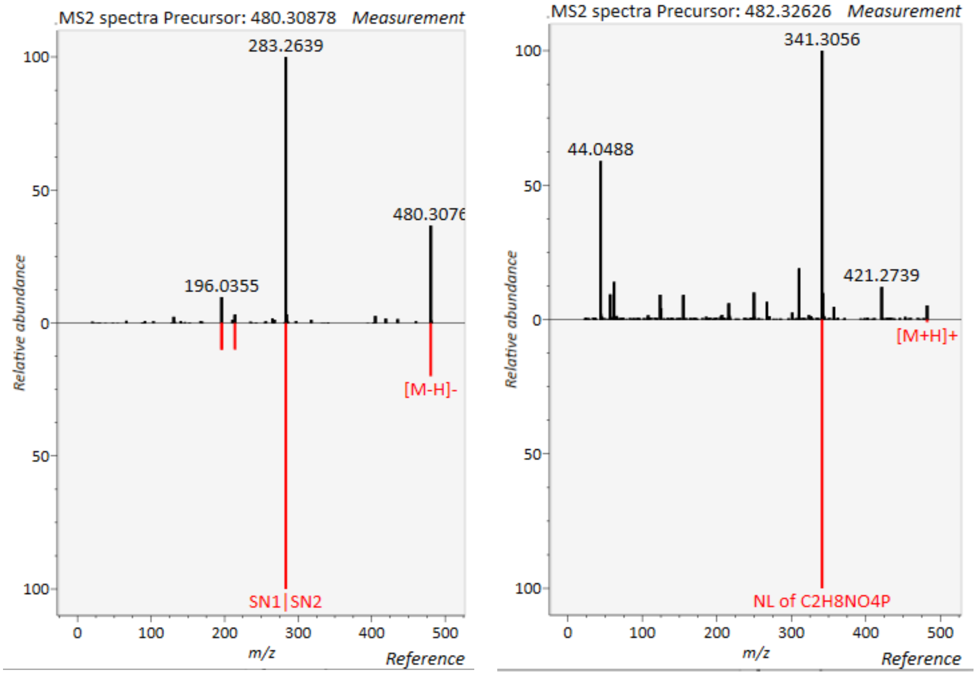

LPE 18:1 (2), left: RP neg, m/z 478.2933 (M-H)-, MW 479.2999, RT 10.23 min, 20V

right: RP pos, m/z 480.3106 (M+H)+, RT 10.22 min, 20V, isomers possible

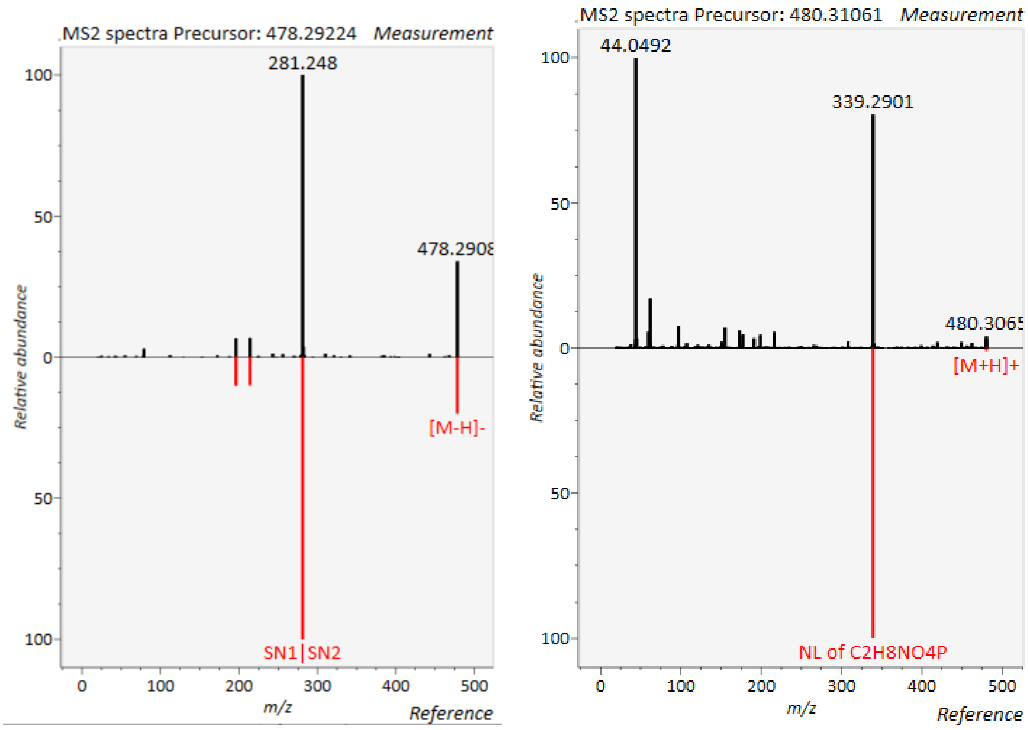

LPE 18:2 (2), left: RP neg, m/z 476.2777 (M-H)-, MW 477.2843, RT 9.97 min, 20V

right: RP pos, m/z 478.2941 (M+H)+, RT 9.94 min, 10V, isomers possible

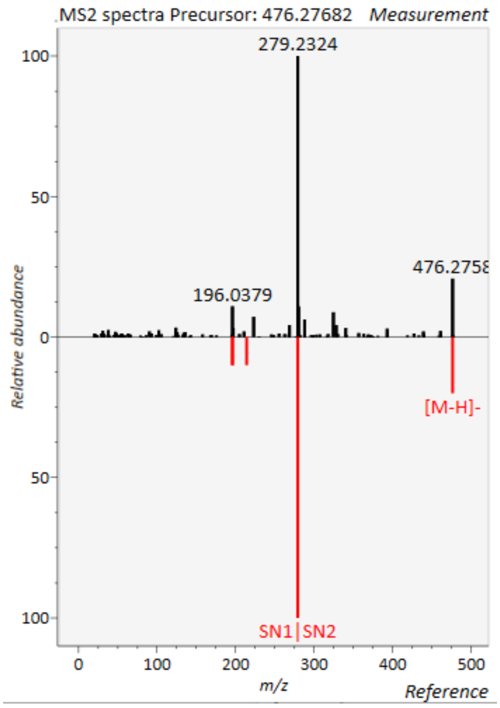

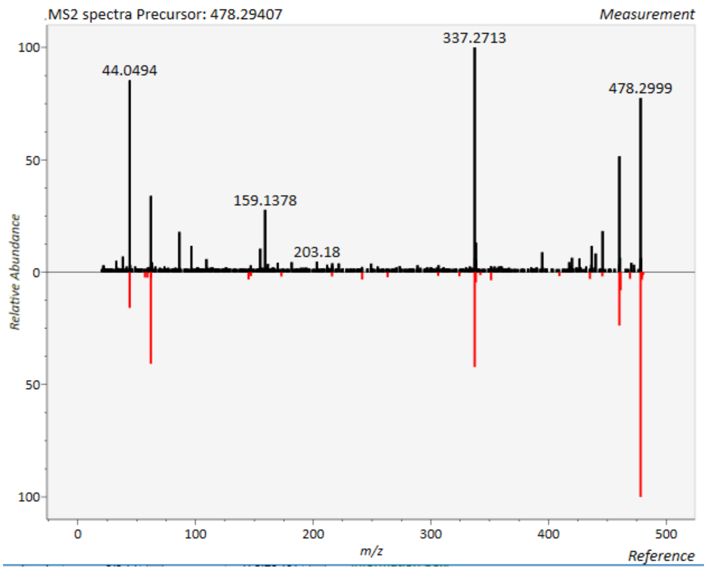

LPE 20:4 (2), left: RP neg, m/z 500.2775 (M-H)-, MW 501.2843, RT 9.96 min, 20V

right: RP pos, m/z 502.2936 (M+H)+, RT 9.93 min, 20V, isomers possible

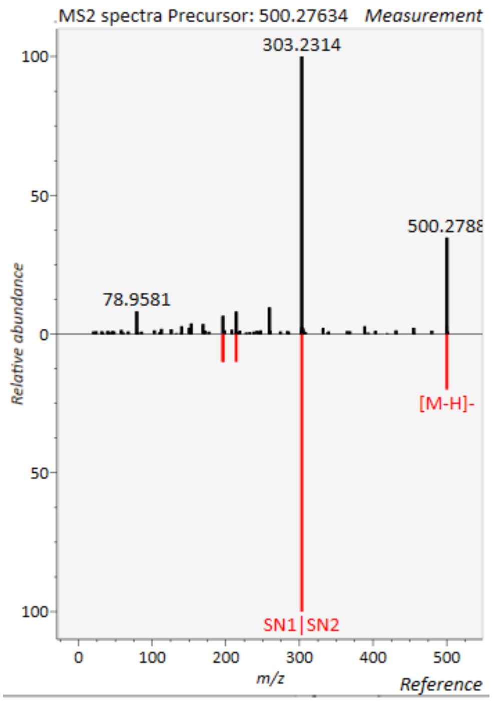

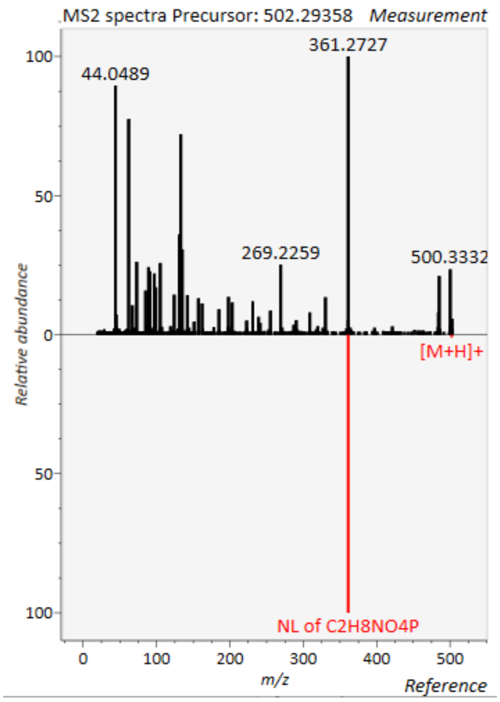

panthenol (2), RP pos, m/z 206.1391 (M+H)+, MW 205.1313, RT 2.33 min, 20V, HMDB04231

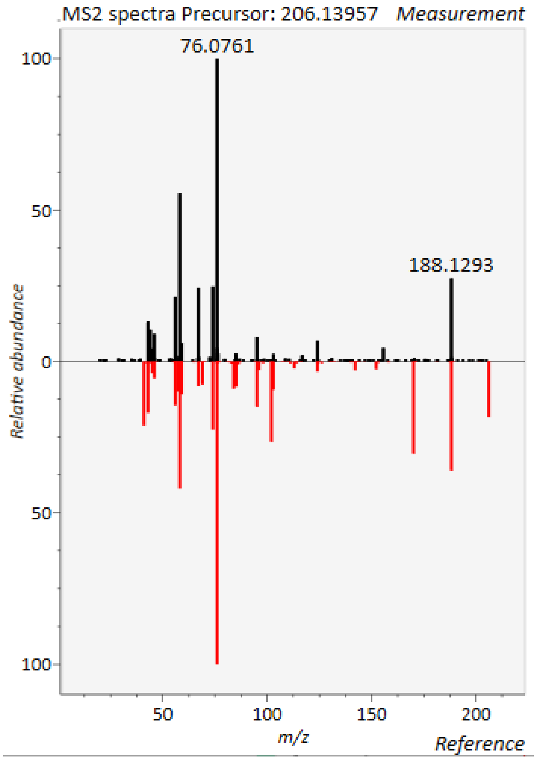

PC 32:1 (16:0_16:1) (2), left: RP neg, m/z 776.5432 (M+FA-H)-, MW 731.5465, RT 12.17 min, 20V

right: RP pos, m/z 732.5543 (M+H)+, RT 12.09 min, 10V, isomers possible

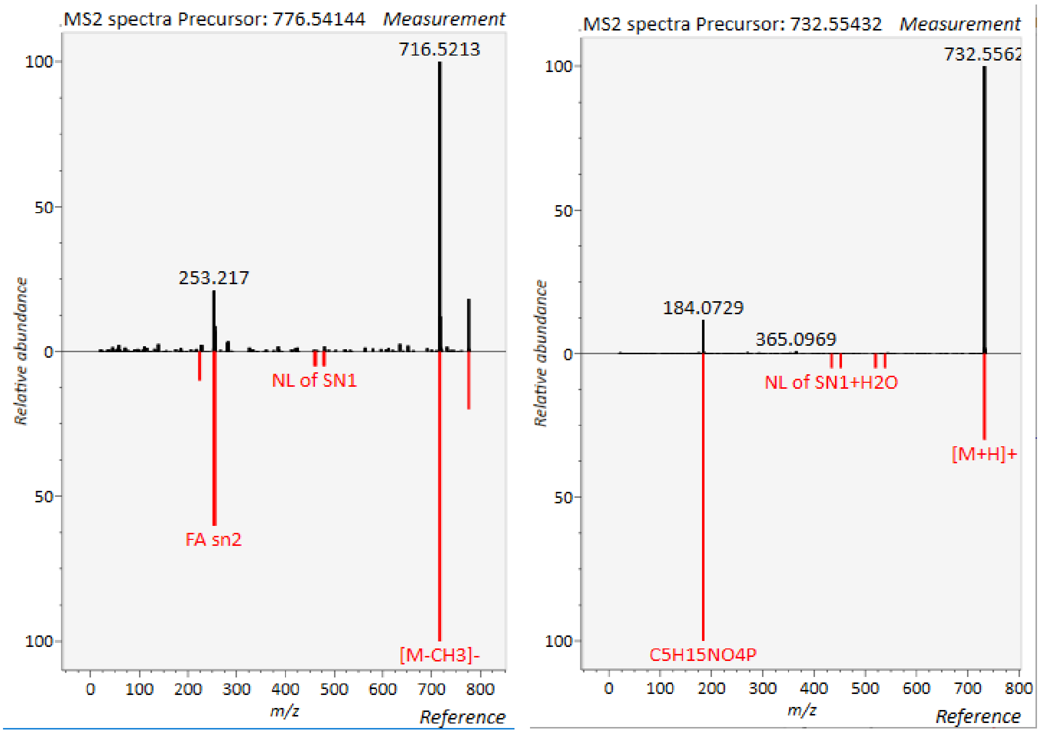

PC 32:1e (16:0e_16:1) (2), left: RP pos, m/z 718.5747 (M+H)+, MW 717.5667, RT 12.56 min, 10V

right: RP neg, m/z 762.5639 (M+FA-H)-, RT 12.63 min, 20V, isomers possible

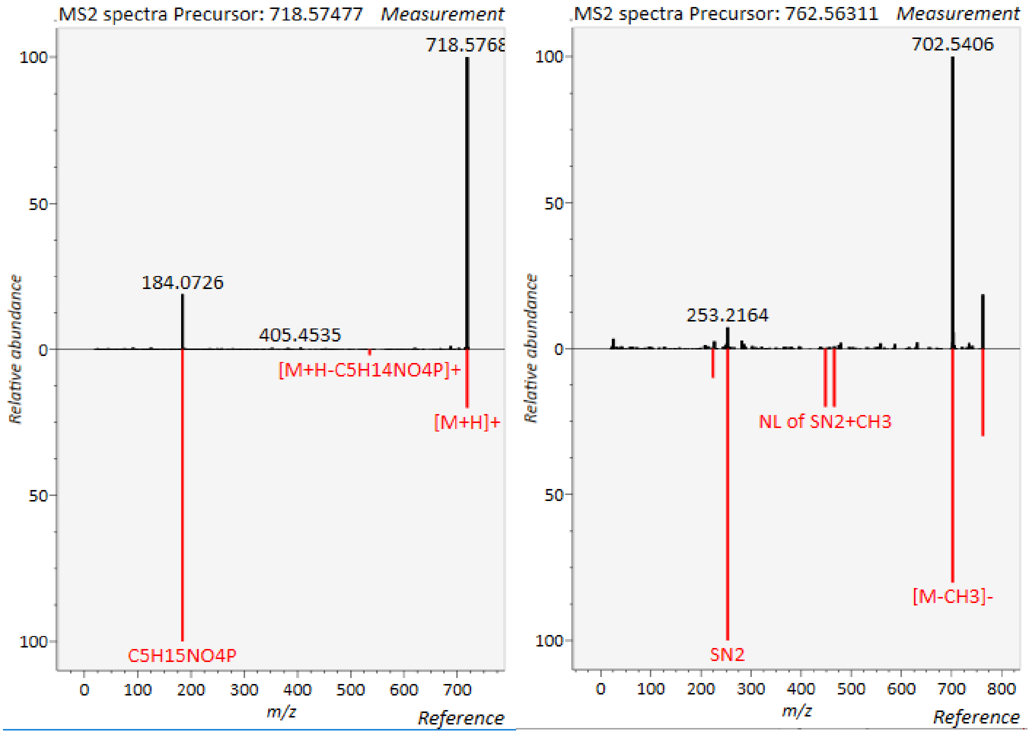

PC 34:1 (16:0_18:1) (2), left: RP neg, m/z 804.575 (M+FA-H)-, MW 759.5789, RT 12.76 min, 20V

right: RP pos, m/z 760.5867 (M+H)+, RT 12.67 min, 20V, isomers possible

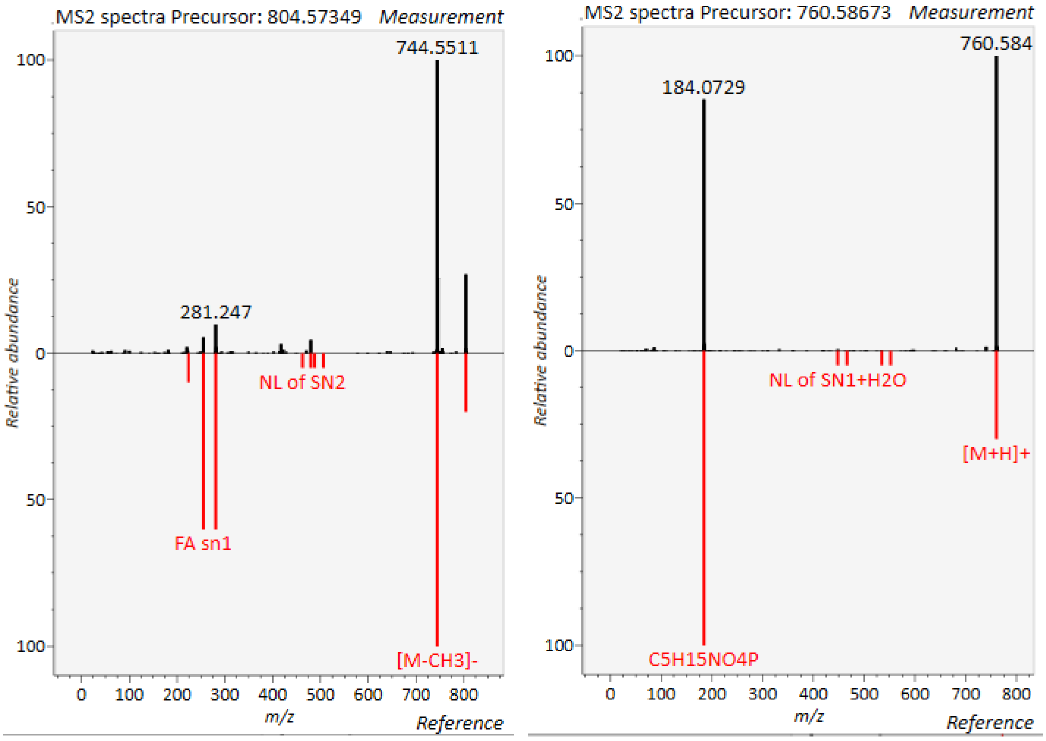

PC 34:2 (16:0_18:2) (1), left: RP neg, m/z 802.559 (M+FA-H)-, MW 757.5637, RT 12.31 min, 20V

right: RP pos, m/z 758.5715 (M+H)+, RT 12.24 min, 20V, HMDB07973

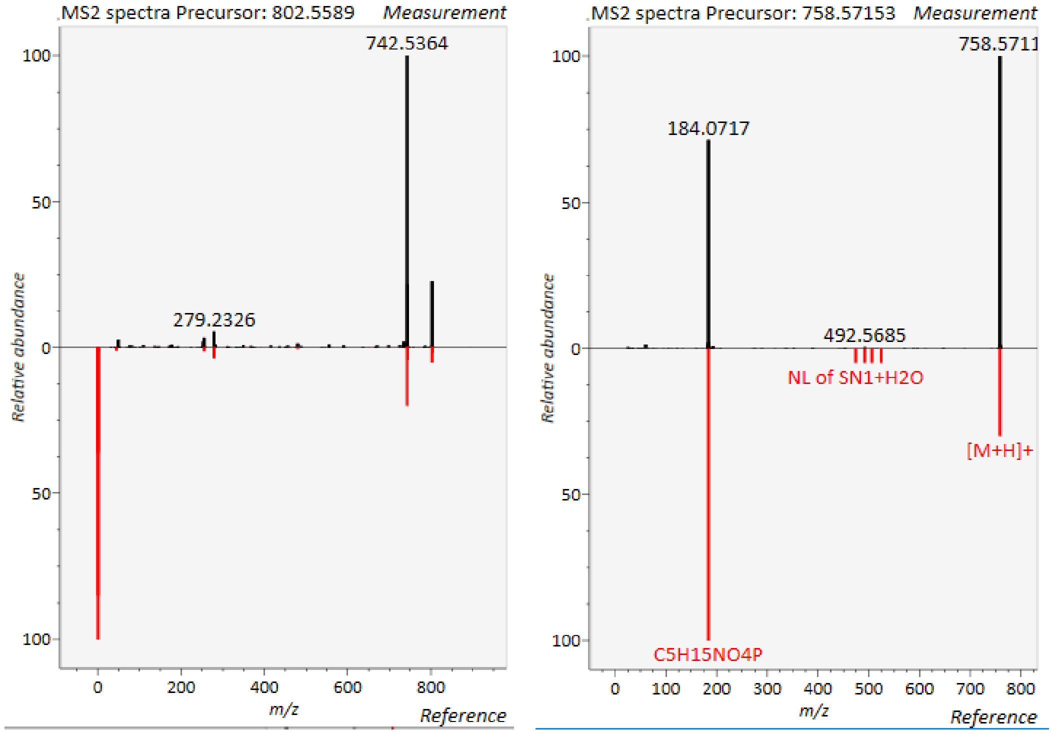

PC 34:2e (16:0e_18:2) (2), RP neg, m/z 788.5795 (M-H)-, MW 789.5849, RT 12.75 min, 20V, isomers possible

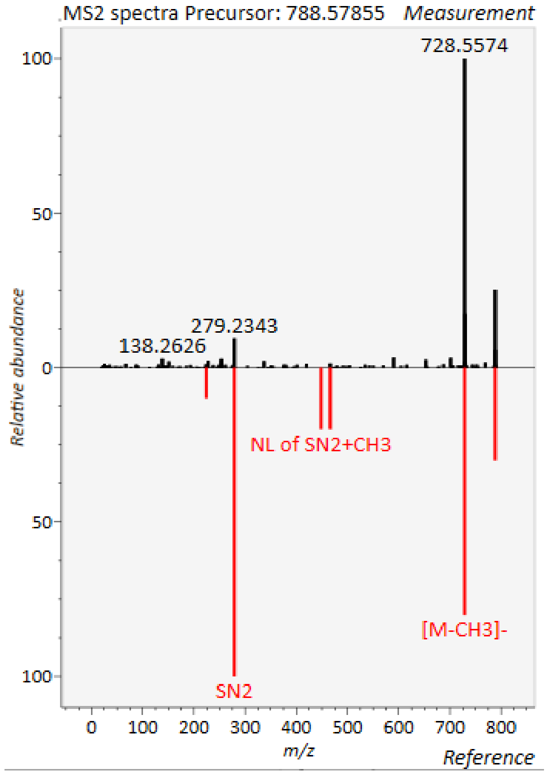

PC 34:3 (16:1_18:2) (2), RP neg, m/z 800.5435 (M-H)-, MW 801.5504, RT 11.93 min, 20V, isomers possible

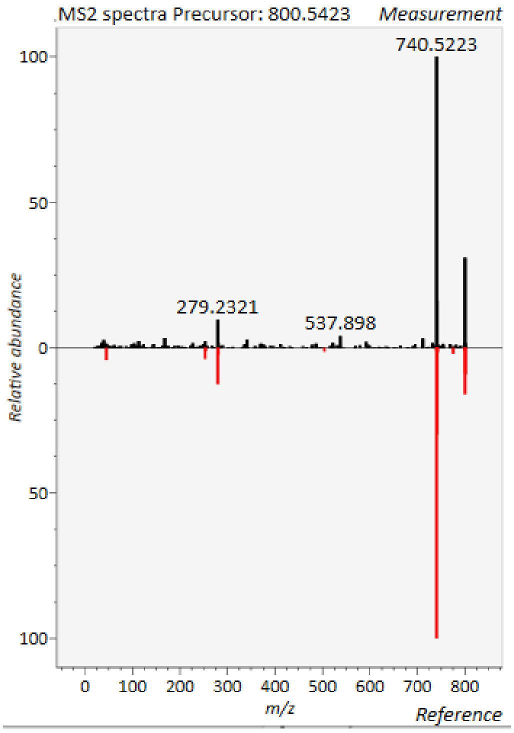

PC 36:2 (18:1_18:1) (2), left: RP neg, m/z 830.5904 (M+FA-H)-, MW 785.5949, RT 13.01 min, 20V

right: RP pos, m/z 786.6027 (M+H)+, RT 12.88 min, 20V, isomers possible

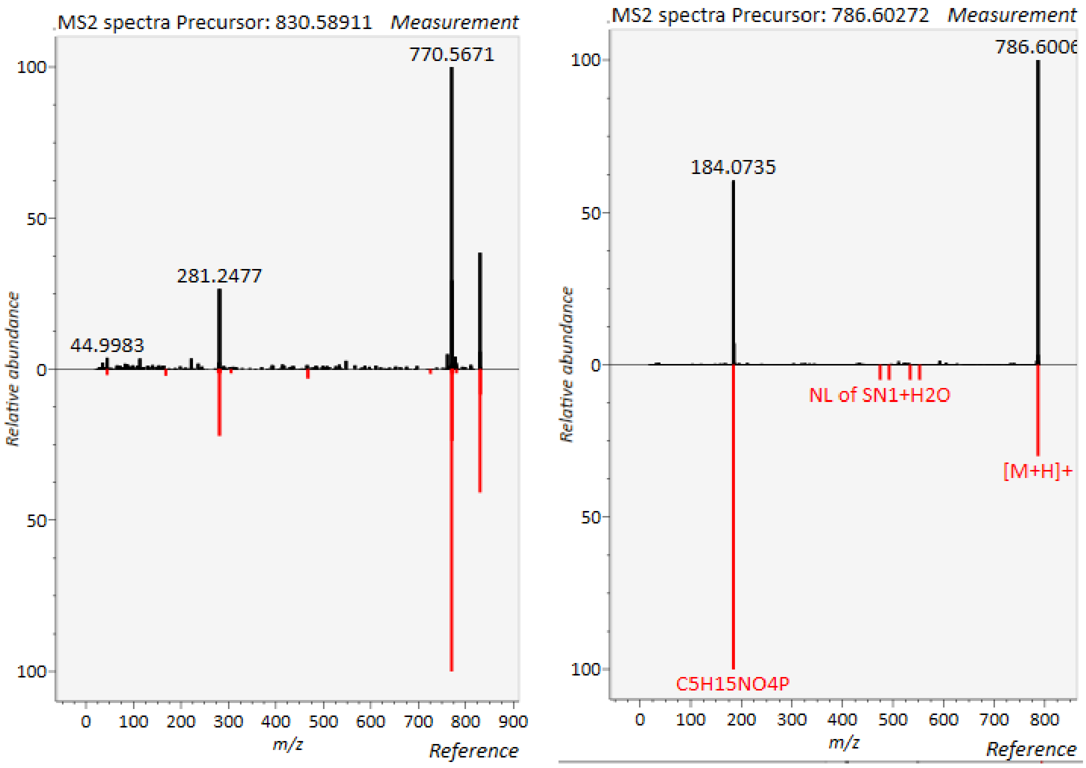

PC 36:3 (18:1_18:2) (2), left: RP pos, m/z 784.5874 (M+H)+, MW 783.5794, RT 12.33 min, 20V

right: RP neg, m/z 828.5748 (M+FA-H)-, RT 12.42 min, 20V, isomers possible

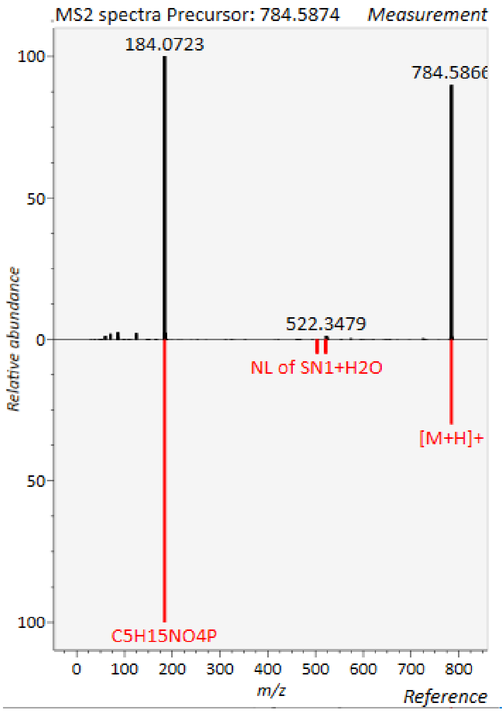

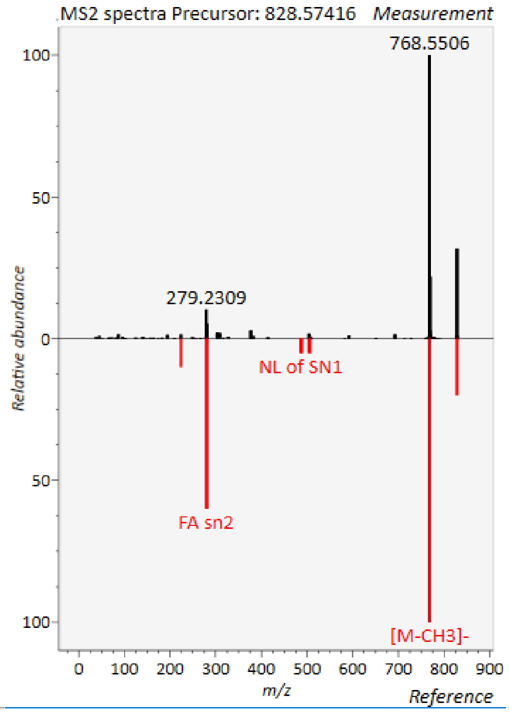

PC 36:4 (2), left: RP pos, m/z 782.5716 (M+H)+, MW 781.5647, RT 12.08 min, 20V

right: RP neg, m/z 826.559 (M+FA-H)-, RT 12.22, 20V, isomers possible

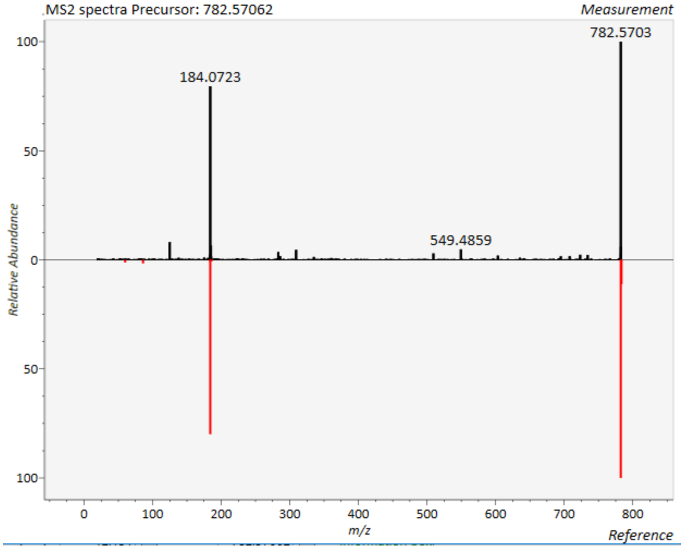

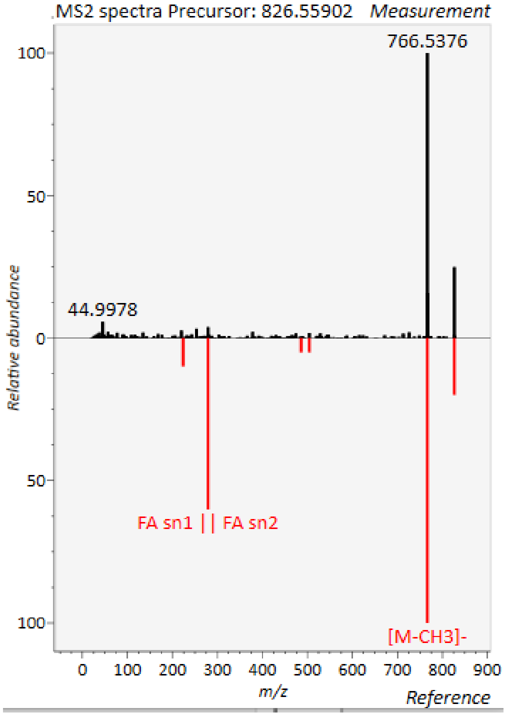

PC 38:4 (18:0_20:4) (2), left: RP pos, m/z 810.6027 (M+H)+, MW 809.5964, RT 12.78 min, 20V

right: RP neg, m/z 854.5901 (M+FA-H)-, RT 12.87, 40V, isomers possible

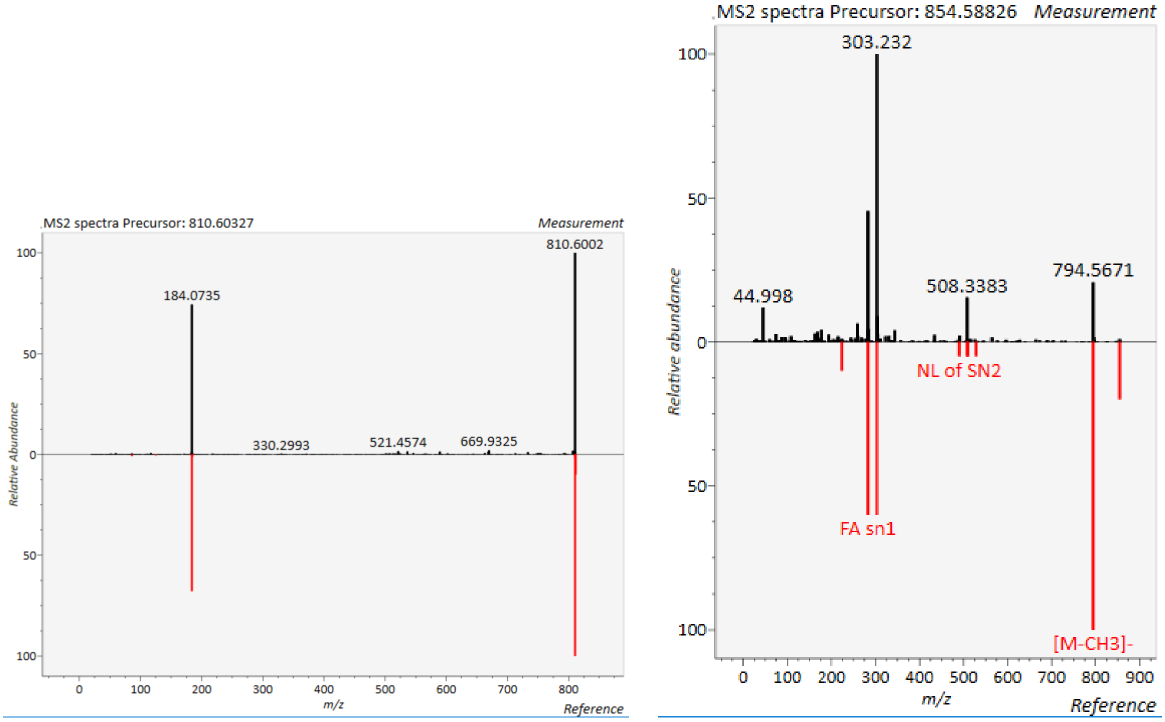

PC 38:5 (18:1_20:4) (2), left: RP pos, m/z 808.5853 (M+H)+, MW 807.5807, RT 12.28, 20V

right: RP neg, m/z 852.5746 (M+FA-H)-, RT 12.33 min, 20V, isomers possible

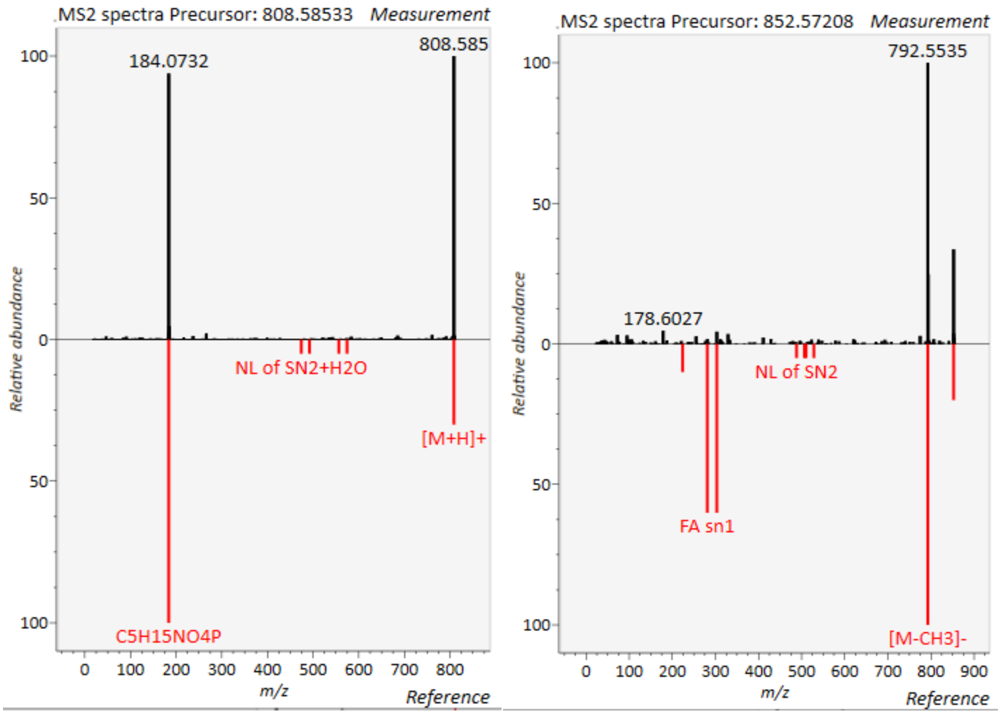

PC 38:5e (18:1e_20:4) (2), left: RP pos, m/z 794.6085 (M+H)+, MW 793.5995, RT 12.67 min, 10V

right: RP neg, m/z 838.5952 (M+FA-H)-, RT 12.76 min, 20V, isomers possible

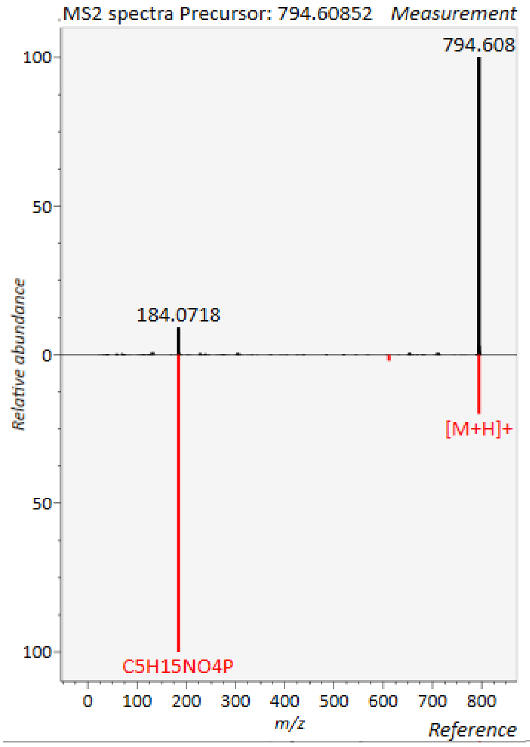

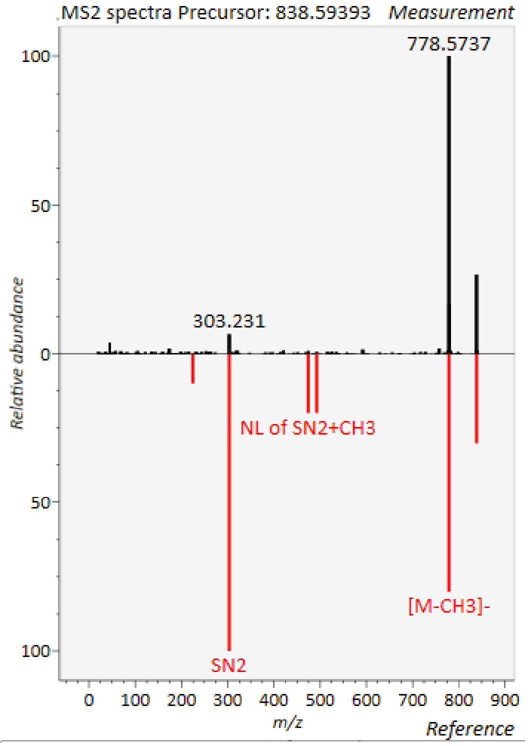

PE 28:0 (13:0_15:0) (2), RP neg, m/z 634.4441 (M-H)-, MW 635.451, RT 11.38 min, 20V, isomers possible

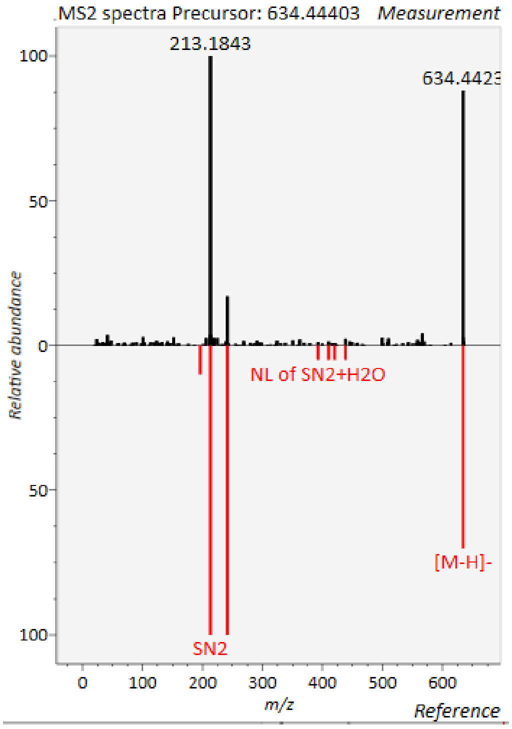

PE 30:0 (15:0_15:0) (2), left: RP neg, m/z 662.4753 (M-H)-, MW 663.4817, RT 11.78 min, 20V

right: RP pos, m/z 664.4899 (M+H)+, RT 11.75 min, 20V, isomers possible

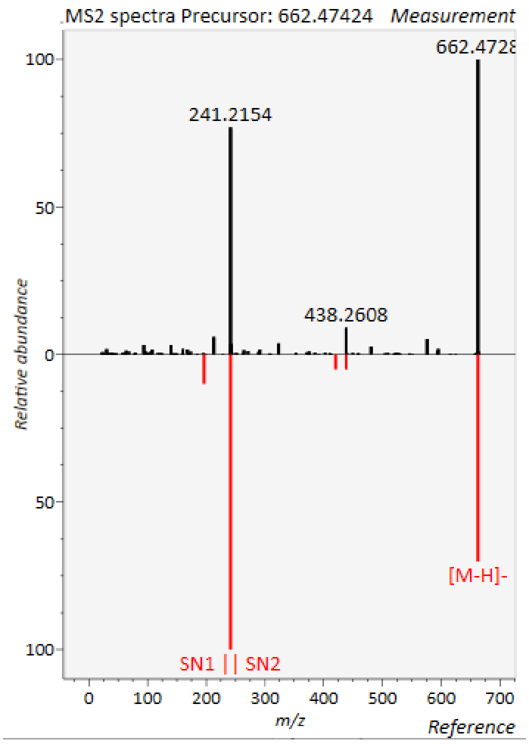

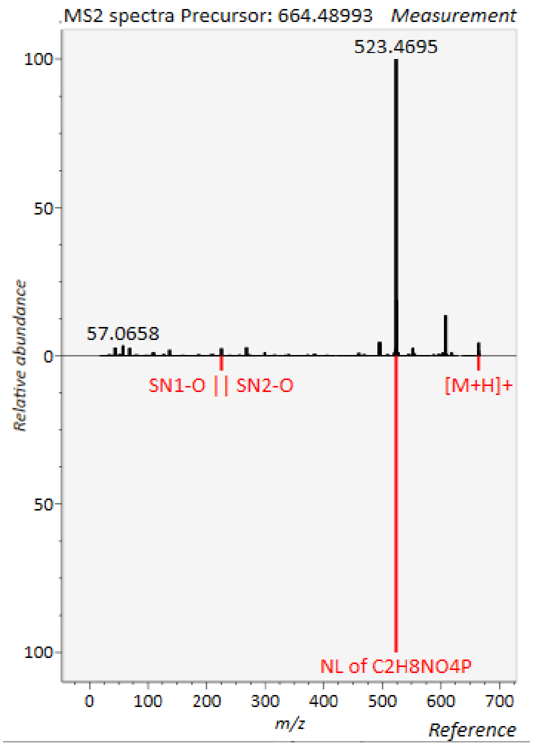

PE 32:1 (15:0-17:1) (2), left: RP neg, m/z 688.4916 (M-H)-, MW 689.4977, RT 12.05 min, 40V

right: RP pos, m/z 690.5084 (M+H)+, RT 11.99 min, 10V, isomers possible

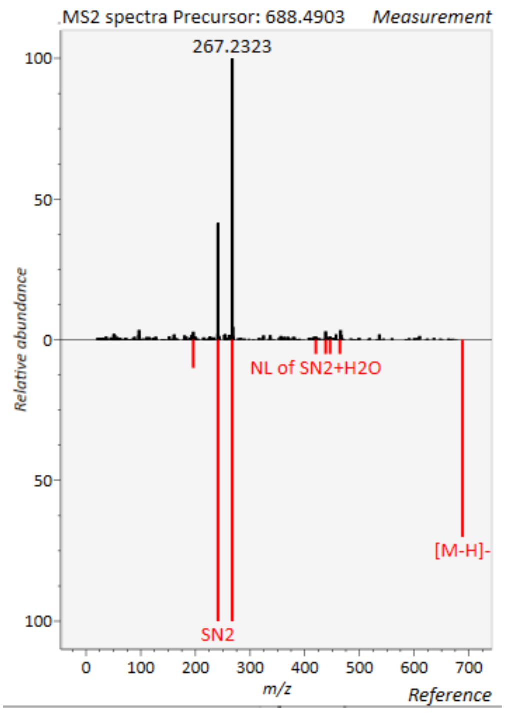

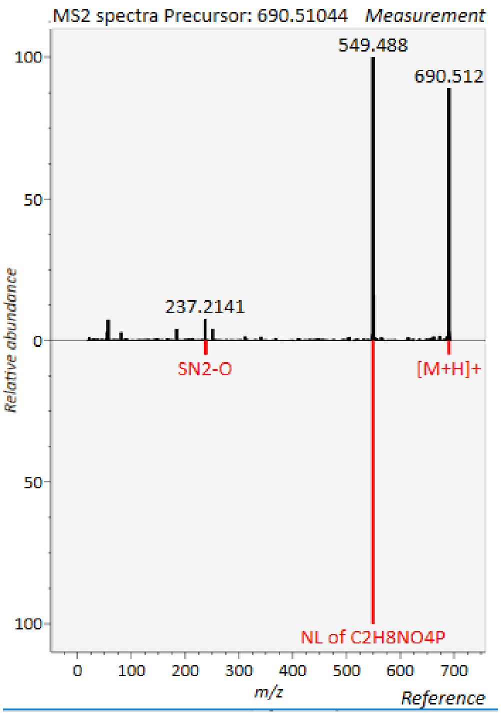

PE 32:2 (16:1_16:1) (2), RP neg, m/z 686.4751 (M-H)-, MW 687.4828, RT 11.70 min, 20V, isomers possible

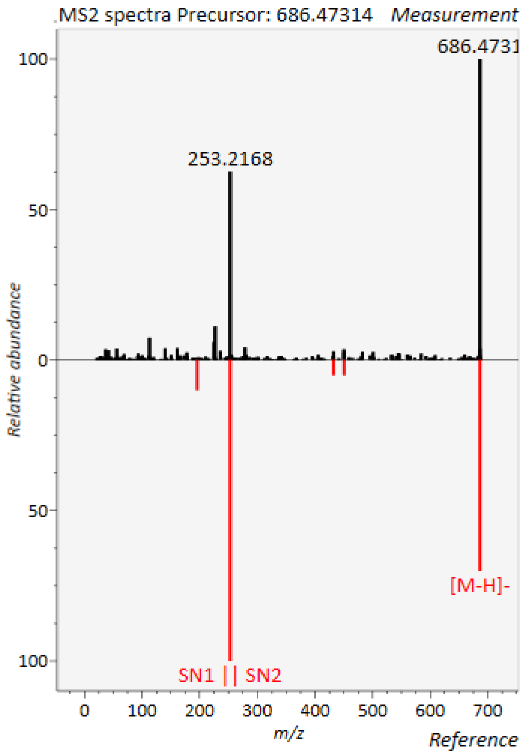

PE 33:2 (15:0_18:2) (2), RP neg, m/z 700.4909 (M-H)-, MW 701.4984, RT 11.88 min, 20V, isomers possible

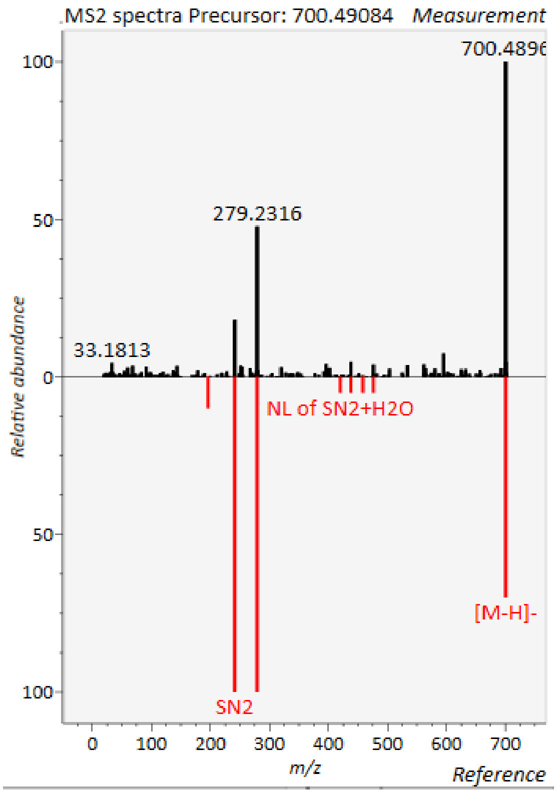

PE 34:2 (16:1_18:1) (2), left: RP pos, m/z 716.5237 (M+H)+, MW 715.5208, RT 12.13 min, 20V

right: RP neg, m/z 714.5052 (M-H)-, RT 12.17 min, 20V, isomers possible

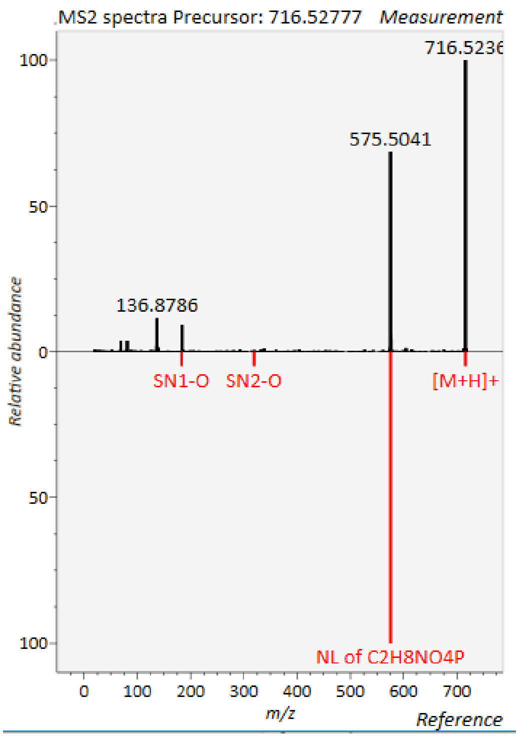

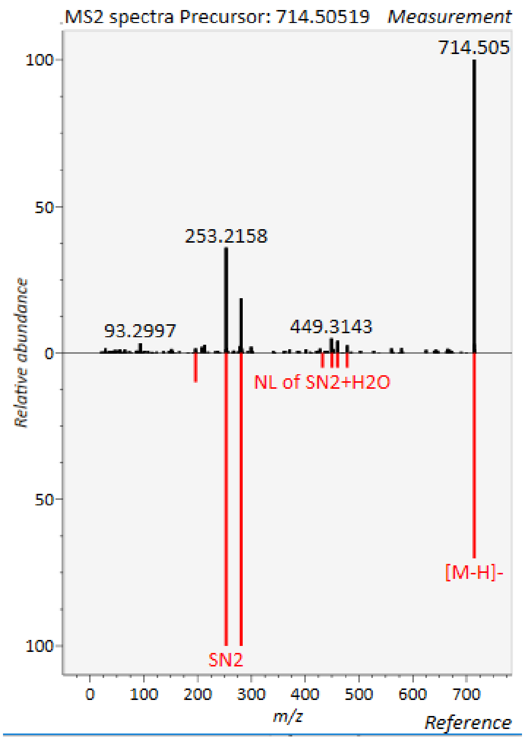

PE 36:2 (18:1_18:1) (2), RP neg, m/z 742.5369 (M-H)-, MW 743.5448, RT 12.79 min, 20V, isomers possible

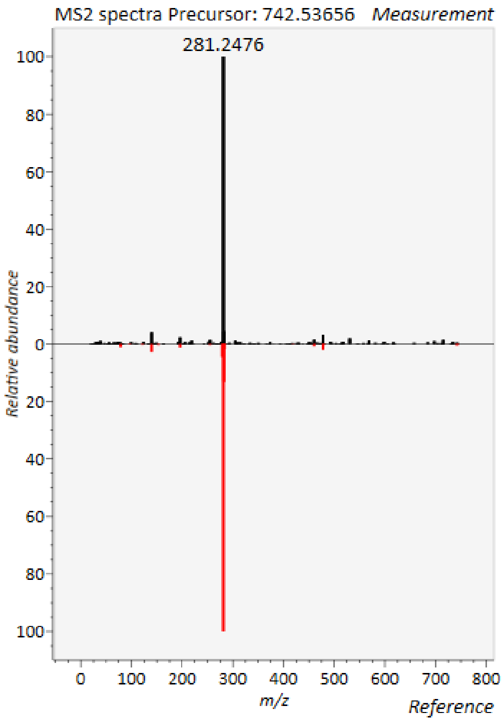

PE 36:2e (18:1e_18:1) (2), RP neg, m/z 728.5583 (M-H)-, MW 729.5655, RT 13.92 min, 20V, isomers possible

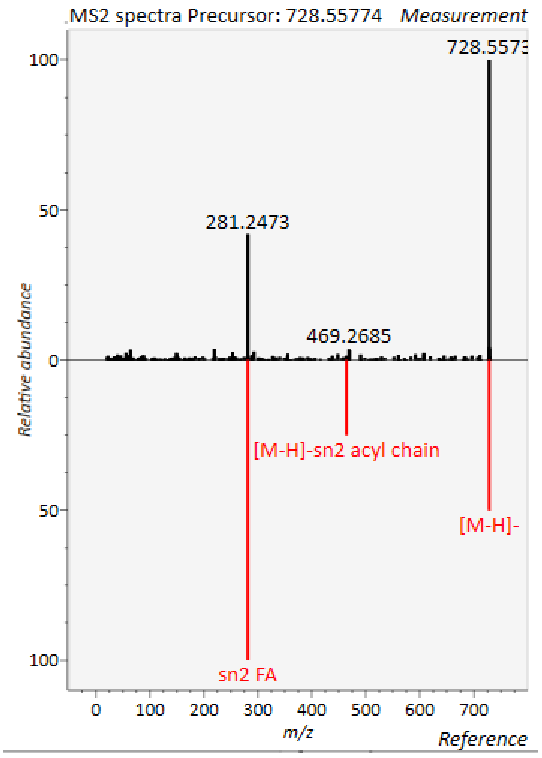

PE 36:3 (18:1_18:2) (2), left: RP neg, m/z 740.5215 (M-H)-, MW 741.5282, RT 12.31 min, 40V

right: RP pos, m/z 742.5424 (M+H)+, RT 12.24 min, 20V, isomers possible

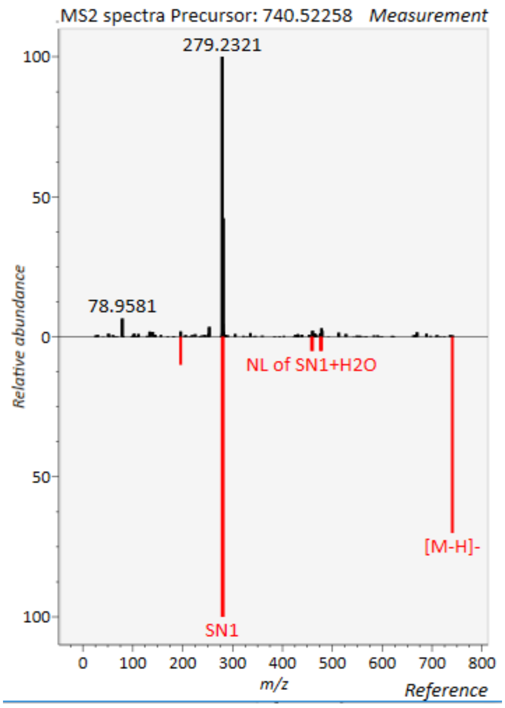

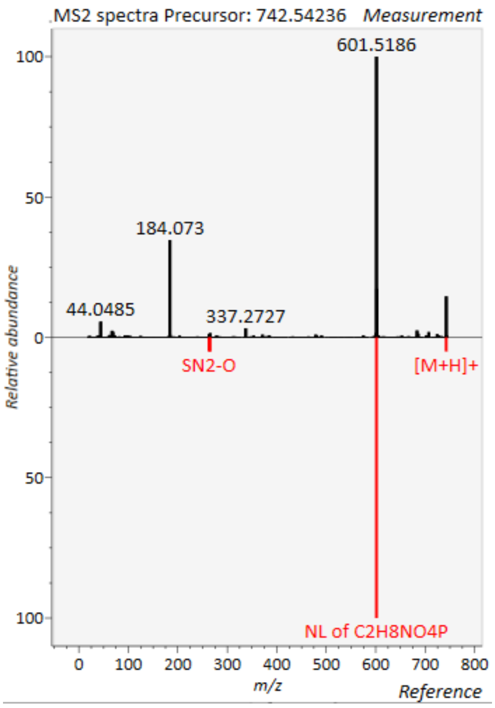

PE 36:3e (18:2e_18:1) (2), left: RP neg, m/z 726.543 (M-H)-, MW 727.5499, RT 13.19 min, 20V

right: RP pos, m/z 728.5594 (M+H)+, RT 13.11 min, 10V, isomers possible

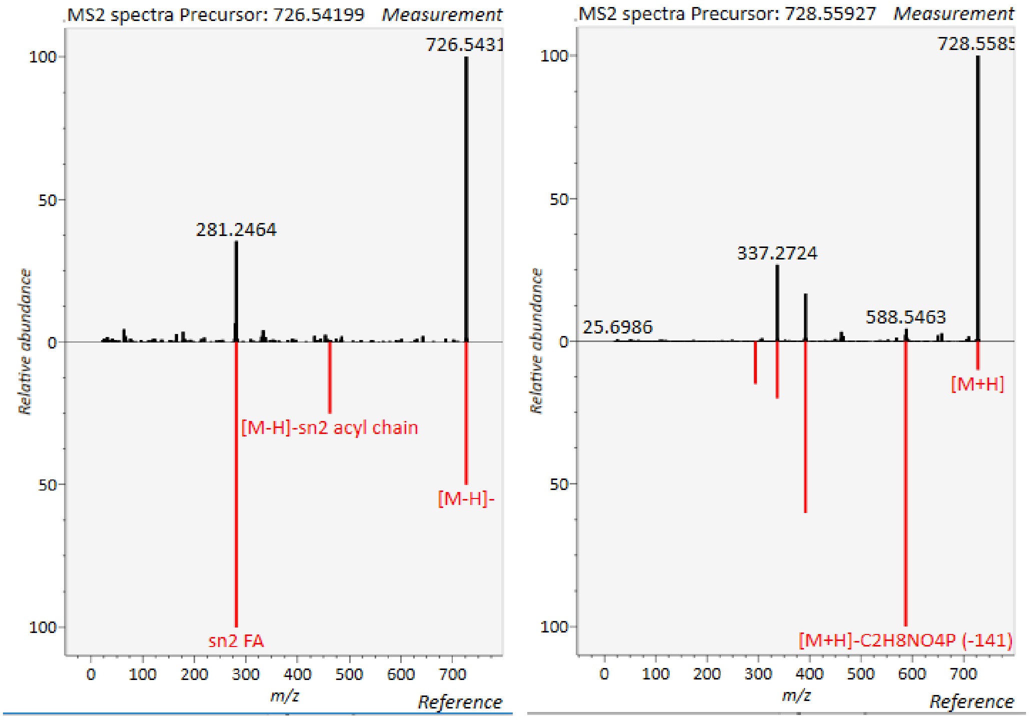

PE 36:4 (16:0_20:4) (2), left: RP neg, m/z 738.5063 (M-H)-, MW 739.5134, RT 12.13 min, 20V

right: RP pos, m/z 740.5243 (M+H)+, RT 12.07 min, 20V, isomers possible

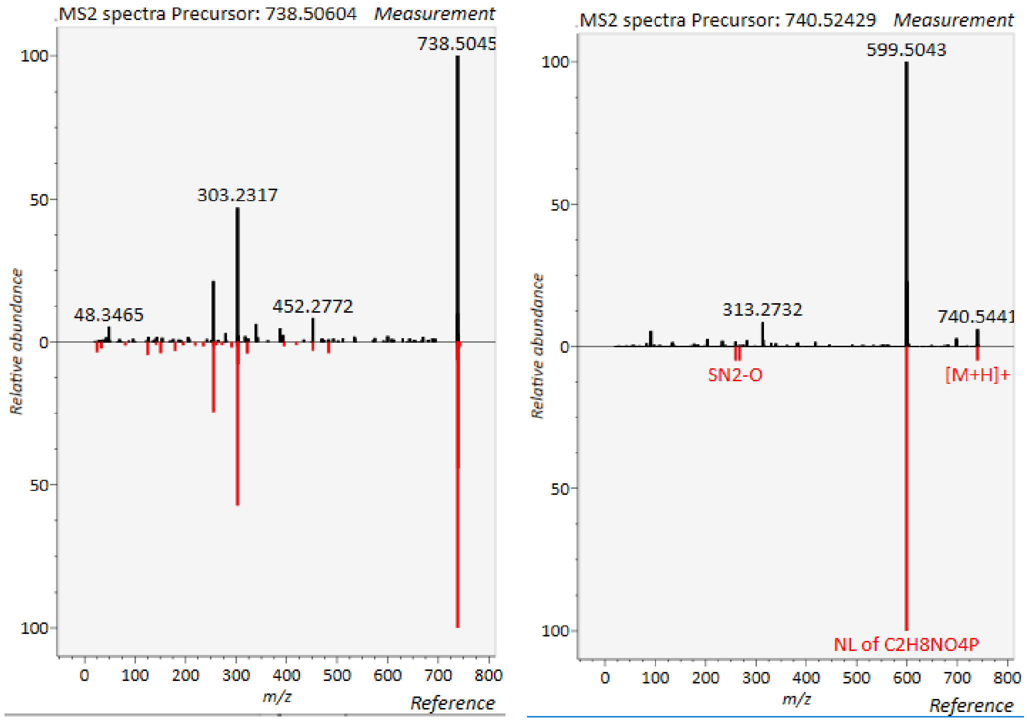

PE 36:4e (16:0e_20:4) (2), left: RP neg, m/z 724.5274 (M-H)-, MW 725.5343, RT 12.59 min, 20V

right: RP pos, m/z 726.5432 (M+H)+, RT 12.50, 20V, isomers possible

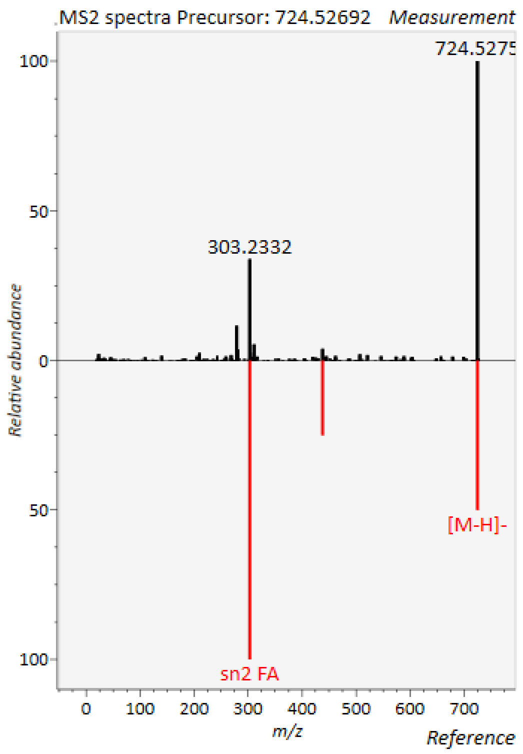

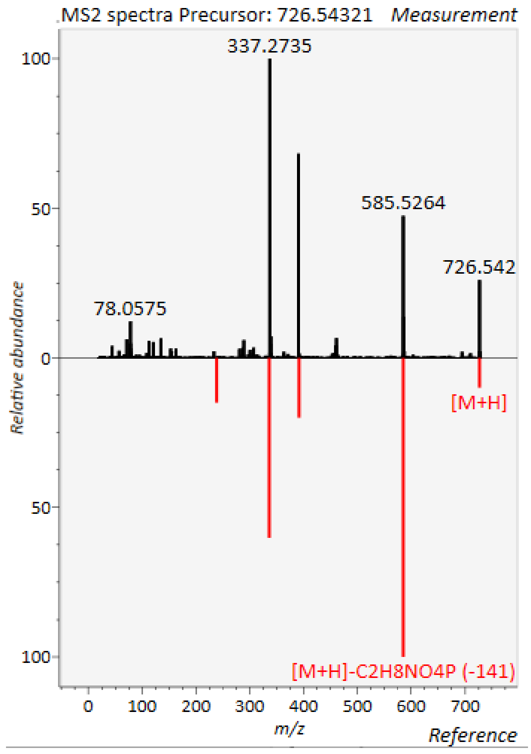

PE 36:5e (16:1e-20:4) (2), RP neg, m/z 722.5116 (M-H)-, MW 723.5187, RT 12.39 min, 20V, isomers possible

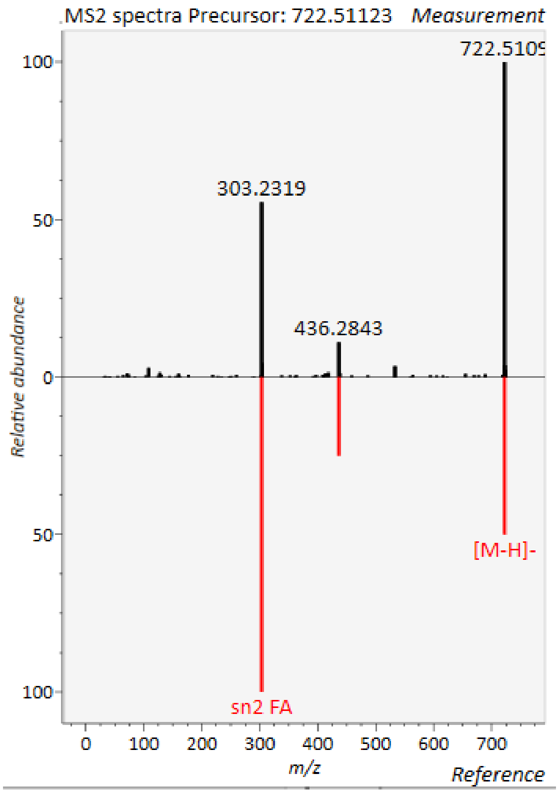

PE 38:4 (18:0_20:4) (2), RP neg, m/z 766.5379 (M-H)-, MW 767.5449, RT 12.76 min, 20V, isomers possible

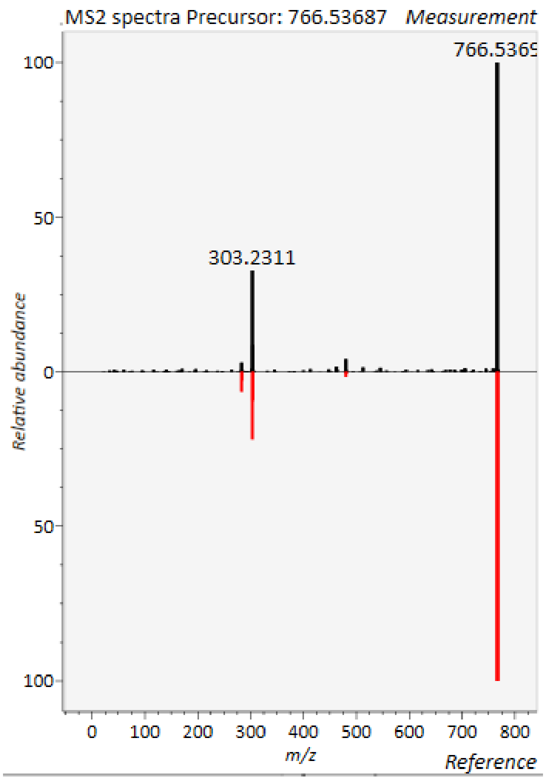

PE 38:4e (18:0e_20:4) (2), left: RP pos, m/z 754.5761 (M+H)+, MW 753.5681, RT 13.26, 20V

right: RP neg, m/z 752.5581 (M-H)-, RT 13.35 min, 20V, isomers possible

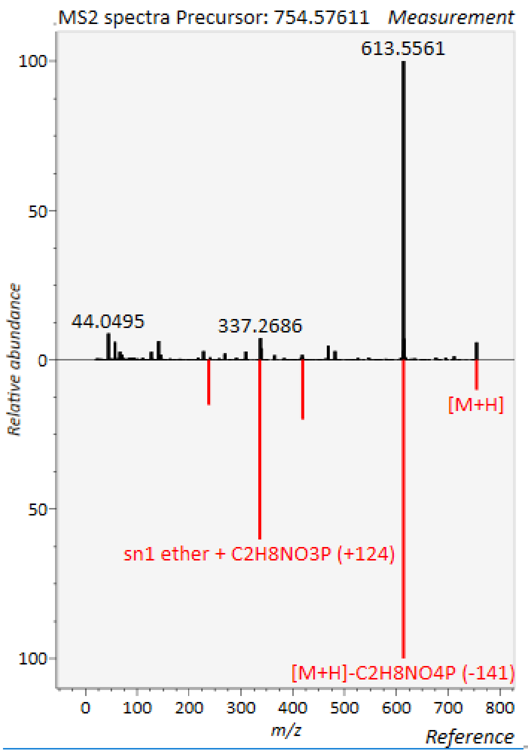

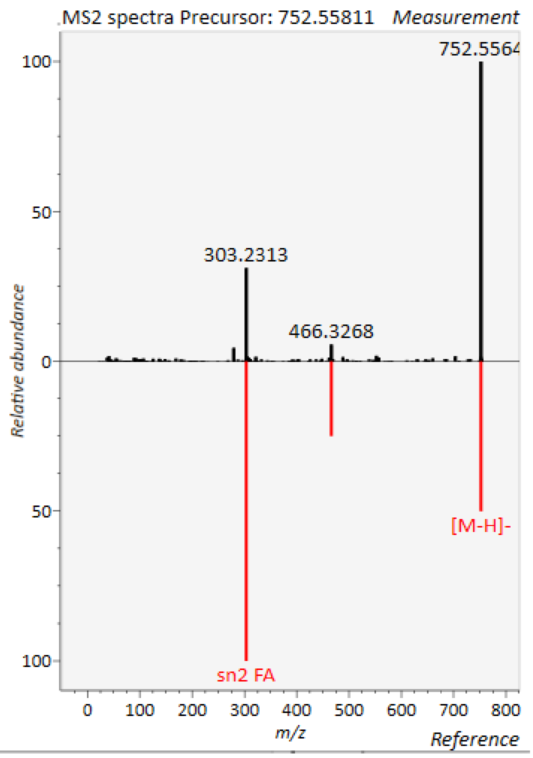

PE 38:5 (18:1_20:4) (2), RP neg, m/z 764.5252 (M-H)-, MW 765.5313, RT 12.26 min, 20V, isomers possible

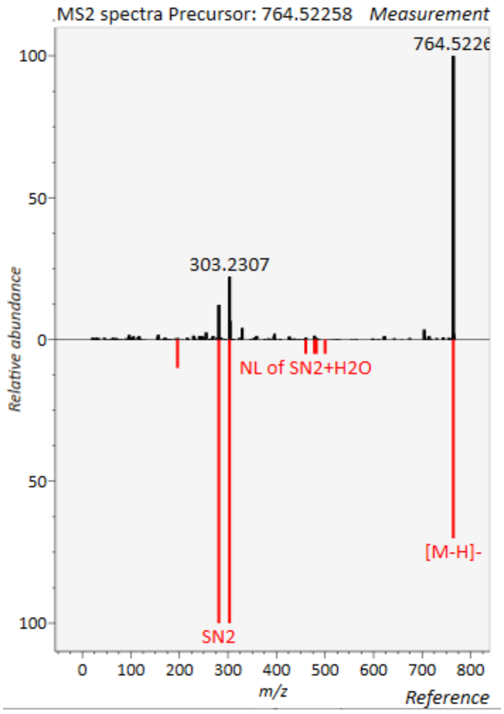

PE 38:5e (18:1e_20:4) (2), left: RP neg, m/z 750.5426 (M-H)-, MW 751.5495, RT 13.11 min, 20V

right: RP pos, m/z 752.5607 (M+H)+, RT 13.00, 10V, isomers possible

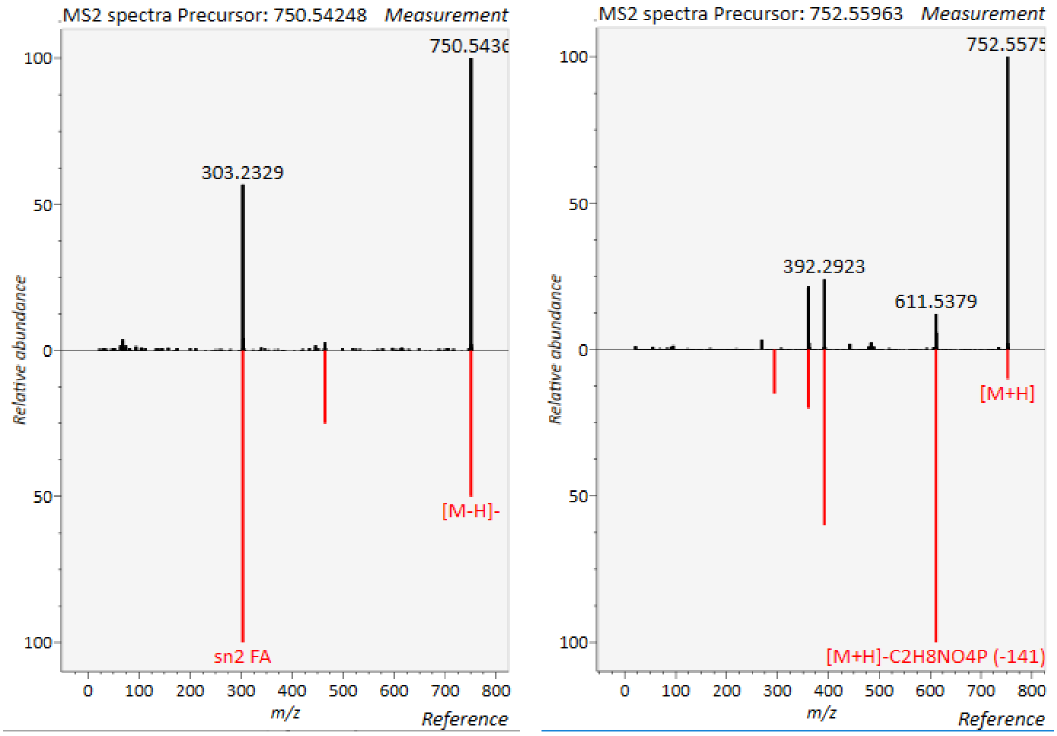

PE 38:6e (18:2e_20:4) (2), left: RP neg, m/z 748.5282 (M-H)-, MW 749.5339, RT 12.52 min, 20V

right: RP pos, m/z 750.5452 (M+H)+, RT 12.43, 20V, isomers possible

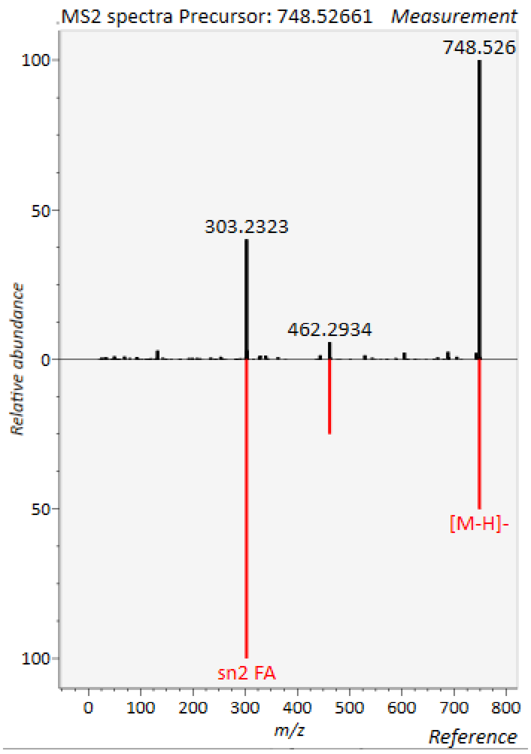

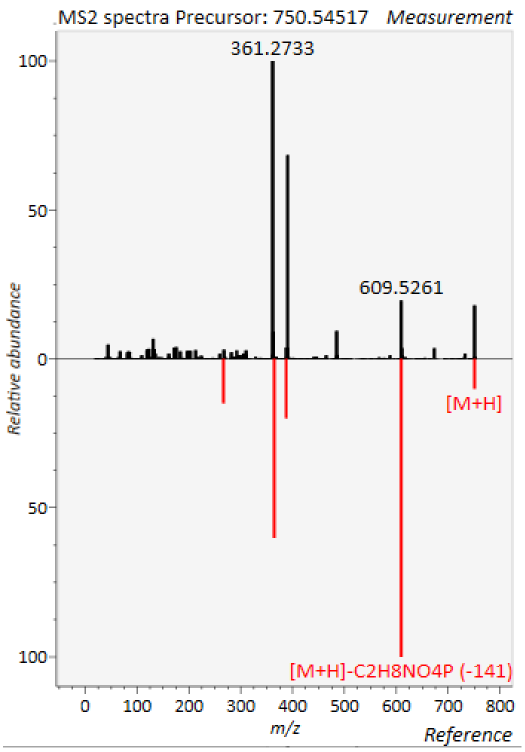

propionylcarnitine (1), hilic pos, m/z 218.1391 (M+H)+, MW 217.1318, RT 2.10 min, 20V, HMDB00824

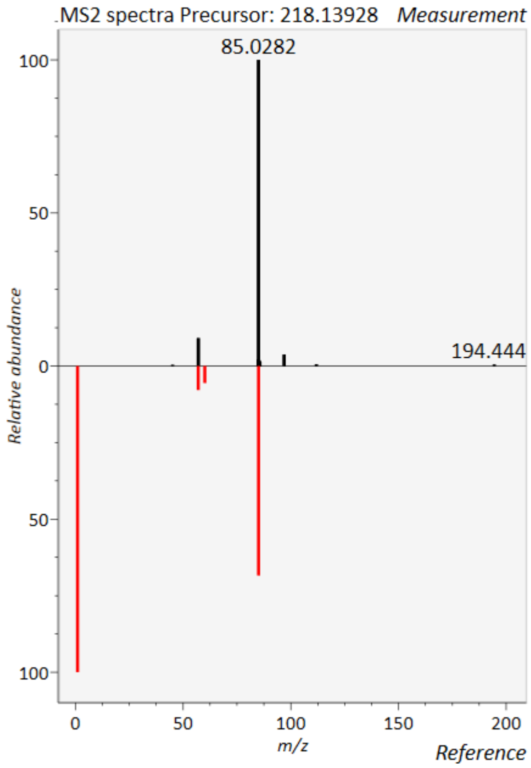

sebacic acid (2), RP neg, m/z 201.1131 (M-H)-, MW 202.1204, RT 6.39 min, 10V, HMDB00792

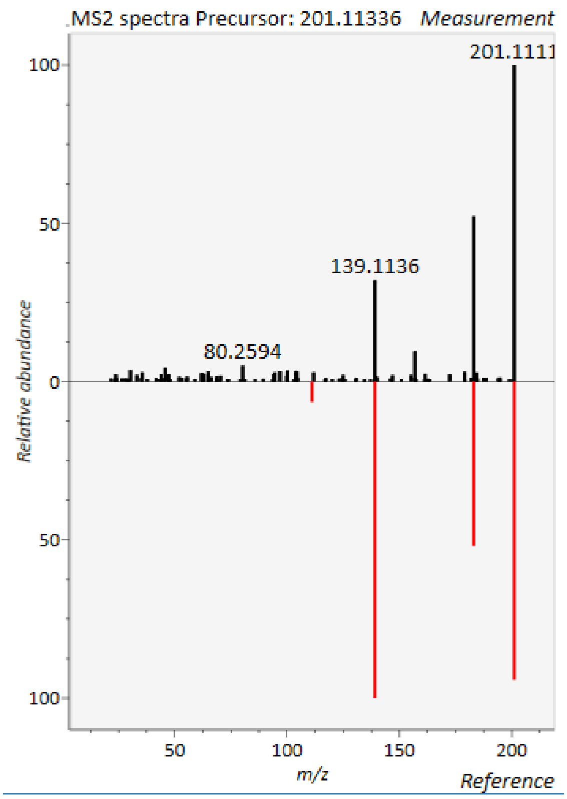

suberic acid (2), RP neg, m/z 173.0816 (M-H)-, MW 174.089, RT 4.64 min, 20V, HMDB00893

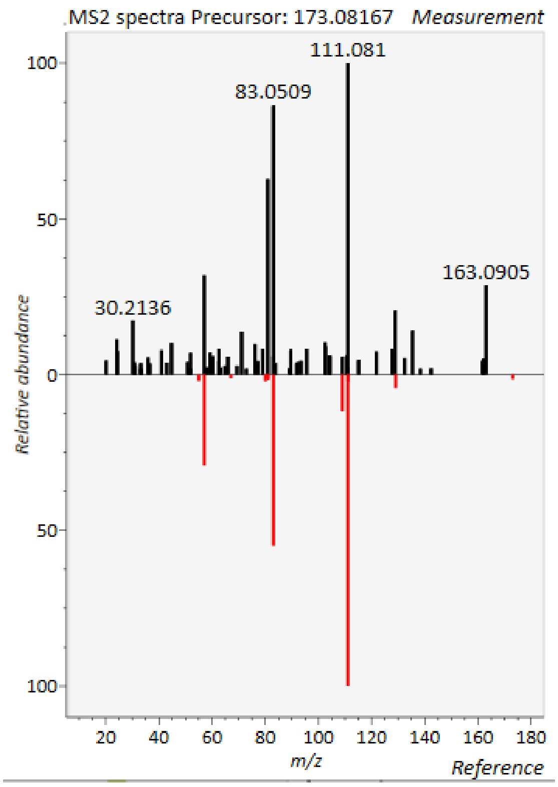

TAG 36:0 (2), RP pos, m/z 656.5841 $(M+NH_4)+$, MW 638.5485, RT 12.34 min, 10V, isomers possible

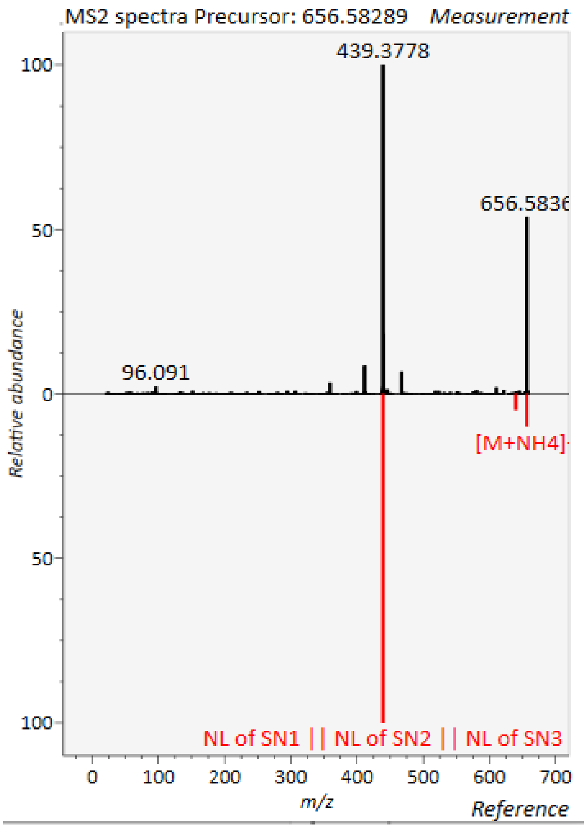

TAG 38:0 (2), RP pos, m/z 684.6152 $(M+NH_4)+$, MW 666.5798, RT 14.98 min, 10V, isomers possible

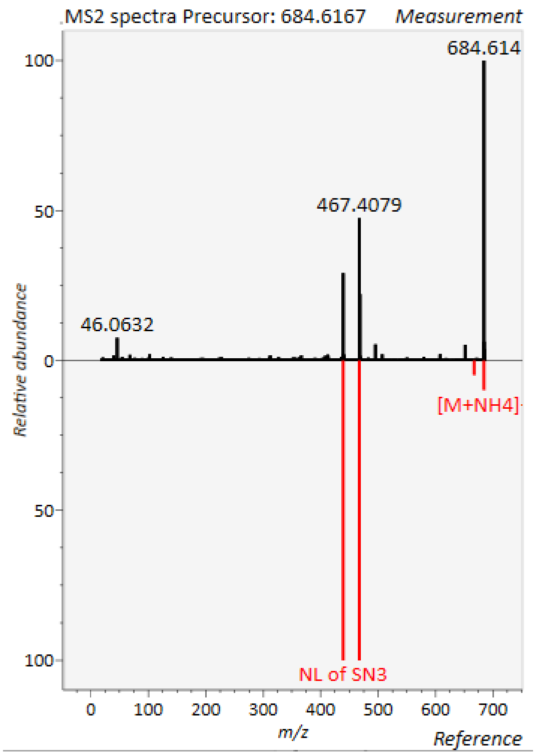

Nucleic acid subunits

2'-deoxy-cytidine (2), hilic pos, m/z 228.0986 (M+H)+, MW 227.0908, RT 2.16 min, 20V, HMDB00014

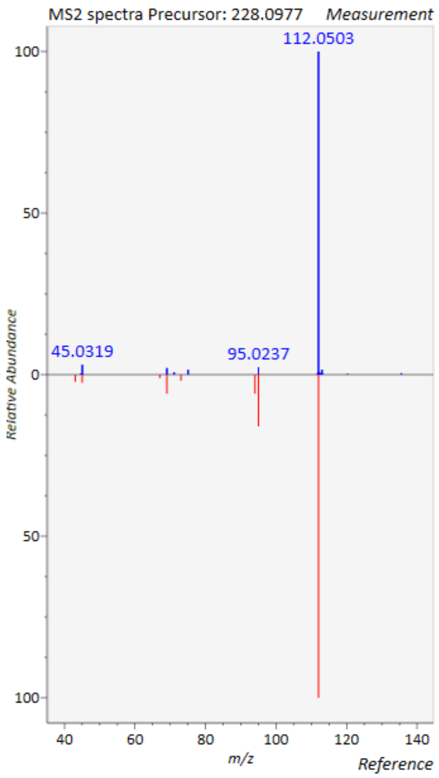

adenine (1), hilic pos, m/z 136.0622 (M+H)+, MW 135.0544, RT 1.16 min, 20V, HMDB00034

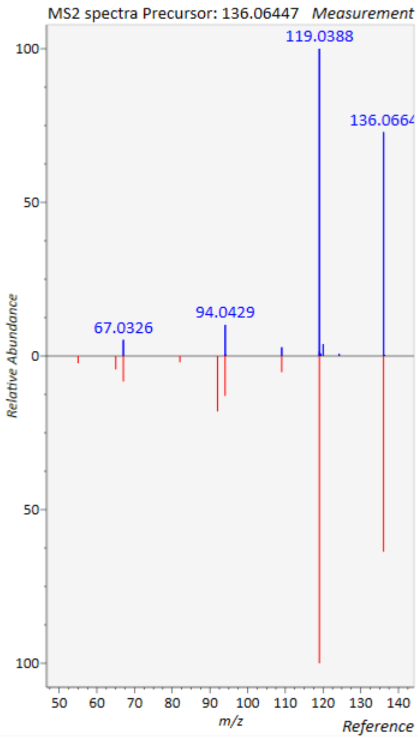

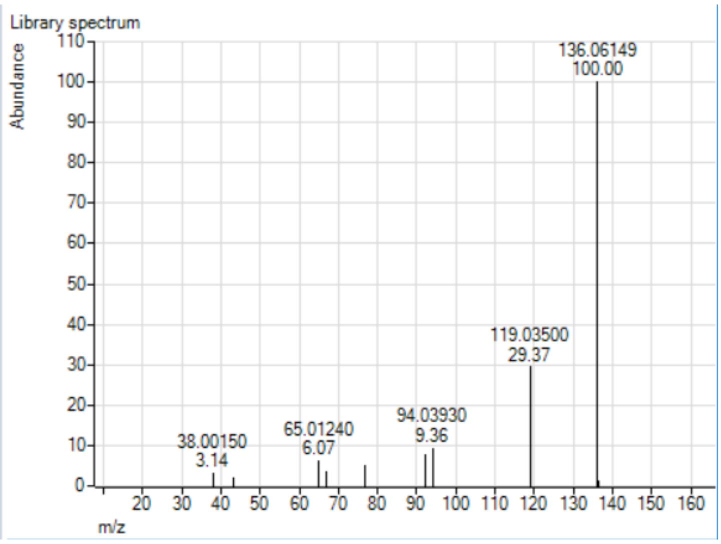

adenosine (1), hilic pos, m/z 268.1045 (M+H)+, MW 267.0977, RT 1.39 min, 20V, HMDB00050

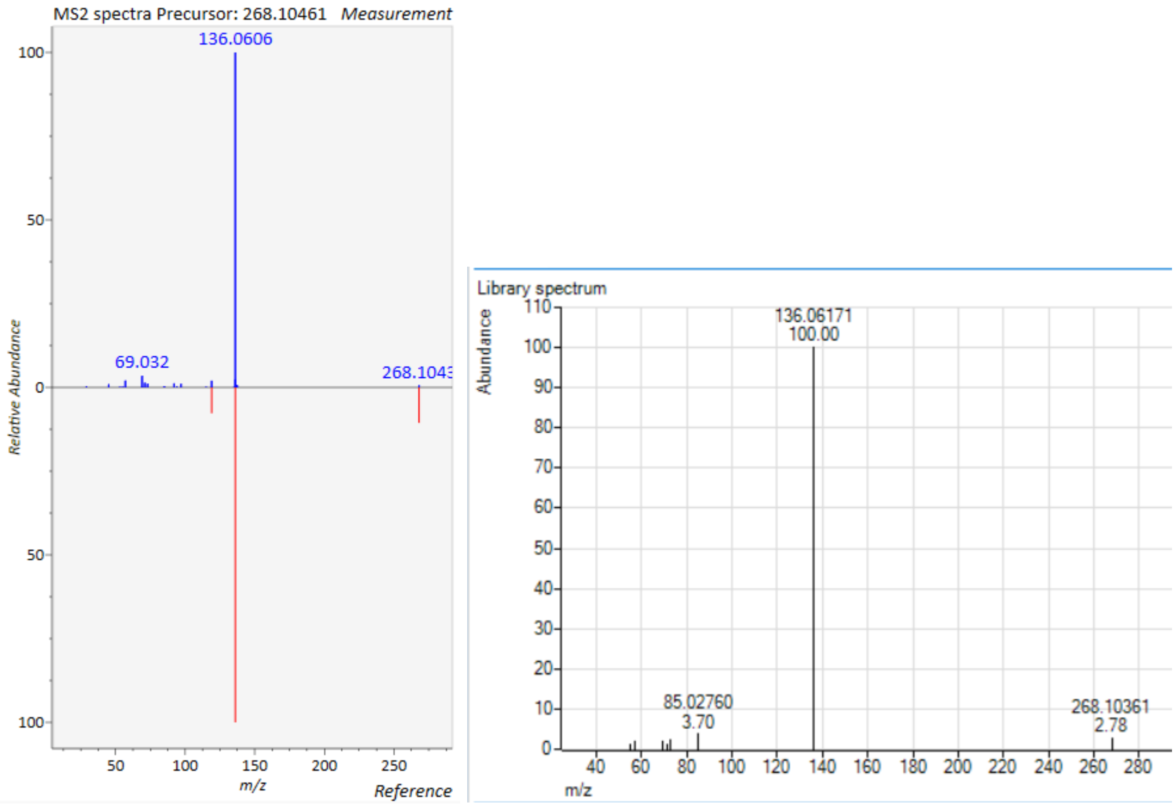

cytidine (1), hilic pos, m/z 244.0932 (M+H)+, MW 243.0858, RT 3.47 min, 40V, HMDB00089

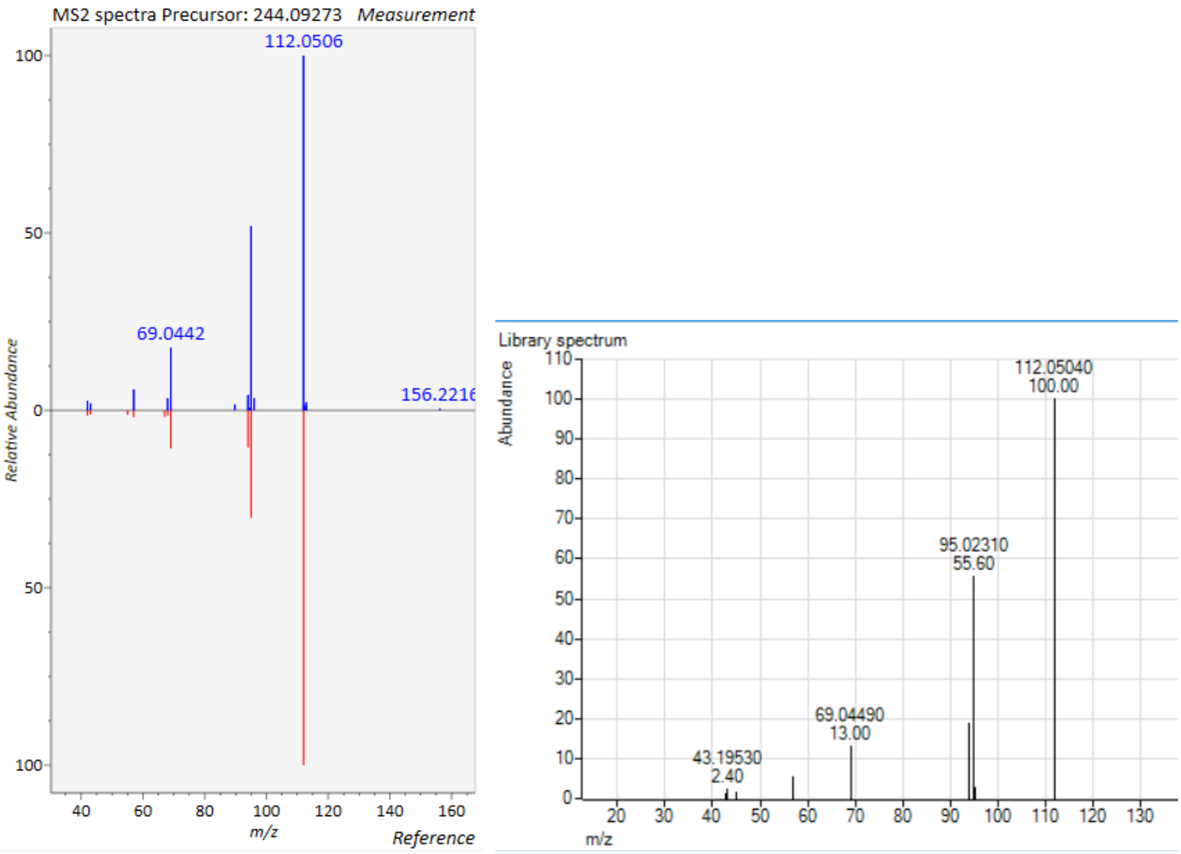

cytosine (1), hilic pos, m/z 112.0507 (M+H)+, MW 111.0431, RT 1.94 min, 40V, HMDB00630

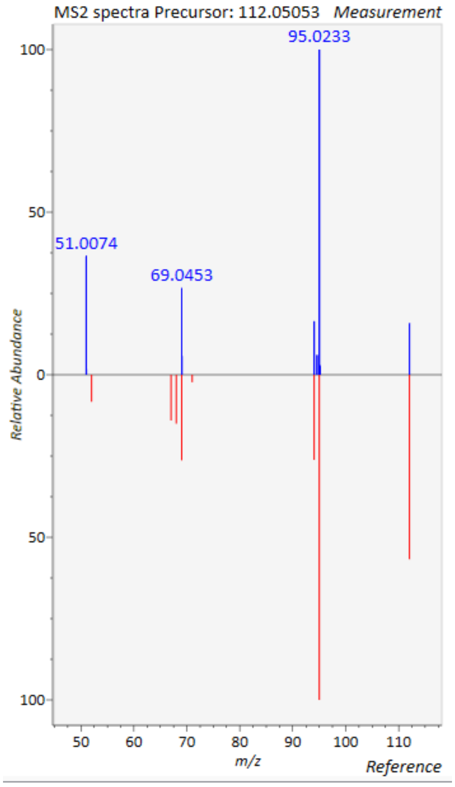

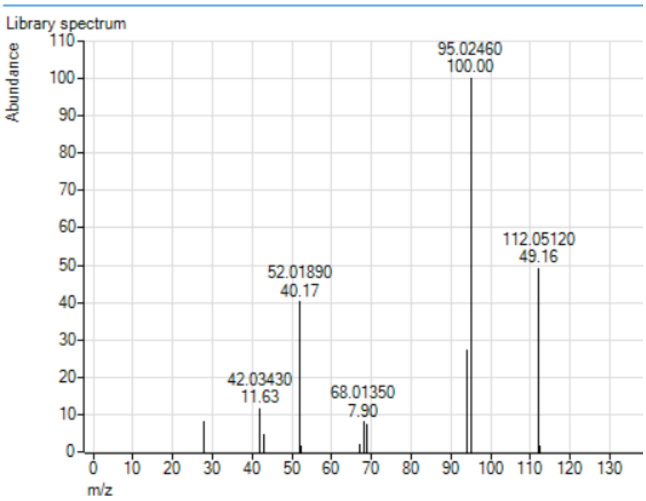

guanine (2), hilic pos, m/z 152.0567 (M+H)+, MW 151.0497, RT 2.88 min, 20V, HMDB00132

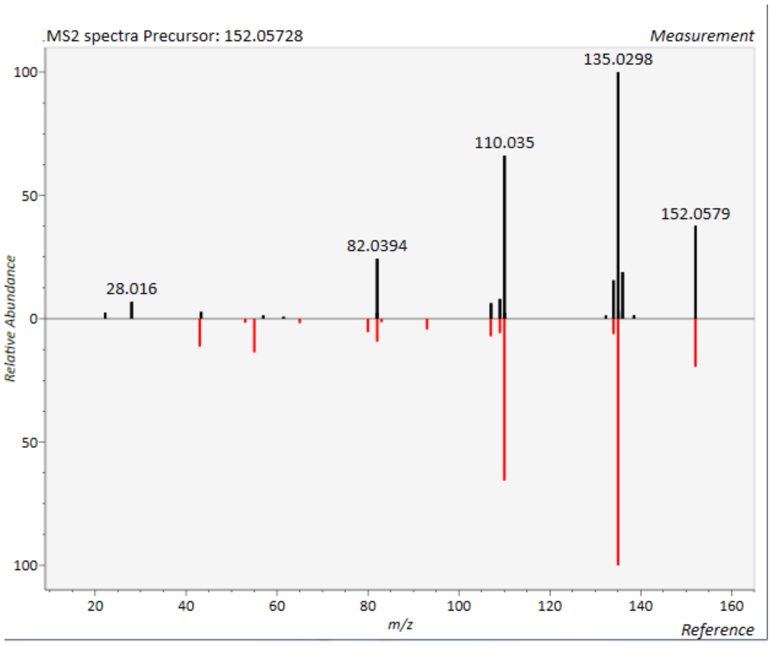

inosine (1), hilic pos, m/z 269.0887 (M+H)+, MW 268.0813, RT 2.55 min, 40V, HMDB00195

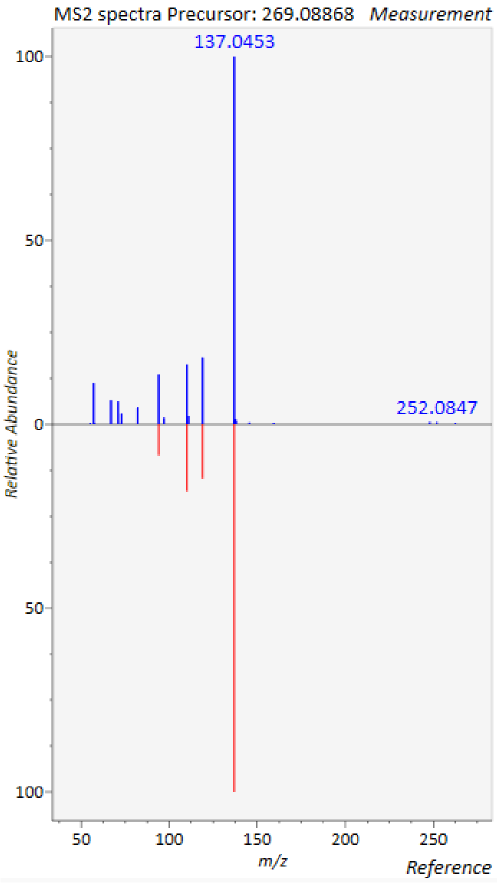

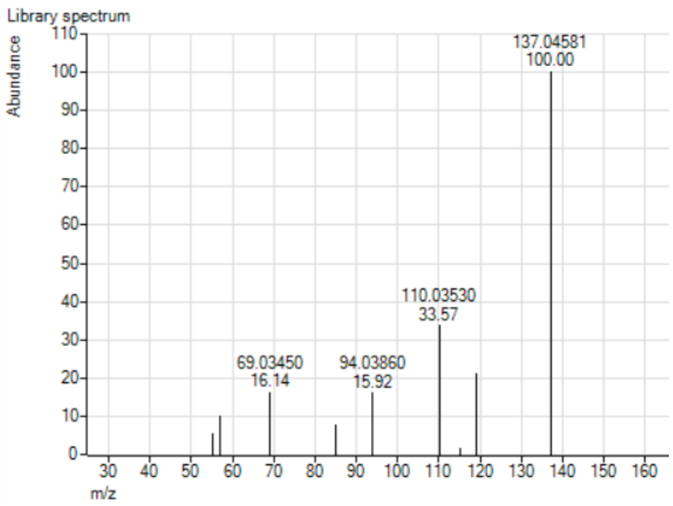

N6-methyl-adenine (2), hilic pos, m/z 150.0773 (M+H)+, MW 149.0702, RT 2.04 min, 20V, HMDB02099

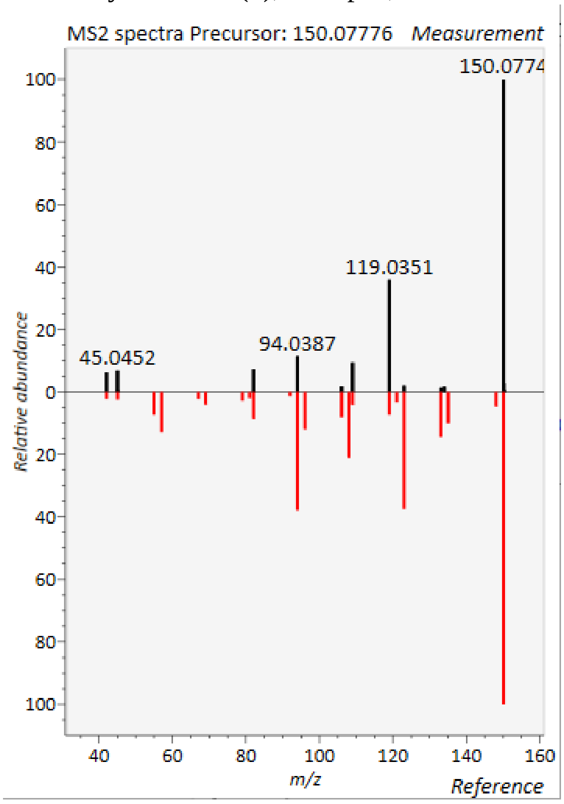

Organic acids

4-guanidinobutanoic acid (1), hilic pos, m/z 146.0925 (M+H)+, MW 145.0853, RT 3.80 min, 20V, HMDB03464

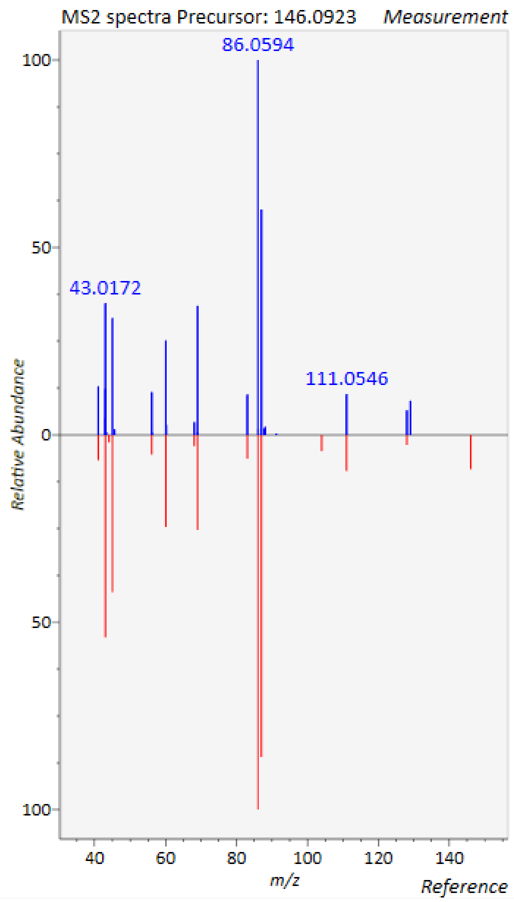

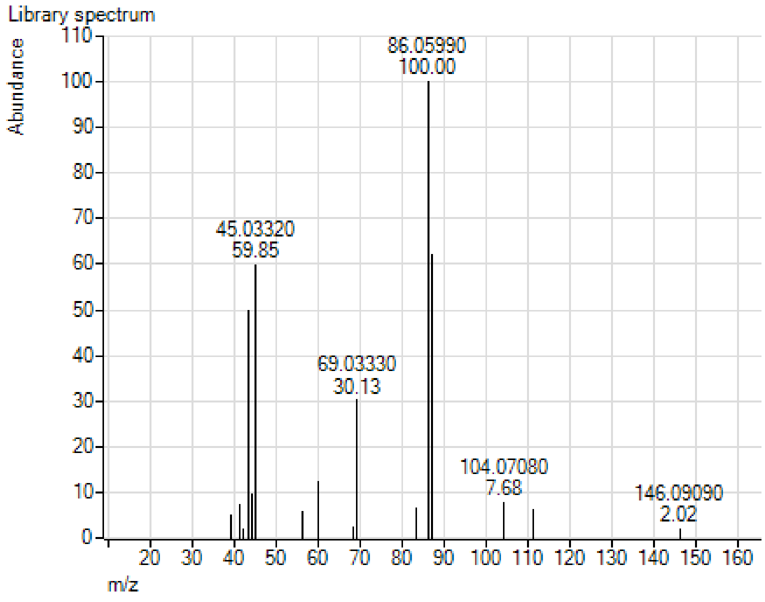

gamma-aminobutyric acid (GABA) (1), hilic pos, m/z 104.0705 (M+H)+, MW 103.0633, RT 4.98 min, 20V, HMDB00112

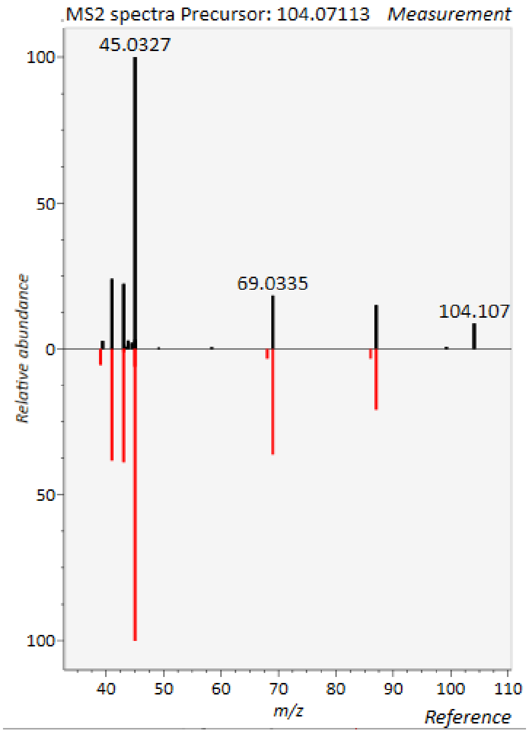

indoxyl sulfate (1), RP neg, m/z 212.0019 (M-H)-, MW 213.0092, RT 2.37 min, 10V, HMDB00682

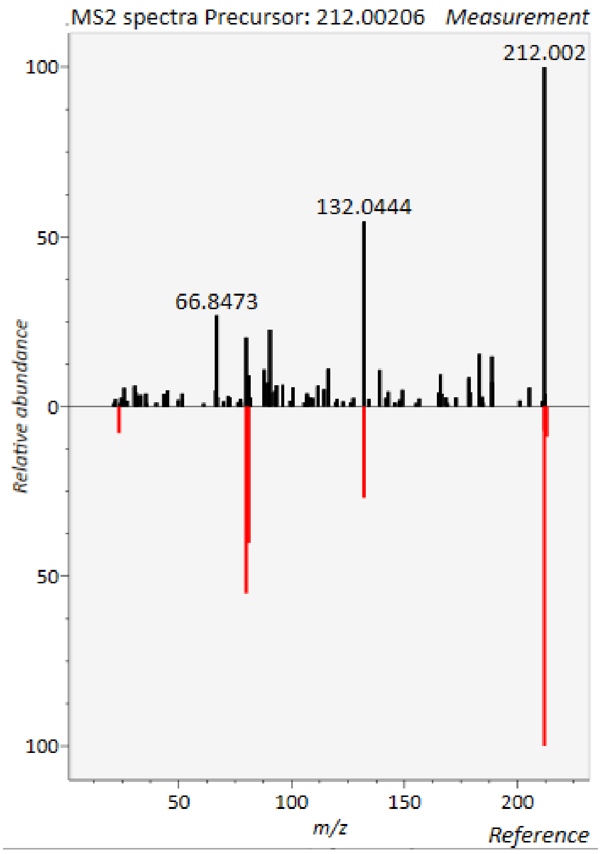

lactic acid (2), hilic neg, m/z 89.0244 (M-H)-, MW 90.0315, RT 1.38 min, 10V, HMDB00190

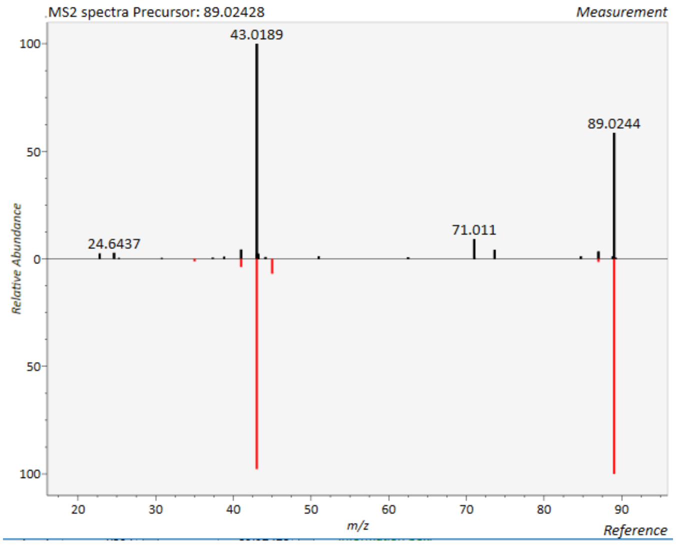

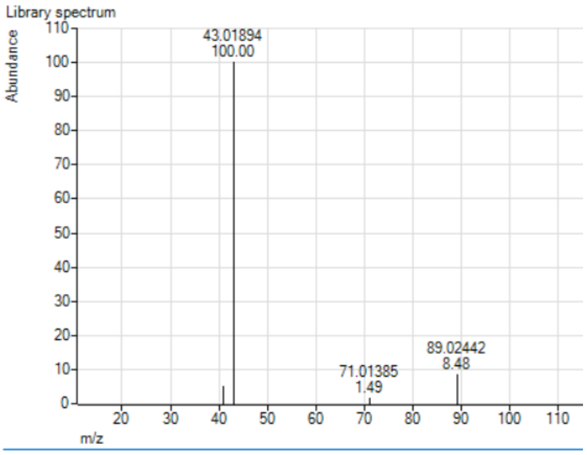

pyrocatechol sulfate (2), hilic neg, m/z 188.9863 (M-H)-, MW 189.9936, RT 0.63 min, 20V, HMDB59724

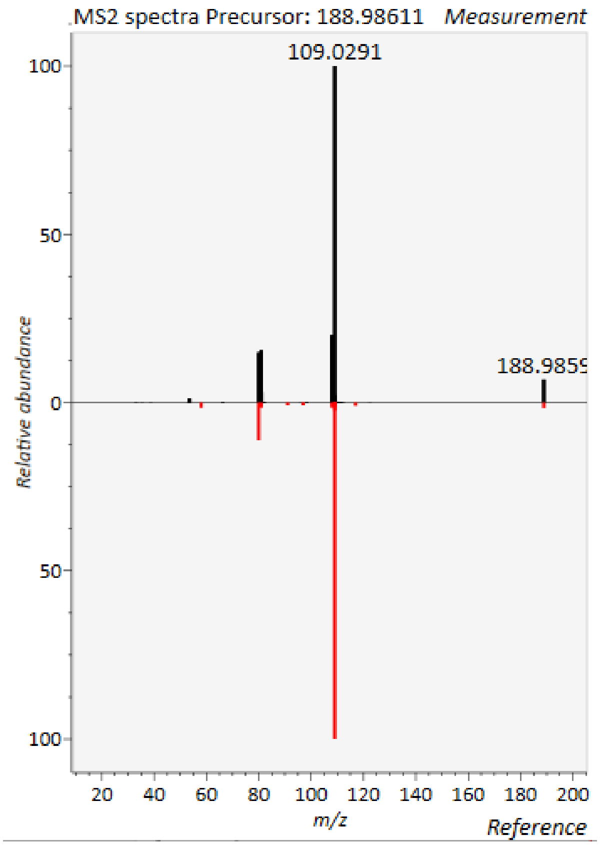

succinic acid (1), hilic neg, m/z 117.0195 (M-H)-, MW 118.0266, RT 1.02 min, 10V, HMDB00254

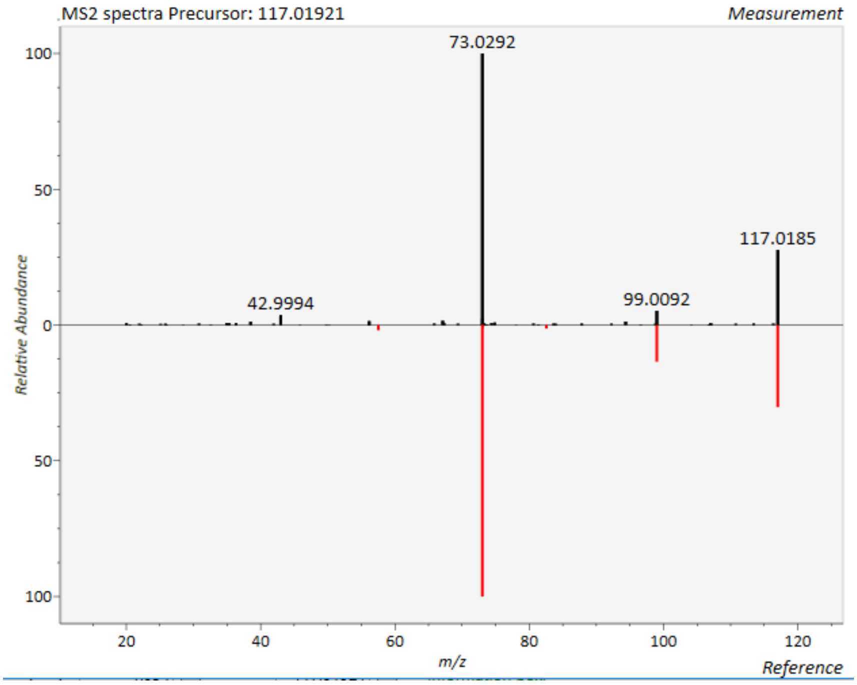

Other metabolites

1-methylnicotinamide (1), hilic pos, m/z 137.0708 (M+H)+, MW 136.0636, RT 2.24 min, 20V, HMDB00699

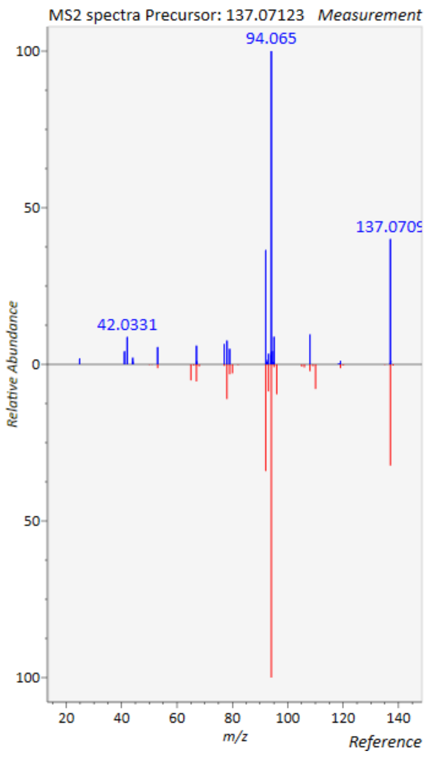

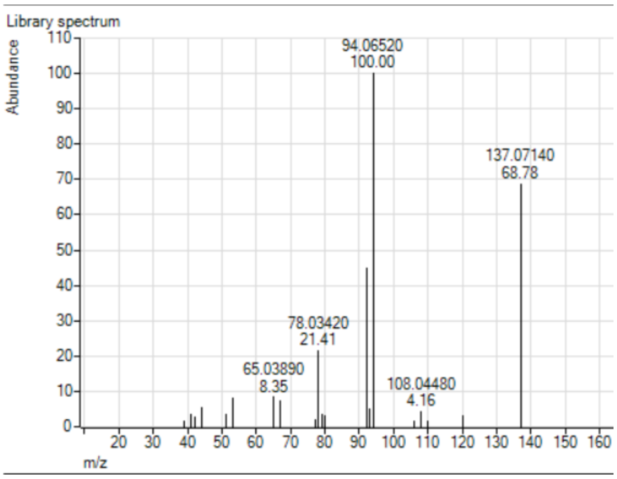

2-amino-1-phenylethanol (2), RP pos, m/z 120.081 (M+H-H2O)+, MW 137.0841, RT 1.38 min, 20V, HMDB01065

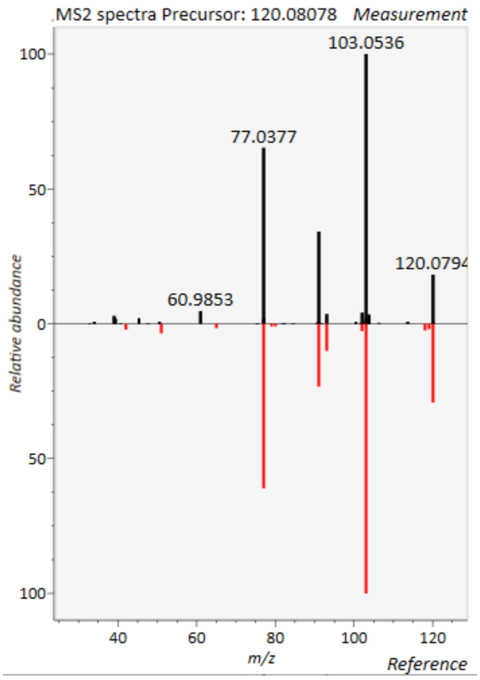

2-amino-2-methyl-1-propanol (2), hilic pos, m/z 90.0913 (M+H)+, MW 89.0836, RT 2.12 min, 20V, PubChem CID 11807

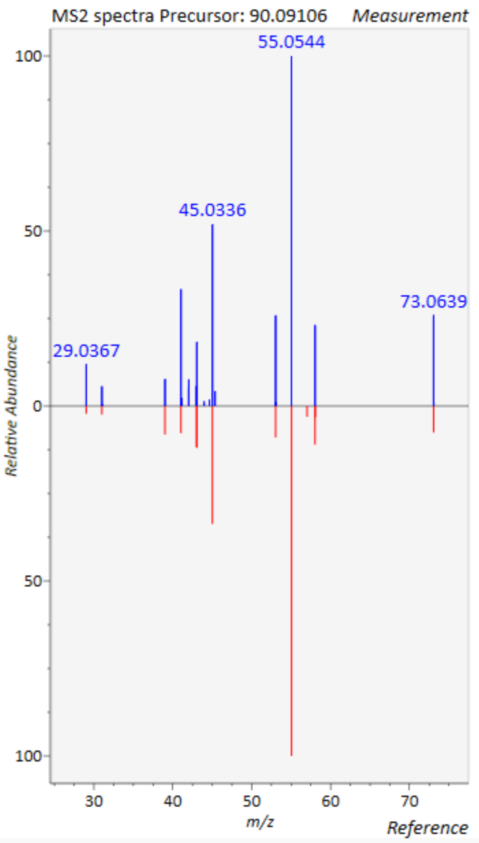

3-indoleacetic acid (1), RP pos, m/z 176.0706 (M+H)+, MW 175.0634, RT 4.82 min, 40V, HMDB00197

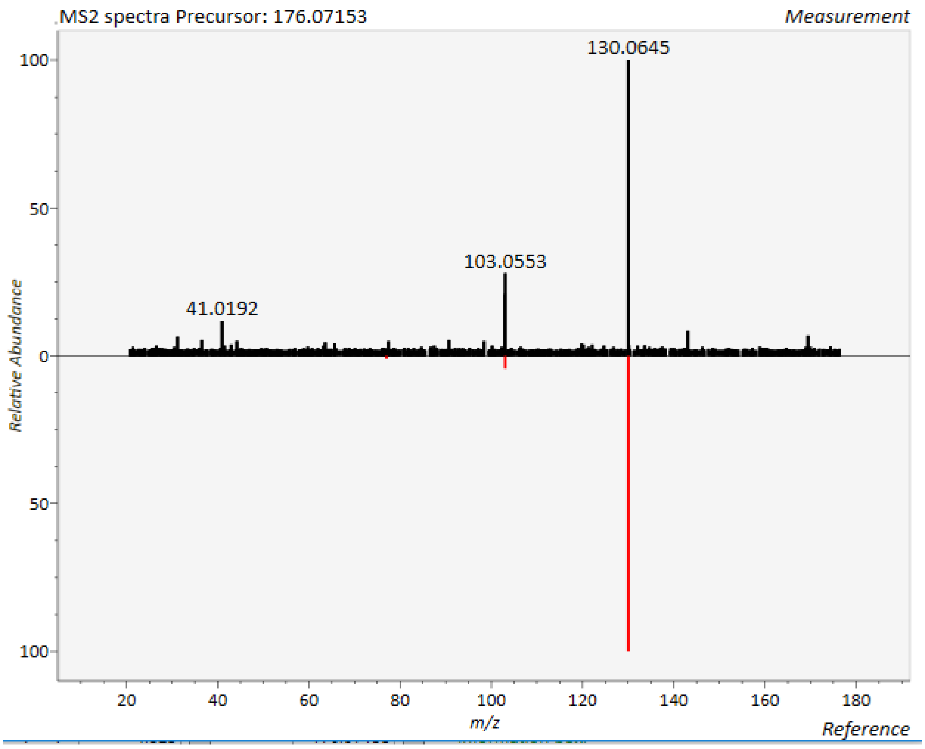

4-hydroxybenzaldehyde (1) RP neg, m/z 121.0297 (M-H)-, MW 122.0369, RT 3.30 min, 20V, HMDB11718

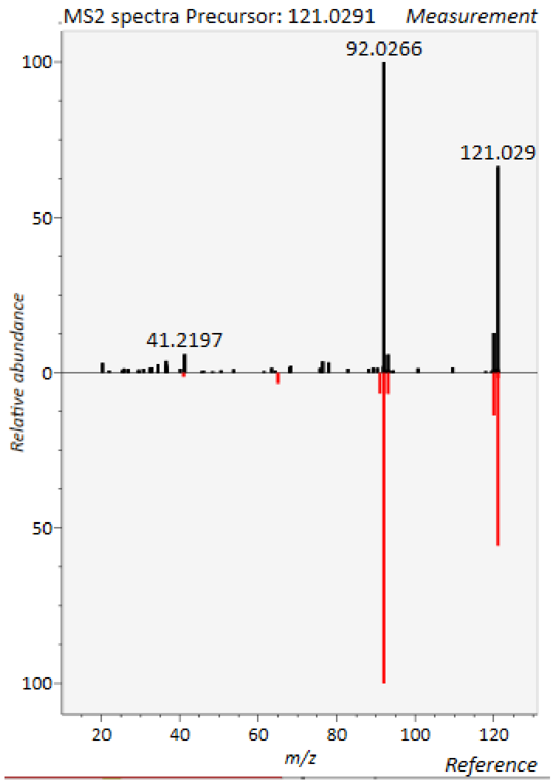

4-methylpyridine (2), hilic pos, m/z 94.0653 (M+H)+, MW 93.0579, RT 1.27 min, 20V, PubChem CID 7963

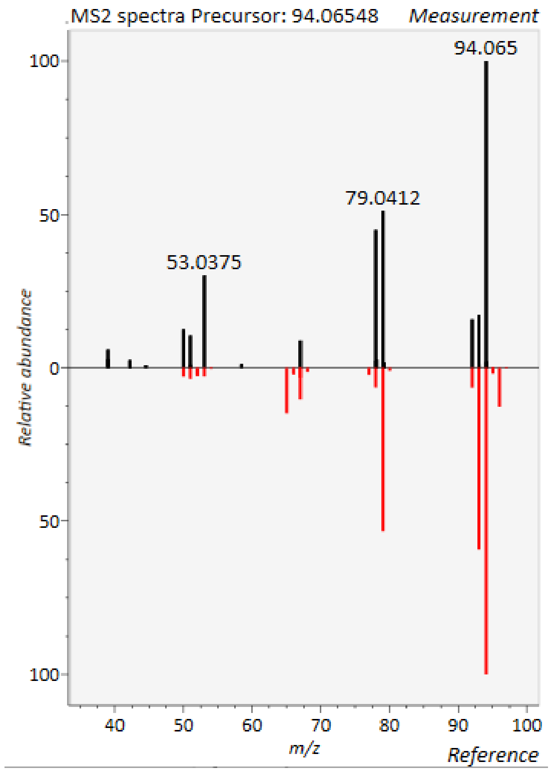

5-aminovaleric acid (1), hilic pos, m/z 118.0861 (M+H)+, MW 117.0792, RT 4.23 min, 10V, HMDB03355

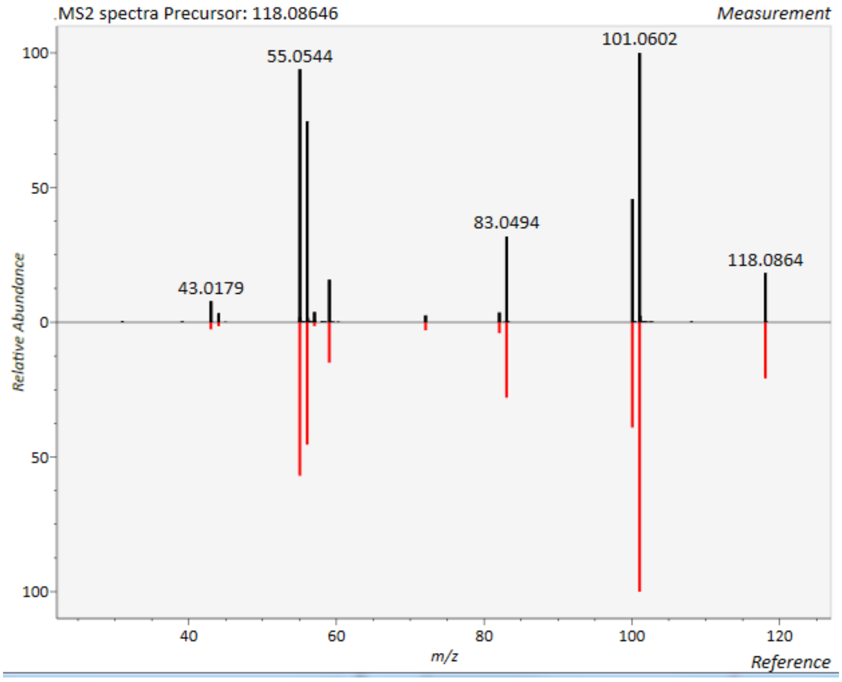

allantoin (1) hilic neg, m/z 157.0367 (M-H)-, MW 158.0439, RT 1.70 min, 20V, HMDB00462

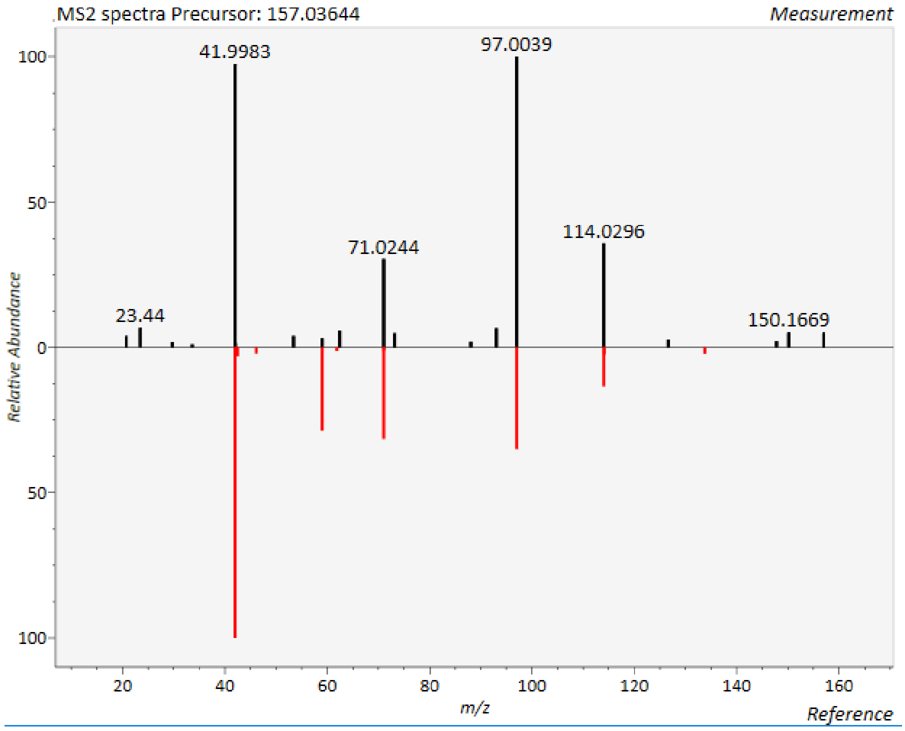

caffeine (1), hilic pos, m/z 195.0877 (M+H)+, MW 194.0809, RT 0.51 min, 20V, HMDB01847

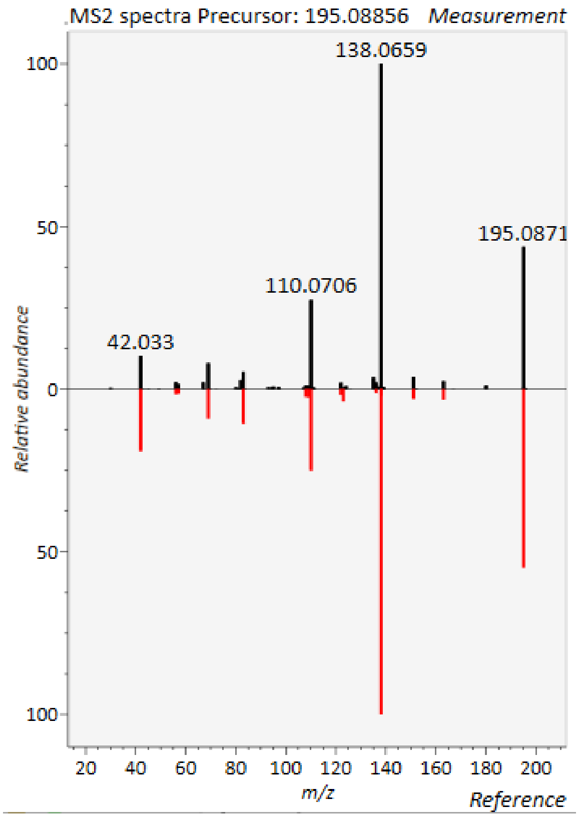

choline (2), hilic pos, m/z 104.107 (M+H)+, MW 103.1, RT 1.52 min, 20V, HMDB00097

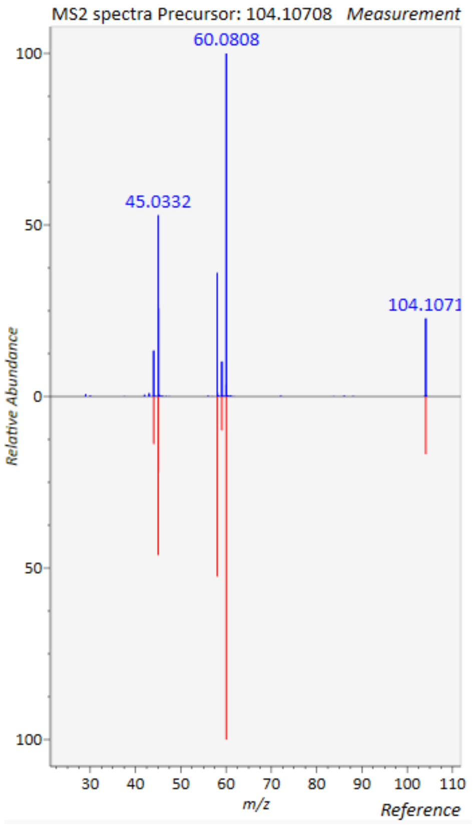

hydroxyphenyllactic acid (1) RP neg, m/z 181.0505 (M-H)-, MW 182.0579, RT 2.62 min, 10V, HMDB00755

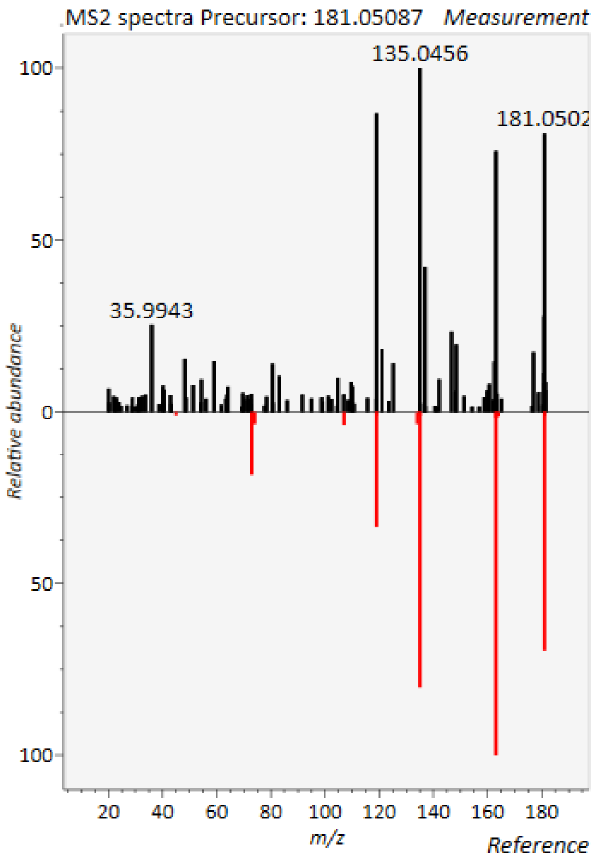

kynurenic acid (1), hilic pos, m/z 190.05 (M+H)+, MW 189.0426, RT 2.37 min, 20V, HMDB00715

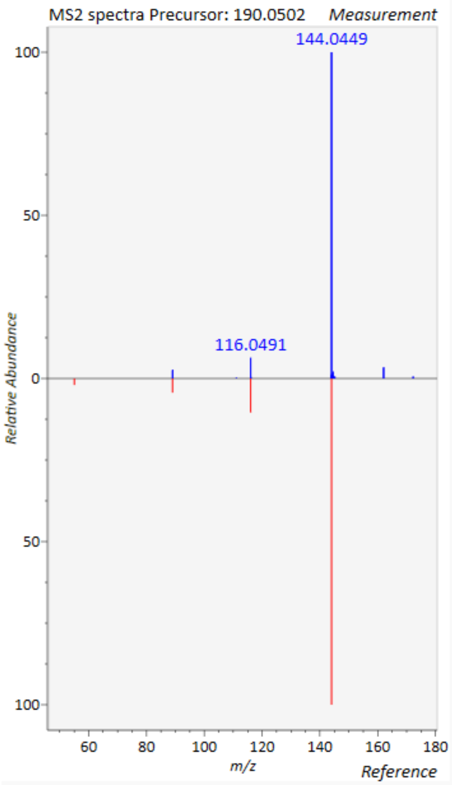

N-acetylgalactosamine 4-sulfate (2) hilic neg, m/z 300.0393 (M-H)-, MW 301.0463, RT 3.35 min, 20V, HMDB00781

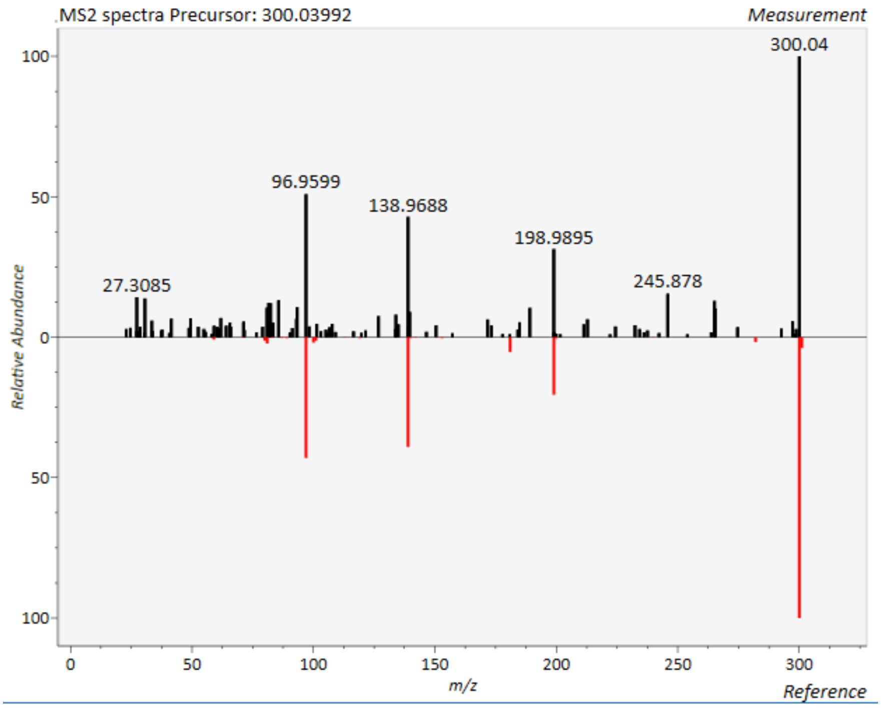

N-acetylglucosamine (2), hilic pos, m/z 222.098 (M+H)+, MW 221.0905, RT 3.90 min, 20V, HMDB00803

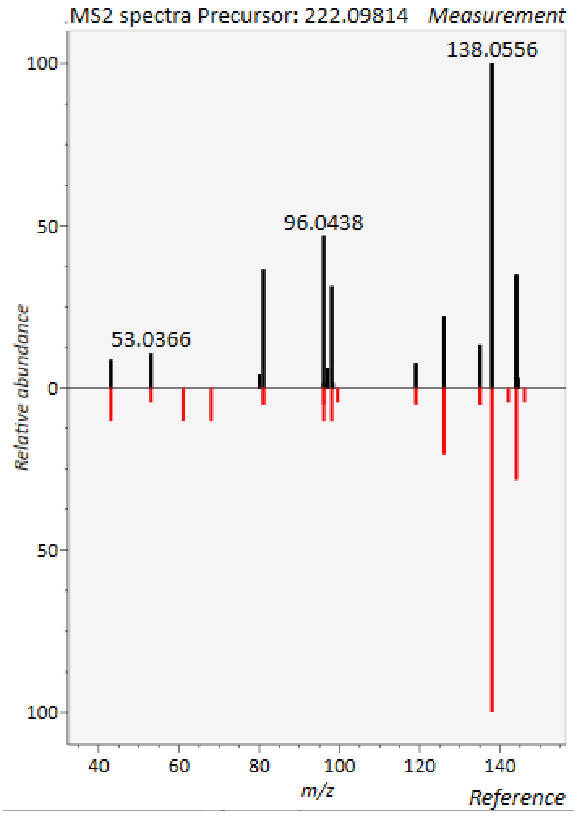

N-acetylneuraminic acid (1) hilic neg, m/z 308.0984 (M-H)-, MW 309.1054, RT 6.14 min, 20V, HMDB00800

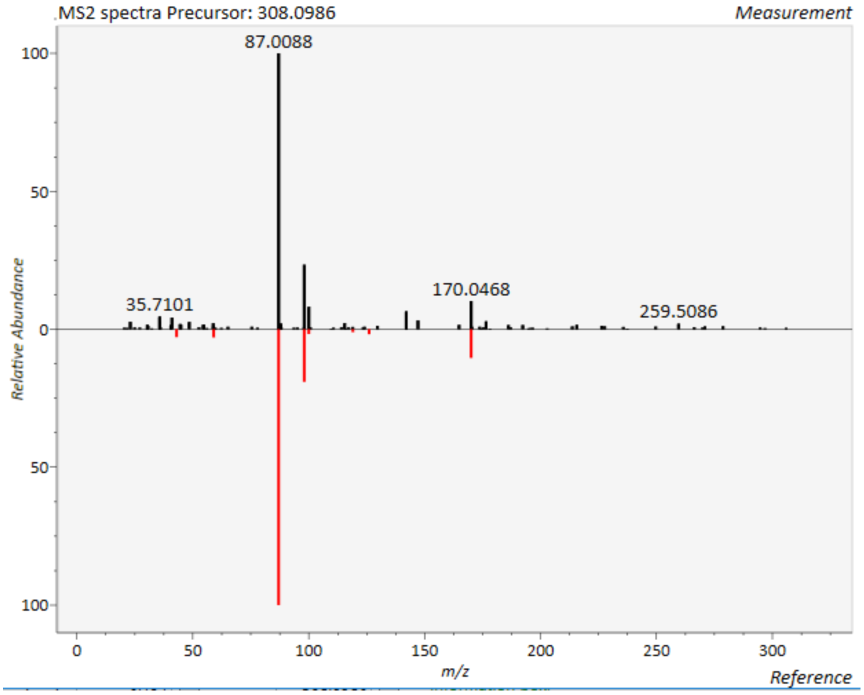

nicotinic acid (1), hilic pos, m/z 124.039 (M+H)+, MW 123.032, RT 1.35 min, 20V, HMDB01488

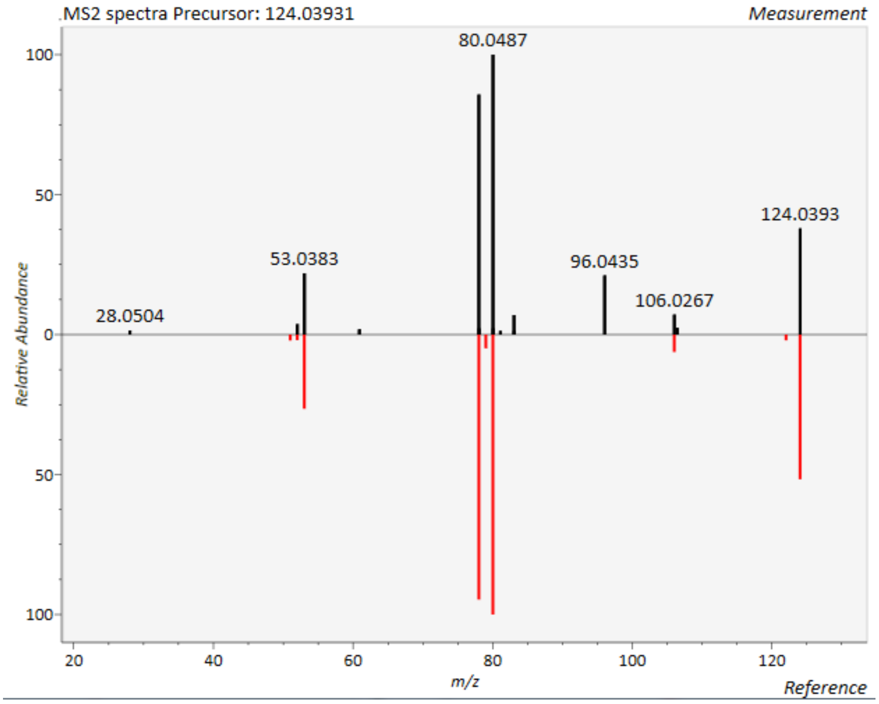

pantothenic acid (vitamin B5) (1) hilic neg, m/z 218.1034 (M-H)-, MW 219.1103, RT 0.95 min, 10V, HMDB00210

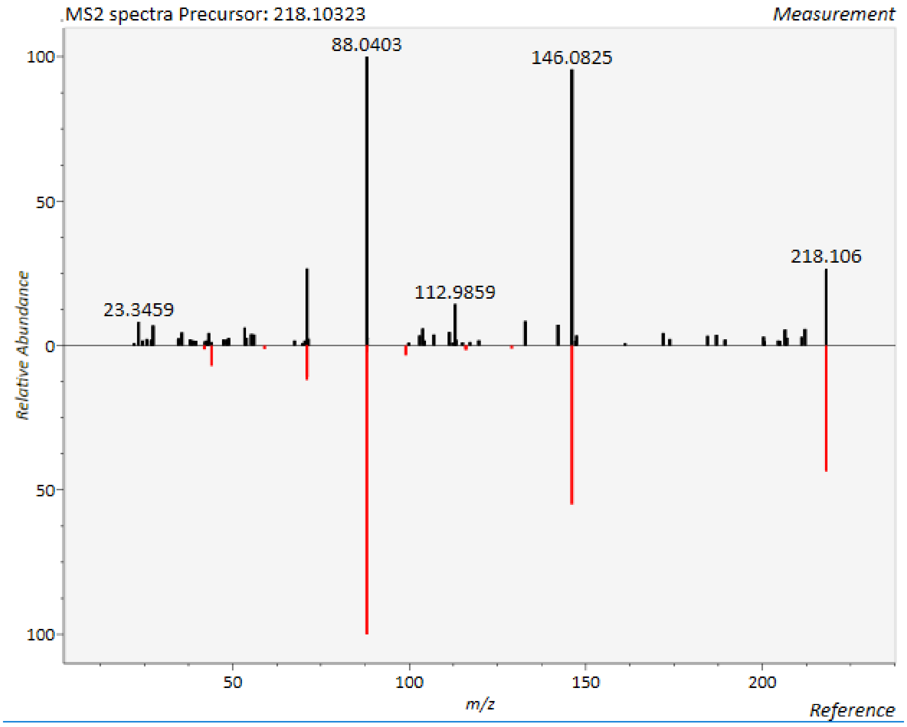

paraxanthine (1), RP pos, m/z 181.0723 (M+H)+, MW 180.0649, RT 2.64 min, 20V, HMDB01860

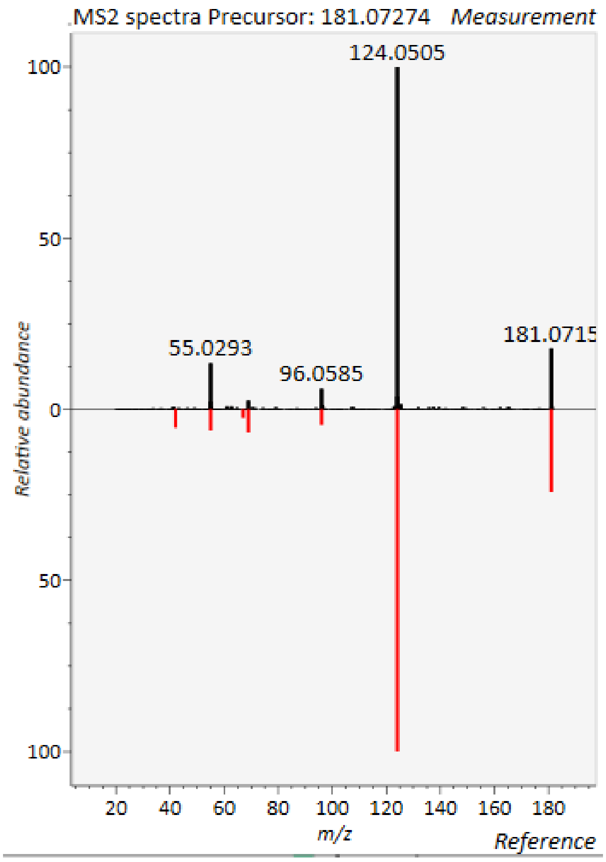

phosphocholine (1), hilic pos, m/z 184.0735 (M+H)+, MW 183.0662, RT 6.97 min, 20V, HMDB01565

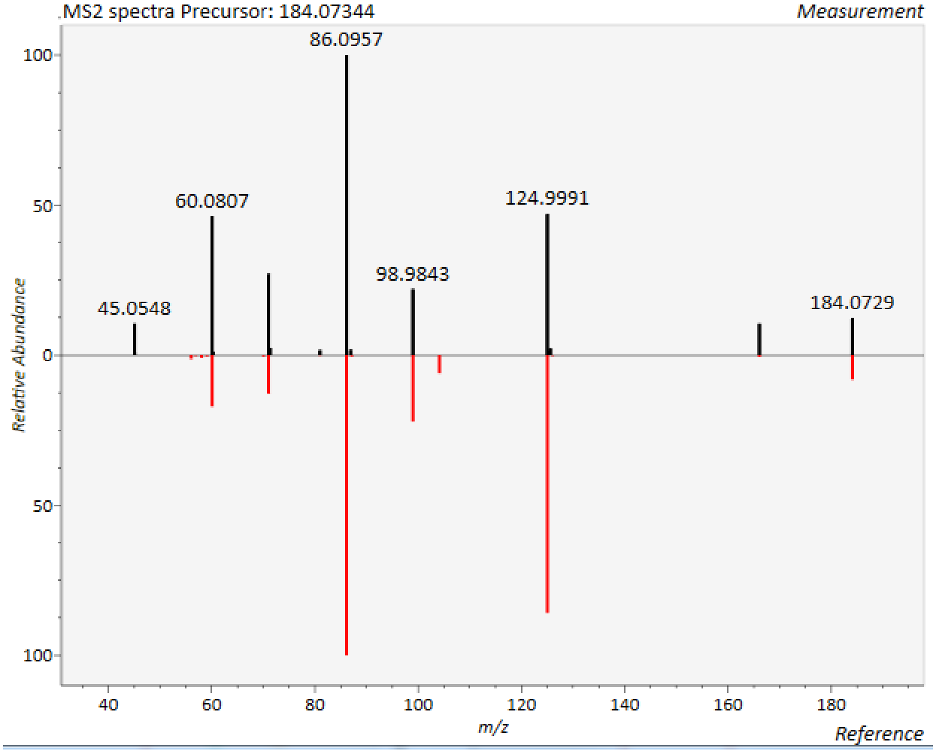

purine (2), hilic pos, m/z 121.0508 (M+H)+, MW 120.0435, RT 0.77 min, 20V, HMDB01366

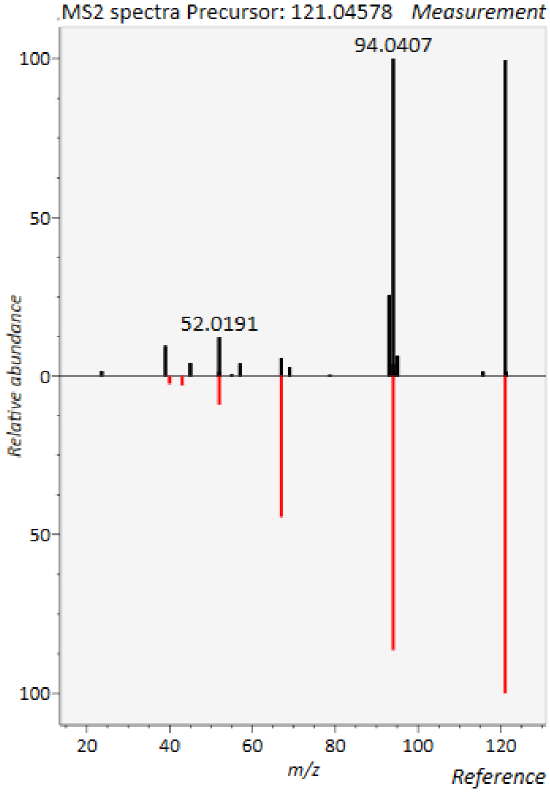

quinaldic acid (2), RP pos, m/z 174.0551 (M+H)+, MW 173.0479, RT 3.18 min, 10V, HMDB00842

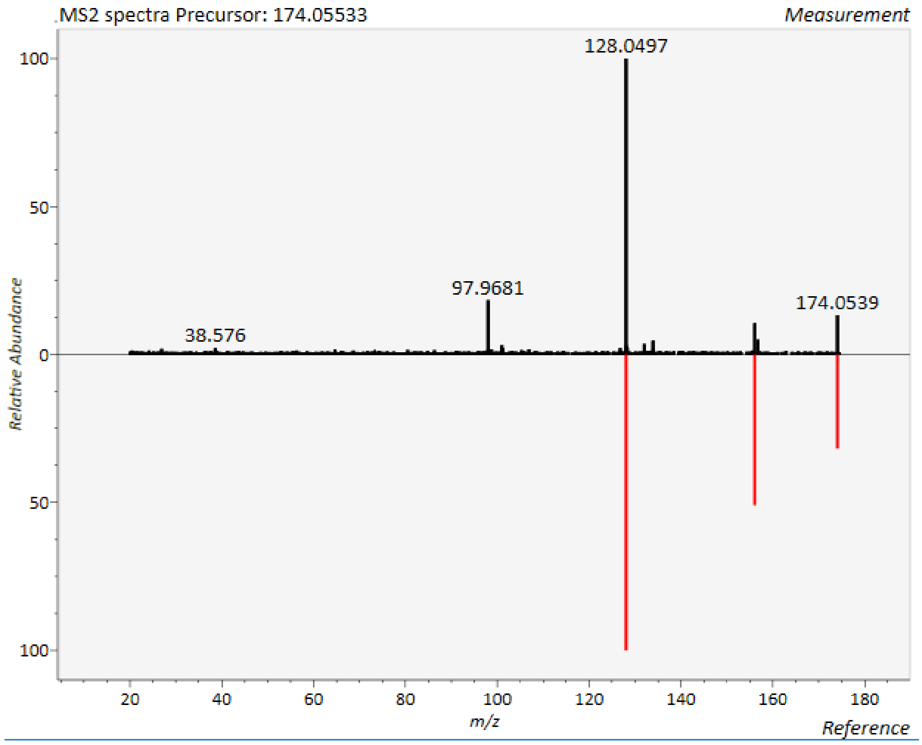

riboflavin (vitamin B2) (1), RP pos, m/z 377.1461 (M+H)+, MW 376.139, RT 3.99 min, 20V, HMDB00244

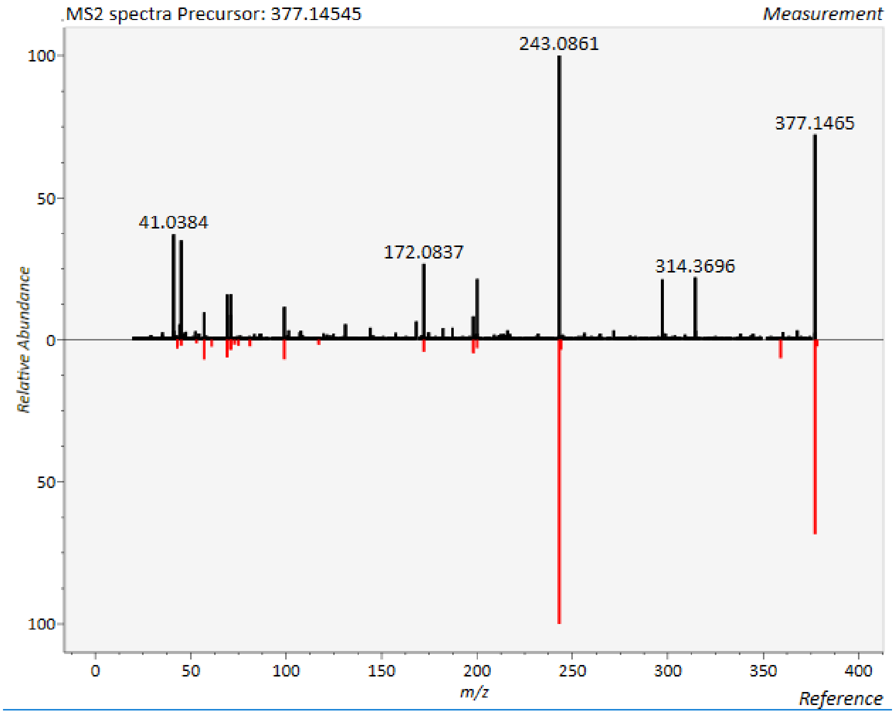

sphinganine (1), RP pos, m/z 302.3059 (M+H)+, MW 301.2989, RT 8.89 min, 20V, HMDB00269

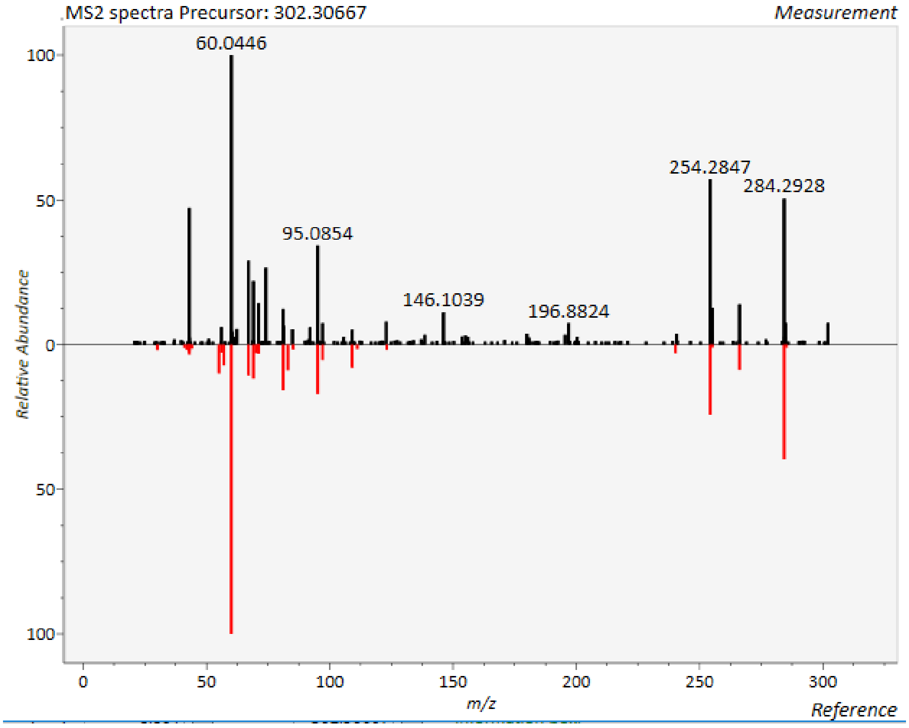

sphingosine (2), RP pos, m/z 300.2905 (M+H)+, MW 299.2829, RT 8.73 min, 20V, HMDB00252

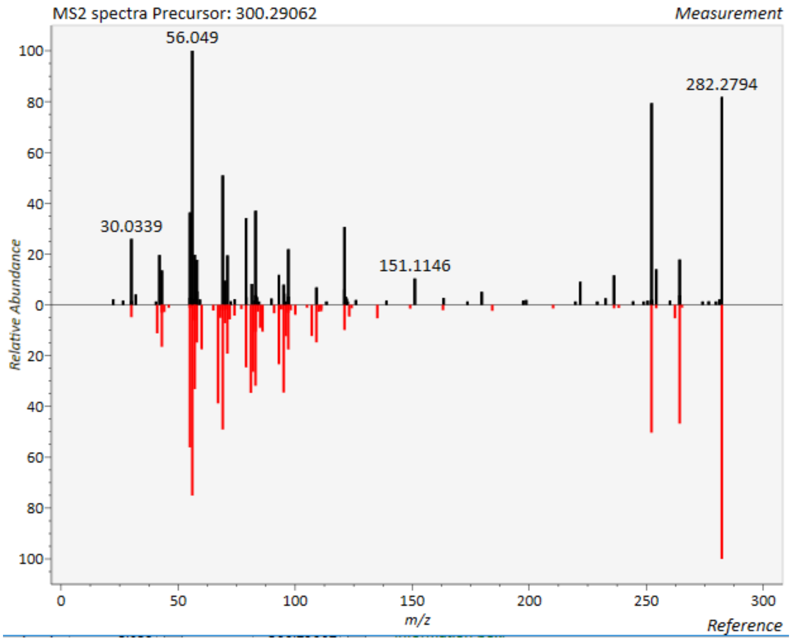

theobromine (1), RP pos, m/z 181.0723 (M+H)+, MW 180.0648, RT 2.26 min, 20V, HMDB02825

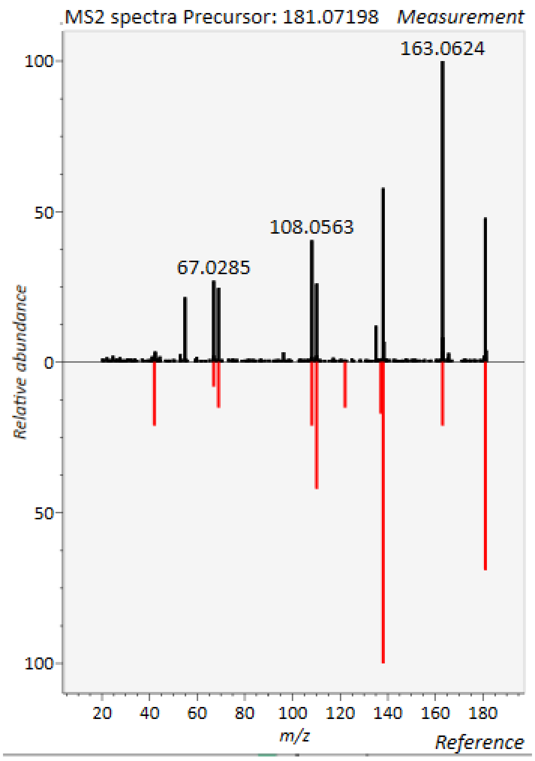

trigonelline (1), hilic pos, m/z 138.055 (M+H)+, MW 137.048, RT 4.22 min, 20V, HMDB00875

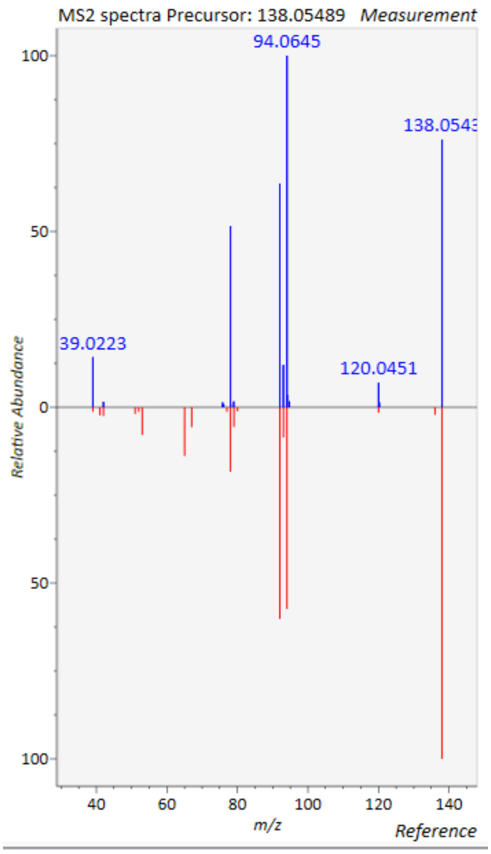

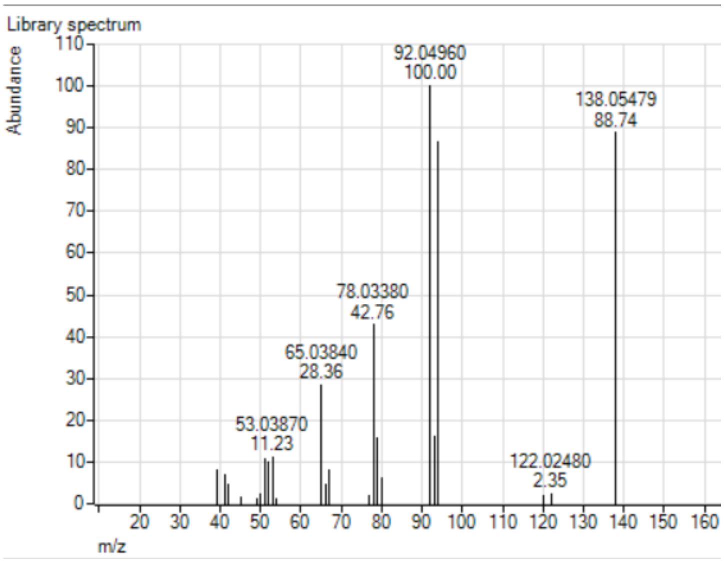

urea (2), hilic pos, m/z 61.0399 (M+H)+, MW 60.032, RT 0.98 min, 20V, HMDB00294

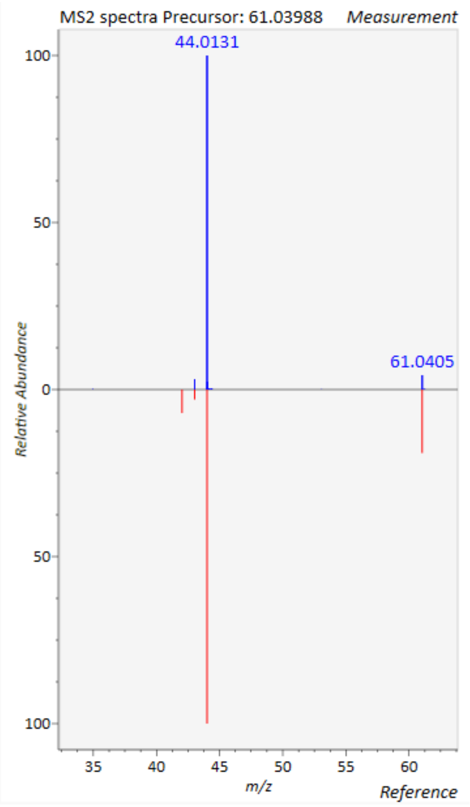

urocanic acid (1) hilic neg, m/z 137.0355 (M-H)-, MW 138.0429, RT 0.99 min, 10V, cis: HMDB0000301 trans: HMDB62562

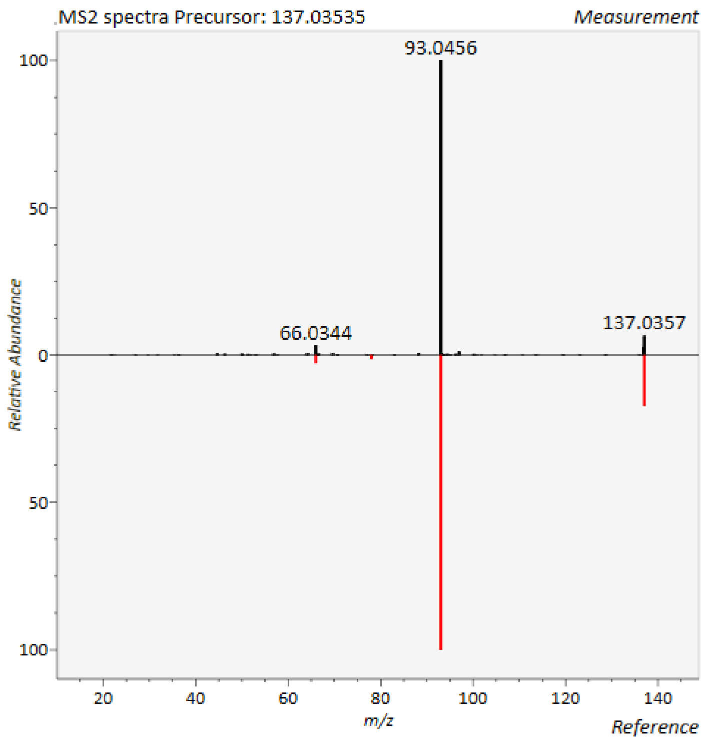

usnic acid (2) RP neg, m/z 343.0818 (M-H)-, MW 344.0889, RT 9.93 min, 20V, PubChem CID 5646

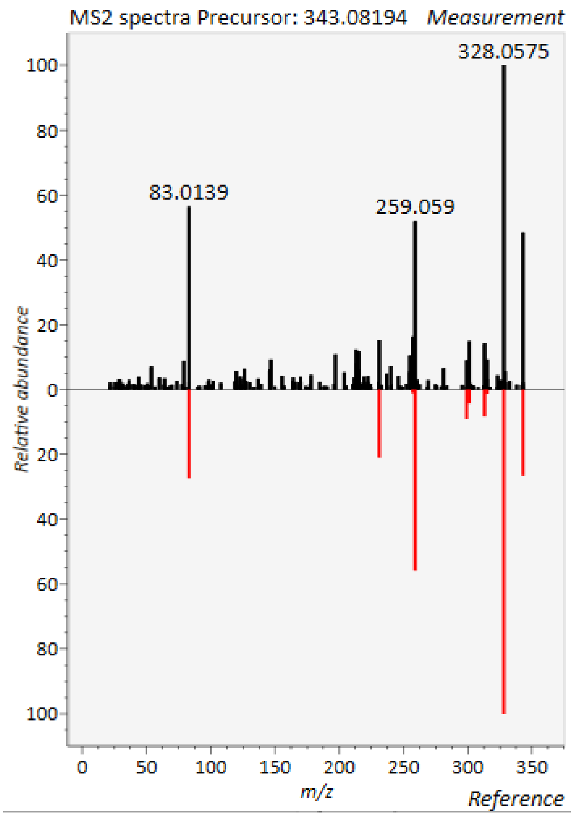

xanthine (1) hilic neg, m/z 151.026 (M-H)-, MW 152.0334, RT 1.53 min, 20V, HMDB00292

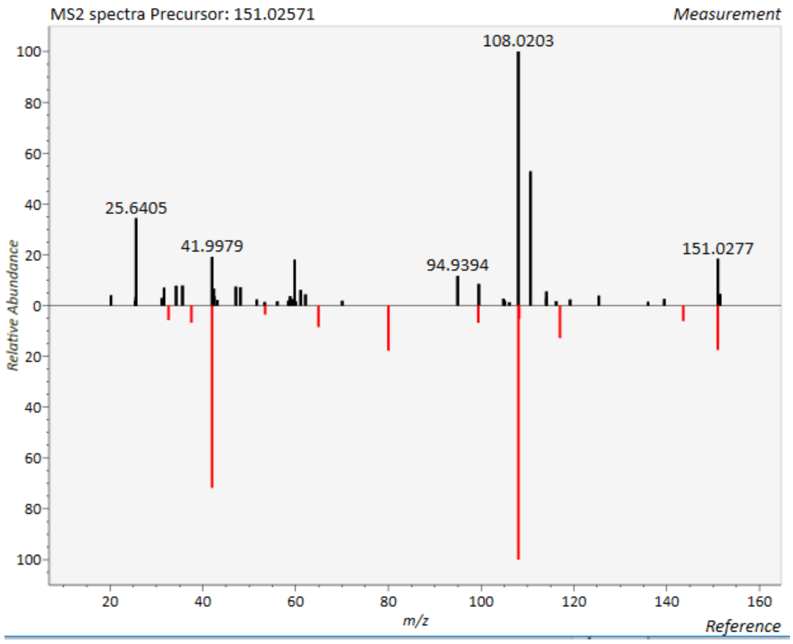

Small peptides

arg-ile (2), hilic pos, m/z 288.2042 (M+H)+, MW 287.1953, RT 6.05 min, 20V, HMDB28712

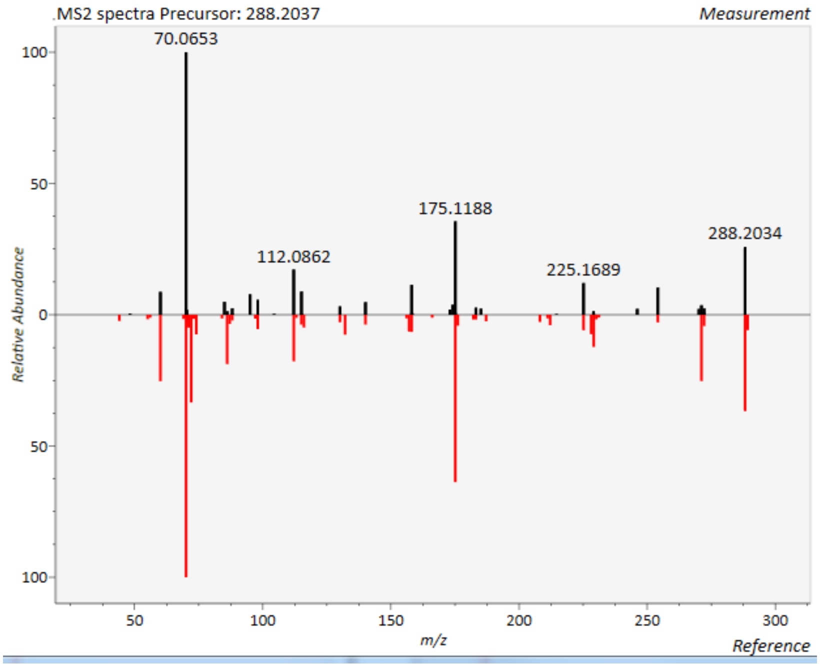

arg-phe (2), hilic pos, m/z 322.1886 (M+H)+, MW 321.1808, RT 6.02 min, 20V, HMDB28716

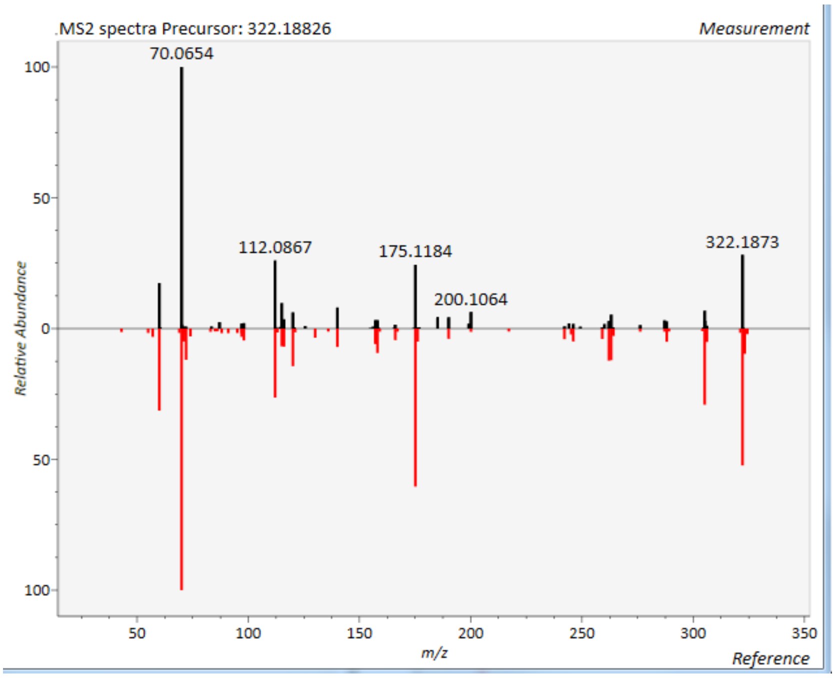

arg-ser (2), hilic pos, m/z 262.1516 (M+H)+, MW 261.1441, RT 7.36 min, 20V, HMDB28718

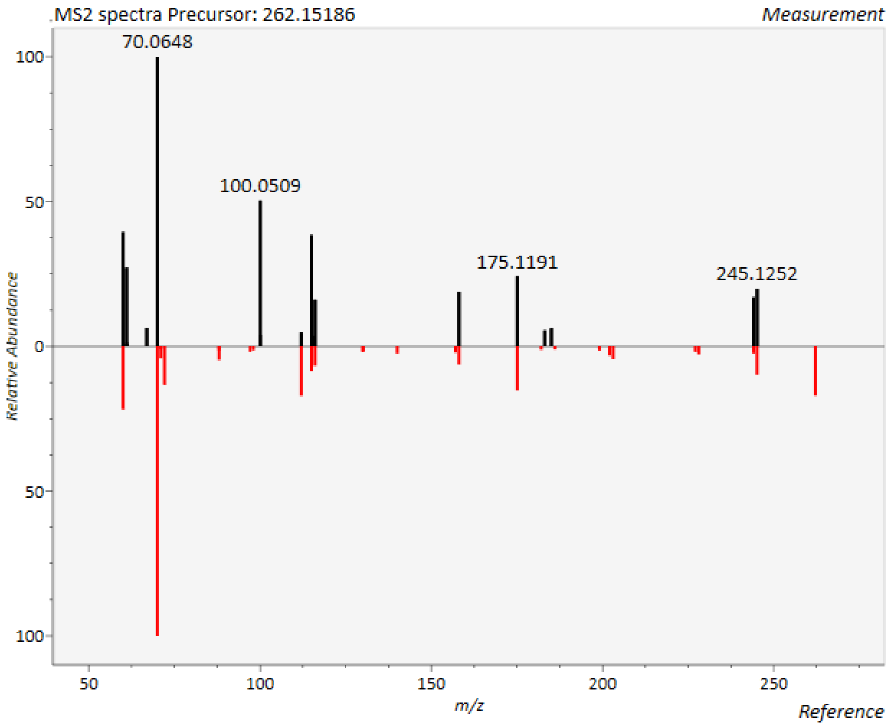

gly-pro (2), hilic pos, m/z 173.0922 (M+H)+, MW 172.0845, RT 5.98 min, 10V, HMDB00721

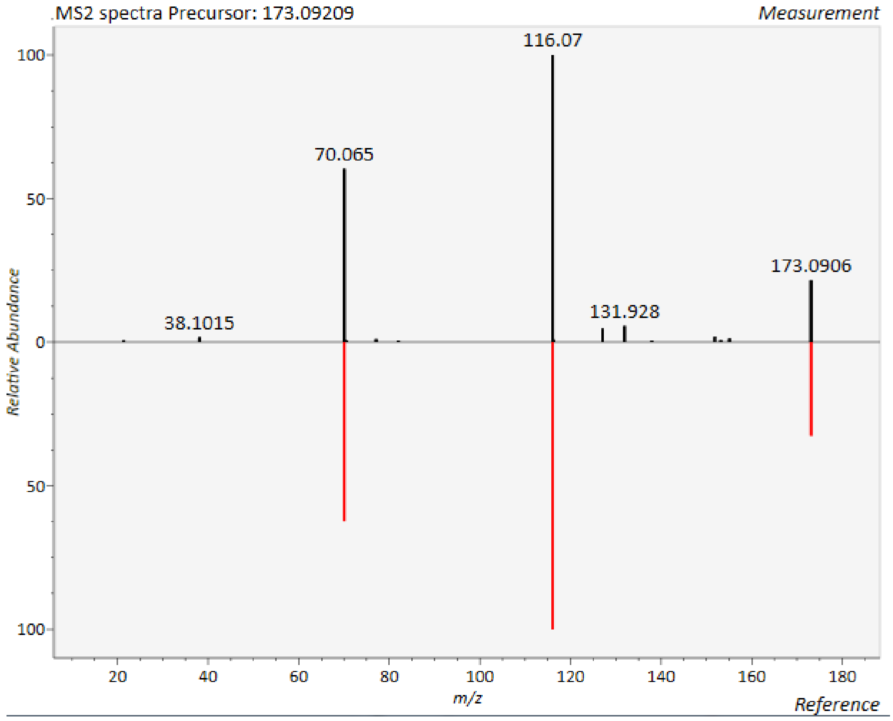

gly-tyr (2), hilic pos, m/z 239.1033 (M+H)+, MW 238.0959, RT 5.61 min, 20V, HMDB28853

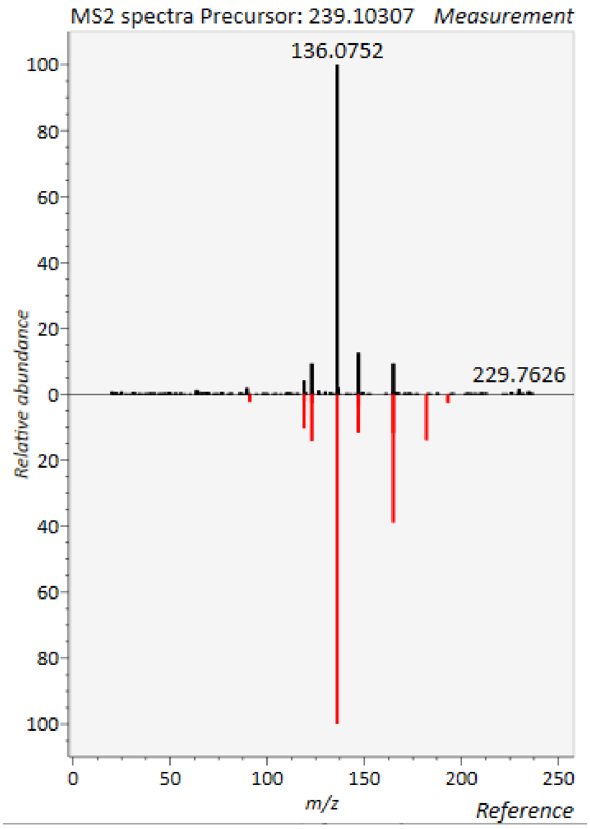

his-glu (2), hilic pos, m/z 285.1199 (M+H)+, MW 284.113, RT 7.09 min, 20V, HMDB28884

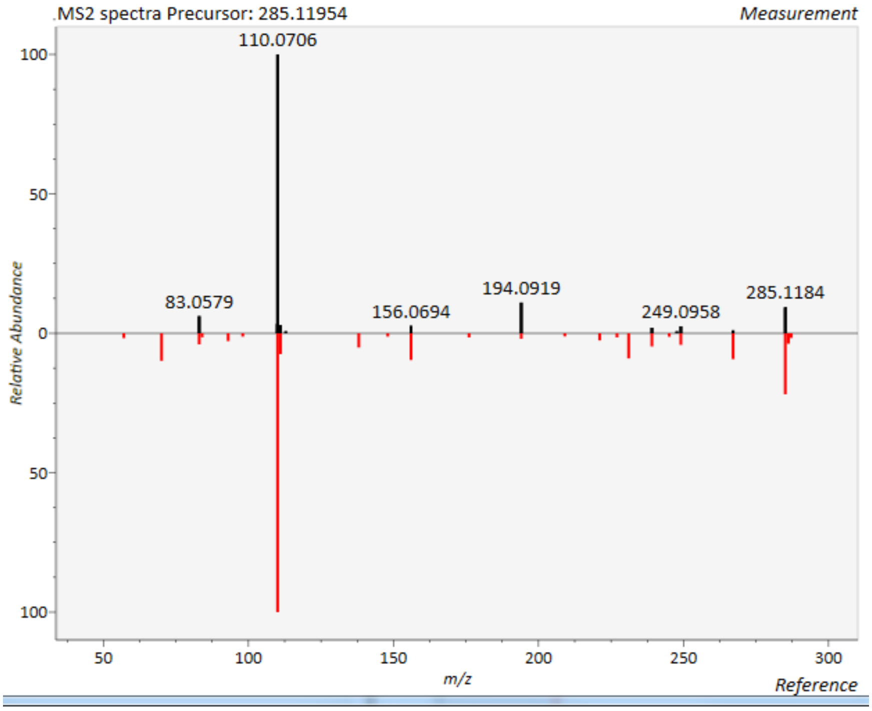

his-gly (2), hilic pos, m/z 213.0989 (M+H)+, MW 212.0915, RT 6.93 min, 20V, HMDB28885

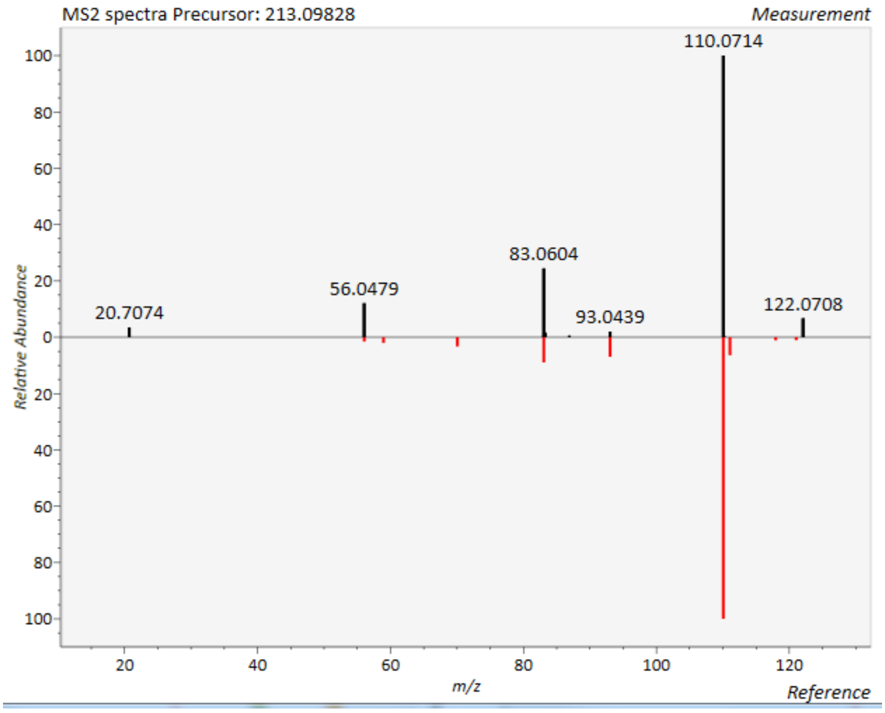

his-his (2), hilic pos, m/z 293.1365 (M+H)+, MW 292.1286, RT 7.61 min, 20V, HMDB28887

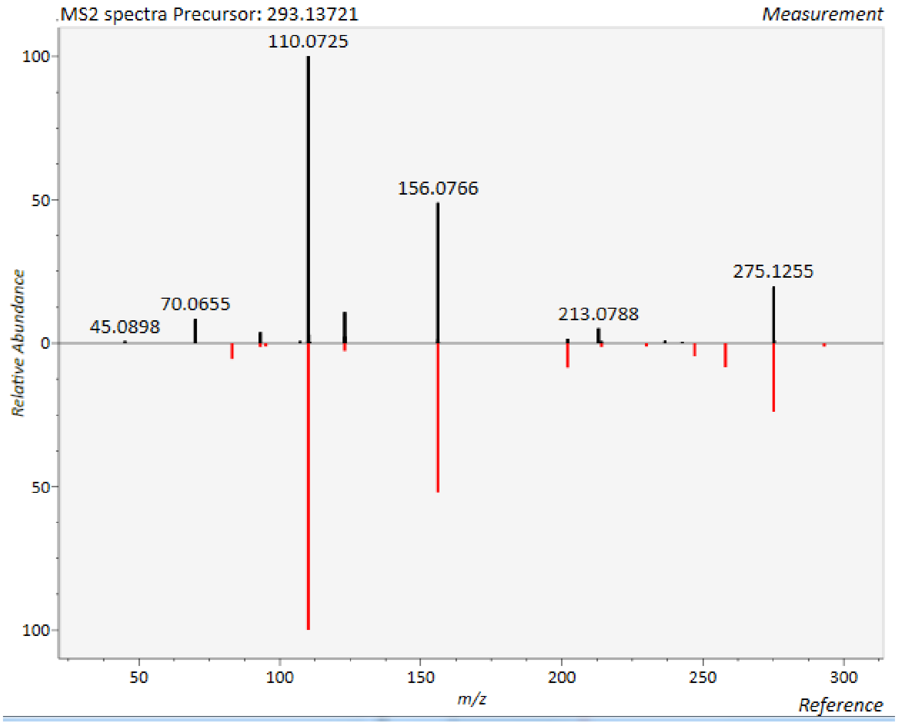

his-ile/leu (2), hilic pos, m/z 269.1614 (M+H)+, MW 268.1544, RT 5.55 min, 20V, his-ile: HMDB28888, his-leu: HMDB28889

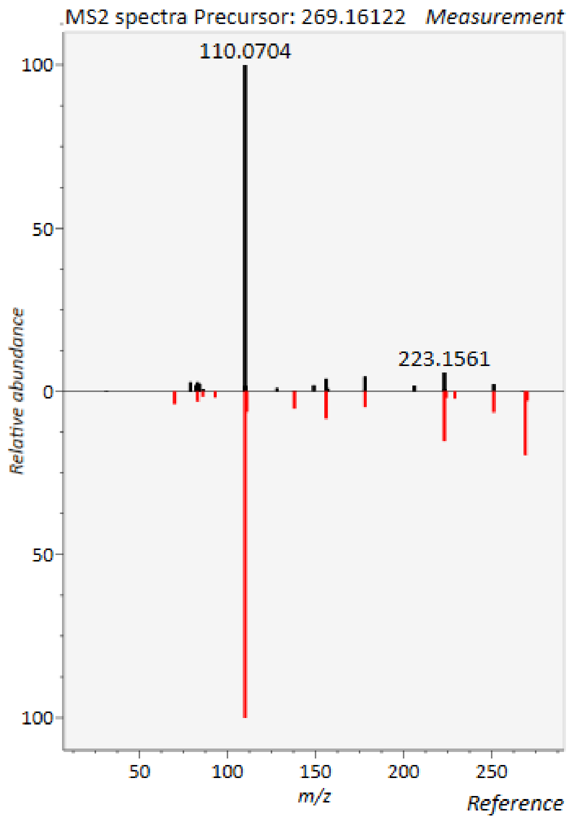

his-ser (2), hilic pos, m/z 243.1094 (M+H)+, MW 242.102, RT 7.14 min, 40V, HMDB28894

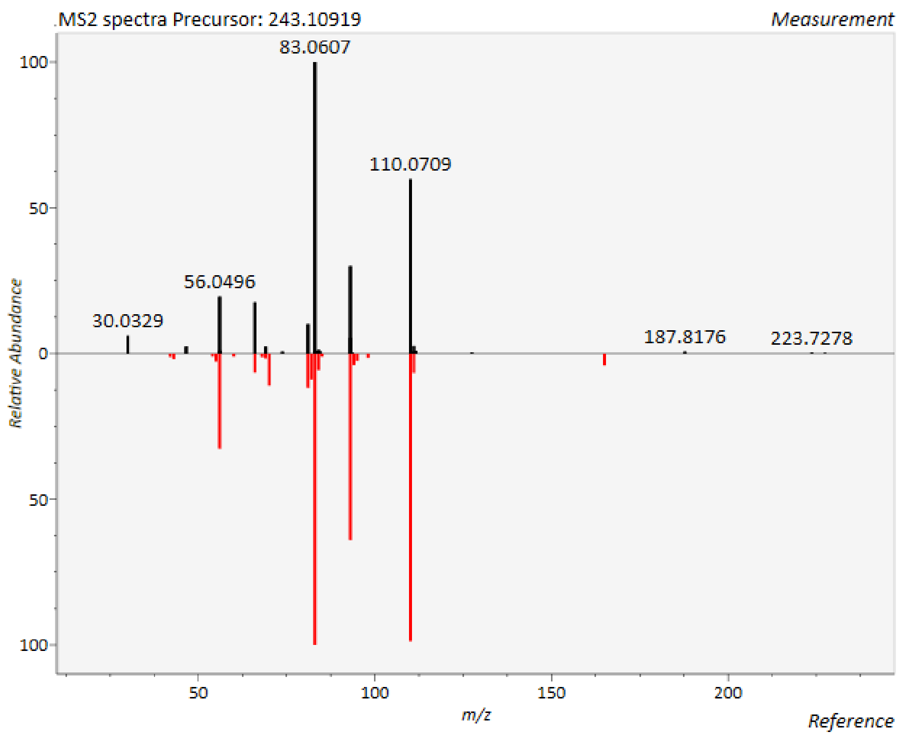

ile-ser (2), hilic pos, m/z 219.1345 (M+H)+, MW 218.1273, RT 5.52 min, 20V, HMDB28916

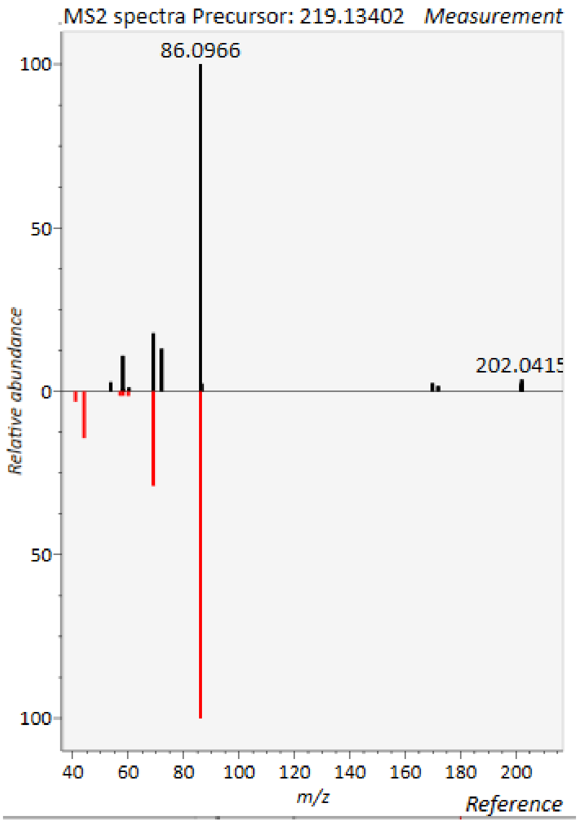

leu-leu (2), hilic pos, m/z 245.1867 (M+H)+, MW 244.179, RT 1.97 min, 40V, HMDB28933

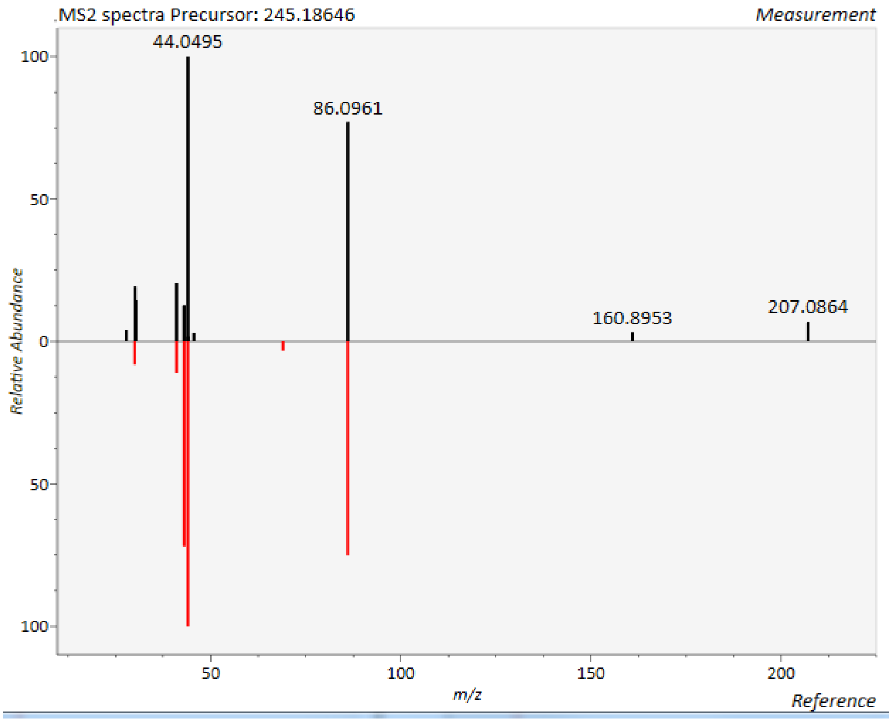

leu-phe (2), RP pos, m/z 279.171 (M+H)+, MW 278.1633, RT 4.10 min, 20V, HMDB13243

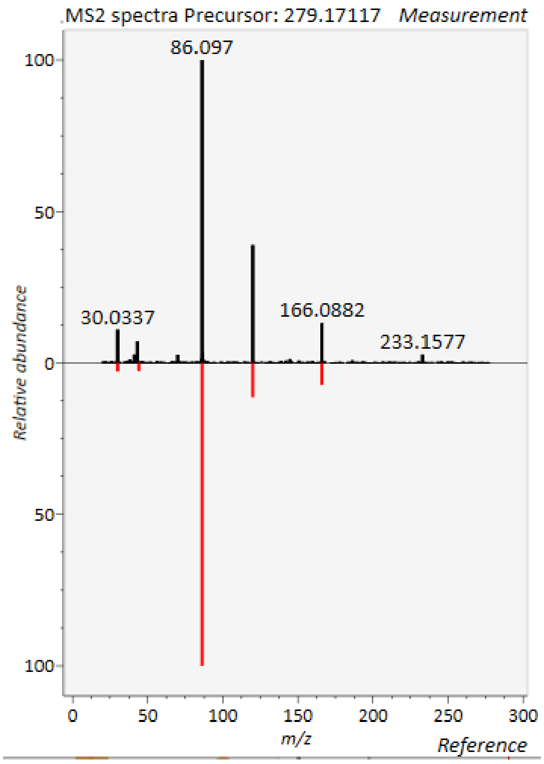

leu-tyr (2), RP pos, m/z 295.1657 (M+H)+, MW 294.1581, RT 2.60 min, 20V, HMDB28941

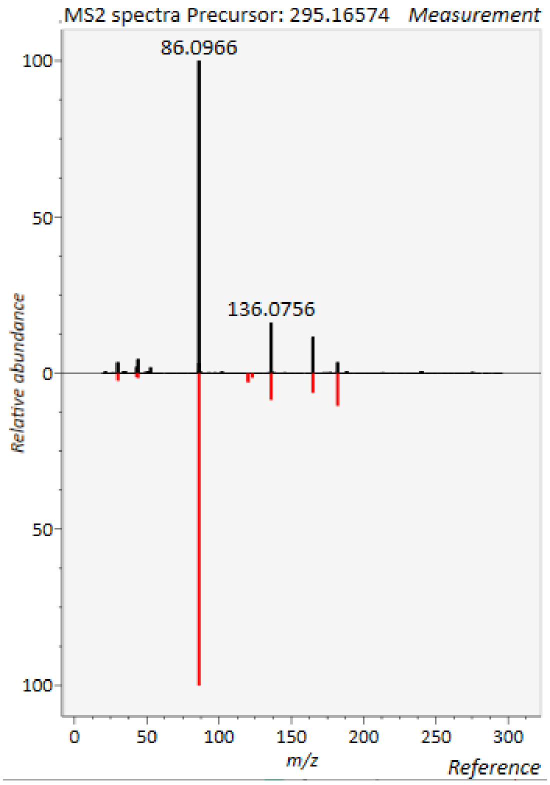

lys-phe (2), hilic pos, m/z 294.1819 (M+H)+, MW 293.1747, RT 6.13 min, 20V, HMDB28958

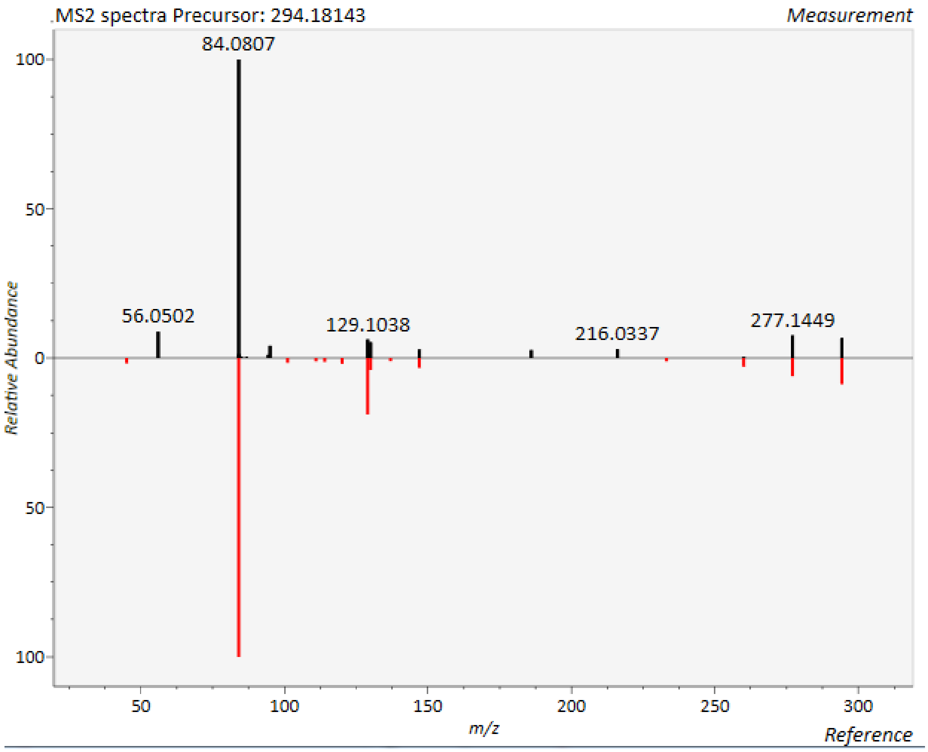

lys-pro (2), hilic pos, m/z 244.1657 (M+H)+, MW 243.1587, RT 6.97 min, 20V, HMDB28959

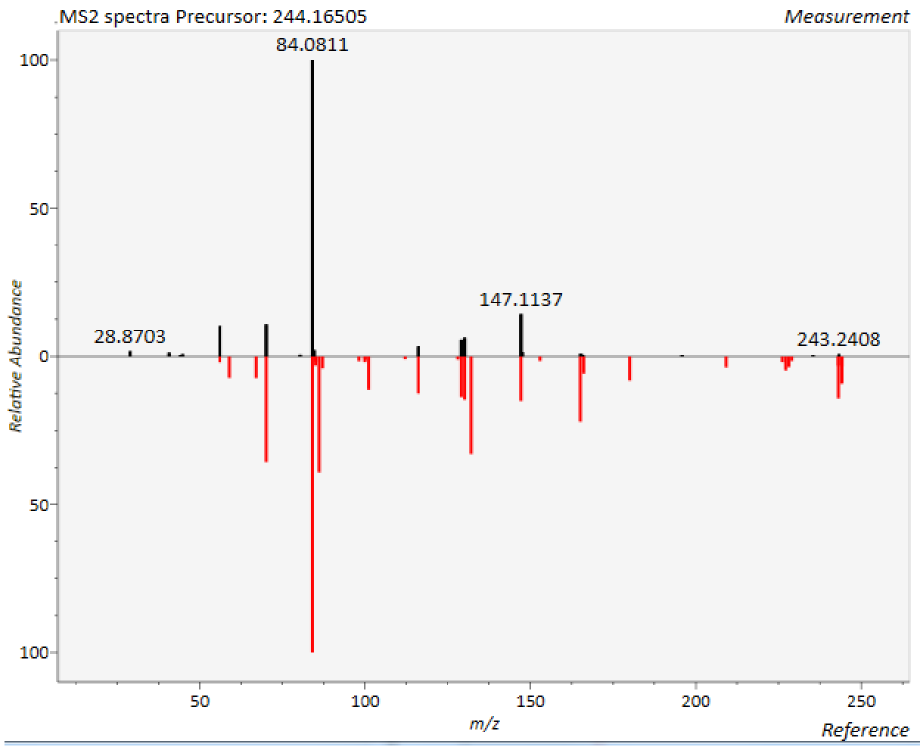

phe-his (2), hilic pos, m/z 303.1463 (M+H)+, MW 302.1381, RT 5.94 min, 20V, HMDB28997

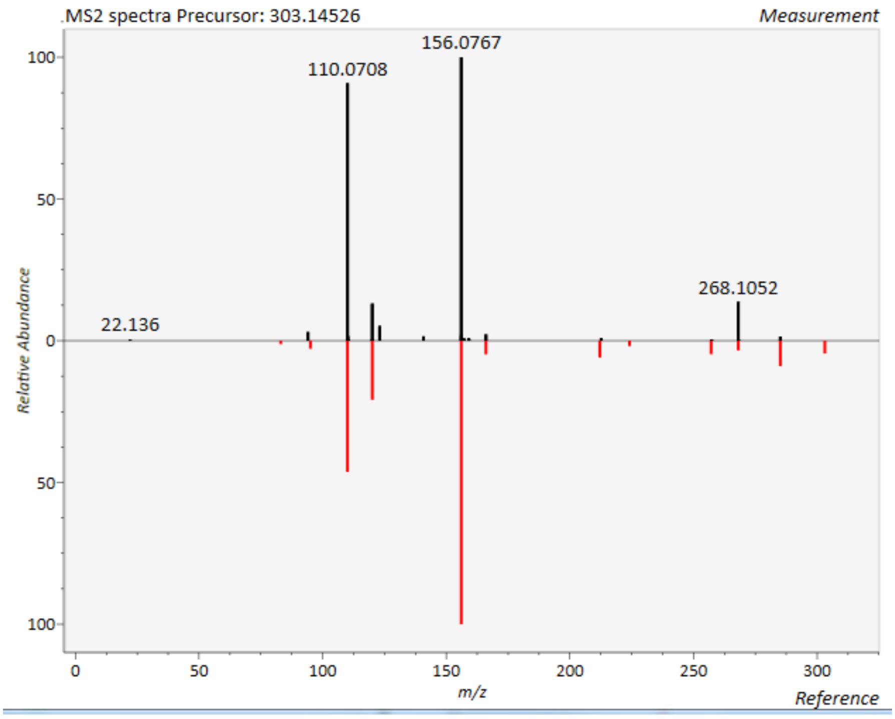

phe-ile/leu (2), RP pos, m/z 279.1709 (M+H)+, MW 278.1639, RT 3.73 min, 20V, phe-ile: HMDB28998
phe-leu: PubChem CID 76808

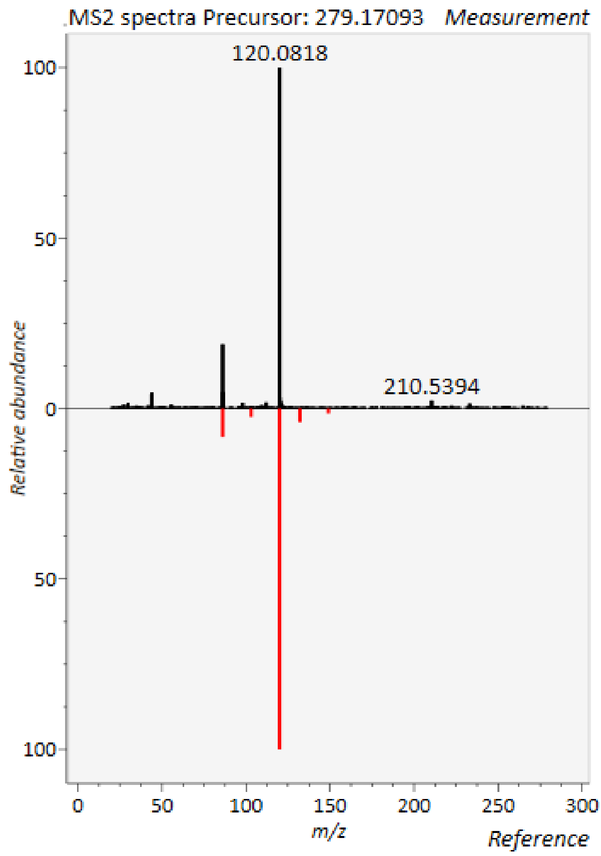

phe-ile-arg (2), hilic pos, m/z 435.2724 (M+H)+, MW 434.2642, RT 5.25 min, 20V,

PubChem CID 18223227

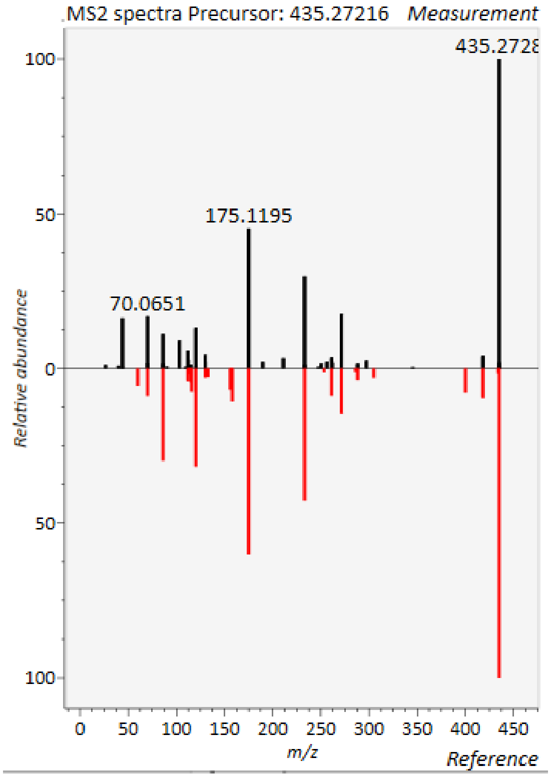

phe-phe (2), RP pos, m/z 313.1557 (M+H)+, MW 312.1477, RT 4.35 min, 20V, HMDB13302

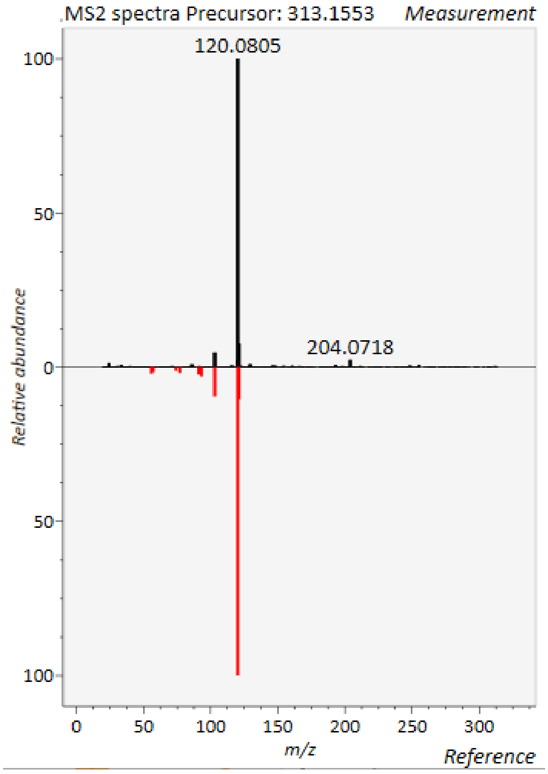

phe-tyr (2), RP pos, m/z 329.1503 (M+H)+, MW 328.1427, RT 3.04 min, 20V, HMDB29007

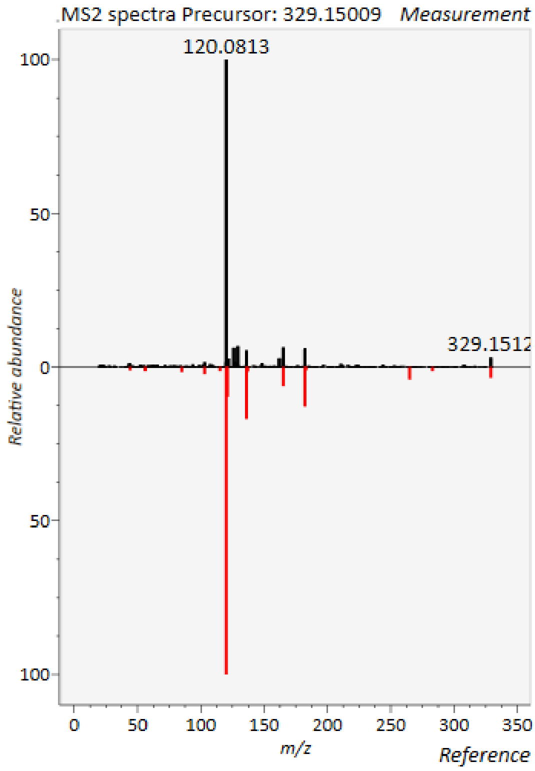

pro-leu (2), RP pos, m/z 229.1554 (M+H)+, MW 228.1477, RT 4.26 min, 20V, PubChem CID 444109

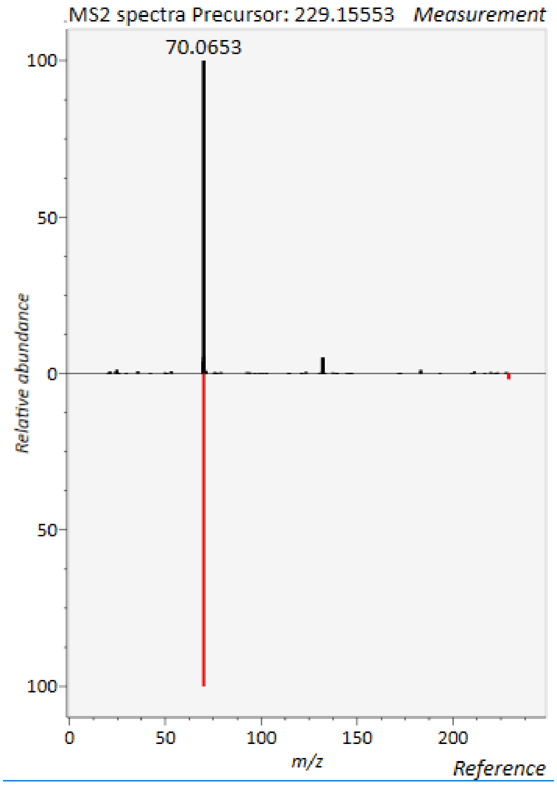

pyroglu-pro (2), RP pos, m/z 227.1027 (M+H)+, MW 226.0956, RT 1.73 min, 20V,

PubChem CID 13306588

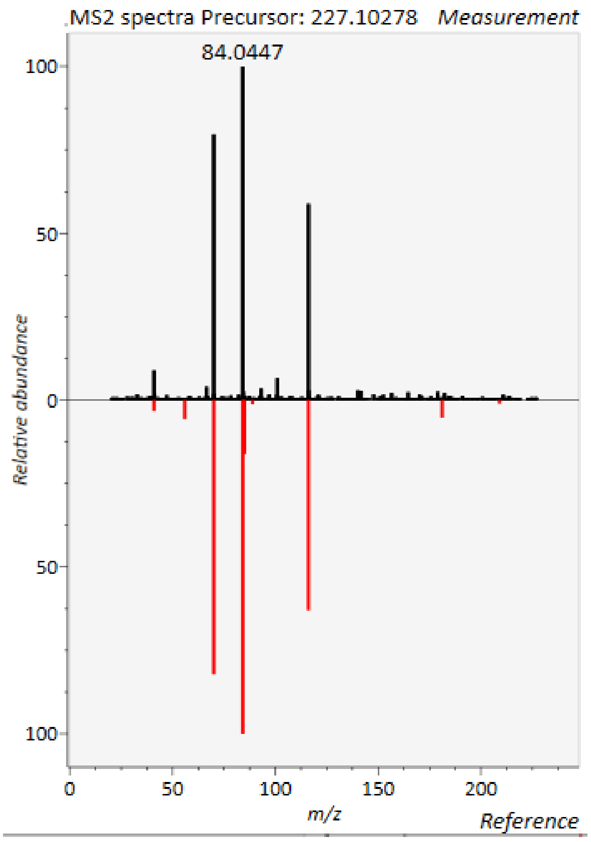

ser-ala (2), hilic pos, m/z 177.0873 (M+H)+, MW 176.0797, RT 6.20 min, 20V, HMDB29032

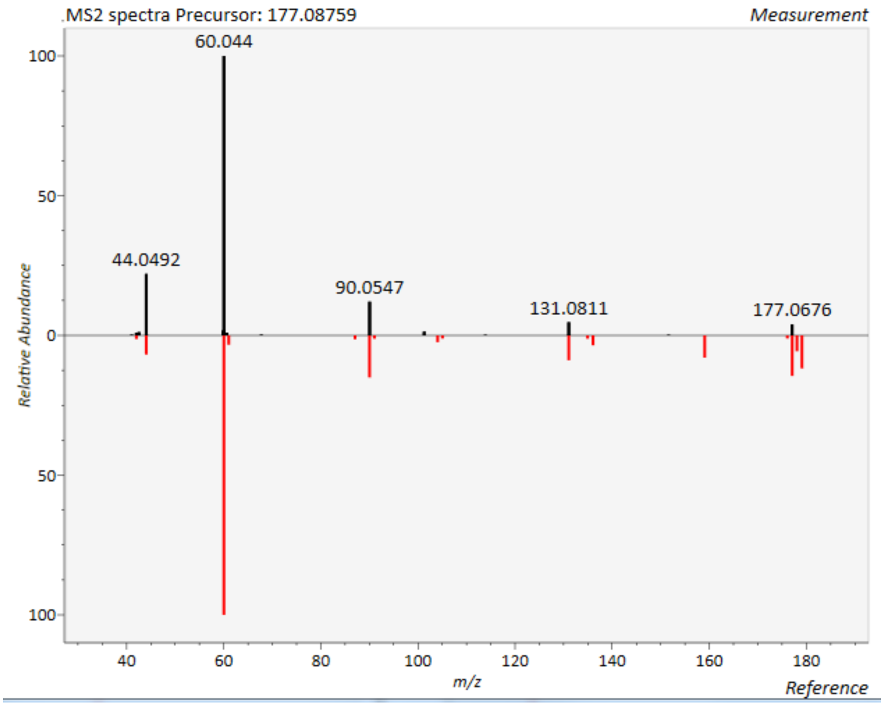

ser-ala-arg (2), hilic pos, m/z 333.1885 (M+H)+, MW 332.1811, RT 7.27 min, 20V,

PubChem CID 18223786

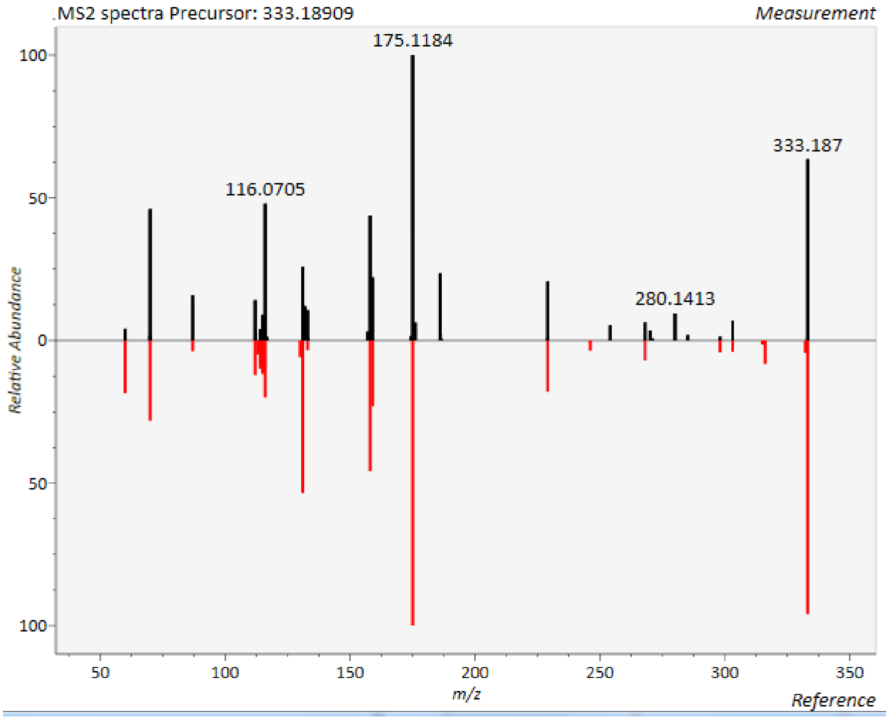

ser-gln (2), hilic pos, m/z 234.109 (M+H)+, MW 233.1014, RT 6.84 min, 40V, HMDB29037

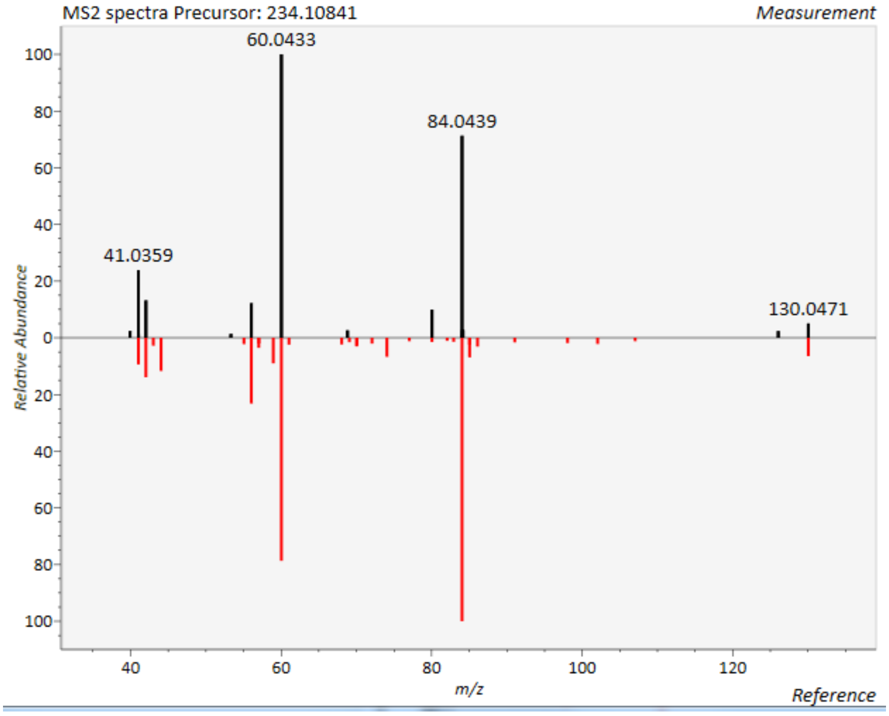

ser-leu (2), RP pos, m/z 219.1346 (M+H)+, MW 218.1265, RT 1.91 min, 20V, HMDB29043

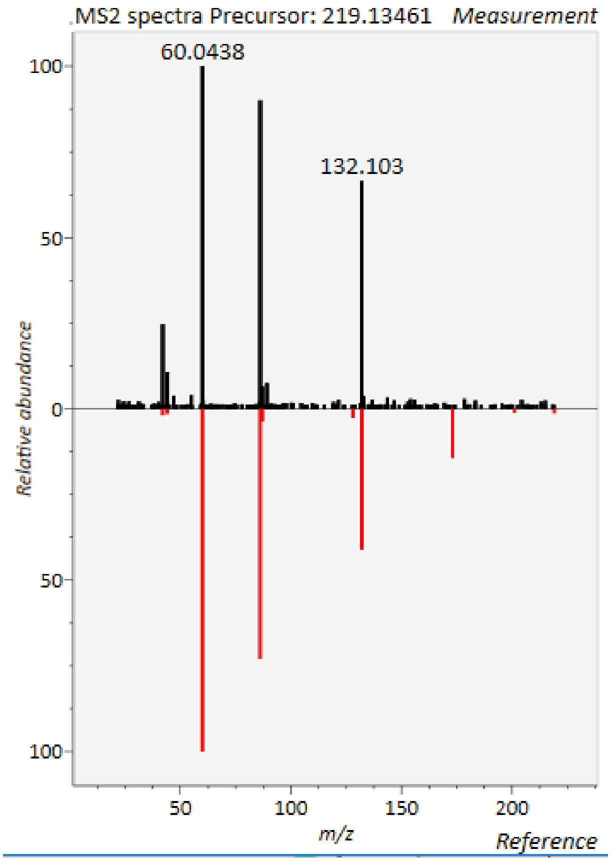

ser-pro (2), hilic pos, m/z 203.1032 (M+H)+, MW 202.0956, RT 6.04 min, 20V, HMDB29047

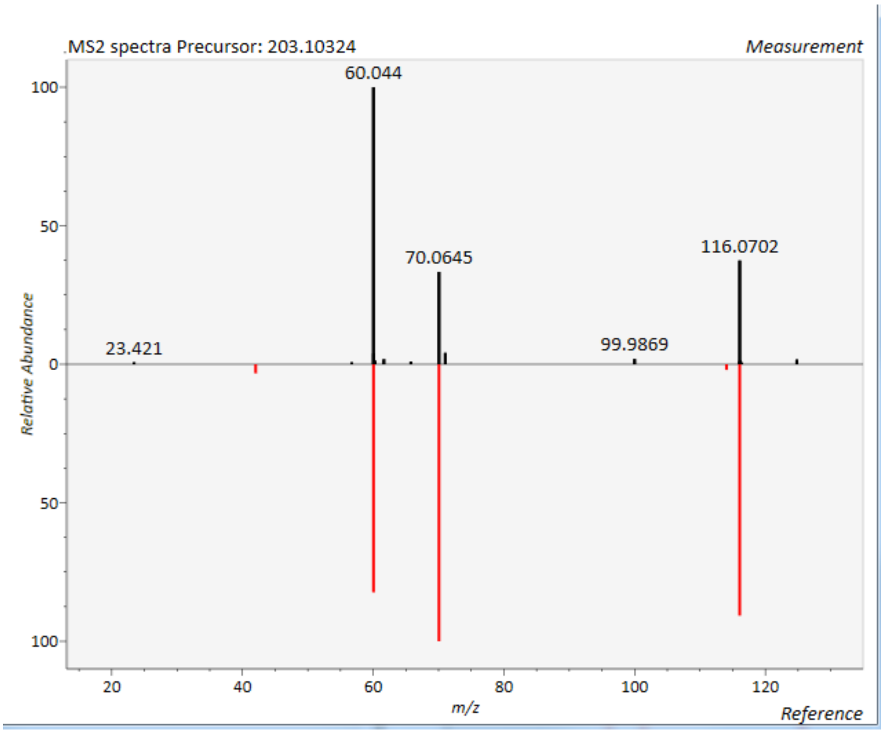

thr-phe (2), RP pos, m/z 267.1344 (M+H)+, MW 266.1274, RT 2.51 min, 20V, HMDB29068

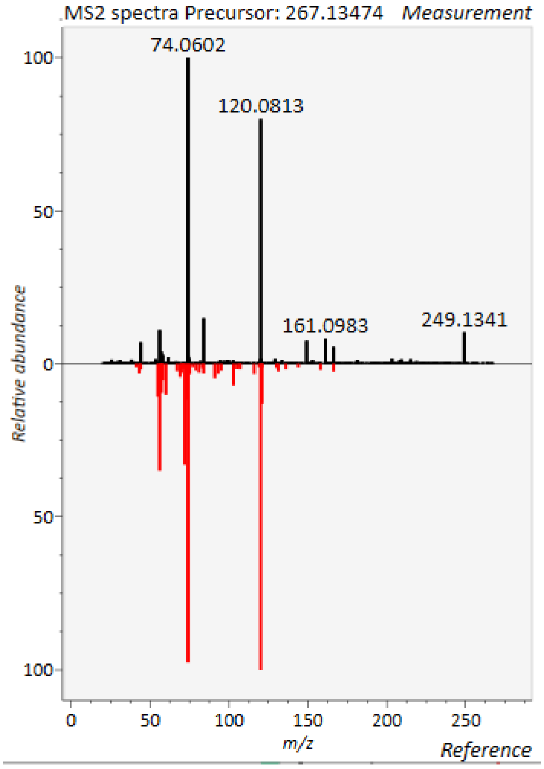

tyr-arg (2), hilic pos, m/z 338.1826 (M+H)+, MW 337.1749, RT 6.56 min, 20V, HMDB29099

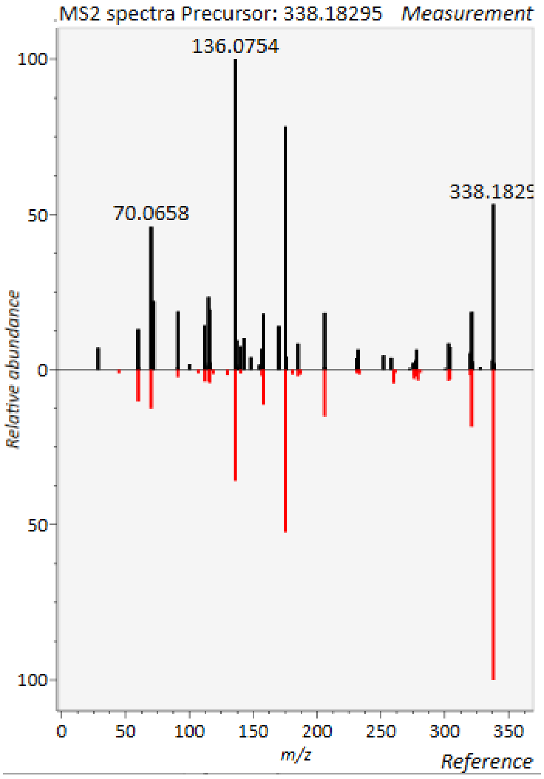

tyr-gly (2), hilic pos, m/z 239.1033 (M+H)+, MW 238.0961, RT 5.62 min, 20V, HMDB29105

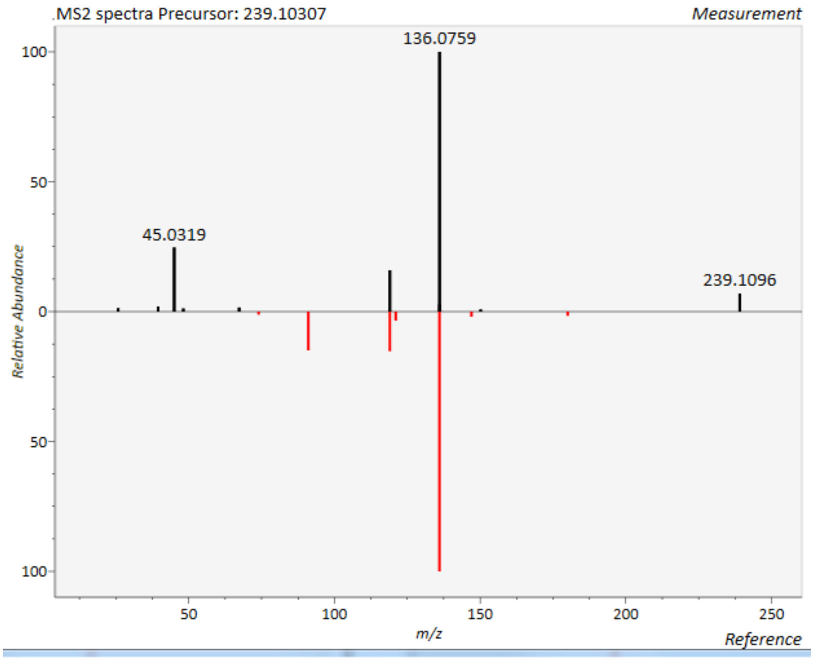

tyr-ile/leu (2), RP pos, m/z 295.166 (M+H)+, MW 294.1584, RT 3.02 min, 20V, tyr-ile: HMDB29108, tyr-leu: HMDB29109

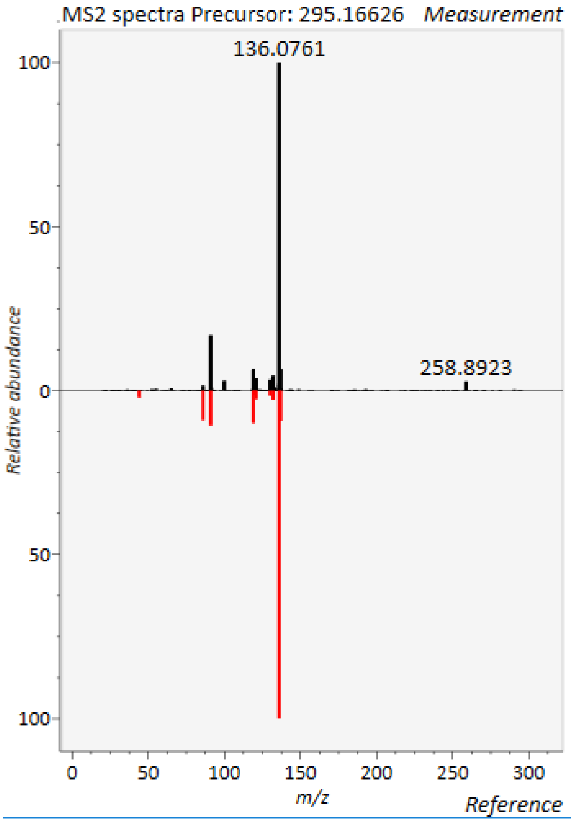

val-arg (2), hilic pos, m/z 274.1878 (M+H)+, MW 273.1811, RT 6.55 min, 20V, HMDB29121

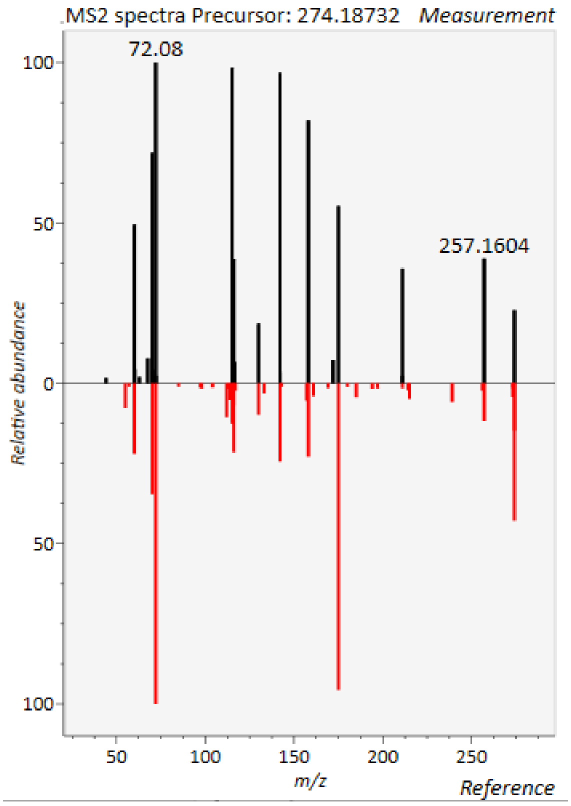

val-leu (2), hilic pos, m/z 231.1711 (M+H)+, MW 230.163, RT 2.75 min, 40V, HMDB29131

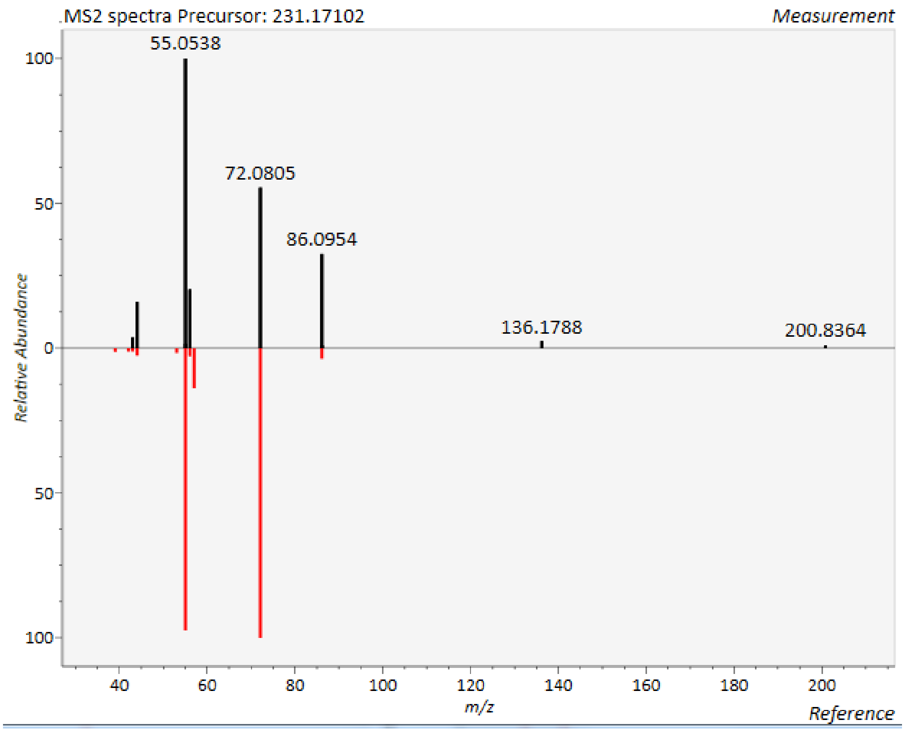

Supplement: Supplementary file 1 — Supplementary file1 (PDF 3325 kb) [file 11306_2020_1711_MOESM1_ESM.pdf]
