# Supplementary material for: Metabolome of canine and human saliva: a non-targeted metabolomics study
Source: Metabolomics. 2020 Aug 25;16(9):90. doi: 10.1007/s11306-020-01711-0 (PMC7447669; doi:10.1007/s11306-020-01711-0)
Supplement: Supplementary file 2 — Supplementary file2 (PDF 176 kb) [file 11306_2020_1711_MOESM2_ESM.pdf]

S2 Descriptive statistics of inter-individual and sample variation

Compound ID	Level of ID	DOG			HUMAN		
		Mean	SD	n	Mean	SD	n
**Amino acids**							
alanine	1	35065	16157	13	24230	8319	14
arginine	1	292938	213902	12	1489431	447851	14
aspartic acid	1	30027	14180	11	226772	102281	14
glutamic acid	1	407961	257382	13	224739	92767	14
glutamine	1	647844	303635	13	180241	76951	14
histidine	1	558554	376606	13	563633	238881	14
isoleucine	1	359034	229174	13	147429	62886	14
leucine	2	271917	125195	13	112620	46369	14
lysine	1	87199	56834	10	318715	131502	14
phenylalanine	1	244420	175784	13	614751	174399	14
proline	1	261675	134318	13	3976177	3076374	14
serine	2	116395	60612	13	44710	18924	12
threonine	1	133928	26257	10	25260	12162	14
tryptophan	1	41780	16710	12	22474	8173	5
tyrosine	1	26851	6484	11	212325	92235	14
**Amino acid derivatives**							
1-methyl-histidine	1	76906	40454	11	21900	10602	14
3-methyl-histidine	2	37350	19023	9	19375	12606	6
5-aminovaleric acid betaine	1	145624	112065	13	139903	126323	14
carnitine	1	1021016	549789	13	1917738	1102753	14
citrulline	1	400742	224042	13	369305	145733	14
creatine	1	2649085	1230360	13	1700864	494218	14
gamma glutamylglutamic acid	2	0	0	13	33655	27037	13
glycinebetaine	1	11080841	5479587	13	1241617	504031	14
ornithine	1	127780	106603	11	204923	83444	14
phenylacetylglycine	2	142737	114940	13	0	0	14
**Biogenic amines**							
asymmetric dimethylarginine	2	102591	49380	11	21326	7535	14
cadaverine	1	32920	26485	4	133957	106304	14
carnosine	1	137353	116957	10	14069		1
creatinine	1	4204018	2168685	12	2005721	372065	10
histamine	1	20555		1	62439	65065	12
spermidine	1	112876	134762	11	596	595	14
taurine	2	116903	64431	13	59969	18544	14
**Lipids and carnitines**							
acetylcarnitine	1	963922	756608	13	209886	155149	12
acylcarnitine C16:0	1	68916	33206	12	0	0	14
acylcarnitine C18:0	1	115687	76968	13	1280	785	14
acylcarnitine C18:1	2	90506	46482	13	0	0	14
azelaic acid	1	168481	95122	13	46004	5591	14
DAG 34:1	2	343687	241719	12	14244	4913	6
DAG 34:2	2	295504	255956	13	12893	2044	5
DAG 36:3	2	630344	523832	13	18128	6431	3
DAG 36:4	2	292573	284508	13	18895	2930	2
DAG 38:4	2	335782	285999	13	0	0	14
FA 15:0	2	377848	292690	13	66323	24722	14
FA 16:0	2	2231903	291047	13	2000694	183002	14
FA 16:1	2	1549532	1523042	13	291295	177097	14
FA 17:0	1	151880	73097	13	94333	27753	14
FA 17:1	2	143107	108703	13	35190	16365	14
FA 18:0	1	2297464	350401	13	2195405	326722	14

S2 Descriptive statistics of inter-individual and sample variation

Compound ID	Level of ID	DOG			HUMAN		
		Mean	SD	n	Mean	SD	n
FA 18:1	1	1977667	836715	13	583143	303394	14
FA 18:2	2	697020	244290	13	279117	203476	14
FA 18:3	2	63285	34098	12	28859	18151	9
FA 20:0	1	45449	13386	13	32248	10277	14
FA 20:1	2	172640	95177	13	20803	7991	14
FA 20:2	2	161645	82542	13	19957	10024	13
FA 20:3	2	130066	71513	13	42078	31377	14
FA 20:4	2	920036	448593	13	130923	118481	13
FA 20:5	2	88041	51910	13	20218	9928	7
FA 21:0	2	39009	37345	8	3227	683	14
FA 22:0	2	31169	10738	13	12280	3097	10
FA 22:1	1	117571	50645	13	11653	43	2
FA 22:2	2	62852	27494	12	2361	1002	14
FA 22:3	2	56328	46471	12	3691	1764	14
FA 22:4	2	99677	52877	13	17823	10383	6
FA 24:1 (n-9)	1	106958	47608	13	3616	1829	14
gamma-butyrobetaine	2	294875	220740	13	834362	488931	14
glycerophosphocholine	1	340518	256787	13	23768		1
hydroxypalmitic acid	2	213616	103388	13	13525	2604	8
isobutyryl carnitine	2	202439	234210	13	50626	23350	14
isovalerylcarnitine	1	147542	100722	13	88812	96779	14
leucic acid	2	26831	8033	11	61064	25243	14
LPC 16:0	2	3304221	4972704	13	13555	4181	4
LPC 16:1	2	76509	38422	9	0	0	14
LPC 18:0	2	1099281	1348888	13	26734	12999	3
LPC 18:1	2	632642	624970	12	1628	2640	14
LPC 18:2	2	237244	154814	13	1826	1036	14
LPE 16:0	2	97315	92667	13	2670	1572	14
LPE 16:1	2	128665	133178	12	0	0	14
LPE 18:0	2	308175	141617	13	12287		1
LPE 18:1	2	217724	121915	13	3004	1390	14
LPE 18:2	2	109043	70331	13	0	0	14
LPE 20:4	2	137991	108319	13	0	0	14
panthenol	2	109610	69931	8	2178	2291	14
PC 32:1 (16:0_16:1)	2	556559	476508	13	0	0	14
PC 32:1e (16:0e_16:1)	2	915489	548959	13	0	0	14
PC 34:1 (16:0_18:1)	2	1090549	482664	12	0	0	14
PC 34:2 (16:0_18:2)	1	1797939	655479	13	0	0	14
PC 34:2e (16:0e_18:2)	2	1673015	681399	11	0	0	14
PC 34:3 (16:1_18:2)	2	483543	353415	7	0	0	14
PC 36:2 (18:1_18:1)	2	1382896	611469	12	0	0	14
PC 36:3 (18:1_18:2)	2	2208218	1558741	12	0	0	14
PC 36:4	2	757695	722838	10	0	0	14
PC 38:4 (18:0_20:4)	2	1021051	425159	11	0	0	14
PC 38:5 (18:1_20:4)	2	508304	345871	11	0	0	14
PC 38:5e (18:1e_20:4)	2	1603092	1013764	11	4188	770	14
PE 28:0 (13:0_15:0)	2	164530	117355	12	0	0	14
PE 30:0 (15:0_15:0)	2	274410	187385	9	519	397	14
PE 32:1 (15:0-17:1)	2	212108	123272	13	14513		1
PE 32:2 (16:1_16:1)	2	148316	92082	13	0	0	14
PE 33:2 (15:0_18:2)	2	126460	64129	12	0	0	14
PE 34:2 (16:1_18:1)	2	374822	152472	10	13358		1

S2 Descriptive statistics of inter-individual and sample variation

Compound ID	Level of ID	DOG			HUMAN		
		Mean	SD	n	Mean	SD	n
PE 36:2 (18:1_18:1)	2	159231	63572	13	19097		1
PE 36:2e (18:1e_18:1)	2	131450	68246	13	13880	3175	2
PE 36:3 (18:1_18:2)	2	227712	61247	13	0	0	14
PE 36:3e (18:2e_18:1)	2	525163	318261	13	10947	3231	14
PE 36:4 (16:0_20:4)	2	226455	93890	13	0	0	14
PE 36:4e (16:0e_20:4)	2	459212	240460	13	12711	4358	3
PE 36:5e (16:1e-20:4)	2	233800	104402	13	16982	5299	10
PE 38:4 (18:0_20:4)	2	395798	162784	13	5457	2304	14
PE 38:4e (18:0e_20:4)	2	350719	184337	13	1794	814	14
PE 38:5 (18:1_20:4)	2	302168	142676	13	0	0	14
PE 38:5e (18:1e_20:4)	2	931469	515382	13	18679	5241	8
PE 38:6e (18:2e_20:4)	2	902824	458814	13	13180	407	4
propionylcarnitine	1	308906	202826	13	91310	57056	14
sebacic acid	2	32788	10374	13	17232	2818	14
suberic acid	2	43230	20358	13	15539	2373	14
TAG 36:0	2	133925	121891	9	82716	86496	6
TAG 38:0	2	75058	85352	8	46535	31840	3
**Nucleic acid subunits**							
2'-deoxy-cytidine	2	171354	176857	13	12157	750	2
adenine	1	489089	360887	13	233338	235232	14
adenosine	1	3462402	1414350	13	31839	26569	5
cytidine	1	437976	345512	13	82150	76581	7
cytosine	1	36120	22482	12	28117	31709	12
guanine	2	58874	51022	11	13326	2264	2
inosine	1	377034	252728	13	137433	166688	9
N6-methyl-adenine	2	26768	12023	8	55816	27354	14
**Organic acids**							
4-guanidinobutanoic acid	1	1755060	1455473	13	16864	4783	10
gamma-aminobutyric acid (GABA)	1	36516	17877	11	101765	144164	13
indoxyl sulfate	1	99625	95805	11	12160		1
lactic acid	2	135557	73967	12	50945	21448	10
pyrocatechol sulfate	2	1575331	1161467	13	0	0	14
succinic acid	1	140793	63018	13	447175	256345	14
**Other metabolites**							
1-methylnicotinamide	1	176640	120933	13	11940	1594	6
2-amino-1-phenylethanol	2	72221	27490	8	198721	63134	14
2-amino-2-methyl-1-propanol	2	192101	189967	12	22366	10867	6
3-indoleacetic acid	1	20433	9559	11	55963	44570	12
4-hydroxybenzaldehyde	1	47661	47620	12	13761		1
4-methylpyridine	2	2800	2459	13	44244	28406	9
5-aminovaleric acid	1	594572	405132	13	4334341	1914220	14
allantoin	1	287316	141143	13	82260	57824	14
caffeine	1	18970	4259	6	1041892	622177	14
choline	2	15678789	7210416	13	5453217	2359520	13
hydroxyphenyllactic acid	1	54443	30915	12	57885	23596	14
kynurenic acid	1	384322	247988	13	782	406	14
N-acetylgalactosamine 4-sulfate	2	68490	35432	12	27636	9649	4
N-acetylglucosamine	2	18666	10204	3	526247	416014	14
N-acetylneuraminic acid	1	66451	46749	12	485899	514042	14
nicotinic acid	1	81804	44940	12	43642	31275	11
pantothenic acid	1	56650	31102	12	5004	2443	11
paraxanthine	1	22369	9743	5	419424	256939	14

S2 Descriptive statistics of inter-individual and sample variation

Compound ID	Level of ID	DOG			HUMAN		
		Mean	SD	n	Mean	SD	n
phosphocholine	1	46810	28850	5	358497	270997	13
purine	2	1542713	1073549	13	132732	35577	10
quinaldic acid	2	49473	51439	12	0	0	14
riboflavin	1	35233	16450	9	1996	1069	13
sphinganine	1	73277	32909	13	0	0	14
sphingosine	2	160477	68385	13	0	0	14
theobromine	1	2645	1932	12	88418	52465	14
trigonelline	1	91200	66563	12	646219	544769	14
urea	2	137280	23206	6	107003	30822	14
urocanic acid	1	403894	162424	12	36386	16273	14
usnic acid	2	160373	231121	9	0	0	14
xanthine	1	57365	38594	12	173813	123732	14
**Small peptides**							
arg-ile	2	25010	7305	11	320444	215988	14
arg-phe	2	76271	79286	12	781501	373392	14
arg-ser	2	2177	1395	13	653047	308815	14
gly-pro	2	10276	6605	13	317455	177859	14
gly-tyr	2	0	0	13	91261	64126	14
his-glu	2	0	0	13	73938	55262	14
his-gly	2	1524	1113	13	113059	83707	14
his-his	2	0	0	13	53110	31105	14
his-ile/leu	2	13308	2067	8	33166	17330	8
his-ser	2	655	506	13	279670	120578	14
ile-ser	2	1481	907	13	40065	22717	13
leu-leu	2	26635	12610	9	405343	301780	14
leu-phe	2	12514		1	136678	72849	14
leu-tyr	2	776	656	13	69066	43098	13
lys-phe	2	2763	1515	13	196044	88265	14
lys-pro	2	0	0	13	54790	46774	8
phe-his	2	1120	882	13	357211	214378	14
phe-ile/leu	2	2636	1206	13	35793	41934	7
phe-ile-arg	2	0	0	13	63662	52243	9
phe-phe	2	883	824	13	32422	12970	14
phe-tyr	2	33854	21300	9	76610	69517	11
pro-leu	2	0	0	13	103586	86414	13
pyroglu-pro	2	2045	1682	13	41311	32980	11
ser-ala	2	9166	2708	13	144653	107199	10
ser-ala-arg	2	0	0	13	236955	142975	14
ser-gln	2	0	0	13	93367	47091	14
ser-leu	2	11902	1732	3	72616	49118	12
ser-pro	2	24637	6138	7	311551	266567	14
thr-phe	2	369	324	13	413981	224576	14
tyr-arg	2	16020	5081	2	262863	270167	14
tyr-gly	2	903	548	13	108972	52709	14
tyr-ile/leu	2	1533	1182	13	84837	45972	14
val-arg	2	18446	9548	5	40493	28688	14
val-leu	2	20128	7892	8	320566	283561	14
**Chemicals**							
dibutyl adipate	2	162098	163050	13	73783	33110	14
diethanolamine	1	56527	34354	13	147588	168396	13
diethylhexyl adipate	2	2090268	2341180	13	249685	403937	14
diisodecyl phthalate	2	500679	524875	13	225726	9688	14

S2 Descriptive statistics of inter-individual and sample variation

Compound ID	Level of ID	DOG			HUMAN		
		Mean	SD	n	Mean	SD	n
dioctyl phthalate	1	2467163	2020371	13	277037	17334	14
dodecyl sulfate	2	1115358	769462	13	3650004	7175356	14
dodecylbenzenesulfonic acid	2	939998	444371	13	216457	141432	14
linoleamide	2	34265418	16340617	13	30287081	4290249	14
myristamide	2	4497535	2392395	13	4427290	838115	14
oleamide	2	93856780	26402347	13	85188588	7996398	14
palmitoleamide	2	13992187	6348206	13	12508377	1779147	14
pentaerythritol tetrakis(3,5-di-tert-butyl-4-hydroxyhydrocinnamate)	2	94073	54746	13	97419	36912	14
phthalic acid mono-2-ethylhexyl ester	2	77815	12939	13	132858	18364	14
stearamide	2	10780862	5148519	13	10065816	1374684	14
triethanolamine	2	621250	1258590	10	53520	57605	13
tris(hydroxymethyl)aminomethane	2	198996	60421	11	183391	36688	12

Legend: n, number of samples where metabolite was detected. Level of identification (ID) based on Sumner et al. (2007).
