# Supplementary material for: Metabolome of canine and human saliva: a non-targeted metabolomics study
Source: Metabolomics. 2020 Aug 25;16(9):90. doi: 10.1007/s11306-020-01711-0 (PMC7447669; doi:10.1007/s11306-020-01711-0)
Supplement: Supplementary file 3 — Supplementary file3 (PDF 152 kb) [file 11306_2020_1711_MOESM3_ESM.pdf]

Turunen et al.

Supplementary Materials

METABOLOME OF CANINE AND HUMAN SALIVA: A NON-TARGETED METABOLOMICS STUDY

Soile Turunen[1]*, Jenni Puurunen[2,3], Seppo Auriola[1], Arja M Kullaa[4], Olli Kärkkäinen[1], Hannes Lohi[2,3], Kati Hanhineva[5]

[1]School of Pharmacy, Faculty of Health Sciences, University of Eastern Finland, Kuopio, Finland

[2]Department of Veterinary Biosciences, and Department of Medical and Clinical Genetics, University of Helsinki, Helsinki, Finland

[3]Folkhälsan Research Center, Helsinki, Finland

[4]Institute of Dentistry, School of Medicine, Faculty of Health Sciences, University of Eastern Finland, Kuopio, Finland

[5]Institute of Public Health and Clinical Nutrition, Faculty of Health Sciences, University of Eastern Finland, Kuopio, Finland

*corresponding author Soile Turunen, soiru@uef.fi, +358503455549

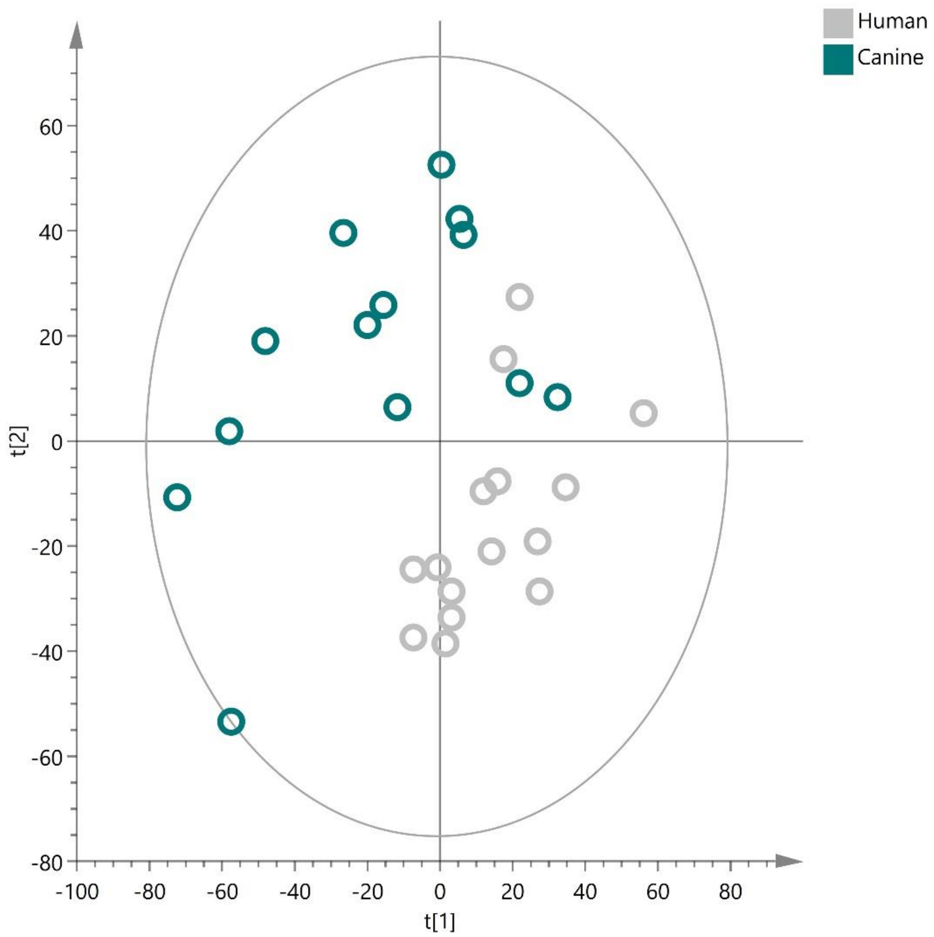

Supplementary figure S3: Principal component analysis.

Two first latent components of the principal component analysis are shown. Separation between metabolite profile of canine and human saliva samples is observed.
