# Supplementary material for: Metabolome of canine and human saliva: a non-targeted metabolomics study
Source: Metabolomics. 2020 Aug 25;16(9):90. doi: 10.1007/s11306-020-01711-0 (PMC7447669; doi:10.1007/s11306-020-01711-0)
Supplement: Supplementary file 4 — Supplementary file4 (PDF 374 kb) [file 11306_2020_1711_MOESM4_ESM.pdf]

Turunen et al.

Supplementary Materials

METABOLOME OF CANINE AND HUMAN SALIVA: A NON-TARGETED METABOLOMICS STUDY

Soile Turunen[1]*, Jenni Puurunen[2,3], Seppo Auriola[1], Arja M Kullaa[4], Olli Kärkkäinen[1], Hannes Lohi[2,3], Kati Hanhineva[5]

[1]School of Pharmacy, Faculty of Health Sciences, University of Eastern Finland, Kuopio, Finland

[2]Department of Veterinary Biosciences, and Department of Medical and Clinical Genetics, University of Helsinki, Helsinki, Finland

[3]Folkhälsan Research Center, Helsinki, Finland

[4]Institute of Dentistry, School of Medicine, Faculty of Health Sciences, University of Eastern Finland, Kuopio, Finland

[5]Institute of Public Health and Clinical Nutrition, Faculty of Health Sciences, University of Eastern Finland, Kuopio, Finland

*corresponding author Soile Turunen, soiru@uef.fi, +358503455549

A

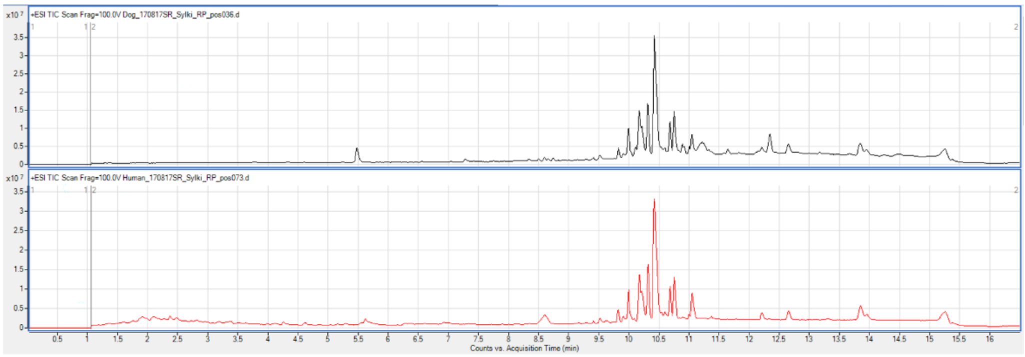

B

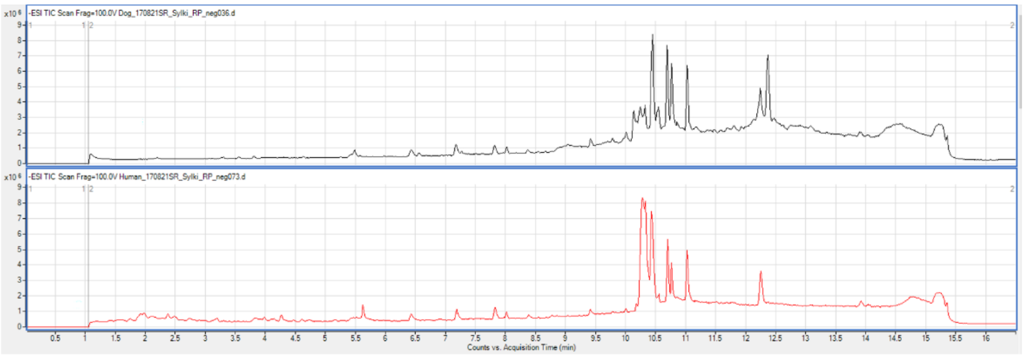

C

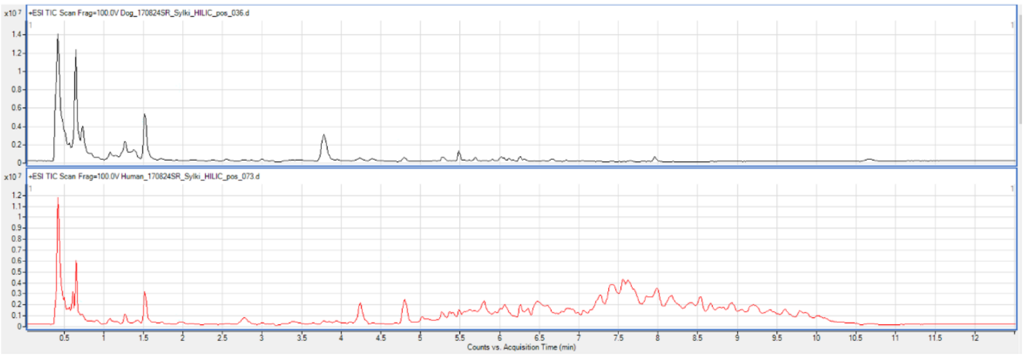

D

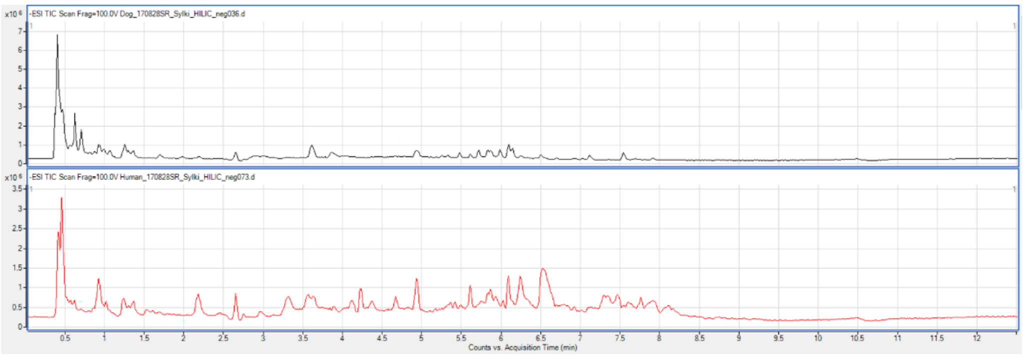

S4 Total ion chromatograms of dog and human saliva in four analytical modes.

Examples of total ion chromatograms are shown for saliva samples from dogs (black line) and humans (red line) for all four different analytical modes: reverse phase positive mode (A) and negative mode (B), and hydrophilic interaction chromatography positive mode (C) and negative mode (D).
